# Association between human blood metabolome and the risk of delirium: a Mendelian Randomization study

**DOI:** 10.3389/fendo.2023.1332712

**Published:** 2024-01-11

**Authors:** Chubing Long, Dong Lin, Lieliang Zhang, Yue Lin, Qing Yao, Guangyong Zhang, Longshan Li, Hailin Liu, Jun Ying, Xifeng Wang, Fuzhou Hua

**Affiliations:** ^1^ Department of Anesthesiology, The Second Affiliated Hospital of Nanchang University, Nanchang, Jiangxi, China; ^2^ Key Laboratory of Anesthesiology of Jiangxi Province, Department of Anesthesiology, The Second Affiliated Hospital of Nanchang University, Nanchang, Jiangxi, China; ^3^ Department of Anesthesiology, The First Affiliated Hospital of Nanchang University, Nanchang, Jiangxi, China

**Keywords:** metabolites, delirium, Mendelian Randomization, genome-wide association study, phenotype-wide study

## Abstract

**Background:**

Delirium significantly contributes to both mortality and morbidity among hospitalized older adults. Furthermore, delirium leads to escalated healthcare expenditures, extended hospital stays, and enduring cognitive deterioration, all of which are acknowledged detrimental outcomes. Nonetheless, the current strategies for predicting and managing delirium remain constrained. Our aim was to employ Mendelian randomization (MR) to investigate the potential causal relationship between metabolites and delirium, as well as to identify potential therapeutic targets.

**Methods:**

We identified 129 distinct blood metabolites from three genome-wide association studies (GWASs) conducted on the metabolome, involving a total of 147,827 participants of European descent. Genetic information pertaining to delirium was sourced from the ninth iteration of the Finngen Biobank, encompassing 359,699 individuals of Finnish ancestry. We conducted MR analyses to evaluate the connections between blood metabolites and delirium. Additionally, we extended our analysis to encompass the entire phenome using MR, aiming to uncover potential on-target consequences resulting from metabolite interventions.

**Results:**

In our investigation, we discovered three metabolites serving as causal mediators in the context of delirium: clinical low density lipoprotein cholesterol (LDL-C) (odds ratio [OR]: 1.47, 95% confidence interval [CI]: 1.25-1.73, *p* = 3.92 x 10^-6^), sphingomyelin (OR: 1.47, 95% CI: 1.25-1.74, *p* = 5.97 x 10^-6^), and X-11593–O-methylascorbate (OR: 0.21, 95% CI: 0.10-0.43, *p* = 1.86 x 10^-5^). Furthermore, utilizing phenome-wide MR analysis, we discerned that clinical LDL-C, sphingomyelin, and O-methylascorbate not only mediate delirium susceptibility but also impact the risk of diverse ailments.

**Limitations:**

(1) Limited representation of the complete blood metabolome, (2) reliance on the PheCode system based on hospital diagnoses may underrepresent conditions with infrequent hospital admissions, and (3) limited to European ancestry.

**Conclusion:**

The genetic prediction of heightened O-methylascorbate levels seems to correspond to a diminished risk of delirium, in contrast to the association of elevated clinical LDL-C and sphingomyelin levels with an amplified risk. A comprehensive analysis of side-effect profiles has been undertaken to facilitate the prioritization of drug targets. Notably, O-methylascorbate emerges as a potentially auspicious target for mitigating and treating delirium, offering the advantage of lacking predicted adverse side effects.

## 1 Introduction

Delirium ranks as the predominant psychiatric syndrome encountered among hospitalized older adults subsequent to acute illness or surgical procedures (1). Clinically, delirium is marked by sudden regressions and variations in attention, cognitive capabilities, and impaired consciousness (1). Delirium is connected to protracted hospitalization and escalated expenditures, elevated morbidity and mortality rates, the decline of cognitive faculties, the development of dementia, and a more unfavorable overall prognosis (2–4). A range of prominent mechanisms hypothesized to play a role in delirium encompass neurotransmitter imbalances, inflammation, physiological stress, metabolic dysregulation, electrolyte imbalances, and genetic factors (5, 6). Given the limited effectiveness of medications primarily employed for symptom management and addressing underlying conditions (7), there is a pressing necessity to identify risk factors amenable to modification and potential targets for therapeutic intervention.

The human metabolome not only serves as a repository of disease biomarkers but also encompasses an individual’s metabolic signature (8, 9). Furthermore, metabolomics holds the potential to influence cellular physiology by orchestrating various interactions across the genome, epigenome, transcriptome, and proteome (10). Consequently, it offers a more profound understanding of the foundational mechanisms governing diseases (11, 12). The latest strides in mass spectrometry and high-throughput genotyping technologies have empowered the execution of GWASs with a comprehensive scope, unearthing the genetic factors that govern the metabolome (13–15). This progress opens up a promising avenue to precisely discern potential drug targets for delirium treatment. This is accomplished by harmoniously merging genomics and metabolomics data within the framework of MR designs.

MR constitutes a burgeoning analytical approach that leverages genetically determined variations linked to an exposure, serving as instrumental variants to gauge the plausible causal impact of said exposure on ensuing outcomes (16). Notably, alleles undergo random assignment during gamete formation, rendering MR akin to a “natural” randomized controlled trial that effectively mitigates the impact of confounding and reverse causality biases (17). Previously, MR designs have been employed to evaluate the impact of specific illnesses, biomarkers, and gut flora on the susceptibility to delirium (18–20). To date, there has been no systematic MR analysis of the human metabolome to search for promising etiological mediators of delirium or to identify potential drug targets for delirium treatment. Phenotype-wide MR (Phe-MR) analysis additionally possesses the capability to unveil potential side effects associated with conceivable drug targets prior to embarking on clinical trials. Consequently, our initial step encompassed an expansive large-scale two-sample MR analysis with the aim of meticulously scrutinizing 129 circulating metabolites in search of conceivable causative mediators of delirium. Subsequently, we expanded our analytical horizon by conducting Phe-MR analysis involving 678 distinct disease attributes. This endeavor aimed to anticipate target-linked side effects stemming from metabolite interventions, thereby providing a comprehensive evaluation of their clinical safety (21).

## 2 Methods

### 2.1 Data source for blood metabolites, delirium, and disease traits

Summary-level data of single nucleotide polymorphisms (SNPs) associated with the human metabolome, serving as genetic instruments, were extracted from three extensive GWASs encompassing a total of 147,827 individuals of European descent (**Table 1**) (13–15). Briefly, Shin et al. scrutinized 453 metabolic traits in a cohort of 7,824 participants (15), employing roughly 3 million SNPs and a Metabolon assay; Kettunen et al. examined 123 metabolic traits across 24,925 participants (14), employing nearly 12 million SNPs and a nuclear magnetic resonance assay; whereas Borges et al. explored 249 metabolic traits in a cohort of 115,078 participants (13), harnessing approximately 12 million SNPs via the Nightingale Health assay (**Table 1**). The public databases containing the aforementioned metabolite data were accessible through the IEU GWAS database (https://gwas.mrcieu.ac.uk/). Following the exclusion of redundant metabolites identified in these three metabolome GWASs, a total of 469 metabolites were retained.

**Table 1:** Characteristics of human blood metabolites GWASs used for genetic instruments selection.

References	Study Cohort (s)	Cohort Description	GWAS Sample Size	Population		Number of Human Blood Metabolites Analyzed	Blood Fraction Test
**Shin et al.** **(2014)** (15)	TwinsUK	An adult twin British registry cohort studis	7,824	German	European	453	Serum; Plasma
	KORA	Population-based cohort studies		British			
**Kettunen et al. (2016)** (14)	COROGENE	Case–control study, only controls used	24,925	Finnish	European	123	Serum; Plasma
	EGCUT	Population-based cohort study		Estonian			
	ERF	Family-based study		Dutch			
	FR97	Population-based cohort study		Finnish			
	GenMets	Case–control study		Finnish			
	HBCS	Birth cohort study		Finnish			
	KORA-F4	Population based cohort study		German			
	LLS	Family-based study		Dutch			
	NFBC 1966	Birth cohort study		Finnish			
	NTR	Population based twin study		Dutch			
	Predict CVD	Cohort study		Finnish			
	Prote	Population based study		Estonian			
	Twins	Population based twin study		Finnish			
	YFS	Follow up study in children		Finnish			
**Borges et al. (2020)**	UK Biobank	Population-based study	115,078	European		249	Blood
**Total**			147,827			825	

COROGENE, Genetic Predisposition of Coronary Heart Disease in Patients Verified with Coronary Angiogram; EGCUT, Estonian Genome Center of University of Tartu Cohort; ERF, Erasmus Rucphen Family Study; FR97, a subsample of FINRISK 1997; GenMets, Genetics of METabolic Syndrome; HBCS, Helsinki Birth Cohort Study; KORA, kooperative gesundheit Forschung in the region Augsburg; LLS, Leiden Longevity Study; GWAS, genome-wide association study; NFBC 1966, Northern Finland Birth Cohort 1966; NTR, Netherlands Twin Register; PredictCVD, FINRISK subsample of incident cardiovascular cases and controls; Prote, EGCUT 2nd cohort; Twins, Finnish Twin Cohort; YFS, The Cardiovascular Risk in Young Finns Study.

We acquired GWAS summary data pertaining to delirium from the FinnGen Consortium. This dataset comprised 3,039 cases and 356,660 controls of Finnish ancestry, resulting in the identification of approximately 20 million SNPs. The definition of delirium relied on the International Classification of Diseases, 10th Revision (ICD-10).

Summary statistics for 1,403 disease traits were sourced from Zhou et al.’s GWAS, which included 408,961 participants of white British ancestry and 28 million SNPs in the UK Biobank cohort (ICD-9) (https://www.leelabsg.org/resources) (22). Ethical approval for the protocol and data collection was granted by the ethics committee overseeing the original GWASs, and written informed consent was diligently secured from every participant prior to data collection. The study is reported following the Strengthening the Reporting of Observational Studies in Epidemiology using Mendelian Randomization (STROBE-MR) statement (23).

### 2.2 Genetic instruments for blood metabolites

In this MR study, we utilized SNPs that had been identified as being significantly associated with blood metabolites in previously published GWASs at the stringent threshold of genome-wide significance (*p* < 5 x 10^-8^). These selected SNPs were specifically chosen for their lack of linkage disequilibrium (LD) with other SNPs (r2 < 0.1, clump window 500 kb), ensuring their independence as instrumental variants for the respective metabolites. Additionally, certain sensitivity analyses in the MR framework necessitated the inclusion of a minimum of three SNPs as genetic instruments for exposure variants (24). Consequently, metabolites associated with fewer than three SNPs were excluded from the analysis. Weak SNPs were excluded based on F-statistics (F < 10).

**Figure 1:**
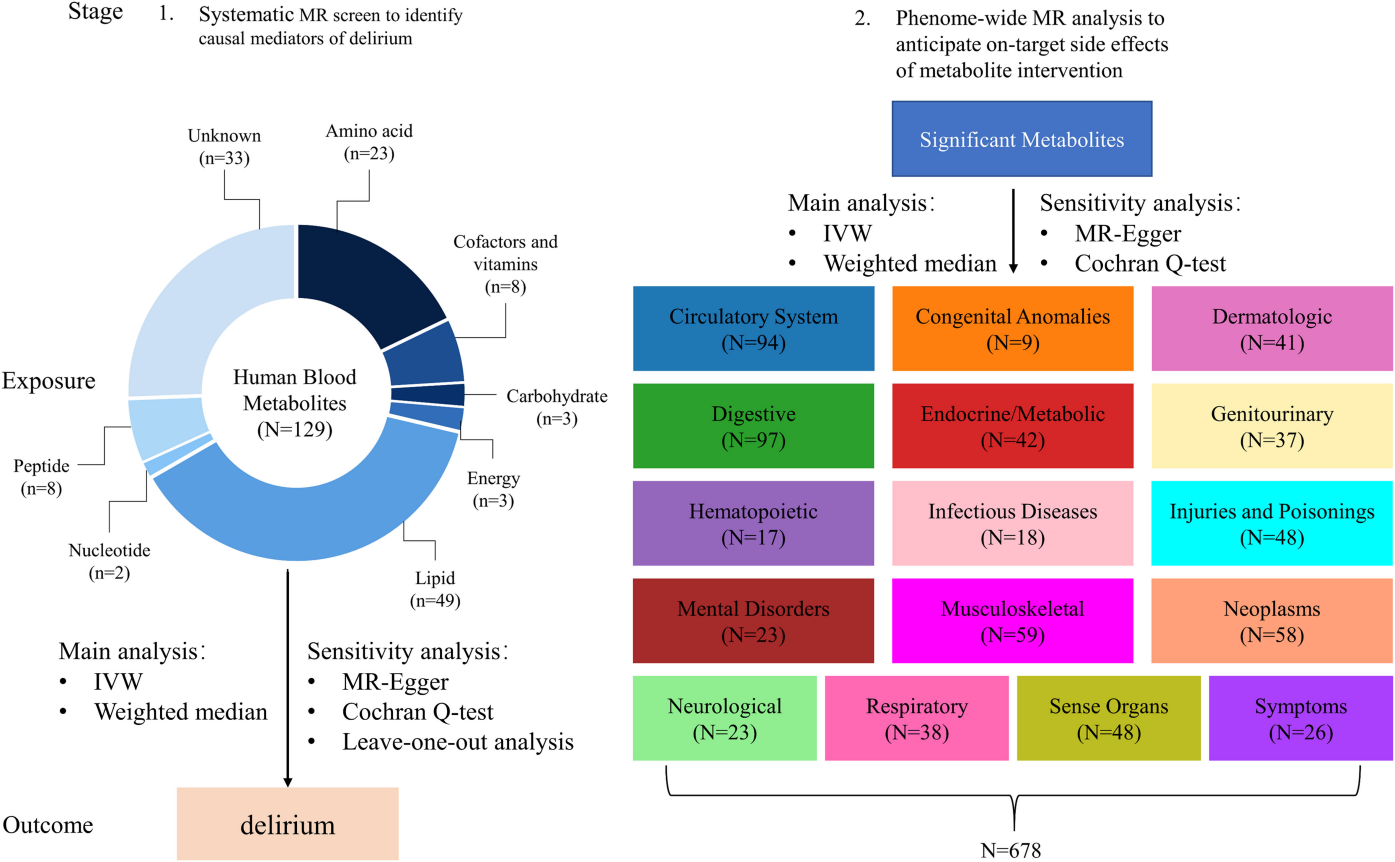
Conceptual framework of two-stage MR study. The study employs a two-stage design rooted in MR principles, as illustrated in this figure. In the initial stage, we thoroughly assessed the causal connections between 129 distinct blood metabolites and the susceptibility to delirium. Advancing to the subsequent stage, we extensively explored a wide spectrum of potential side effects arising from targeting the identified metabolites within the context of 678 non-delirium diseases. It’s important to highlight that each of these diseases falls under one of the 16 distinct chapters of ICD-9. Throughout both stages, we meticulously applied a Bonferroni-corrected p-value threshold, taking into account the multitude of metabolites and diseases subjected to analysis.

After removing metabolites with less than three associated SNPs and excluding weak SNPs, a total of 340 out of the initial 469 metabolites were excluded from the analysis. Consequently, only 129 distinct blood metabolites met the inclusion criteria and were subsequently utilized in the MR analysis (**Figure 1**). For a simplified overview of the SNPs data used as instruments in this MR study, please refer to **Supplementary Table 1**, while more comprehensive information can be found in **Supplementary Tables 2, 3**.

### 2.3 MR assumptions and statistical analyses

**Figure 1** illustrates the conduct of a two-stage MR study with the aim of systematically identifying novel and secure drug targets for delirium. MR design relies on three fundamental assumptions: (1) genetic variants exert a direct impact on exposures, (2) genetic variants exhibit no associations with potential confounders, and (3) genetic variants exclusively influence outcomes through their effects on exposures (25). The summary-level data concerning the blood metabolome, delirium, and 678 non-delirium diseases utilized in this study were sourced from publicly available GWASs involving individuals of European descent (13–15, 22).

In our primary analysis, we employed two distinct MR methods, namely the inverse-variance weighted (IVW) MR method (26) and the weighted median method (27), to assess the relationships between 129 blood metabolites and the risk of delirium. To examine potential heterogeneity among genetic instruments, we conducted Cochran’s Q test (28). If significant heterogeneity was detected, we employed a random-effect IVW model; otherwise, a fixed-effect IVW model was used. To ensure the robustness of the associations identified in the IVW analysis, we conducted a sensitivity analysis using the MR Egger method (29). We also conducted a leave-one-out analysis to perform sensitivity analysis, where we systematically excluded individual SNPs one at a time and calculated the remaining SNP effects. This was done to account for potential violations of the assumptions underlying the MR design. Additionally, we conducted a specific assessment of potential directional pleiotropy through the intercept term in the MR-Egger method (29).

We conducted Phe-MR analysis to assess potential on-target side effects associated with hypothetical interventions aimed at reducing delirium risk by targeting the identified metabolites. Disease traits were categorized using “Phecodes”, a system designed to systematically integrate International Classification of Diseases and Related Health Problems codes into phenotypic outcomes suitable for comprehensive genetic analysis of various disease traits (22, 30). To enhance interpretability and minimize redundancy, we selected representative phenotypes in this study. Furthermore, we excluded sex-specific disease traits and those with fewer than 500 cases due to data availability and statistical power concerns. Consequently, a total of 678 non-delirium traits were included in the Phe-MR analysis to further explore potential on-target side effects of metabolites related to delirium (**Figure 1**; **Supplementary Table 4**). In the Phe-MR analysis, we utilized the IVW method as the primary analysis approach (26). Additionally, we employed MR-Egger analysis to assess pleiotropy (29). The Phe-MR findings were standardized to reflect a change in metabolite levels corresponding to a 10% reduction in the risk of delirium. This standardization allowed us to uncover the side effects of interventions targeting metabolites for delirium and to directly compare the magnitude and direction of these side effects.

All results are presented as ORs with their 95% CIs of outcomes. In stage 1, an observed 2-sided *p* < 3.88 x 10^-4^ (Bonferroni-corrected significance threshold calculated as 0.05 divided by 129 [for 129 blood metabolites]) was considered statistically significant evidence for a causal association. In stage 2, the statistical significance for Phe-MR analysis was set at *p* = 2.46 x 10^-5^, which was corrected for multiple comparisons using the Bonferroni method (0.05/2034[3 identified delirium metabolites in stage 1 x 678 diseases]).

Most statistical analyses were performed in R software (version 4.3.1; R Development Core Team) with the packages CMplot (version 4.4.1), TwoSampleMR (version 0.5.7), ggplot2(version 3.4.3), ggrepel (version 0.9.3). Some data analysis was performed through Microsoft Office software.

## 3 Results

### 3.1 Identification of causal mediators for delirium from the blood metabolome

**Figure 2:**
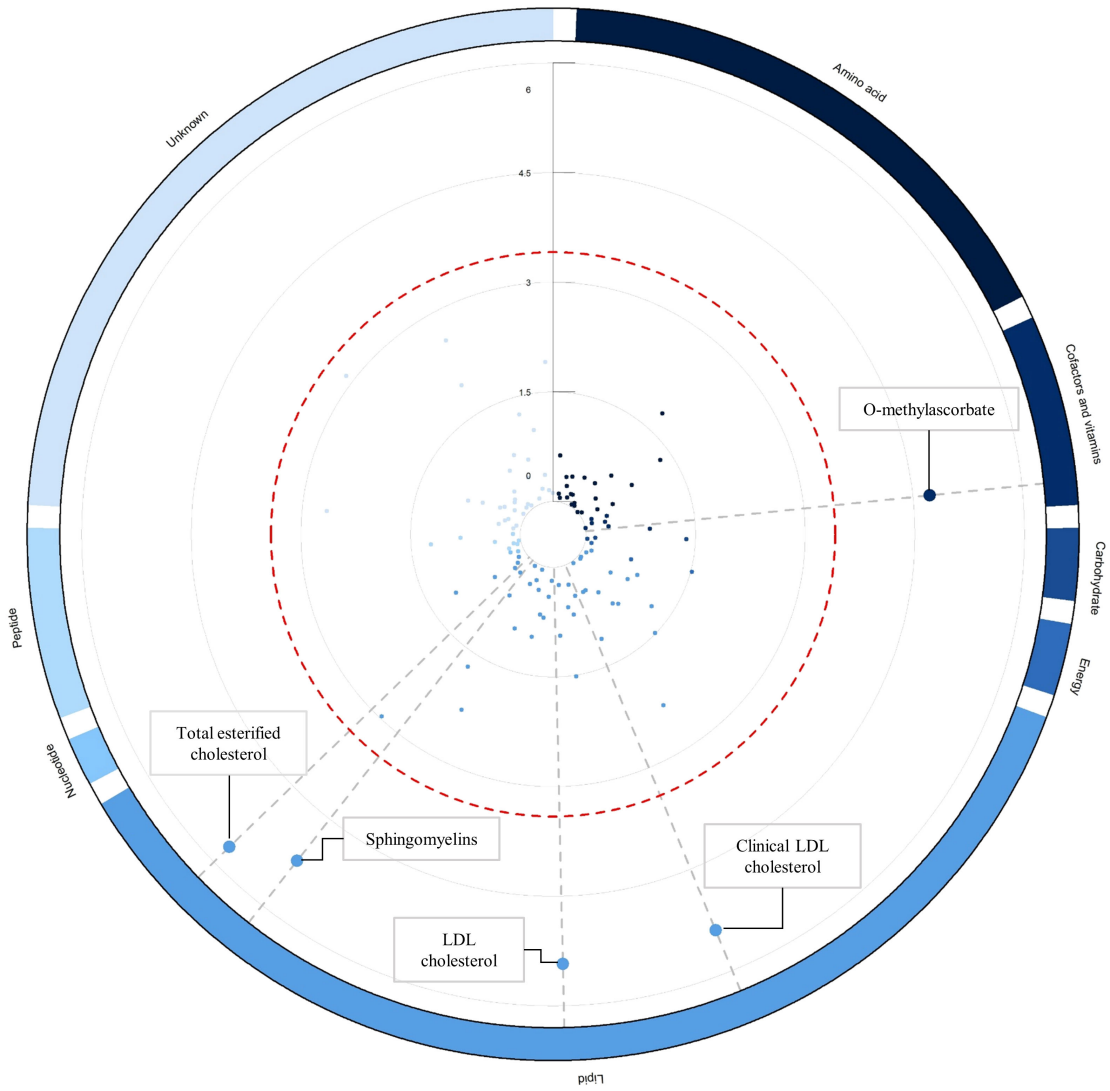
Circular Manhattan plot illustrating associations between blood metabolites and delirium risk. The circular Manhattan plot illustrates the relationships between blood metabolites and the vulnerability to delirium through the lens of inverse-variance weighted MR analysis. The red dashed line demarcates the threshold denoting Bonferroni-corrected significance (p < 0.05/129 = p < 3.88 x 10^-4^), and significant metabolite data points are magnified for emphasis. Worth noting, the collection of 129 blood metabolites is systematically arranged into clusters and differentiated by color based on their corresponding super-pathways, as elucidated in **Supplementary Table 1**. Elaborated and comprehensive results regarding the associations between blood metabolites and delirium, derived from the inverse-variance weighted MR analysis, are comprehensively detailed in **Supplementary Table 5**.

As an initial step, we employed the IVW method within the primary MR analysis to examine the connections between the 129 blood metabolites and the propensity for delirium. Within the primary analysis, we observed significant correlations: genetically determined low O-methylascorbate, elevated levels of LDL-C, clinical LDL-C, total esterified cholesterol, and sphingomyelin were all markedly linked to an escalated vulnerability to delirium (**Figure 2**; **Supplementary Table 5**). Following this, we proceeded with a series of sensitivity analyses aimed at evaluating the robustness of the associations uncovered in the primary analysis (**Supplementary Tables 5, 6**; **Supplementary Figure 1**). As detailed in **Supplementary Table 6**, the MR-Egger regression method indicated the presence of directional pleiotropy in relation to the associations involving LDL-C and total esterified cholesterol with respect to delirium risk.

**Figure 3:**
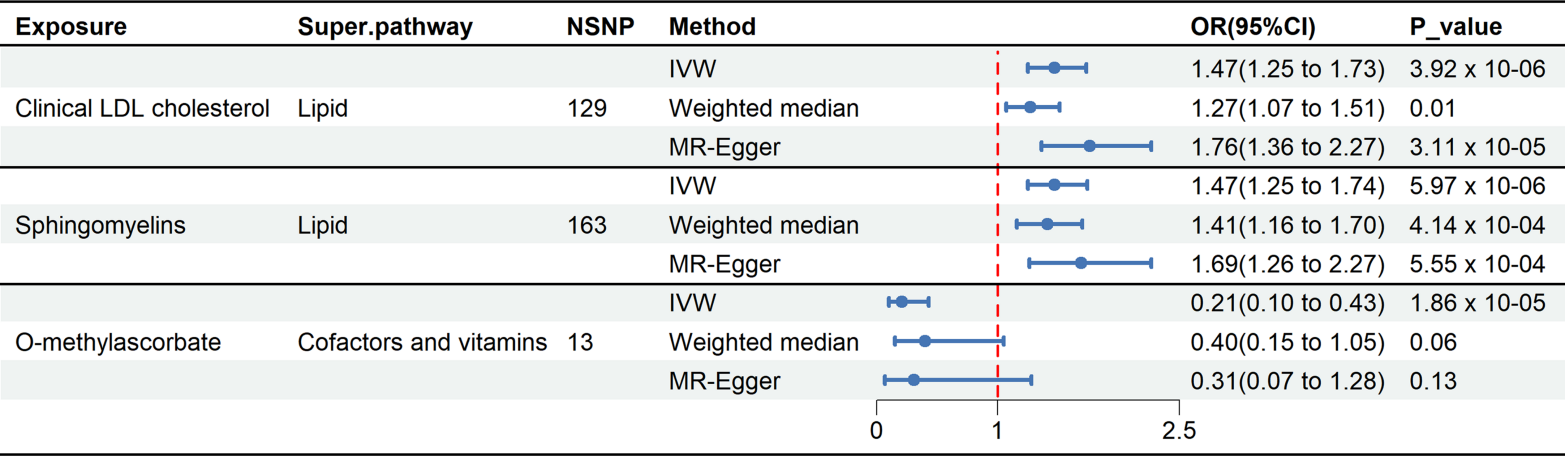
Forest plot illustrates the causal relationship between metabolites and delirium by using different methods. This forest plot displays the analytical results using different methods for clinical LDL-C, sphingomyelin, and O-methylascorbate. Among these three metabolites, the IVW method showed p-values below the threshold (3.88 x 10^-4^), and MR-Egger regression indicated the absence of directional pleiotropy.

In total, three causal mediators were identified concerning the risk of delirium, as detailed in **Figure 3**. In relation to these metabolites, a one-standard deviation (SD) increase in genetically determined O-methylascorbate (OR: 0.21, 95% CI: 0.10-0.43, *p* = 1.86 x 10^-5^) was linked to a decreased risk of delirium. Conversely, each one SD increment in genetically determined sphingomyelin (OR: 1.47, 95% CI: 1.25-1.74, *p* = 5.97 x 10^-6^) and clinical LDL-C (OR: 1.47, 95% CI: 1.25-1.73, *p* = 3.92 x 10^-6^) corresponded to an increased risk of delirium.

### 3.2 Phe-MR analysis for the associations between identified metabolites and 678 non-delirium diseases

**Figure 4:**
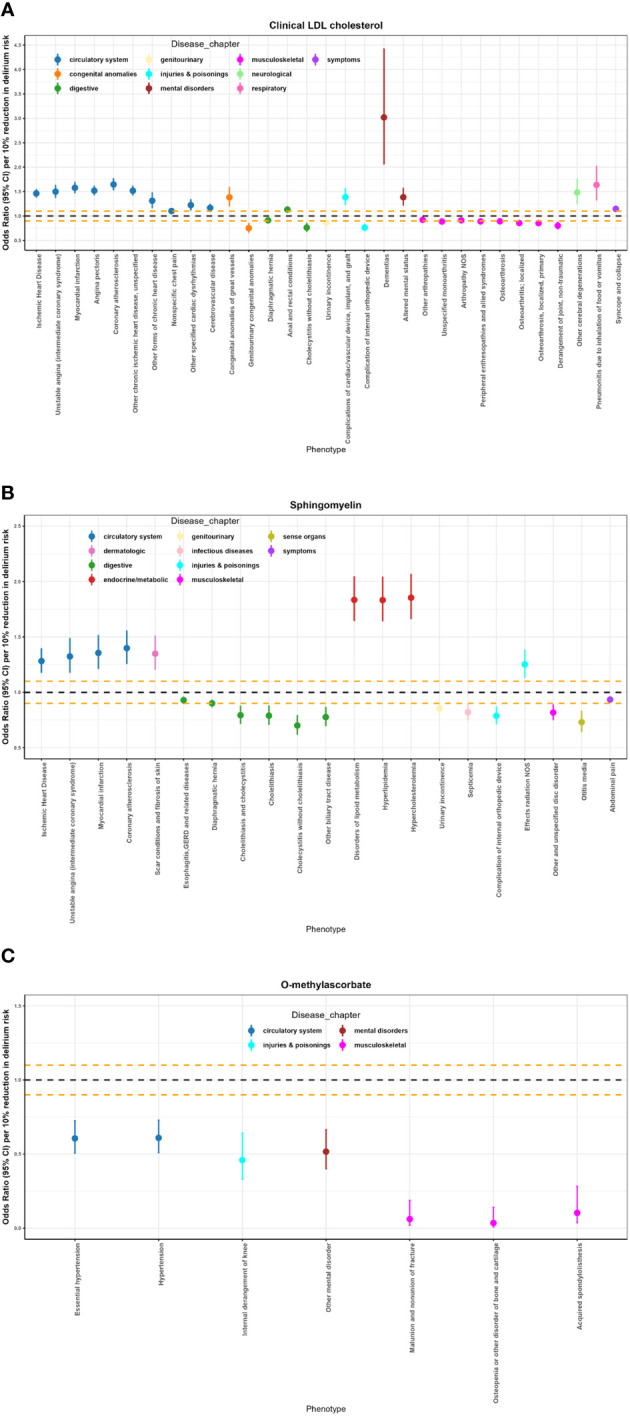
Potential on-target side effects of clinical LDL cholesterol, sphingomyelin, and O-methylascorbate interventions revealed by Phe-MR analysis. **(A)** Significant results of Phe-MR analysis for clinical LDL-C. **(B)** Significant results of Phe-MR analysis for sphingomyelin. **(C)** Significant results of Phe-MR analysis for O-methylascorbate. The OR estimates, along with their corresponding 95% CI, represent the susceptibility impact to various non-delirium conditions. Associations crossing the horizontal black dashed line indicate adverse effects, while those below the line signify favorable outcomes. Additionally, the horizontal orange dashed lines denote scales for OR of 0.90 and 1.10, respectively.

We expanded our investigation through Phe-MR to systematically evaluate the effects of the identified metabolites associated with delirium on the susceptibilities of 678 non-delirium diseases. This approach allowed us to explore the potential profiles of side effects. Differing from the previous MR analyses, the outcomes from Phe-MR were standardized based on a 10% reduction in the delirium risk achieved by targeting specific metabolites. Consequently, the associations derived can be interpreted as concurrent side effects expected to manifest when targeting O-methylascorbate, sphingomyelin, and clinical LDL-C for the mitigation or treatment of delirium. In the context of the Phe-MR analysis employing the IVW method, a cumulative total of 70 associations attained a significance threshold that underwent Bonferroni correction, with a p-value set at *p* = 2.46 x 10^-5^ (calculated as 0.05 divided by 2034, the result of 3 metabolites multiplied by 678 diseases). These significant associations are illustrated in **Figure 4** and expounded upon in **Supplementary Tables 7–9**, as well as in **Supplementary Figures 1–3**. Furthermore, we identified 11 significant associations in the Phe-MR analysis that exhibited directional pleiotropy, as indicated by the MR-Egger regression results (details can be found in **Supplementary Table 10**). As a result, these 11 associations were excluded, leaving a final selection of 59 significant associations for subsequent comprehensive investigation, as elucidated in **Supplementary Table 11**.

A total of fifty-nine significant associations were identified upon the targeting of clinical LDL-C, sphingomyelin, and O-methylascorbate in the context of various non-delirium diseases (**Figure 4**; **Supplementary Table 12**). In a concise overview, clinical LDL-C exerts a detrimental effect on 15 conditions associated with the circulatory system, mental disorders, neurological conditions, respiratory issues, and symptoms. Conversely, it contributes positively to 9 conditions encompassing the musculoskeletal and genitourinary realms. Simultaneously, it has adverse impacts on 3 out of the remaining 7 diseases spanning 3 distinct disease chapters. Additionally, sphingomyelin has adverse effects on 8 conditions encompassing the circulatory, dermatologic, and endocrine/metabolic realms. In contrast, it yields favorable outcomes for 11 conditions related to the digestive, genitourinary, infectious diseases, musculoskeletal system, sense organs, and symptoms, while exerting a detrimental influence on one of the remaining two diseases within a specific disease chapter. Furthermore, O-methylascorbate exhibits protective effects across 7 conditions, encompassing circulatory systems, mental disorders, injuries & poisonings, and the musculoskeletal system.

Among the most prominent associations, ischemic heart disease exhibited a per 10% reduction in delirium risk OR of 1.20 (95% CI: 1.38–1.55; *p* = 7.82 x 10^-42^) in relation to clinical LDL-C. Hypercholesterolemia was notably linked to an OR of 1.85 (95% CI: 1.66–2.07; *p* = 1.42 x 10^-28^) per 10% reduction in delirium risk, associated with sphingomyelin. Moreover, essential hypertension demonstrated an OR of 0.61 (95% CI: 0.51–0.72; *p* = 3.87 x 10^-8^) per 10% reduction in delirium risk, correlated with O-methylascorbate.

## 4 Discussion

Through the integration of metabolomics and genomics, the present MR study has yielded novel insights into the quest for promising and safe drug targets for delirium. From an initial pool of 129 blood metabolites, we identified three metabolites—clinical LDL-C, sphingomyelin, and O-methylascorbate—with potential causal links to delirium. Among these three, O-methylascorbate demonstrated protective effects, whereas clinical LDL-C and sphingomyelin exhibited deleterious impacts on delirium. Furthermore, we conducted a Phe-MR analysis to forecast on-target side effects associated with prospective delirium treatments involving interventions targeting these metabolites. Within the Phe-MR analysis, clinical LDL-C displayed adverse consequences across 18 diseases (with 58% of these cases being detrimental), whereas sphingomyelin correlated with a reduced risk in 21 diseases (with 52% being favorable associations). Additionally, it is noteworthy that O-methylascorbate manifested beneficial effects on the risk of seven additional diseases apart from delirium.

Clinical LDL-C, as a commonly measured clinical indicator, is widely recognized for its close association with cardiovascular disease (31). Recently, mounting evidence suggests a significant correlation between LDL-C and cognitive decline (32, 33), potentially linked to its role in promoting vascular pathology that affects cerebral blood flow. However, some studies have indicated that LDL-C levels may not be related to cognitive impairment (34, 35). Presently, an observational cross-sectional study on LDL-C and delirium post-surgery suggests that LDL-C is a risk factor for postoperative delirium (36). Nevertheless, the study is limited by various confounding factors, making it inconclusive regarding the relationship between LDL-C and delirium. Currently, there is a lack of large-scale, multicenter studies to substantiate the association between LDL-C and delirium. In this context, our MR study delved into the causal relationship between LDL-C and delirium at the genetic level, indicating that elevated LDL-C levels increase the risk of delirium. Furthermore, in Phe-MR analysis, LDL-C was found to have adverse effects on ten circulatory system diseases, two mental disorders, and one neurological disorder, while benefiting musculoskeletal diseases. Interestingly, our research findings reveal that elevated LDL-C levels also have adverse effects on dementias, altered mental status, and other cerebral degenerations. It is possible that LDL-C plays an intermediary role in the pathological progression of these three diseases. Therefore, the possibility of treating delirium by lowering LDL-C levels may be a viable approach, although the benefits and risks should be carefully weighed.

Sphingomyelin, essential components of neuronal membrane structures, serves both as structural constituents and signal carriers in physiological processes, and their role in cognitive functions has been extensively researched (37–39). Delirium is an independent risk factor associated with increased hospitalization costs and mortality rates and is closely linked to cognitive impairment (40, 41). However, the research findings regarding the relationship between sphingomyelin and cognitive function are inconsistent. Some studies suggest that elevated sphingomyelin levels are associated with a decline in cognitive function (39, 42), while others indicate that reduced sphingomyelin levels lead to cognitive decline (38, 43). Currently, there is a lack of large-scale randomized controlled trials (RCTs) to investigate the causal relationship between sphingomyelin and delirium. In this context, it is challenging to determine whether elevated sphingomyelin is a risk or protective factor for delirium. Given the current dilemma, our MR study employs genetically determined variations allocated at conception as instrumental variants to explore the causal relationship between sphingomyelin and the risk of delirium. Based on a cohort of 359,699 European individuals, our current MR study reveals a positive correlation between genetically determined sphingomyelin levels and the risk of delirium. The potential mechanism underlying the influence of phospholipids on delirium may be attributed to the overall impact of phospholipid metabolism on pro-inflammatory processes, such as the nuclear factor κB signaling pathway (44), as well as the downregulation of phospholipid synthases, which can alleviate inflammation in murine microglial cells (45). Considering that Phe-MR analysis has identified some adverse effects associated with sphingomyelin, mostly related to cardiovascular and endocrine/metabolic diseases, caution is warranted when considering sphingomyelin-based therapeutic strategies for the prevention and treatment of delirium.

O-methylascorbate, a naturally occurring metabolite of ascorbic acid, exhibits relatively low cytotoxicity while possessing potent antioxidant stress response capabilities (46, 47). Although the exact pathogenesis of delirium remains uncertain (48), there is a consensus that oxidative stress may be a significant risk factor for delirium (49, 50). A prospective study on elderly cardiovascular surgery patients has suggested that a postoperative decrease in plasma ascorbic acid levels may be associated with the occurrence of delirium (51). However, this study had limitations in terms of sample size, the specific nature of the surgeries, and its observational design, which cannot establish a clear causal relationship between ascorbic acid and delirium. In our MR study, we integrated metabolomics and genetic data to investigate the causal relationship between O-methylascorbate and delirium. Our findings indicate a negative correlation between genetically determined O-methylascorbate levels and the risk of delirium. Further Phe-MR analysis revealed that O-methylascorbate not only lacked the anticipated adverse effects but also had a protective effect against seven diseases. Therefore, O-methylascorbate holds promise as a potential therapeutic target for treating delirium.

Our findings bear significant implications for public health and clinical practice. Delirium, characterized by a substantial increase in patient mortality (3), frequently portends an unfavorable prognosis (4), and it may even result in persistent cognitive decline and dementia (2), yet it currently lacks effective pharmacological treatment options (5). Thus, identifying key biomarkers to identify high-risk individuals for cognitive impairment and enhancing early prevention through active monitoring and intervention are crucial. Our research results suggest that certain blood metabolites, including clinical LDL-C, sphingomyelin, and O-methylascorbate, may serve as potential predictive biomarkers for delirium. Currently, only dexmedetomidine is considered effective in preventing delirium (52), as other medications are not recommended for delirium prevention (53, 54). In this context, our MR study, based on metabolomics and genomics, offers new insights into promising targets for delirium prevention and treatment. Based on our research findings, these three identified blood metabolites, especially O-methylascorbate without the anticipated adverse effects, may represent potential targets for the development of delirium treatment drugs. Further clinical trials are needed to confirm their effectiveness and safety.

This study presents several notable advantages. Firstly, to our knowledge, it constitutes the inaugural comprehensive MR investigation integrating genomics and metabolomics data, aiming to unearth novel causal mediators of delirium. Secondly, our research leveraged meticulously designed GWASs featuring substantial sample sizes, facilitating the formulation of robust causal inferences with substantial statistical power. Lastly, we extended our analysis with Phe-MR to meticulously vet promising and secure candidate drug targets for the development of delirium therapeutics and subsequent clinical trials, while forecasting potential on-target side effects.

Nevertheless, it is imperative to acknowledge several limitations inherent in this study. Firstly, despite the inclusion of 129 distinct metabolites within our MR investigation derived from three extensive GWASs via stringent selection criteria, it is imperative to recognize that these metabolites constitute merely a modest fraction of the entire blood metabolome. Consequently, our study framework lends itself to expansion in future investigations encompassing a broader spectrum of metabolites. Secondly, our participant cohort exclusively consisted of individuals of European ancestry. While this choice mitigated the potential for spurious associations stemming from population stratification biases, it concurrently imposes constraints on the generalizability of our findings to non-European populations. Therefore, future research endeavors employing multiethnic cohorts are warranted to substantiate our results. Lastly, our reliance on the PheCode system hinged upon hospital diagnoses, wherein maladies characterized by infrequent hospital admissions might be inadequately represented.

## 5 Conclusions

This systematic MR investigation revealed that genetically predicted elevated O-methylascorbate levels may be linked to a reduced risk of incident delirium. Conversely, clinical LDL-C and sphingomyelin were associated with an increased risk of delirium. The study also meticulously characterized side-effect profiles, contributing valuable insights to the prioritization of drug targets. Notably, O-methylascorbate emerges as a promising candidate for the prevention and treatment of delirium, with no anticipated detrimental side effects.

## Data availability statement

The original contributions presented in the study are included in the article/**Supplementary Material**. Further inquiries can be directed to the corresponding authors.

## Author contributions

CBL: Conceptualization, Investigation, Methodology, Visualization, Writing – original draft. DL: Formal analysis, Software, Visualization, Writing – original draft. LLZ: Investigation, Methodology, Software, Writing – original draft. YL: Formal analysis, Software, Writing – original draft. QY: Investigation, Methodology, Software, Writing – original draft. GYZ: Investigation, Methodology, Writing – original draft. LSL: Investigation, Methodology, Writing – original draft. HLL: Investigation, Methodology, Writing – original draft. JY: Investigation, Methodology, Supervision, Writing – original draft. XFW: Software, Visualization, Writing – review & editing. FZH: Funding acquisition, Project administration, Resources, Supervision, Writing – review & editing.
