# Supplementary material for: Association between human blood metabolome and the risk of delirium: a Mendelian Randomization study
Source: Front Endocrinol (Lausanne). 2024 Jan 11;14:1332712. doi: 10.3389/fendo.2023.1332712 (PMC10808797; doi:10.3389/fendo.2023.1332712)
Supplement: Supplementary file 1 [file DataSheet_1.docx]

**Association between human blood metabolome and delirium risk: A Systematic Mendelian Randomization Study**

**Supplementary Material**

**Summary of supplementary tables and figures**

**Table S1** Overview of the blood metabolites included in the MR study.

**Table S2** Detailed description of the genetic instruments for blood metabolites used in stage one of the MR study.

**Table S3** Detailed description of the genetic instruments for blood metabolites used in stage two of the MR study.

**Table S4** 678 diseases incorporated in the Phe-MR analysis.

**Table S5** Inverse-variance weighted MR analysis for the associations between blood metabolites and delirium.

**Table S6** Sensitivity analyses for significant blood metabolites.

**Table S7** Phe-MR analyses for the associations between clinical LDL cholesterol and 678 diseases using the inverse-variance weighted method.

**Table S8** Phe-MR analyses for the associations between sphingomyelin and 678 diseases using the inverse-variance weighted method.

**Table S9** Phe-MR analyses for the associations between O-methylascorbate and 678 diseases using the inverse-variance weighted method.

**Table S10** The MR-Egger regression for directional pleiotropy of clinical LDL cholesterol, sphingomyelin, and O-methylascorbate with the risk of multiple non-delirium’s diseases in Phe-MR analyses.

**Table S11** Phe-MR analyses for causal associations of clinical LDL cholesterol, sphingomyelin, and O-methylascorbate with the risk of multiple non-delirium’s diseases.

**Table S12** Summary of significant Phe-MR findings representing side effects associated with targeting identified metabolites.

**Figure S1.** Leave-one-out plots of MR analysis results for three significant metabolites: clinical LDL-C, sphingomyelin, and O-methylascorbate.

**Figure S2** The Manhattan plot displaying on-target side effects of clinical LDL cholesterol in the inverse-variance weighted Phe-MR analysis.

**Figure S3** The Manhattan plot displaying on-target side effects of sphingomyelin in the inverse-variance weighted Phe-MR analysis.

**Figure S4** The Manhattan plot displaying on-target side effects of O-methylascorbate in the inverse-variance weighted Phe-MR analysis.

**Table S1. Overview of the blood metabolites included in the MR study.**

| **Metabolites** | **Super-pathway** | **Used SNPs** | **Sample size** | **F value^a^** |
| --- | --- | --- | --- | --- |
| 2-hydroxyisobutyrate | Amino acid | 4 | 6,539 | 50.99 |
| 3-methyl-2-oxovalerate | Amino acid | 3 | 7,779 | 42.01 |
| 4-acetamidobutanoate | Amino acid | 6 | 6,930 | 72.54 |
| alpha-hydroxyisovalerate | Amino acid | 3 | 7,668 | 63.26 |
| asparagine | Amino acid | 3 | 7,761 | 77.82 |
| betaine | Amino acid | 5 | 7,806 | 59.41 |
| citrulline | Amino acid | 3 | 7,773 | 31.75 |
| glutaroyl carnitine | Amino acid | 9 | 7,701 | 77.33 |
| glycine | Amino acid | 5 | 7,802 | 170.47 |
| isobutyrylcarnitine | Amino acid | 6 | 7,812 | 69.78 |
| isovalerylcarnitine | Amino acid | 7 | 7,789 | 54.26 |
| kynurenine | Amino acid | 5 | 7,816 | 69.74 |
| leucine | Amino acid | 11 | 7,799 | 36.12 |
| N-acetylglycine | Amino acid | 6 | 7,135 | 69.46 |
| N-acetylornithine | Amino acid | 9 | 7,574 | 122.81 |
| proline | Amino acid | 4 | 7,816 | 86.38 |
| pyroglutamine | Amino acid | 4 | 7,800 | 71.97 |
| serine | Amino acid | 3 | 7,796 | 83.51 |
| tryptophan | Amino acid | 17 | 7,804 | 33.93 |
| tryptophan betaine | Amino acid | 3 | 7,439 | 53.36 |
| tyrosine | Amino acid | 4 | 7,807 | 38.72 |
| X-03056--N-[3-(2-Oxopyrrolidin-1-yl)propyl]acetamide | Amino acid | 5 | 7,812 | 39.17 |
| X-12510--2-aminooctanoic acid | Amino acid | 6 | 7,566 | 96.90 |
| 1,5-anhydroglucitol (1,5-AG) | Carbohydrate | 6 | 7,746 | 62.67 |
| erythronate | Carbohydrate | 3 | 7,752 | 37.93 |
| mannose | Carbohydrate | 5 | 7,793 | 76.56 |
| Acetate | Cofactors and vitamins | 18 | 115,046 | 45.14 |
| Acetoacetate | Cofactors and vitamins | 6 | 115,075 | 48.82 |
| bilirubin (E,E) | Cofactors and vitamins | 6 | 7,748 | 71.21 |
| bilirubin (E,Z or Z,E) | Cofactors and vitamins | 4 | 5,295 | 57.66 |
| bilirubin (Z,Z) | Cofactors and vitamins | 8 | 6,812 | 92.52 |
| biliverdin | Cofactors and vitamins | 8 | 6,686 | 94.39 |
| X-11593--O-methylascorbate | Cofactors and vitamins | 13 | 7,788 | 59.47 |
| X-11793--oxidized bilirubin | Cofactors and vitamins | 10 | 7,611 | 51.12 |
| Acetone | Energy | 15 | 115,075 | 50.08 |
| citrate | Energy | 7 | 7,813 | 37.95 |
| succinylcarnitine | Energy | 10 | 6,948 | 54.80 |
| 10-undecenoate (11:1n1) | Lipid | 3 | 7,806 | 104.22 |
| 18:2, linoleic acid (LA) | Lipid | 19 | 13,527 | 46.75 |
| 1-arachidonoylglycerophosphocholine | Lipid | 5 | 7,507 | 74.19 |
| 1-arachidonoylglycerophosphoethanolamine | Lipid | 3 | 7,798 | 49.48 |
| 1-arachidonoylglycerophosphoinositol | Lipid | 3 | 7,797 | 85.03 |
| 22:6, docosahexaenoic acid | Lipid | 5 | 13,499 | 43.21 |
| 3-dehydrocarnitine | Lipid | 5 | 7,809 | 52.66 |
| 4-androsten-3beta,17beta-diol disulfate 1 | Lipid | 6 | 7,804 | 83.66 |
| 5alpha-androstan-3beta,17beta-diol disulfate | Lipid | 6 | 7,345 | 79.14 |
| androsterone sulfate | Lipid | 8 | 7,785 | 182.04 |
| Apolipoprotein A1 | Lipid | 201 | 115,078 | 63.16 |
| Apolipoprotein B | Lipid | 139 | 115,078 | 92.33 |
| arachidonate (20:4n6) | Lipid | 3 | 7,816 | 53.78 |
| butyrylcarnitine | Lipid | 17 | 7,796 | 105.15 |
| carnitine | Lipid | 20 | 7,797 | 60.85 |
| cis-4-decenoyl carnitine | Lipid | 5 | 7,660 | 73.76 |
| Clinical LDL cholesterol | Lipid | 129 | 115,078 | 106.65 |
| decanoylcarnitine | Lipid | 5 | 7,766 | 68.05 |
| dihomo-linolenate (20:3n3 or n6) | Lipid | 3 | 7,805 | 55.48 |
| Docosahexaenoic acid | Lipid | 138 | 114,999 | 78.00 |
| clinical low density lipoprotein cholesterol | Lipid | 7 | 7,769 | 84.38 |
| Free cholesterol | Lipid | 34 | 13,497 | 57.30 |
| HDL cholesterol | Lipid | 228 | 115,078 | 67.94 |
| hexadecanedioate | Lipid | 3 | 6,887 | 124.97 |
| hexanoylcarnitine | Lipid | 7 | 7,786 | 86.12 |
| LDL cholesterol | Lipid | 130 | 115,078 | 97.51 |
| Mono-unsaturated fatty acids | Lipid | 180 | 114,999 | 59.10 |
| octadecanedioate | Lipid | 3 | 7,300 | 49.40 |
| octanoylcarnitine | Lipid | 5 | 7,790 | 101.93 |
| Omega-3 fatty acids | Lipid | 201 | 114,999 | 77.66 |
| Omega-6 fatty acids | Lipid | 146 | 114,999 | 60.50 |
| Omega-7, omega-9 and saturated fatty acids | Lipid | 6 | 13,506 | 51.40 |
| Other polyunsaturated fatty acids than 18:2 | Lipid | 42 | 13,549 | 59.50 |
| Phosphatidylcholine and other cholines | Lipid | 11 | 13,542 | 45.66 |
| Polyunsaturated fatty acids | Lipid | 183 | 114,999 | 59.40 |
| propionylcarnitine | Lipid | 4 | 7,813 | 72.78 |
| Remnant cholesterol (non-HDL, non-LDL -cholesterol) | Lipid | 138 | 115,078 | 81.93 |
| Saturated fatty acids | Lipid | 134 | 114,999 | 56.08 |
| Serum total cholesterol | Lipid | 51 | 21,491 | 56.40 |
| Serum total triglycerides | Lipid | 16 | 21,545 | 50.90 |
| Sphingomyelins | Lipid | 163 | 114,999 | 75.82 |
| tetradecanedioate | Lipid | 3 | 6,046 | 127.49 |
| Total cholesterol minus HDL-C | Lipid | 135 | 115,078 | 91.47 |
| Total esterified cholesterol | Lipid | 142 | 115,078 | 78.93 |
| Total fatty acids | Lipid | 11 | 13,505 | 47.74 |
| Total phosphoglycerides | Lipid | 11 | 13,519 | 48.69 |
| VLDL cholesterol | Lipid | 158 | 115,078 | 64.99 |
| X-11445--5-alpha-pregnan-3beta,20alpha-disulfate | Lipid | 3 | 2,570 | 38.61 |
| X-13431--nonanoylcarnitine | Lipid | 7 | 6,591 | 84.13 |
| urate | Nucleotide | 3 | 7,819 | 60.53 |
| uridine | Nucleotide | 3 | 7,800 | 41.50 |
| Albumin | Peptide | 38 | 115,060 | 79.61 |
| bradykinin, des-arg(9) | Peptide | 5 | 4,570 | 73.02 |
| gamma-glutamylglutamine | Peptide | 3 | 7,662 | 45.43 |
| gamma-glutamyltyrosine | Peptide | 3 | 7,468 | 31.61 |
| Glycoprotein acetyls | Peptide | 131 | 115,078 | 64.13 |
| Glycoproteins | Peptide | 72 | 18,734 | 65.45 |
| HWESASXX | Peptide | 3 | 7,700 | 39.27 |
| X-12244--N-acetylcarnosine | Peptide | 6 | 6,608 | 53.69 |
| X-02269 | Unknown | 3 | 7,701 | 45.72 |
| X-03094 | Unknown | 3 | 7,804 | 52.99 |
| X-08402 | Unknown | 6 | 7,726 | 72.07 |
| X-08988 | Unknown | 3 | 7,776 | 63.57 |
| X-10510 | Unknown | 3 | 7,792 | 67.68 |
| X-11204 | Unknown | 3 | 7,799 | 33.06 |
| X-11261 | Unknown | 5 | 7,771 | 41.14 |
| X-11315 | Unknown | 3 | 7,785 | 46.76 |
| X-11440 | Unknown | 5 | 7,686 | 117.41 |
| X-11441 | Unknown | 4 | 7,072 | 128.27 |
| X-11442 | Unknown | 6 | 7,142 | 89.35 |
| X-11444 | Unknown | 3 | 7,758 | 51.00 |
| X-11469 | Unknown | 4 | 7,779 | 44.28 |
| X-11491 | Unknown | 4 | 6,584 | 55.63 |
| X-11529 | Unknown | 11 | 6,664 | 91.75 |
| X-11530 | Unknown | 8 | 7,409 | 81.63 |
| X-11538 | Unknown | 7 | 7,804 | 109.81 |
| X-11787 | Unknown | 7 | 7,811 | 71.28 |
| X-11792 | Unknown | 3 | 2,442 | 45.50 |
| X-11905 | Unknown | 3 | 4,761 | 106.10 |
| X-12063 | Unknown | 14 | 7,197 | 91.52 |
| X-12092 | Unknown | 16 | 7,500 | 111.39 |
| X-12093 | Unknown | 3 | 2,854 | 76.24 |
| X-12456 | Unknown | 3 | 4,774 | 97.57 |
| X-12556 | Unknown | 4 | 7,483 | 52.68 |
| X-12644 | Unknown | 3 | 7,795 | 35.51 |
| X-12696 | Unknown | 5 | 7,409 | 66.29 |
| X-12728 | Unknown | 8 | 537 | 32.75 |
| X-12798 | Unknown | 12 | 7,552 | 114.03 |
| X-12844 | Unknown | 4 | 7,768 | 41.80 |
| X-13429 | Unknown | 4 | 6,344 | 118.30 |
| X-13435 | Unknown | 3 | 6,970 | 73.83 |
| X-14626 | Unknown | 3 | 6,904 | 97.72 |

Abbreviations: MR, Mendelian randomization; SNP, single nucleotide polymorphism.

^a^ The strength of genetic instruments for each blood metabolite in the present MR study.

**Table S2. Detailed description of the genetic instruments for blood metabolites used in stage one of the MR study.**

| **Metabolites** | **SNPs used in stage one** |
| --- | --- |
| 2-hydroxyisobutyrate | rs11160112, rs11635023, rs1795963, rs493519 |
| 3-methyl-2-oxovalerate | rs11084396, rs1260326, rs1440581 |
| 4-acetamidobutanoate | rs1041983, rs13271577, rs2029830, rs721399, rs7228099, rs9657488 |
| alpha-hydroxyisovalerate | rs12141041, rs2403254, rs893971 |
| asparagine | rs2011069, rs3017098, rs8008020 |
| betaine | rs16876394, rs185077, rs2291919, rs495360, rs715 |
| citrulline | rs10164524, rs1509820, rs682103 |
| glutaroyl carnitine | rs12927959, rs13375749, rs17641971, rs1981524, rs2291449, rs715, rs8012, rs8056893, rs896388 |
| glycine | rs12328639, rs13021675, rs13401425, rs2719966, rs715 |
| isobutyrylcarnitine | rs12202350, rs2404602, rs3798167, rs648253, rs9456496, rs9457843 |
| isovalerylcarnitine | rs11950562, rs17672041, rs2291449, rs2662314, rs4706020, rs6596022, rs9635324 |
| kynurenine | rs10085935, rs17630235, rs3184504, rs750950, rs8051149 |
| leucine | rs10923016, rs1440581, rs1871053, rs2713737, rs6735596, rs6737109, rs6820011, rs6820313, rs7584842, rs7647029, rs9541214 |
| N-acetylglycine | rs12328639, rs12468557, rs13021675, rs1367053, rs715, rs7948073 |
| N-acetylornithine | rs1036113, rs11679923, rs13415017, rs1653259, rs17348756, rs17349853, rs1881244, rs2043099, rs7573275 |
| proline | rs2518802, rs715544, rs9605907, rs9680846 |
| pyroglutamine | rs11613331, rs17279437, rs2159892, rs715 |
| serine | rs1163251, rs4947534, rs715 |
| tryptophan | rs1016522, rs13122250, rs1373962, rs2111118, rs284191, rs38271, rs4306882, rs4615256, rs4958379, rs603446, rs6480970, rs6935961, rs710580, rs7463805, rs7584842, rs9511152, rs972459 |
| tryptophan betaine | rs2405522, rs2548993, rs7733814 |
| tyrosine | rs12728678, rs172650, rs174408, rs9400467 |
| X-03056--N-[3-(2-Oxopyrrolidin-1-yl)propyl]acetamide | rs1005390, rs10776672, rs11978239, rs3798167, rs6502844 |
| X-12510--2-aminooctanoic acid | rs1165209, rs1318987, rs13538, rs1466245, rs17348756, rs6710726 |
| 1,5-anhydroglucitol (1,5-AG) | rs12465802, rs3800993, rs4954573, rs6736412, rs7570971, rs9756306 |
| erythronate | rs10263766, rs2391217, rs4687717 |
| mannose | rs1260326, rs2141371, rs7349418, rs7583698, rs937813 |
| Acetate | rs11066283, rs12005199, rs1260326, rs138319634, rs139097404, rs142237051, rs145679432, rs185281343, rs2424699, rs3184504, rs4813543, rs4988235, rs6036912, rs6050281, rs6057240, rs6106989, rs6138465, rs78845105 |
| Acetoacetate | rs12721041, rs144305620, rs150515955, rs2255400, rs4240624, rs6889983 |
| bilirubin (E,E) | rs28900371, rs3796092, rs6723936, rs7608713, rs838718, rs887829 |
| bilirubin (E,Z or Z,E) | rs28898590, rs4281899, rs6723936, rs887829 |
| bilirubin (Z,Z) | rs12479240, rs28900385, rs3771342, rs3796092, rs6717546, rs6760588, rs838718, rs887829 |
| biliverdin | rs10170160, rs17864661, rs28900371, rs6717546, rs7608713, rs838705, rs887829, rs988344 |
| X-11593--O-methylascorbate | rs10412803, rs11089325, rs16982844, rs2686184, rs3804043, rs438798, rs4597638, rs4680, rs7290062, rs7707010, rs887200, rs9318225, rs9606212 |
| X-11793--oxidized bilirubin | rs10175949, rs1115381, rs11563251, rs11683356, rs12479240, rs28899194, rs3923630, rs6760588, rs838705, rs887829 |
| Acetone | rs11061153, rs11216157, rs117643180, rs117651719, rs117810762, rs146459385, rs1536826, rs2238691, rs2246634, rs2582783, rs318020, rs35953019, rs6889983, rs72864795, rs9590637 |
| citrate | rs1509123, rs170149, rs6507399, rs807670, rs835154, rs868395, rs906749 |
| succinylcarnitine | rs10988217, rs11637751, rs12899230, rs1472631, rs17806888, rs2686513, rs2729786, rs6703518, rs8060756, rs901273 |
| 10-undecenoate (11:1n1) | rs2494248, rs6077678, rs9333029 |
| 18:2, linoleic acid (LA) | rs11216129, rs113105517, rs11580527, rs1260326, rs143341434, rs144064722, rs144723570, rs17414716, rs174418, rs1800588, rs1825955, rs190934192, rs429358, rs4296389, rs4609471, rs6662286, rs7412, rs79225634, rs99780 |
| 1-arachidonoylglycerophosphocholine | rs1692120, rs174535, rs3738544, rs7104849, rs748196 |
| 1-arachidonoylglycerophosphoethanolamine | rs10468017, rs11045906, rs4149056 |
| 1-arachidonoylglycerophosphoinositol | rs11045834, rs1871395, rs8736 |
| 22:6, docosahexaenoic acid | rs11604424, rs143988316, rs145717049, rs174546, rs2281591 |
| 3-dehydrocarnitine | rs10070034, rs17171585, rs273913, rs316019, rs7737937 |
| 4-androsten-3beta,17beta-diol disulfate 1 | rs11669552, rs11761528, rs296396, rs4149452, rs4802397, rs7259671 |
| 5alpha-androstan-3beta,17beta-diol disulfate | rs10267212, rs10278040, rs13222543, rs2547231, rs4149056, rs4149452 |
| androsterone sulfate | rs10278040, rs13222543, rs182420, rs2141160, rs4236541, rs474229, rs6465737, rs7778571 |
| Apolipoprotein A1 | rs1002687, rs10091649, rs10184004, rs1047891, rs1077835, rs10810374, rs10838687, rs11039238, rs11040055, rs11071374, rs11076170, rs111929233, rs112001035, rs112495680, rs11249925, rs112688782, rs112853430, rs113149294, rs113194763, rs115047514, rs11643781, rs11662691, rs11671872, rs116843064, rs117154602, rs117628555, rs117687565, rs117749052, rs11789603, rs117901517, rs118078695, rs118136762, rs12021623, rs12230272, rs12287106, rs1233396, rs12447986, rs1260326, rs1263149, rs1264350, rs12708983, rs12720917, rs12962112, rs12963212, rs12969081, rs12975366, rs13107325, rs1320700, rs1358980, rs1367117, rs139957766, rs140947438, rs141368429, rs143376213, rs144305743, rs145205375, rs146390218, rs146548146, rs147275653, rs147464145, rs147556125, rs150911013, rs15285, rs1545614, rs1645779, rs1672867, rs16940302, rs1711041, rs172337, rs17240566, rs17269250, rs174574, rs17696736, rs1779823, rs1790781, rs1800961, rs1865834, rs1875236, rs187929675, rs188501176, rs190927521, rs1968493, rs2023473, rs2066716, rs2115429, rs2119693, rs2176040, rs2236252, rs2245793, rs2276329, rs2292318, rs2298428, rs235314, rs247615, rs2494748, rs2569550, rs261290, rs2642438, rs267738, rs2740488, rs2792735, rs283, rs28382814, rs28690720, rs289703, rs289719, rs289727, rs289752, rs291040, rs2925979, rs308, rs3130893, rs3289, rs34003087, rs34663616, rs34707604, rs35138338, rs35311766, rs35364714, rs35511894, rs357905, rs36018387, rs3768321, rs3794649, rs3859113, rs3892214, rs3898938, rs402465, rs41272663, rs4149307, rs4240624, rs4263041, rs4285809, rs429358, rs445093, rs4752890, rs4784717, rs4803760, rs4846921, rs4969141, rs501942, rs5167, rs56129100, rs56208677, rs583104, rs58680978, rs5883, rs6012281, rs60219248, rs60455398, rs6125085, rs61854123, rs61941676, rs62001835, rs62117161, rs62119267, rs652455, rs6586891, rs6587973, rs6606717, rs676210, rs686030, rs6993714, rs7003526, rs7133378, rs71352241, rs7139079, rs7198642, rs7200805, rs7229377, rs72786786, rs72836561, rs72913825, rs72925817, rs73624696, rs737338, rs74018729, rs75129833, rs75566930, rs75663614, rs75911530, rs76769796, rs76967117, rs77027049, rs77197725, rs77960347, rs78893833, rs78965095, rs7956099, rs8023503, rs899228, rs907866, rs921919, rs9268812, rs9304381, rs9471972, rs9491697, rs967645, rs9938413, rs9947678, rs9972789 |
| Apolipoprotein B | rs102275, rs1081106, rs11102964, rs111278137, rs112027066, rs11206513, rs11206517, rs112172017, rs11571787, rs11591147, rs11601507, rs11679386, rs11691986, rs118147862, rs12151108, rs12208357, rs12546944, rs1260326, rs12740374, rs12916, rs12983316, rs1337247, rs13702, rs140824606, rs141989097, rs143843429, rs144261139, rs144545816, rs145400326, rs146390218, rs146576912, rs148356565, rs1499279, rs1500188, rs150262789, rs151000110, rs151330717, rs17035665, rs17249001, rs1800777, rs181765708, rs1836278, rs190712692, rs199717562, rs203711, rs204480, rs204540, rs2107449, rs2326077, rs247616, rs2570344, rs261290, rs2618566, rs28399637, rs28597716, rs2965157, rs2980874, rs35057129, rs35836101, rs3794695, rs3798167, rs41279684, rs4263041, rs429358, rs4299376, rs4426495, rs45518133, rs4665698, rs4704262, rs4704834, rs472495, rs4803748, rs4803791, rs4804510, rs4876611, rs4927206, rs4927214, rs4970835, rs4971548, rs507766, rs533617, rs553427, rs55634260, rs55714927, rs56113850, rs562338, rs56325564, rs58542926, rs59379014, rs6016505, rs611060, rs62118464, rs648253, rs6511721, rs651649, rs6601299, rs6602909, rs6859, rs6920309, rs693, rs6938647, rs71364511, rs71435586, rs7203984, rs7246100, rs7246666, rs7249244, rs7251161, rs72655677, rs72660539, rs72694391, rs72703210, rs72902594, rs72929768, rs73013176, rs73048351, rs7343130, rs74607435, rs7523141, rs75237799, rs76186504, rs76670936, rs7700719, rs77278227, rs77303550, rs7737496, rs7750288, rs77542162, rs7773004, rs79220007, rs79429216, rs79915079, rs8042174, rs8107530, rs8113311, rs9295128, rs9302635, rs9682783, rs9973228 |
| arachidonate (20:4n6) | rs174602, rs17764324, rs412334 |
| butyrylcarnitine | rs11065176, rs11065202, rs11065208, rs11065390, rs11610379, rs1171617, rs1179368, rs1186055, rs12368199, rs12369156, rs208294, rs2708092, rs274567, rs278136, rs4767918, rs494632, rs7965649 |
| carnitine | rs11183620, rs11620955, rs11620973, rs1171618, rs12356193, rs12709393, rs1466788, rs2114713, rs2279014, rs2396004, rs2492788, rs3736438, rs419291, rs4860022, rs6479648, rs6862024, rs7098081, rs735315, rs7737937, rs9842133 |
| cis-4-decenoyl carnitine | rs11161521, rs12143716, rs17304141, rs721204, rs8396 |
| Clinical LDL cholesterol | rs102275, rs10901256, rs10953298, rs11102964, rs11127048, rs111278137, rs112027066, rs11206513, rs11206517, rs112172017, rs112481437, rs1135062, rs113588790, rs115740542, rs11591147, rs11621792, rs11691986, rs117261169, rs118147862, rs12151108, rs12208357, rs12740374, rs12981555, rs1337247, rs140824606, rs141645791, rs144261139, rs144545816, rs1461729, rs146576912, rs148356565, rs1499279, rs1500188, rs150262789, rs151000110, rs151330717, rs1661174, rs17035665, rs17249001, rs173539, rs174467, rs17696736, rs181765708, rs1836278, rs199717562, rs2015257, rs204480, rs204540, rs2065397, rs2272272, rs241440, rs2479413, rs2523764, rs2570344, rs2618566, rs2642438, rs2740488, rs28399637, rs2865505, rs2927439, rs2965157, rs2965167, rs34449399, rs35057129, rs35823804, rs35836101, rs3794695, rs3798167, rs3846662, rs405509, rs41279684, rs4263041, rs429358, rs4299376, rs4507059, rs4703665, rs4704262, rs4722551, rs4802216, rs4803791, rs4804510, rs4927206, rs4927214, rs4970835, rs533617, rs553427, rs55714927, rs562338, rs56325564, rs58542926, rs59379014, rs6016505, rs602662, rs60960031, rs611060, rs62115552, rs62116948, rs62118464, rs62120793, rs6511721, rs651649, rs6859, rs6882345, rs6920309, rs693, rs7012814, rs7246666, rs7251161, rs7254892, rs72660539, rs73013176, rs73036542, rs73048351, rs7343130, rs74607435, rs7523141, rs76186504, rs76670936, rs7700719, rs77278227, rs77303550, rs7750288, rs77542162, rs7773004, rs78912080, rs79915079, rs8113311, rs9749236, rs9973228 |
| decanoylcarnitine | rs11722868, rs17304141, rs2062541, rs7552404, rs8396 |
| dihomo-linolenate (20:3n3 or n6) | rs174455, rs6498540, rs968567 |
| Docosahexaenoic acid | rs1077835, rs11075278, rs11122450, rs11230741, rs11230749, rs11230759, rs112687416, rs114863007, rs115428654, rs11591147, rs11603763, rs116262432, rs11681659, rs116962591, rs117110139, rs117186302, rs117414940, rs117486534, rs117876551, rs117901517, rs117983270, rs118078695, rs12362237, rs1260326, rs12786457, rs12799017, rs12805902, rs13424225, rs138012803, rs138110361, rs139957766, rs139974673, rs141502961, rs142158911, rs14415, rs145786300, rs145908530, rs146651998, rs147981159, rs150156331, rs150684478, rs1675090, rs16940126, rs16940904, rs17200189, rs17269250, rs1729377, rs174467, rs174528, rs174569, rs174624, rs17821298, rs1791786, rs1791796, rs1794072, rs182611493, rs183130, rs183986993, rs188370817, rs191790331, rs198442, rs198476, rs2009875, rs2229738, rs2232143, rs2239370, rs2278426, rs2394976, rs2524292, rs259872, rs261291, rs2727260, rs273912, rs2853968, rs28690720, rs2924438, rs34663616, rs34707604, rs34780867, rs34793, rs35138338, rs35429473, rs35511894, rs35827276, rs36018387, rs36030967, rs4343027, rs445093, rs482548, rs5026246, rs525028, rs55901398, rs562338, rs58542926, rs59752567, rs61896137, rs61897792, rs62036306, rs62037519, rs62039124, rs638714, rs6507945, rs660240, rs673335, rs6931604, rs694954, rs695867, rs7115739, rs7116318, rs7119809, rs71374758, rs7229377, rs72655677, rs72836561, rs72920193, rs72924290, rs73487492, rs740006, rs7412, rs75354047, rs7570971, rs75938339, rs76380214, rs76978219, rs77027049, rs77053629, rs77407800, rs77960347, rs78353689, rs78893833, rs79126702, rs7924036, rs79787170, rs79969098, rs8042174, rs9304381, rs952275, rs9987289 |
| clinical low density lipoprotein cholesterol | rs10228787, rs11981478, rs13222543, rs2141160, rs474229, rs6465737, rs7778571 |
| Free cholesterol | rs1077835, rs10888908, rs113105517, rs113445611, rs113531395, rs115534052, rs11591147, rs11604424, rs116302332, rs12043403, rs13392272, rs142130958, rs144064722, rs146568567, rs174418, rs1825955, rs182695896, rs190934192, rs3005923, rs429358, rs4426495, rs4609471, rs4635554, rs56028521, rs565436, rs61770425, rs629301, rs6511721, rs72664202, rs726815, rs7412, rs75679663, rs77021821, rs79225634 |
| HDL cholesterol | rs10091649, rs10184004, rs10268632, rs10405357, rs10468017, rs1047891, rs10774625, rs10810374, rs11040322, rs11071374, rs11076170, rs111514504, rs112001035, rs113194763, rs114721139, rs114760566, rs115047514, rs11609805, rs11640954, rs116843064, rs11696696, rs117209788, rs117687565, rs117738782, rs117749052, rs117892711, rs11789603, rs117901517, rs118078695, rs118092024, rs118136762, rs11820504, rs11828763, rs1215112, rs12230272, rs12421131, rs12422125, rs12447986, rs12597428, rs1264372, rs12708983, rs12720917, rs12786130, rs12962112, rs12963212, rs12976739, rs13107325, rs1320700, rs13248499, rs138570705, rs139957766, rs141368429, rs143376213, rs145205375, rs145326144, rs1461728, rs146459385, rs147464145, rs148005124, rs148303195, rs150564454, rs150844304, rs150911013, rs1565097, rs1672867, rs16842, rs1716409, rs172337, rs17269250, rs17301781, rs1761457, rs17696736, rs17699030, rs1779823, rs17821274, rs1790781, rs1800777, rs1800961, rs180360, rs1875236, rs187929675, rs188501176, rs2011186, rs2066716, rs2070895, rs2115429, rs2119693, rs2176040, rs2245221, rs2276329, rs2280723, rs2292318, rs2298428, rs2307111, rs247615, rs2494748, rs2642438, rs267738, rs2740488, rs2777803, rs2792735, rs283, rs28382814, rs28623088, rs28690720, rs28742908, rs28818616, rs289703, rs289719, rs289727, rs289752, rs291040, rs2925979, rs3289, rs333947, rs34514836, rs34663616, rs34955499, rs35138338, rs35184771, rs35311766, rs35511894, rs35608584, rs35633876, rs357905, rs36018387, rs36226283, rs3768321, rs3794649, rs3811361, rs3859113, rs3892214, rs3898938, rs3899015, rs3926666, rs402465, rs41272663, rs41309280, rs4149307, rs4240624, rs4285809, rs435306, rs439401, rs445093, rs4752973, rs4784770, rs4846921, rs4969141, rs4985155, rs5030789, rs5167, rs56050009, rs56129100, rs56208677, rs570920454, rs58680978, rs5883, rs5896, rs59104589, rs59283948, rs59542880, rs6012281, rs60219248, rs6073958, rs6125085, rs61854123, rs61897792, rs61905084, rs61941676, rs62001835, rs62101705, rs652455, rs6586891, rs66514732, rs66806308, rs6734506, rs676210, rs688671, rs7010610, rs7012814, rs7017756, rs7133378, rs71336055, rs7134375, rs71352241, rs7197489, rs7198642, rs7200805, rs7203984, rs7229377, rs7241918, rs72784753, rs72786786, rs72823014, rs72836561, rs72959041, rs7308864, rs74018729, rs75566930, rs75609851, rs75663614, rs76083992, rs76769796, rs76860108, rs769449, rs77027049, rs77437185, rs77509279, rs77842142, rs77960347, rs78058190, rs7810507, rs7817574, rs7845090, rs78893833, rs79546662, rs799158, rs79968526, rs80041799, rs8023503, rs921919, rs9469899, rs9491697, rs9644636, rs9687846, rs9938413, rs9947678, rs998584 |
| hexadecanedioate | rs11045818, rs12228798, rs1871395 |
| hexanoylcarnitine | rs11161521, rs1171615, rs17304141, rs272869, rs5745515, rs721204, rs7534288 |
| LDL cholesterol | rs102275, rs10407439, rs10504255, rs10953298, rs11102964, rs111278137, rs112027066, rs11206517, rs112172017, rs112481437, rs1135062, rs114863007, rs11571787, rs11591147, rs1160983, rs11621792, rs11679386, rs11691986, rs117261169, rs118147862, rs12022410, rs12151108, rs12208357, rs1260326, rs12740374, rs12981555, rs13278097, rs1337247, rs140824606, rs144261139, rs144545816, rs1461729, rs146576912, rs148356565, rs1499279, rs1500188, rs150262789, rs150956718, rs151000110, rs151330717, rs1661174, rs17035665, rs17111503, rs17249001, rs174467, rs17696736, rs181765708, rs1836278, rs2015257, rs204480, rs204540, rs2065397, rs2570344, rs261290, rs2618566, rs2740488, rs28399637, rs2927439, rs2965157, rs2965167, rs34449399, rs35057129, rs35823804, rs35836101, rs36018387, rs3794695, rs3798167, rs3846662, rs405509, rs41279684, rs4149307, rs4263041, rs429358, rs4299376, rs4507059, rs472495, rs4803791, rs4804510, rs4927206, rs4927214, rs4970835, rs525028, rs533617, rs553427, rs55714927, rs562338, rs56325564, rs58542926, rs59379014, rs59950280, rs6016505, rs602662, rs6073958, rs60960031, rs611060, rs62115552, rs62116947, rs62118464, rs6511721, rs651649, rs6859, rs6882345, rs693, rs6938647, rs7246666, rs7251161, rs72655677, rs72660539, rs72703210, rs72843670, rs72902594, rs73013176, rs73048351, rs7343130, rs74607435, rs75237799, rs76186504, rs76670936, rs7700719, rs77278227, rs77303550, rs7750288, rs77542162, rs7773004, rs79220007, rs79429216, rs79915079, rs9276826, rs9749236, rs9973228 |
| Mono-unsaturated fatty acids | rs1002687, rs10160397, rs10160799, rs10189899, rs10207315, rs10214652, rs10455872, rs10458569, rs10892004, rs112259268, rs1128249, rs113271819, rs113296769, rs1145210, rs115047514, rs116316096, rs116843064, rs116886525, rs117619191, rs11775088, rs117788606, rs117794084, rs11828763, rs11940694, rs12055445, rs12270961, rs1240658, rs1260326, rs12720816, rs12721041, rs12916, rs12965067, rs13108218, rs13142655, rs13214992, rs13280055, rs1394092, rs139957766, rs139974673, rs141414463, rs141469619, rs142084074, rs142158911, rs142288236, rs142385484, rs143875230, rs1441770, rs148489550, rs148784079, rs1495741, rs149615216, rs1540037, rs16842, rs16891156, rs174564, rs182611493, rs186696265, rs186782888, rs187929675, rs1890896, rs193108398, rs1979023, rs1998045, rs2000999, rs2035816, rs2081687, rs2119693, rs2228607, rs2409836, rs2478236, rs2523675, rs261290, rs268, rs2736380, rs2740488, rs283, rs28562532, rs2943635, rs2980874, rs3094228, rs3130668, rs3134931, rs3198697, rs3289, rs34052301, rs34335422, rs35366682, rs35686293, rs3777411, rs3777420, rs386453, rs3898938, rs393155, rs3936511, rs402465, rs40270, rs405697, rs41265930, rs41267809, rs429358, rs4407894, rs4413654, rs4564803, rs4635554, rs4704834, rs4708870, rs4713427, rs4713555, rs4846914, rs4971548, rs523549, rs526748, rs534417, rs56322906, rs58542926, rs59774409, rs59859410, rs602633, rs61904855, rs62140449, rs62222988, rs622591, rs62496677, rs632057, rs6547820, rs6587973, rs6601299, rs6606725, rs6657707, rs67120644, rs67981690, rs6932167, rs6937722, rs6938550, rs6938647, rs7010610, rs7012891, rs7140110, rs72647336, rs72647352, rs72655677, rs72804880, rs72808908, rs72929768, rs731450, rs73196888, rs73596816, rs739320, rs73959582, rs74434374, rs74873433, rs75466392, rs75542613, rs7583698, rs75919952, rs75992720, rs7661844, rs76769796, rs7679, rs76895963, rs77281975, rs77449055, rs77524918, rs783144, rs7831557, rs7837587, rs78590292, rs79429216, rs7979473, rs799158, rs8042174, rs8090363, rs907866, rs920048, rs9271573, rs9347414, rs9368661, rs937813, rs9947678, rs998584 |
| octadecanedioate | rs12829704, rs1871395, rs2108622 |
| octanoylcarnitine | rs17304141, rs5745515, rs721204, rs7552404, rs8396 |
| Omega-3 fatty acids | rs10096633, rs10407283, rs10455872, rs1077835, rs1105654, rs11208050, rs11230741, rs11230749, rs11230759, rs11242109, rs112687416, rs114863007, rs115428654, rs11563251, rs11591147, rs11600886, rs116262432, rs11681659, rs116962591, rs116962777, rs117008109, rs117110139, rs117143374, rs117186302, rs117414940, rs117486534, rs117876551, rs117901517, rs117983270, rs118071950, rs118078695, rs11895352, rs1217616, rs12362237, rs12546944, rs1260326, rs12676079, rs12718462, rs12799017, rs12907378, rs13191810, rs13424225, rs1374117, rs137915577, rs138012803, rs138110361, rs139524394, rs139957766, rs139974673, rs141414463, rs142158911, rs143376213, rs143843429, rs143875230, rs144545816, rs145786300, rs145908530, rs146651998, rs147981159, rs148664265, rs150156331, rs150370599, rs150684478, rs151279009, rs1675090, rs16940904, rs17200189, rs17269250, rs1729377, rs17301746, rs17381383, rs174472, rs174528, rs174569, rs17798991, rs17821268, rs17821298, rs1791786, rs1791796, rs1794072, rs182611493, rs183986993, rs188370817, rs191790331, rs198442, rs198476, rs2009875, rs2074225, rs2131925, rs2141371, rs222140, rs2229094, rs2229738, rs2278426, rs2394976, rs2523659, rs2524292, rs259872, rs261291, rs2727261, rs28364531, rs28917234, rs2980874, rs2980883, rs3019553, rs34663616, rs34707604, rs34821369, rs35138338, rs35511894, rs35686293, rs35827276, rs374952396, rs3867518, rs4000713, rs417919, rs4273010, rs429358, rs4343027, rs4418728, rs4704834, rs4781682, rs4808185, rs4871602, rs4930630, rs498793, rs5026246, rs533617, rs55901398, rs579890, rs58542926, rs59752567, rs6129624, rs61896137, rs61897792, rs62036306, rs62037519, rs62039124, rs62466318, rs628993, rs629301, rs6507945, rs6587973, rs6601924, rs668948, rs6693447, rs67308910, rs673335, rs695867, rs7012891, rs7115739, rs7116318, rs7118175, rs71374758, rs71460589, rs71553276, rs72655677, rs72740858, rs72920193, rs72923298, rs72924290, rs72929768, rs73420594, rs73487492, rs7349418, rs740006, rs74974644, rs7520254, rs75916629, rs75938339, rs76059597, rs76380214, rs76755654, rs76978219, rs77022126, rs77053629, rs77407800, rs77524918, rs77535061, rs77960347, rs78353689, rs78893833, rs7924036, rs7935835, rs79429216, rs7970695, rs79787170, rs799157, rs79969098, rs8042174, rs8179219, rs886264, rs9257779, rs9268219, rs9295128, rs937813, rs938509, rs9457827, rs952275, rs9947684, rs9987289 |
| Omega-6 fatty acids | rs1002687, rs10102271, rs10214652, rs10455872, rs10458569, rs1077835, rs111809714, rs11206517, rs11250098, rs114863007, rs11591147, rs116316096, rs11662691, rs116962777, rs117159625, rs117261169, rs117901517, rs118078695, rs11828763, rs12208357, rs1260326, rs12740374, rs12907378, rs12963212, rs13108218, rs1320700, rs139524394, rs140231532, rs141414463, rs141469619, rs142158911, rs143376213, rs143843429, rs144261139, rs144545816, rs1461729, rs146576912, rs147275653, rs149511741, rs151000110, rs151330717, rs1551891, rs1692821, rs17269250, rs17301746, rs17696736, rs1787328, rs1790796, rs1800961, rs181765708, rs182611493, rs183130, rs186782888, rs188501176, rs1942417, rs1968493, rs2239013, rs2245793, rs2284178, rs2384049, rs261290, rs2740488, rs28383314, rs2844795, rs2858331, rs2976940, rs2980874, rs3130668, rs34121855, rs35106244, rs35138338, rs35311766, rs35686293, rs36018387, rs3734854, rs3770586, rs3777411, rs386453, rs395908, rs402465, rs4149307, rs429358, rs4299376, rs4426495, rs4439799, rs445093, rs4665698, rs4803748, rs486142, rs4876611, rs4921913, rs4938301, rs516226, rs522645, rs533617, rs534417, rs560898656, rs56208677, rs56322906, rs58542926, rs59774409, rs622591, rs6453106, rs6471717, rs6499863, rs6507945, rs6587973, rs6602911, rs6657707, rs668948, rs67120644, rs6859, rs6882345, rs688456, rs693, rs6934962, rs6938647, rs7005363, rs7124275, rs7139079, rs7229377, rs72655677, rs72902594, rs72929768, rs72997616, rs74018729, rs75406471, rs77437185, rs77449055, rs7750288, rs77524918, rs77960347, rs7830939, rs79429216, rs79787170, rs8042174, rs8192701, rs907866, rs9272302, rs9295128, rs9304381, rs9365100, rs937813, rs9457827, rs9457857, rs9921780 |
| Omega-7, omega-9 and saturated fatty acids | rs115849089, rs1260326, rs174418, rs1800588, rs1825955, rs9358816 |
| Other polyunsaturated fatty acids than 18:2 | rs1077835, rs111509315, rs11227881, rs112687416, rs116962591, rs11820792, rs12421620, rs12577276, rs1260326, rs139872716, rs139957766, rs143988316, rs145717049, rs174418, rs174528, rs175133, rs17762402, rs17827918, rs1791796, rs1825955, rs183986993, rs195158, rs2429862, rs2524292, rs259874, rs3019558, rs36016715, rs3741252, rs3741259, rs3741298, rs4608457, rs498793, rs528285, rs56226083, rs6591668, rs695186, rs72920193, rs73487492, rs73496248, rs740006, rs7935835, rs7936002 |
| Phosphatidylcholine and other cholines | rs1077835, rs11591147, rs1168041, rs117376818, rs1532085, rs3741298, rs4609471, rs490098, rs7203984, rs73424577, rs8100204 |
| Polyunsaturated fatty acids | rs1002687, rs10096633, rs10102271, rs10214652, rs102275, rs10407283, rs10455872, rs10458569, rs1077835, rs10888898, rs111809714, rs11206517, rs11208036, rs112866833, rs114863007, rs115413710, rs11591147, rs116316096, rs11662691, rs116878346, rs116962591, rs116962777, rs117159625, rs117261169, rs117414940, rs117901517, rs117983270, rs118078695, rs118136762, rs12208357, rs12459686, rs12462573, rs1260326, rs12721041, rs12740374, rs12907378, rs12963212, rs13108218, rs1320700, rs139524394, rs139957766, rs140824606, rs141414463, rs141469619, rs142158911, rs143376213, rs143843429, rs144261139, rs144545816, rs1461729, rs146576912, rs147275653, rs149511741, rs150423652, rs151000110, rs151330717, rs1551891, rs157595, rs1692821, rs17269250, rs17301746, rs17405319, rs174467, rs174569, rs17696736, rs1787328, rs1790796, rs1800961, rs182611493, rs183130, rs186782888, rs188501176, rs193108398, rs195161, rs198476, rs1985129, rs199717562, rs2229738, rs2239013, rs2245793, rs2284178, rs2326077, rs233716, rs2524293, rs261290, rs2740488, rs2762335, rs28383314, rs2844795, rs2858331, rs28917234, rs2976940, rs2980874, rs2980883, rs3130668, rs3130887, rs34121855, rs34821369, rs34955778, rs35138338, rs35311766, rs35511894, rs35686293, rs36018387, rs3770586, rs3777411, rs3822855, rs3843482, rs386453, rs395908, rs402465, rs413582, rs4149307, rs4299376, rs4426495, rs445093, rs4561509, rs4665698, rs4666052, rs4713841, rs4803748, rs4876611, rs4921915, rs496654, rs533617, rs534417, rs560898656, rs56322906, rs579890, rs58542926, rs59752567, rs59774409, rs6016505, rs6129629, rs62001835, rs62119320, rs6453106, rs6499863, rs6507945, rs6587973, rs6602911, rs668948, rs67120644, rs6857, rs6882345, rs688456, rs693, rs6938647, rs695867, rs7005363, rs7012891, rs7115739, rs7124275, rs7229377, rs7254133, rs72655677, rs72902594, rs72929768, rs72997616, rs73013176, rs731450, rs74018729, rs75406471, rs76116020, rs77188937, rs77524918, rs77960347, rs7830939, rs78893833, rs79429216, rs7970695, rs79787170, rs8042174, rs8192701, rs838912, rs907866, rs9267152, rs9272302, rs9295128, rs9304381, rs9365100, rs937813, rs9457827 |
| propionylcarnitine | rs10128501, rs12356193, rs2715311, rs7727544 |
| Remnant cholesterol (non-HDL, non-LDL -cholesterol) | rs1002687, rs102275, rs10401176, rs1057558, rs1081106, rs1105654, rs11057601, rs11102964, rs111278137, rs112027066, rs11206513, rs11206517, rs11208056, rs112172017, rs113588790, rs11591147, rs116316096, rs11679386, rs11691986, rs1169292, rs12151108, rs12208357, rs12546944, rs1260326, rs12740374, rs12907378, rs12916, rs12981050, rs12983316, rs1337247, rs141989097, rs144261139, rs144545816, rs145400326, rs146390218, rs146576912, rs148356565, rs1500188, rs150262789, rs151000110, rs151330717, rs1540037, rs17035665, rs174467, rs1800777, rs1836278, rs190712692, rs2015257, rs204480, rs2326077, rs247616, rs2478237, rs2570344, rs261290, rs28399637, rs2886232, rs2927439, rs2965106, rs2965162, rs2965167, rs2980874, rs35836101, rs3777411, rs3794695, rs384653, rs402465, rs41279684, rs41290120, rs4263041, rs429358, rs4299376, rs4426495, rs4665698, rs4704262, rs472495, rs4803791, rs4804510, rs4876611, rs4927206, rs4927214, rs4959063, rs4970835, rs533617, rs553427, rs55634260, rs562338, rs56261258, rs56325564, rs58542926, rs59379014, rs602662, rs611060, rs62120576, rs6511721, rs651649, rs653178, rs6587973, rs6601299, rs6602911, rs6859, rs6882345, rs693, rs6938647, rs71364511, rs71435586, rs7203984, rs7246100, rs7246666, rs7251161, rs72655677, rs72660539, rs72694391, rs72703210, rs72929768, rs73013176, rs73048351, rs7343130, rs74607435, rs7523141, rs753381, rs76186504, rs7700719, rs77278227, rs77303550, rs7737496, rs7750288, rs77542162, rs77960347, rs79429216, rs79915079, rs8042174, rs8107530, rs8113311, rs907866, rs9276826, rs9295128, rs9302635, rs9682783 |
| Saturated fatty acids | rs1002687, rs10214652, rs102275, rs10455872, rs10458569, rs10504255, rs10810374, rs10884966, rs11076175, rs1128249, rs1145210, rs11591147, rs11620783, rs116316096, rs11662059, rs116843064, rs117094150, rs117488242, rs118078695, rs12151108, rs12263369, rs12446515, rs12546944, rs1260326, rs12676079, rs12721041, rs12740374, rs12907378, rs13108218, rs139315015, rs139524394, rs139974673, rs141414463, rs141469619, rs143875230, rs144545816, rs1495741, rs16891156, rs1711041, rs17269250, rs17301746, rs182611493, rs186696265, rs186782888, rs1890896, rs1998045, rs2000999, rs2126259, rs2239013, rs2245793, rs2409836, rs2523675, rs261290, rs2740488, rs28383314, rs28917234, rs2980874, rs3094228, rs3134931, rs34052301, rs34335422, rs34894639, rs35138338, rs35686293, rs3777411, rs3846661, rs386453, rs3936511, rs402465, rs40270, rs41265930, rs4149307, rs4149455, rs429358, rs4413654, rs4426495, rs445093, rs4665698, rs4704834, rs4708870, rs523549, rs533617, rs562338, rs56322906, rs579890, rs58199976, rs58542926, rs58633957, rs59752567, rs622591, rs62496677, rs6507945, rs6587973, rs6602911, rs6657707, rs67120644, rs6847980, rs688456, rs693, rs6938647, rs7012891, rs7229377, rs72647352, rs72655677, rs72929768, rs73196888, rs7349418, rs73596816, rs73664375, rs739320, rs74018729, rs7461833, rs74873433, rs7551124, rs7559475, rs77027049, rs77082266, rs77449055, rs77524918, rs77960347, rs783144, rs78590292, rs79429216, rs79713262, rs79787170, rs7979473, rs799158, rs8042174, rs8192701, rs894210, rs907866, rs918106, rs9347414, rs937813 |
| Serum total cholesterol | rs10221768, rs10449300, rs111617668, rs113445611, rs115534052, rs11591147, rs1168041, rs12043403, rs1260326, rs138270540, rs144064722, rs145717049, rs146568567, rs1532085, rs16940213, rs17699030, rs182695896, rs185415345, rs190934192, rs2207132, rs2495477, rs2746683, rs28399657, rs2965149, rs2980853, rs3005923, rs3741298, rs395908, rs405509, rs429358, rs4426495, rs4608457, rs4609471, rs4635554, rs4803748, rs62117161, rs62120794, rs629301, rs6511721, rs6662286, rs6859, rs72664201, rs72999033, rs7412, rs7604788, rs76670936, rs77021821, rs78080497, rs79225634, rs79702288, rs952275 |
| Serum total triglycerides | rs115849089, rs1168041, rs1260326, rs1367117, rs188247550, rs2980853, rs34346326, rs429358, rs4296389, rs4608457, rs486394, rs5167, rs579674, rs72645459, rs72999033, rs9472125 |
| Sphingomyelins | rs102275, rs1057208, rs10822145, rs11066320, rs11097807, rs11102964, rs111278137, rs112027066, rs11206515, rs112172017, rs11230741, rs112952893, rs1135062, rs115740542, rs11591147, rs116843064, rs11691986, rs116962591, rs117261169, rs11751347, rs117901517, rs117983270, rs118078695, rs118136762, rs12067569, rs12151108, rs12546962, rs1260326, rs12720917, rs12740374, rs12924331, rs12974200, rs12979820, rs13107325, rs1320700, rs13337205, rs13396400, rs139957766, rs144261139, rs146576912, rs149754235, rs1500187, rs150342704, rs151000110, rs151330717, rs16940126, rs16940302, rs16962014, rs1711041, rs17301746, rs174467, rs174569, rs17699030, rs1791786, rs1800777, rs1800961, rs181765708, rs198476, rs199717562, rs204480, rs2070895, rs2245793, rs2281721, rs247615, rs2479413, rs261290, rs2642438, rs268, rs2727260, rs2740488, rs2792735, rs2820232, rs28399637, rs28399657, rs28690720, rs287227, rs28834434, rs2925979, rs2943650, rs2965162, rs333948, rs34514836, rs35138338, rs35511894, rs35633876, rs36018387, rs3741521, rs3764261, rs3768321, rs395908, rs402465, rs405509, rs4149307, rs4263041, rs429358, rs4299376, rs445093, rs4752881, rs4752973, rs4803748, rs485186, rs4970835, rs558971, rs55964643, rs56030824, rs56208677, rs562172, rs562338, rs56325564, rs56394238, rs58542926, rs5883, rs59752567, rs60318332, rs611060, rs61897792, rs62001835, rs62115552, rs62117161, rs624698, rs6499863, rs6507945, rs6511721, rs651649, rs653178, rs6586891, rs673335, rs6859, rs6882345, rs695867, rs7115739, rs7118175, rs7203984, rs7229377, rs72836561, rs73013176, rs73048351, rs73189551, rs740006, rs74018729, rs7412, rs74607435, rs75392670, rs76423146, rs77020029, rs77027049, rs77278227, rs77437185, rs77542162, rs77960347, rs78893833, rs78912080, rs78921492, rs7941030, rs79787170, rs8042174, rs8192701, rs9304381, rs949790, rs952274, rs9644636, rs984976, rs9987289 |
| tetradecanedioate | rs11045821, rs12228798, rs12317268 |
| Total cholesterol minus HDL-C | rs102275, rs10504255, rs1081106, rs11102964, rs111278137, rs112027066, rs11206513, rs11206517, rs112172017, rs1135062, rs114863007, rs11591147, rs11621792, rs11679386, rs11691986, rs1169292, rs118147862, rs12151108, rs12208357, rs12546944, rs1260326, rs12740374, rs12916, rs12979820, rs12981050, rs12983316, rs13278097, rs1337247, rs140824606, rs141989097, rs144261139, rs144545816, rs146390218, rs146576912, rs148356565, rs1499279, rs1500188, rs150262789, rs151000110, rs151330717, rs17035665, rs17249001, rs173539, rs174467, rs17696736, rs181765708, rs1836278, rs190712692, rs199717562, rs2015257, rs204480, rs204540, rs2065397, rs2570344, rs261290, rs2618566, rs2740488, rs28399637, rs2965157, rs2980874, rs34449399, rs35057129, rs35836101, rs3794695, rs3798167, rs41279684, rs4263041, rs429358, rs4299376, rs4426495, rs4507059, rs4516335, rs4704262, rs472495, rs4803748, rs4803791, rs4804510, rs4927206, rs4927214, rs4970835, rs533617, rs553427, rs55714927, rs562338, rs56325564, rs58542926, rs59379014, rs6016505, rs602662, rs60960031, rs611060, rs62118464, rs6511721, rs651649, rs6587973, rs6601299, rs6859, rs6882345, rs6920309, rs693, rs6938647, rs7246100, rs7246666, rs7249244, rs7251161, rs72655677, rs72660539, rs72703210, rs72843670, rs72902594, rs72929768, rs73013176, rs73048351, rs7343130, rs74607435, rs7523141, rs75237799, rs76186504, rs76670936, rs7700719, rs77278227, rs77303550, rs7750288, rs77542162, rs7773004, rs77960347, rs79220007, rs79429216, rs79915079, rs8042174, rs8107530, rs8113311, rs9276826, rs9295128, rs9302635 |
| Total esterified cholesterol | rs1002687, rs102275, rs10401176, rs1057558, rs10838687, rs10838724, rs11065358, rs11102964, rs111278137, rs112027066, rs11206513, rs112172017, rs112853430, rs1135062, rs114863007, rs115740542, rs11591147, rs11621792, rs116843064, rs11691986, rs12022410, rs12067569, rs12151108, rs12198895, rs12208357, rs12448528, rs1260326, rs12740374, rs12974200, rs12979820, rs12981080, rs13107325, rs1320700, rs1337247, rs140824606, rs144261139, rs144545816, rs1461729, rs146390218, rs146576912, rs1500188, rs150342704, rs151000110, rs151330717, rs16940126, rs16962014, rs17035665, rs174467, rs17696736, rs17699030, rs1800769, rs1800777, rs1800961, rs1864163, rs190712692, rs1917754, rs199717562, rs2015257, rs204480, rs219546, rs2384049, rs261290, rs2618566, rs2642438, rs268, rs2740488, rs2762335, rs2792735, rs28399637, rs2927439, rs2943650, rs2965162, rs34449399, rs34514836, rs36018387, rs3768321, rs3777411, rs3794695, rs3846662, rs402465, rs405509, rs41290120, rs4149307, rs4263041, rs429358, rs4299376, rs4307732, rs4507059, rs4605275, rs4703665, rs4704262, rs472495, rs4803766, rs485186, rs4927206, rs4959063, rs4970835, rs533617, rs553427, rs562338, rs56325564, rs58542926, rs6016505, rs611060, rs62115552, rs62118464, rs6507945, rs6511721, rs651649, rs6586891, rs6587973, rs673335, rs6859, rs6882345, rs688456, rs693, rs6938647, rs7012814, rs7229377, rs7246666, rs7251161, rs72836561, rs72902594, rs73013176, rs73048351, rs7343130, rs74607435, rs7523141, rs76186504, rs7700719, rs77278227, rs77303550, rs7750288, rs77542162, rs77960347, rs78921492, rs79429216, rs79915079, rs8042174, rs907866, rs9304381, rs9973228 |
| Total fatty acids | rs10051260, rs115849089, rs1168041, rs11820792, rs1260326, rs17414716, rs174418, rs1800588, rs1825955, rs3741298, rs429358 |
| Total phosphoglycerides | rs10468017, rs1077835, rs1168041, rs1260326, rs17414716, rs17821298, rs3741298, rs4609471, rs7203984, rs77451458, rs8100204 |
| VLDL cholesterol | rs10096633, rs10214652, rs10221768, rs10458569, rs1081106, rs11057397, rs11057601, rs11102964, rs11206517, rs1128249, rs1145210, rs11591147, rs116316096, rs116530815, rs11680233, rs116843064, rs116962777, rs117159625, rs117261169, rs12151108, rs12208357, rs12462573, rs12546944, rs1260326, rs12708983, rs12720917, rs12721041, rs12740374, rs12916, rs12983316, rs13108218, rs1337247, rs138458033, rs139524394, rs139974673, rs141414463, rs141469619, rs142084074, rs144261139, rs146576912, rs148356565, rs150856119, rs151000110, rs151330717, rs157595, rs16891156, rs17035665, rs17721559, rs1800777, rs1801177, rs1801695, rs1865834, rs186696265, rs186782888, rs187429064, rs187929675, rs1985129, rs199717562, rs203711, rs2122982, rs2186037, rs2326077, rs247615, rs247616, rs2478236, rs2523675, rs2570344, rs261290, rs28399654, rs28550053, rs28917234, rs289744, rs289752, rs2902941, rs2980874, rs2980883, rs34335269, rs34693282, rs34894639, rs35449889, rs35836101, rs369558950, rs3777411, rs3777420, rs3794695, rs386453, rs3899015, rs3936511, rs395908, rs4299376, rs437444, rs4456810, rs4635554, rs4665698, rs4704834, rs4705000, rs4708870, rs472495, rs4803748, rs4846914, rs4876611, rs4970835, rs507766, rs55634260, rs55747707, rs56208677, rs56325564, rs56393506, rs579890, rs58542926, rs59379014, rs60403635, rs611060, rs62115552, rs62117161, rs62119320, rs622591, rs635634, rs6511721, rs651649, rs654689, rs6547409, rs6587973, rs6602909, rs67120644, rs6729843, rs6857, rs693, rs6938647, rs7010610, rs7012891, rs7203984, rs7205692, rs7254133, rs72655677, rs72703210, rs72929768, rs73013176, rs73597688, rs74434374, rs74717300, rs7519205, rs7523141, rs7543163, rs75919952, rs76272805, rs77082266, rs77303550, rs7750288, rs77542162, rs79429216, rs7970695, rs799158, rs8042174, rs822928, rs907866, rs9302635, rs937813 |
| X-11445--5-alpha-pregnan-3beta,20alpha-disulfate | rs10491431, rs296381, rs4149081 |
| X-13431--nonanoylcarnitine | rs12566232, rs1509821, rs2302539, rs2539861, rs3738934, rs4672574, rs992029 |
| urate | rs10939663, rs11737601, rs4697910 |
| uridine | rs131794, rs2686796, rs532545 |
| Albumin | rs10419198, rs11128594, rs114949263, rs11589479, rs11621961, rs116933761, rs12364432, rs1260326, rs12896856, rs13108218, rs139974673, rs142385484, rs143875230, rs1461729, rs1672981, rs17696736, rs2749539, rs28929474, rs2933243, rs34284056, rs34754216, rs3740688, rs3768321, rs4886992, rs4929885, rs55772354, rs55881006, rs57274629, rs58546652, rs58895965, rs60333700, rs6734238, rs72692809, rs72829457, rs74892229, rs77542162, rs8107347, rs9912287 |
| bradykinin, des-arg(9) | rs12511874, rs2731672, rs4253243, rs4253311, rs5030062 |
| gamma-glutamylglutamine | rs1260326, rs1751956, rs2657879 |
| gamma-glutamyltyrosine | rs1512342, rs875740, rs956966 |
| Glycoprotein acetyls | rs10160799, rs10189899, rs10405357, rs10455872, rs1076158, rs11208654, rs113452856, rs113580328, rs113816145, rs115550566, rs11645475, rs11648003, rs11648622, rs1168032, rs116843064, rs116992380, rs117155836, rs117365732, rs11757660, rs117655998, rs117753658, rs117841020, rs117847715, rs117870898, rs11828763, rs12032372, rs12073837, rs1249749, rs12546944, rs12598219, rs1260326, rs13108218, rs138910759, rs139273281, rs139781980, rs140110174, rs141414463, rs143307443, rs143786320, rs144883114, rs145408202, rs148845156, rs149807892, rs150617, rs150844304, rs16971543, rs16973000, rs17105232, rs17289227, rs1801689, rs180728024, rs181810147, rs204893, rs2070634, rs2253491, rs2294915, rs236012, rs2409784, rs2434886, rs2445818, rs2497337, rs2787337, rs28834434, rs28929474, rs2925979, rs2980874, rs2980883, rs3130668, rs34010237, rs34821369, rs34881399, rs4407894, rs4666042, rs4713460, rs4841472, rs4905179, rs552489131, rs55776462, rs56041751, rs56188865, rs56212732, rs58542926, rs59254395, rs59296513, rs59774409, rs62058275, rs62131877, rs62259939, rs62466318, rs62496677, rs6452937, rs6575424, rs6601299, rs6714780, rs6717858, rs6734238, rs676210, rs6910879, rs71403860, rs7196440, rs72655677, rs72700362, rs72787084, rs72801474, rs72803196, rs7404577, rs74873433, rs76108702, rs76473767, rs76743497, rs7697204, rs77009508, rs77303550, rs77535061, rs7758790, rs78417740, rs78939685, rs7924036, rs79287178, rs79315071, rs79440651, rs79636708, rs799158, rs9295128, rs9302635, rs937813, rs9457827, rs9921494, rs9922718, rs9930140, rs9938539 |
| Glycoproteins | rs10083777, rs10208743, rs1047891, rs10739076, rs10739110, rs10815428, rs10932472, rs112085022, rs113363823, rs114210411, rs114338044, rs114808769, rs115671969, rs116181500, rs117182696, rs117825338, rs118096183, rs11901164, rs12338205, rs12694211, rs13007126, rs13009426, rs13026255, rs13298772, rs139728050, rs140360589, rs141705179, rs142843919, rs143955150, rs146169358, rs146851439, rs1470498, rs148041738, rs149877929, rs17748227, rs1864353, rs186757218, rs190126699, rs193220212, rs1992855, rs2169387, rs2282162, rs2287598, rs2302908, rs2371011, rs2542929, rs34454501, rs41272663, rs4673618, rs55663096, rs56212035, rs62177972, rs62203712, rs62203740, rs62568995, rs6435548, rs67225130, rs6752652, rs6756888, rs7012637, rs72930538, rs72935806, rs72949273, rs7425448, rs74753815, rs7570368, rs75764781, rs7582376, rs77039478, rs77598098, rs78600267, rs78815845 |
| HWESASXX | rs2007084, rs7535263, rs893616 |
| X-12244--N-acetylcarnosine | rs2484984, rs282115, rs4148421, rs6804368, rs7775554, rs8002180 |
| X-02269 | rs11188148, rs1165196, rs2071426 |
| X-03094 | rs1260326, rs2187375, rs6679002 |
| X-08402 | rs10140289, rs1271565, rs12881815, rs4814176, rs7157785, rs8022917 |
| X-08988 | rs10938307, rs13021675, rs715 |
| X-10510 | rs3762660, rs4814176, rs4902243 |
| X-11204 | rs10468017, rs2935461, rs8033940 |
| X-11261 | rs1171614, rs3798167, rs648253, rs6939741, rs9457843 |
| X-11315 | rs1809148, rs3761097, rs4327428 |
| X-11440 | rs11670965, rs2932766, rs296381, rs3732218, rs4802397 |
| X-11441 | rs3796092, rs4148324, rs6717546, rs7608713 |
| X-11442 | rs28899194, rs3796092, rs4148324, rs6723936, rs7608713, rs838705 |
| X-11444 | rs10199511, rs2273664, rs7604035 |
| X-11469 | rs11188148, rs11754288, rs2071426, rs4149081 |
| X-11491 | rs11045824, rs12645107, rs4149056, rs7975166 |
| X-11529 | rs10505870, rs10770803, rs10841797, rs11045824, rs17617462, rs4149021, rs4149056, rs7137767, rs7138177, rs7975166, rs974453 |
| X-11530 | rs17863762, rs2018609, rs28900385, rs4047189, rs6723936, rs7608713, rs838718, rs887829 |
| X-11538 | rs10505870, rs11045824, rs11834359, rs12228798, rs1871395, rs2108622, rs2291073 |
| X-11787 | rs17110031, rs17349853, rs17725971, rs1891071, rs1934963, rs2298037, rs869927 |
| X-11792 | rs2731672, rs4253252, rs698078 |
| X-11905 | rs10505870, rs12370842, rs4149056 |
| X-12063 | rs10242455, rs11045825, rs11762932, rs13230554, rs2291073, rs296396, rs4149056, rs4917639, rs6465760, rs6544713, rs7138177, rs7795644, rs7797978, rs952319 |
| X-12092 | rs1061134, rs1061135, rs10748725, rs11189692, rs11595763, rs12415538, rs12780272, rs1365, rs4917817, rs4919221, rs542386, rs7096654, rs754586, rs8101881, rs942800, rs942812 |
| X-12093 | rs10469966, rs2070581, rs4919209 |
| X-12456 | rs12829704, rs2547231, rs4149056 |
| X-12556 | rs2235649, rs4129624, rs4675874, rs7590032 |
| X-12644 | rs1077835, rs1532085, rs7969341 |
| X-12696 | rs12465802, rs13005339, rs3800993, rs7568884, rs7570971 |
| X-12728 | rs12326998, rs12769714, rs13094553, rs16896783, rs1912320, rs2830801, rs8089543, rs9566930 |
| X-12798 | rs10945673, rs1171615, rs1171650, rs1367211, rs316020, rs316032, rs316174, rs3799344, rs532440, rs628031, rs791174, rs9456505 |
| X-12844 | rs11666603, rs17614939, rs2035647, rs430200 |
| X-13429 | rs11045823, rs12317268, rs12810446, rs296381 |
| X-13435 | rs2062541, rs6597030, rs9392556 |
| X-14626 | rs10770797, rs11045834, rs12317268 |

Abbreviations: MR, Mendelian randomization; SNP, single nucleotide polymorphism.

**Table S3. Detailed description of the genetic instruments for blood metabolites used in stage two of the MR study.**

| **Metabolites** | **SNPs used in stage two** |
| --- | --- |
| X-11593--O-methylascorbate | rs10412803, rs11089325, rs16982844, rs2686184, rs3804043, rs438798, rs4597638, rs4680, rs7290062, rs7707010, rs887200, rs9318225, rs9606212 |
| Sphingomyelins | rs102275, rs1057208, rs10822145, rs11066320, rs11097807, rs11102964, rs111278137, rs112027066, rs11206515, rs112172017, rs11230741, rs112952893, rs1135062, rs115740542, rs11591147, rs116843064, rs11691986, rs116962591, rs117261169, rs11751347, rs117901517, rs117983270, rs118078695, rs118136762, rs12067569, rs12151108, rs12546962, rs1260326, rs12720917, rs12740374, rs12924331, rs12974200, rs12979820, rs13107325, rs1320700, rs13337205, rs13396400, rs139957766, rs144261139, rs146576912, rs149754235, rs1500187, rs150342704, rs151000110, rs151330717, rs16940126, rs16940302, rs16962014, rs1711041, rs17301746, rs174467, rs174569, rs17699030, rs1791786, rs1800777, rs1800961, rs181765708, rs198476, rs199717562, rs204480, rs2070895, rs2245793, rs2281721, rs247615, rs2479413, rs261290, rs2642438, rs268, rs2727260, rs2740488, rs2792735, rs2820232, rs28399637, rs28399657, rs28690720, rs287227, rs28834434, rs2925979, rs2943650, rs2965162, rs333948, rs34514836, rs35138338, rs35511894, rs35633876, rs36018387, rs3741521, rs3764261, rs3768321, rs395908, rs402465, rs405509, rs4149307, rs4263041, rs429358, rs4299376, rs445093, rs4752881, rs4752973, rs4803748, rs485186, rs4970835, rs558971, rs55964643, rs56030824, rs56208677, rs562172, rs562338, rs56325564, rs56394238, rs58542926, rs5883, rs59752567, rs60318332, rs611060, rs61897792, rs62001835, rs62115552, rs62117161, rs624698, rs6499863, rs6507945, rs6511721, rs651649, rs653178, rs6586891, rs673335, rs6859, rs6882345, rs695867, rs7115739, rs7118175, rs7203984, rs7229377, rs72836561, rs73013176, rs73048351, rs73189551, rs740006, rs74018729, rs7412, rs74607435, rs75392670, rs76423146, rs77020029, rs77027049, rs77278227, rs77437185, rs77542162, rs77960347, rs78893833, rs78912080, rs78921492, rs7941030, rs79787170, rs8042174, rs8192701, rs9304381, rs949790, rs952274, rs9644636, rs984976, rs9987289 |
| Clinical LDL cholesterol | rs102275, rs10901256, rs10953298, rs11102964, rs11127048, rs111278137, rs112027066, rs11206513, rs11206517, rs112172017, rs112481437, rs1135062, rs113588790, rs115740542, rs11591147, rs11621792, rs11691986, rs117261169, rs118147862, rs12151108, rs12208357, rs12740374, rs12981555, rs1337247, rs140824606, rs141645791, rs144261139, rs144545816, rs1461729, rs146576912, rs148356565, rs1499279, rs1500188, rs150262789, rs151000110, rs151330717, rs1661174, rs17035665, rs17249001, rs173539, rs174467, rs17696736, rs181765708, rs1836278, rs199717562, rs2015257, rs204480, rs204540, rs2065397, rs2272272, rs241440, rs2479413, rs2523764, rs2570344, rs2618566, rs2642438, rs2740488, rs28399637, rs2865505, rs2927439, rs2965157, rs2965167, rs34449399, rs35057129, rs35823804, rs35836101, rs3794695, rs3798167, rs3846662, rs405509, rs41279684, rs4263041, rs429358, rs4299376, rs4507059, rs4703665, rs4704262, rs4722551, rs4802216, rs4803791, rs4804510, rs4927206, rs4927214, rs4970835, rs533617, rs553427, rs55714927, rs562338, rs56325564, rs58542926, rs59379014, rs6016505, rs602662, rs60960031, rs611060, rs62115552, rs62116948, rs62118464, rs62120793, rs6511721, rs651649, rs6859, rs6882345, rs6920309, rs693, rs7012814, rs7246666, rs7251161, rs7254892, rs72660539, rs73013176, rs73036542, rs73048351, rs7343130, rs74607435, rs7523141, rs76186504, rs76670936, rs7700719, rs77278227, rs77303550, rs7750288, rs77542162, rs7773004, rs78912080, rs79915079, rs8113311, rs9749236, rs9973228 |

Abbreviation: MR, Mendelian randomization; SNP, single nucleotide polymorphism.

**Table S4. 678 diseases incorporated in the Phe-MR analysis.**

| **Phenotype Description** | **Disease Category** | **PheCode** | **Number of excluded controls** | **Number of cases** | **Number of controls** |
| --- | --- | --- | --- | --- | --- |
| Intestinal infection | infectious diseases | 8 | 0 | 8,991 | 399,970 |
| Bacterial enteritis | infectious diseases | 8.5 | 6,254 | 2,737 | 399,970 |
| Intestinal infection due to C. difficile | infectious diseases | 8.52 | 8,341 | 650 | 399,970 |
| Viral Enteritis | infectious diseases | 8.6 | 8,129 | 862 | 399,970 |
| Septicemia | infectious diseases | 38 | 11,059 | 4,005 | 393,897 |
| Gram negative septicemia | infectious diseases | 38.1 | 14,232 | 832 | 393,897 |
| Bacterial infection NOS | infectious diseases | 41 | 2,877 | 12,187 | 393,897 |
| Staphylococcus infections | infectious diseases | 41.1 | 11,915 | 3,149 | 393,897 |
| Streptococcus infection | infectious diseases | 41.2 | 13,420 | 1,644 | 393,897 |
| E. coli | infectious diseases | 41.4 | 12,320 | 2,744 | 393,897 |
| Viral hepatitis | infectious diseases | 70 | 4,430 | 1,215 | 403,316 |
| Viral warts & HPV | infectious diseases | 78 | 4,638 | 1,007 | 403,316 |
| Viral infection | infectious diseases | 79 | 2,839 | 2,806 | 403,316 |
| Postoperative infection | infectious diseases | 80 | 2,129 | 4,489 | 402,343 |
| Infection/inflammation of internal prosthetic device; implant; and graft | infectious diseases | 81 | 4,133 | 2,485 | 402,343 |
| Candidiasis | infectious diseases | 112 | 526 | 2,134 | 406,301 |
| Cancer of mouth | neoplasms | 145 | 1,497 | 643 | 406,821 |
| Cancer of larynx, pharynx, nasal cavities | neoplasms | 149 | 1,512 | 628 | 406,821 |
| Cancer of esophagus | neoplasms | 150 | 14,869 | 720 | 393,372 |
| Cancer of stomach | neoplasms | 151 | 15,035 | 554 | 393,372 |
| Colorectal cancer | neoplasms | 153 | 21,643 | 4,562 | 382,756 |
| Colon cancer | neoplasms | 153.2 | 23,154 | 3,051 | 382,756 |
| Malignant neoplasm of rectum, rectosigmoid junction, and anus | neoplasms | 153.3 | 24,110 | 2,095 | 382,756 |
| Pancreatic cancer | neoplasms | 157 | 15,000 | 589 | 393,372 |
| Neoplasm of unspecified nature of digestive system | neoplasms | 158 | 14,533 | 1,056 | 393,372 |
| Malignant neoplasm of other and ill-defined sites within the digestive organs and peritoneum | neoplasms | 159 | 10,005 | 5,584 | 393,372 |
| Cancer within the respiratory system | neoplasms | 165 | 35 | 2,700 | 406,226 |
| Cancer of bronchus; lung | neoplasms | 165.1 | 634 | 2,101 | 406,226 |
| Skin cancer | neoplasms | 172 | 138 | 13,752 | 395,071 |
| Melanomas of skin, dx or hx | neoplasms | 172.1 | 11,199 | 2,691 | 395,071 |
| Melanomas of skin | neoplasms | 172.11 | 11,199 | 2,691 | 395,071 |
| Other non-epithelial cancer of skin | neoplasms | 172.2 | 2,741 | 11,149 | 395,071 |
| Carcinoma in situ of skin | neoplasms | 172.3 | 13,223 | 667 | 395,071 |
| Breast cancer | neoplasms | 174 | 7,514 | 12,898 | 388,549 |
| Cancer of urinary organs (incl. kidney and bladder) | neoplasms | 189 | 0 | 4,165 | 404,796 |
| Cancer of kidney and renal pelvis | neoplasms | 189.1 | 3,120 | 1,045 | 404,796 |
| Malignant neoplasm of kidney, except pelvis | neoplasms | 189.11 | 3,163 | 1,002 | 404,796 |
| Cancer of bladder | neoplasms | 189.2 | 1,738 | 2,427 | 404,796 |
| Malignant neoplasm of bladder | neoplasms | 189.21 | 2,019 | 2,146 | 404,796 |
| Manlignant and unknown neoplasms of brain and nervous system | neoplasms | 191 | 1,067 | 655 | 407,239 |
| Cancer of brain and nervous system | neoplasms | 191.1 | 1,191 | 531 | 407,239 |
| Cancer, suspected or other | neoplasms | 195 | 21,632 | 16,725 | 370,604 |
| Malignant neoplasm, other | neoplasms | 195.1 | 22,378 | 15,979 | 370,604 |
| Chemotherapy | neoplasms | 197 | 16,559 | 21,798 | 370,604 |
| Secondary malignant neoplasm | neoplasms | 198 | 28,874 | 9,483 | 370,604 |
| Secondary malignancy of lymph nodes | neoplasms | 198.1 | 32,978 | 5,379 | 370,604 |
| Secondary malignancy of respiratory organs | neoplasms | 198.2 | 36,146 | 2,211 | 370,604 |
| Secondary malignant neoplasm of digestive systems | neoplasms | 198.3 | 36,838 | 1,519 | 370,604 |
| Secondary malignant neoplasm of liver | neoplasms | 198.4 | 35,719 | 2,638 | 370,604 |
| Secondary malignancy of brain/spine | neoplasms | 198.5 | 37,551 | 806 | 370,604 |
| Secondary malignancy of bone | neoplasms | 198.6 | 36,206 | 2,151 | 370,604 |
| Neoplasm of uncertain behavior | neoplasms | 199 | 36,945 | 1,412 | 370,604 |
| Myeloproliferative disease | neoplasms | 200 | 3,500 | 995 | 404,466 |
| Cancer of other lymphoid, histiocytic tissue | neoplasms | 202 | 2,225 | 2,270 | 404,466 |
| Non-Hodgkins lymphoma | neoplasms | 202.2 | 2,702 | 1,793 | 404,466 |
| Large cell lymphoma | neoplasms | 202.24 | 3,922 | 573 | 404,466 |
| Leukemia | neoplasms | 204 | 2,834 | 1,661 | 404,466 |
| Lymphoid leukemia | neoplasms | 204.1 | 3,917 | 578 | 404,466 |
| Lymphoid leukemia, chronic | neoplasms | 204.12 | 3,989 | 506 | 404,466 |
| Multiple myeloma | neoplasms | 204.4 | 3,943 | 552 | 404,466 |
| Benign neoplasm of colon | neoplasms | 208 | 2,746 | 20,204 | 386,011 |
| Benign neoplasm of lip, oral cavity, and pharynx | neoplasms | 210 | 1,156 | 984 | 406,821 |
| Benign neoplasm of other parts of digestive system | neoplasms | 211 | 8,380 | 5,280 | 395,301 |
| Lipoma | neoplasms | 214 | 1,077 | 6,271 | 401,613 |
| Lipoma of skin and subcutaneous tissue | neoplasms | 214.1 | 2,737 | 4,611 | 401,613 |
| Other benign neoplasm of connective and other soft tissue | neoplasms | 215 | 6,238 | 1,110 | 401,613 |
| Benign neoplasm of skin | neoplasms | 216 | 621 | 7,722 | 400,618 |
| Vascular hamartomas and non-neoplastic nevi | neoplasms | 217 | 7,694 | 649 | 400,618 |
| Nevus, non-neoplastic | neoplasms | 217.1 | 7,746 | 597 | 400,618 |
| Benign neoplasm of brain and other parts of nervous system | neoplasms | 225 | 894 | 828 | 407,239 |
| Benign neoplasm of brain, cranial nerves, meninges | neoplasms | 225.1 | 948 | 774 | 407,239 |
| Benign neoplasm of other endocrine glands and related structures | neoplasms | 227 | 686 | 876 | 407,399 |
| Hemangioma and lymphangioma, any site | neoplasms | 228 | 0 | 1,603 | 407,358 |
| Benign neoplasm of unspecified sites | neoplasms | 229 | 0 | 2,402 | 406,559 |
| Simple and unspecified goiter | endocrine/metabolic | 240 | 16,930 | 602 | 391,429 |
| Nontoxic nodular goiter | endocrine/metabolic | 241 | 16,389 | 1,143 | 391,429 |
| Nontoxic multinodular goiter | endocrine/metabolic | 241.2 | 16,852 | 680 | 391,429 |
| Hypothyroidism | endocrine/metabolic | 244 | 2,661 | 14,871 | 391,429 |
| Secondary hypothyroidism | endocrine/metabolic | 244.1 | 16,415 | 1,117 | 391,429 |
| Hypothyroidism NOS | endocrine/metabolic | 244.4 | 3,361 | 14,171 | 391,429 |
| Diabetes mellitus | endocrine/metabolic | 250 | 2 | 20,203 | 388,756 |
| Type 1 diabetes | endocrine/metabolic | 250.1 | 17,545 | 2,660 | 388,756 |
| Type 2 diabetes | endocrine/metabolic | 250.2 | 1,260 | 18,945 | 388,756 |
| Type 2 diabetes with ophthalmic manifestations | endocrine/metabolic | 250.23 | 18,907 | 1,298 | 388,756 |
| Type 2 diabetes with neurological manifestations | endocrine/metabolic | 250.24 | 19,630 | 575 | 388,756 |
| Abnormal glucose | endocrine/metabolic | 250.4 | 19,520 | 685 | 388,756 |
| Diabetic retinopathy | endocrine/metabolic | 250.7 | 10,763 | 1,339 | 396,859 |
| Other disorders of pancreatic internal secretion | endocrine/metabolic | 251 | 2,632 | 943 | 405,386 |
| Hypoglycemia | endocrine/metabolic | 251.1 | 21,703 | 939 | 386,319 |
| Disorders of parathyroid gland | endocrine/metabolic | 252 | 2,698 | 877 | 405,386 |
| Hyperparathyroidism | endocrine/metabolic | 252.1 | 2,794 | 781 | 405,386 |
| Disorders of the pituitary gland and its hypothalamic control | endocrine/metabolic | 253 | 2,882 | 693 | 405,386 |
| Disorders of adrenal glands | endocrine/metabolic | 255 | 2,933 | 642 | 405,386 |
| Protein-calorie malnutrition | endocrine/metabolic | 260 | 1,412 | 1,057 | 406,492 |
| Anorexia | endocrine/metabolic | 260.6 | 1,634 | 835 | 406,492 |
| Vitamin deficiency | endocrine/metabolic | 261 | 1,261 | 1,208 | 406,492 |
| Vitamin B-complex deficiencies | endocrine/metabolic | 261.2 | 1,715 | 754 | 406,492 |
| Disorders of lipid metabolism | endocrine/metabolic | 272 | 0 | 35,927 | 373,034 |
| Hyperlipidemia | endocrine/metabolic | 272.1 | 83 | 35,844 | 373,034 |
| Hypercholesterolemia | endocrine/metabolic | 272.11 | 2,685 | 33,242 | 373,034 |
| Gout and other crystal arthropathies | endocrine/metabolic | 274 | 0 | 3,763 | 405,198 |
| Gout | endocrine/metabolic | 274.1 | 568 | 3,195 | 405,198 |
| Crystal arthropathies | endocrine/metabolic | 274.2 | 3,147 | 616 | 405,198 |
| Chondrocalcinosis | endocrine/metabolic | 274.21 | 3,203 | 560 | 405,198 |
| Disorders of mineral metabolism | endocrine/metabolic | 275 | 0 | 2,127 | 406,834 |
| Disorders of iron metabolism | hematopoietic | 275.1 | 1,458 | 669 | 406,834 |
| Disorders of calcium/phosphorus metabolism | endocrine/metabolic | 275.5 | 923 | 1,204 | 406,834 |
| Disorders of fluid, electrolyte, and acid-base balance | endocrine/metabolic | 276 | 0 | 7,455 | 401,506 |
| Electrolyte imbalance | endocrine/metabolic | 276.1 | 3,332 | 4,123 | 401,506 |
| Hyperpotassemia | endocrine/metabolic | 276.13 | 6,480 | 975 | 401,506 |
| Hypopotassemia | endocrine/metabolic | 276.14 | 6,025 | 1,430 | 401,506 |
| Acid-base balance disorder | endocrine/metabolic | 276.4 | 6,294 | 1,161 | 401,506 |
| Acidosis | endocrine/metabolic | 276.41 | 6,400 | 1,055 | 401,506 |
| Hypovolemia | endocrine/metabolic | 276.5 | 4,621 | 2,834 | 401,506 |
| Other disorders of metabolism | endocrine/metabolic | 277 | 0 | 1,424 | 407,537 |
| Overweight, obesity and other hyperalimentation | endocrine/metabolic | 278 | 0 | 10,968 | 397,993 |
| Obesity | endocrine/metabolic | 278.1 | 169 | 10,799 | 397,993 |
| Iron deficiency anemias | hematopoietic | 280 | 11,148 | 7,787 | 390,026 |
| Iron deficiency anemias, unspecified or not due to blood loss | hematopoietic | 280.1 | 11,521 | 7,414 | 390,026 |
| Other deficiency anemia | hematopoietic | 281 | 17,802 | 1,133 | 390,026 |
| Megaloblastic anemia | hematopoietic | 281.1 | 17,859 | 1,076 | 390,026 |
| Pernicious anemia | hematopoietic | 281.11 | 18,181 | 754 | 390,026 |
| Other anemias | hematopoietic | 285 | 6,679 | 12,256 | 390,026 |
| Anemia of chronic disease | hematopoietic | 285.2 | 18,233 | 702 | 390,026 |
| Coagulation defects | hematopoietic | 286 | 1,739 | 941 | 406,281 |
| Purpura and other hemorrhagic conditions | hematopoietic | 287 | 889 | 1,791 | 406,281 |
| Thrombocytopenia | hematopoietic | 287.3 | 1,117 | 1,563 | 406,281 |
| Diseases of white blood cells | hematopoietic | 288 | 3,798 | 3,788 | 401,375 |
| Decreased white blood cell count | hematopoietic | 288.1 | 4,402 | 3,184 | 401,375 |
| Neutropenia | hematopoietic | 288.11 | 4,402 | 3,184 | 401,375 |
| Other diseases of blood and blood-forming organs | hematopoietic | 289 | 3,409 | 4,177 | 401,375 |
| Lymphadenitis | hematopoietic | 289.4 | 4,964 | 2,622 | 401,375 |
| Diseases of spleen | hematopoietic | 289.5 | 7,071 | 515 | 401,375 |
| Dementias | mental disorders | 290.1 | 5,622 | 956 | 402,383 |
| Other specified nonpsychotic and/or transient mental disorders | mental disorders | 291 | 5,937 | 641 | 402,383 |
| Neurological disorders | mental disorders | 292 | 1,923 | 4,655 | 402,383 |
| Aphasia/speech disturbance | mental disorders | 292.1 | 5,064 | 1,514 | 402,383 |
| Memory loss | mental disorders | 292.3 | 5,878 | 700 | 402,383 |
| Altered mental status | mental disorders | 292.4 | 4,389 | 2,189 | 402,383 |
| Symptoms involving head and neck | mental disorders | 293 | 0 | 2,986 | 405,975 |
| Swelling, mass, or lump in head and neck [Space occupying lesion, intracranial NOS] | mental disorders | 293.1 | 2,079 | 907 | 405,975 |
| Schizophrenia and other psychotic disorders | mental disorders | 295 | 42,635 | 850 | 365,476 |
| Schizophrenia | mental disorders | 295.1 | 42,914 | 571 | 365,476 |
| Mood disorders | mental disorders | 296 | 30,925 | 12,560 | 365,476 |
| Bipolar | mental disorders | 296.1 | 42,421 | 1,064 | 365,476 |
| Depression | mental disorders | 296.2 | 31,584 | 11,901 | 365,476 |
| Anxiety disorders | mental disorders | 300 | 36,546 | 6,939 | 365,476 |
| Anxiety disorder | mental disorders | 300.1 | 37,110 | 6,375 | 365,476 |
| Agorophobia, social phobia, and panic disorder | mental disorders | 300.12 | 42,776 | 709 | 365,476 |
| Phobia | mental disorders | 300.13 | 42,982 | 503 | 365,476 |
| Psychogenic and somatoform disorders | mental disorders | 303 | 42,956 | 529 | 365,476 |
| Other mental disorder | mental disorders | 306 | 14,694 | 28,791 | 365,476 |
| Alcohol-related disorders | mental disorders | 317 | 16,684 | 12,922 | 379,355 |
| Alcoholism | mental disorders | 317.1 | 20,638 | 8,968 | 379,355 |
| Alcoholic liver damage | mental disorders | 317.11 | 28,804 | 802 | 379,355 |
| Tobacco use disorder | mental disorders | 318 | 9,826 | 19,780 | 379,355 |
| Sleep disorders | neurological | 327 | 0 | 5,238 | 403,723 |
| Sleep apnea | neurological | 327.3 | 767 | 4,471 | 403,723 |
| Other cerebral degenerations | neurological | 331 | 12,640 | 1,112 | 395,209 |
| Parkinson's disease | neurological | 332 | 12,625 | 1,127 | 395,209 |
| Extrapyramidal disease and abnormal movement disorders | neurological | 333 | 12,861 | 891 | 395,209 |
| Degenerative disease of the spinal cord | neurological | 334 | 11,963 | 1,789 | 395,209 |
| Multiple sclerosis | neurological | 335 | 12,396 | 1,356 | 395,209 |
| Pain | symptoms | 338 | 0 | 771 | 408,190 |
| Other headache syndromes | neurological | 339 | 2,290 | 7,891 | 398,780 |
| Migraine | neurological | 340 | 7,311 | 2,870 | 398,780 |
| Hemiplegia | neurological | 342 | 12,252 | 1,500 | 395,209 |
| Other paralytic syndromes | neurological | 344 | 13,092 | 660 | 395,209 |
| Epilepsy, recurrent seizures, convulsions | neurological | 345 | 8,665 | 5,087 | 395,209 |
| Epilepsy | neurological | 345.1 | 12,851 | 901 | 395,209 |
| Convulsions | neurological | 345.3 | 11,520 | 2,232 | 395,209 |
| Other conditions of brain | neurological | 348 | 12,326 | 1,426 | 395,209 |
| Abnormal movement | neurological | 350 | 0 | 2,744 | 406,217 |
| Abnormal involuntary movements | neurological | 350.1 | 1,856 | 888 | 406,217 |
| Abnormality of gait | neurological | 350.2 | 1,143 | 1,601 | 406,217 |
| Other peripheral nerve disorders | neurological | 351 | 2,302 | 12,592 | 394,067 |
| Disorders of other cranial nerves | neurological | 352 | 13,501 | 1,393 | 394,067 |
| Facial nerve disorders [CN7] | neurological | 352.2 | 14,014 | 880 | 394,067 |
| Nerve root and plexus disorders | neurological | 353 | 13,755 | 1,139 | 394,067 |
| Inflammatory and toxic neuropathy | neurological | 357 | 741 | 1,368 | 406,852 |
| Retinal detachments and defects | sense organs | 361 | 7,937 | 3,263 | 397,761 |
| Retinal detachment with retinal defect | sense organs | 361.1 | 9,808 | 1,392 | 397,761 |
| Other retinal disorders | sense organs | 362 | 8,235 | 3,867 | 396,859 |
| Degeneration of macula and posterior pole of retina | sense organs | 362.2 | 9,911 | 2,191 | 396,859 |
| Macular degeneration (senile) of retina NOS | sense organs | 362.29 | 9,914 | 2,188 | 396,859 |
| Retinal vascular changes and abnomalities | sense organs | 362.4 | 11,253 | 849 | 396,859 |
| Corneal opacity and other disorders of cornea | sense organs | 364 | 10,468 | 732 | 397,761 |
| Glaucoma | sense organs | 365 | 6,738 | 4,462 | 397,761 |
| Open-angle glaucoma | sense organs | 365.1 | 10,157 | 1,043 | 397,761 |
| Primary open angle glaucoma | sense organs | 365.11 | 10,163 | 1,037 | 397,761 |
| Primary angle-closure glaucoma | sense organs | 365.2 | 10,495 | 705 | 397,761 |
| Cataract | sense organs | 366 | 0 | 20,352 | 388,609 |
| Senile cataract | sense organs | 366.2 | 11,983 | 8,369 | 388,609 |
| Disorders of refraction and accommodation; blindness and low vision | sense organs | 367 | 0 | 2,431 | 406,530 |
| Myopia | sense organs | 367.1 | 1,174 | 1,257 | 406,530 |
| Blindness and low vision | sense organs | 367.9 | 1,708 | 723 | 406,530 |
| Visual disturbances | sense organs | 368 | 0 | 3,307 | 405,654 |
| Amblyopia | sense organs | 368.1 | 2,769 | 538 | 405,654 |
| Diplopia and disorders of binocular vision | sense organs | 368.2 | 2,575 | 732 | 405,654 |
| Subjective visual disturbances | sense organs | 368.9 | 2,692 | 615 | 405,654 |
| Infection of the eye | sense organs | 369 | 9,095 | 560 | 399,306 |
| Inflammation of the eye | sense organs | 371 | 6,481 | 3,174 | 399,306 |
| Inflammation of eyelids | sense organs | 371.3 | 7,259 | 2,396 | 399,306 |
| Disorders of conjunctiva | sense organs | 372 | 8,890 | 765 | 399,306 |
| Other disorders of eyelids | sense organs | 374 | 3,929 | 5,726 | 399,306 |
| Ectropion or entropion | sense organs | 374.1 | 8,587 | 1,068 | 399,306 |
| Disorders of lacrimal system | sense organs | 375 | 5,498 | 2,218 | 401,245 |
| Epiphora | sense organs | 375.2 | 6,817 | 899 | 401,245 |
| Strabismus and other disorders of binocular eye movements | sense organs | 378 | 6,274 | 1,442 | 401,245 |
| Strabismus (not specified as paralytic) | sense organs | 378.1 | 6,798 | 918 | 401,245 |
| Other disorders of eye | sense organs | 379 | 3,806 | 3,910 | 401,245 |
| Disorders of vitreous body | sense organs | 379.2 | 6,344 | 1,372 | 401,245 |
| Aphakia and other disorders of lens | sense organs | 379.3 | 5,891 | 1,825 | 401,245 |
| Disorders of external ear | sense organs | 380 | 0 | 1,451 | 407,510 |
| Otitis externa | sense organs | 380.1 | 927 | 524 | 407,510 |
| Otitis media and Eustachian tube disorders | sense organs | 381 | 1,814 | 2,259 | 404,888 |
| Otitis media | sense organs | 381.1 | 2,249 | 1,824 | 404,888 |
| Suppurative and unspecified otitis media | sense organs | 381.11 | 3,217 | 856 | 404,888 |
| Other disorders of tympanic membrane | sense organs | 384 | 2,709 | 1,364 | 404,888 |
| Perforation of tympanic membrane | sense organs | 384.4 | 3,029 | 1,044 | 404,888 |
| Other disorders of middle ear and mastoid | sense organs | 385 | 3,245 | 828 | 404,888 |
| Cholesteatoma | sense organs | 385.3 | 3,465 | 608 | 404,888 |
| Vertiginous syndromes and other disorders of vestibular system | sense organs | 386 | 0 | 6,134 | 402,827 |
| Meniere's disease | sense organs | 386.1 | 5,556 | 578 | 402,827 |
| Labyrinthitis | sense organs | 386.3 | 5,367 | 767 | 402,827 |
| Dizziness and giddiness (Light-headedness and vertigo) | sense organs | 386.9 | 1,523 | 4,611 | 402,827 |
| Hearing loss | sense organs | 389 | 143 | 4,256 | 404,562 |
| Tinnitus | sense organs | 389.4 | 3,884 | 515 | 404,562 |
| Rheumatic disease of the heart valves | circulatory system | 394 | 1,645 | 4,895 | 402,421 |
| Mitral valve disease | circulatory system | 394.2 | 3,555 | 2,985 | 402,421 |
| Aortic valve disease | circulatory system | 394.3 | 5,280 | 1,260 | 402,421 |
| Disease of tricuspid valve | circulatory system | 394.7 | 5,482 | 1,058 | 402,421 |
| Heart valve disorders | circulatory system | 395 | 2,301 | 4,239 | 402,421 |
| Nonrheumatic mitral valve disorders | circulatory system | 395.1 | 3,648 | 2,892 | 402,421 |
| Heart valve replaced | circulatory system | 395.6 | 5,041 | 1,499 | 402,421 |
| Abnormal heart sounds | circulatory system | 396 | 5,491 | 1,049 | 402,421 |
| Hypertension | circulatory system | 401 | 618 | 77,977 | 330,366 |
| Essential hypertension | circulatory system | 401.1 | 872 | 77,723 | 330,366 |
| Hypertensive heart and/or renal disease | circulatory system | 401.2 | 76,876 | 1,719 | 330,366 |
| Hypertensive chronic kidney disease | circulatory system | 401.22 | 77,047 | 1,548 | 330,366 |
| Elevated blood pressure reading without diagnosis of hypertension | circulatory system | 402 | 77,108 | 1,487 | 330,366 |
| Ischemic Heart Disease | circulatory system | 411 | 503 | 31,355 | 377,103 |
| Unstable angina (intermediate coronary syndrome) | circulatory system | 411.1 | 26,677 | 5,181 | 377,103 |
| Myocardial infarction | circulatory system | 411.2 | 20,155 | 11,703 | 377,103 |
| Angina pectoris | circulatory system | 411.3 | 15,683 | 16,175 | 377,103 |
| Coronary atherosclerosis | circulatory system | 411.4 | 11,835 | 20,023 | 377,103 |
| Aneurysm and dissection of heart | circulatory system | 411.41 | 31,160 | 698 | 377,103 |
| Other chronic ischemic heart disease, unspecified | circulatory system | 411.8 | 16,937 | 14,921 | 377,103 |
| Other acute and subacute forms of ischemic heart disease | circulatory system | 411.9 | 30,689 | 1,169 | 377,103 |
| Other forms of chronic heart disease | circulatory system | 414 | 30,062 | 1,796 | 377,103 |
| Pulmonary heart disease | circulatory system | 415 | 2,329 | 4,257 | 402,375 |
| Chronic pulmonary heart disease | circulatory system | 415.2 | 5,996 | 590 | 402,375 |
| Cardiomegaly | circulatory system | 416 | 4,013 | 2,573 | 402,375 |
| Nonspecific chest pain | circulatory system | 418 | 0 | 31,429 | 377,532 |
| Precordial pain | circulatory system | 418.1 | 27,847 | 3,582 | 377,532 |
| Pericarditis | circulatory system | 420.2 | 1,909 | 1,273 | 405,779 |
| Endocarditis | circulatory system | 420.3 | 2,496 | 686 | 405,779 |
| Cardiomyopathy | circulatory system | 425 | 1,935 | 1,247 | 405,779 |
| Primary/intrinsic cardiomyopathies | circulatory system | 425.1 | 1,974 | 1,208 | 405,779 |
| Cardiac conduction disorders | circulatory system | 426 | 21,083 | 6,959 | 380,919 |
| Atrioventricular [AV] block | circulatory system | 426.2 | 25,917 | 2,125 | 380,919 |
| First degree AV block | circulatory system | 426.21 | 27,054 | 988 | 380,919 |
| Atrioventricular block, complete | circulatory system | 426.24 | 27,455 | 587 | 380,919 |
| Bundle branch block | circulatory system | 426.3 | 24,689 | 3,353 | 380,919 |
| Right bundle branch block | circulatory system | 426.31 | 26,544 | 1,498 | 380,919 |
| Left bundle branch block | circulatory system | 426.32 | 26,285 | 1,757 | 380,919 |
| Cardiac pacemaker/device in situ | circulatory system | 426.9 | 25,555 | 2,487 | 380,919 |
| Cardiac pacemaker in situ | circulatory system | 426.91 | 25,759 | 2,283 | 380,919 |
| Cardiac dysrhythmias | circulatory system | 427 | 3,361 | 24,681 | 380,919 |
| Paroxysmal tachycardia, unspecified | circulatory system | 427.1 | 24,817 | 3,225 | 380,919 |
| Paroxysmal supraventricular tachycardia | circulatory system | 427.11 | 25,683 | 2,359 | 380,919 |
| Paroxysmal ventricular tachycardia | circulatory system | 427.12 | 27,104 | 938 | 380,919 |
| Atrial fibrillation and flutter | circulatory system | 427.2 | 13,222 | 14,820 | 380,919 |
| Other specified cardiac dysrhythmias | circulatory system | 427.3 | 24,806 | 3,236 | 380,919 |
| Cardiac arrest and ventricular fibrillation | circulatory system | 427.4 | 26,905 | 1,137 | 380,919 |
| Cardiac arrest | circulatory system | 427.42 | 27,115 | 927 | 380,919 |
| Arrhythmia (cardiac) NOS | circulatory system | 427.5 | 27,120 | 922 | 380,919 |
| Premature beats | circulatory system | 427.6 | 27,506 | 536 | 380,919 |
| Tachycardia NOS | circulatory system | 427.7 | 25,849 | 2,193 | 380,919 |
| Palpitations | circulatory system | 427.9 | 24,210 | 3,832 | 380,919 |
| Congestive heart failure; nonhypertensive | circulatory system | 428 | 712 | 5,415 | 402,834 |
| Heart failure NOS | circulatory system | 428.2 | 1,858 | 4,269 | 402,834 |
| Ill-defined descriptions and complications of heart disease | circulatory system | 429 | 5,331 | 796 | 402,834 |
| Abnormal function study of cardiovascular system | circulatory system | 429.2 | 5,603 | 524 | 402,834 |
| Intracranial hemorrhage | circulatory system | 430 | 8,148 | 1,796 | 399,017 |
| Subarachnoid hemorrhage | circulatory system | 430.1 | 9,132 | 812 | 399,017 |
| Intracerebral hemorrhage | circulatory system | 430.2 | 9,244 | 700 | 399,017 |
| Cerebrovascular disease | circulatory system | 433 | 1,202 | 8,742 | 399,017 |
| Occlusion and stenosis of precerebral arteries | circulatory system | 433.1 | 8,759 | 1,185 | 399,017 |
| Occlusion of cerebral arteries | circulatory system | 433.2 | 5,810 | 4,134 | 399,017 |
| Cerebral artery occlusion, with cerebral infarction | circulatory system | 433.21 | 8,443 | 1,501 | 399,017 |
| Cerebral ischemia | circulatory system | 433.3 | 7,024 | 2,920 | 399,017 |
| Transient cerebral ischemia | circulatory system | 433.31 | 7,798 | 2,146 | 399,017 |
| Late effects of cerebrovascular disease | circulatory system | 433.8 | 8,681 | 1,263 | 399,017 |
| Atherosclerosis | circulatory system | 440 | 7,042 | 1,324 | 400,595 |
| Atherosclerosis of the extremities | circulatory system | 440.2 | 7,555 | 811 | 400,595 |
| Vascular insufficiency of intestine | circulatory system | 441 | 7,790 | 576 | 400,595 |
| Other aneurysm | circulatory system | 442 | 6,558 | 1,808 | 400,595 |
| Aortic aneurysm | circulatory system | 442.1 | 6,992 | 1,374 | 400,595 |
| Abdominal aortic aneurysm | circulatory system | 442.11 | 7,463 | 903 | 400,595 |
| Peripheral vascular disease | circulatory system | 443 | 4,439 | 3,927 | 400,595 |
| Raynaud's syndrome | circulatory system | 443.1 | 7,218 | 1,148 | 400,595 |
| Peripheral vascular disease, unspecified | circulatory system | 443.9 | 5,800 | 2,566 | 400,595 |
| Arterial embolism and thrombosis | circulatory system | 444 | 7,445 | 921 | 400,595 |
| Arterial embolism and thrombosis of lower extremity artery | circulatory system | 444.1 | 7,809 | 557 | 400,595 |
| Polyarteritis nodosa and allied conditions | circulatory system | 446 | 7,538 | 828 | 400,595 |
| Other disorders of arteries and arterioles | circulatory system | 447 | 7,033 | 1,333 | 400,595 |
| Stricture of artery | circulatory system | 447.1 | 7,493 | 873 | 400,595 |
| Noninfectious disorders of lymphatic channels | circulatory system | 450 | 0 | 705 | 408,256 |
| Phlebitis and thrombophlebitis | circulatory system | 451 | 35,469 | 3,900 | 369,592 |
| Phlebitis and thrombophlebitis of lower extremities | circulatory system | 451.2 | 35,782 | 3,587 | 369,592 |
| Other venous embolism and thrombosis | circulatory system | 452 | 38,811 | 558 | 369,592 |
| Varicose veins | circulatory system | 454 | 27,197 | 12,172 | 369,592 |
| Varicose veins of lower extremity | circulatory system | 454.1 | 27,672 | 11,697 | 369,592 |
| Varicose veins of lower extremity, symptomtic | circulatory system | 454.11 | 38,736 | 633 | 369,592 |
| Hemorrhoids | circulatory system | 455 | 15,473 | 23,896 | 369,592 |
| Hypotension | circulatory system | 458 | 15,229 | 5,827 | 387,905 |
| Orthostatic hypotension | circulatory system | 458.1 | 19,709 | 1,347 | 387,905 |
| Hypotension NOS | circulatory system | 458.9 | 17,538 | 3,518 | 387,905 |
| Other disorders of circulatory system | circulatory system | 459 | 4,512 | 16,544 | 387,905 |
| Circulatory disease NEC | circulatory system | 459.9 | 4,690 | 16,366 | 387,905 |
| Acute upper respiratory infections of multiple or unspecified sites | respiratory | 465 | 179 | 2,335 | 406,447 |
| Acute pharyngitis | respiratory | 465.2 | 1,644 | 870 | 406,447 |
| Septal Deviations/Turbinate Hypertrophy | respiratory | 470 | 13,977 | 4,939 | 390,045 |
| Nasal polyps | respiratory | 471 | 15,605 | 3,311 | 390,045 |
| Chronic pharyngitis and nasopharyngitis | respiratory | 472 | 17,950 | 966 | 390,045 |
| Diseases of the larynx and vocal cords | respiratory | 473 | 16,286 | 2,630 | 390,045 |
| Voice disturbance | respiratory | 473.4 | 17,830 | 1,086 | 390,045 |
| Acute tonsillitis | respiratory | 474.1 | 18,302 | 614 | 390,045 |
| Chronic tonsillitis and adenoiditis | respiratory | 474.2 | 17,790 | 1,126 | 390,045 |
| Chronic sinusitis | respiratory | 475 | 16,314 | 2,602 | 390,045 |
| Allergic rhinitis | respiratory | 476 | 17,856 | 1,060 | 390,045 |
| Epistaxis or throat hemorrhage | respiratory | 477 | 16,460 | 2,456 | 390,045 |
| Other upper respiratory disease | respiratory | 479 | 14,815 | 4,101 | 390,045 |
| Pneumonia | respiratory | 480 | 364 | 10,059 | 398,538 |
| Bacterial pneumonia | respiratory | 480.1 | 3,713 | 6,710 | 398,538 |
| Pneumococcal pneumonia | respiratory | 480.11 | 4,472 | 5,951 | 398,538 |
| Asthma | respiratory | 495 | 7,124 | 26,332 | 375,505 |
| Chronic airway obstruction | respiratory | 496 | 22,954 | 10,502 | 375,505 |
| Emphysema | respiratory | 496.1 | 31,729 | 1,727 | 375,505 |
| Chronic bronchitis | respiratory | 496.2 | 30,522 | 2,934 | 375,505 |
| Obstructive chronic bronchitis | respiratory | 496.21 | 30,758 | 2,698 | 375,505 |
| Bronchitis | respiratory | 497 | 32,825 | 631 | 375,505 |
| Pneumonitis due to inhalation of food or vomitus | respiratory | 501 | 10,964 | 586 | 397,411 |
| Postinflammatory pulmonary fibrosis | respiratory | 502 | 10,663 | 887 | 397,411 |
| Empyema and pneumothorax | respiratory | 506 | 10,376 | 1,174 | 397,411 |
| Pleurisy; pleural effusion | respiratory | 507 | 5,102 | 6,448 | 397,411 |
| Respiratory failure, insufficiency, arrest | respiratory | 509 | 8,985 | 2,565 | 397,411 |
| Respiratory insufficiency | respiratory | 509.2 | 9,801 | 1,749 | 397,411 |
| Other diseases of lung | respiratory | 510 | 0 | 781 | 408,180 |
| Other symptoms of respiratory system | respiratory | 512 | 0 | 9,128 | 399,833 |
| Shortness of breath | respiratory | 512.7 | 3,244 | 5,884 | 399,833 |
| Cough | respiratory | 512.8 | 6,244 | 2,884 | 399,833 |
| Other dyspnea | respiratory | 512.9 | 8,216 | 912 | 399,833 |
| Respiratory abnormalities | respiratory | 513 | 0 | 611 | 408,350 |
| Abnormal findings examination of lungs | respiratory | 514 | 0 | 2,500 | 406,461 |
| Abnormal sputum | respiratory | 516 | 0 | 2,167 | 406,794 |
| Other diseases of respiratory system, not elsewhere classified | respiratory | 519 | 0 | 9,436 | 399,525 |
| Other diseases of respiratory system, NEC | respiratory | 519.8 | 592 | 8,844 | 399,525 |
| Disorders of tooth development | digestive | 520 | 8,376 | 2,449 | 398,136 |
| Disturbances in tooth eruption | digestive | 520.2 | 8,461 | 2,364 | 398,136 |
| Diseases of hard tissues of teeth | digestive | 521 | 7,734 | 3,091 | 398,136 |
| Dental caries | digestive | 521.1 | 7,774 | 3,051 | 398,136 |
| Diseases of pulp and periapical tissues | digestive | 522 | 9,030 | 1,795 | 398,136 |
| Periapical abscess | digestive | 522.5 | 9,648 | 1,177 | 398,136 |
| Gingival and periodontal diseases | digestive | 523 | 9,083 | 1,742 | 398,136 |
| Periodontitis (acute or chronic) | digestive | 523.3 | 9,603 | 1,222 | 398,136 |
| Acute periodontitis | digestive | 523.31 | 10,157 | 668 | 398,136 |
| Chronic periodontitis | digestive | 523.32 | 10,264 | 561 | 398,136 |
| Other diseases of the teeth and supporting structures | digestive | 525 | 8,136 | 2,689 | 398,136 |
| Diseases of the jaws | digestive | 526 | 9,865 | 960 | 398,136 |
| Diseases of the salivary glands | digestive | 527 | 4,928 | 710 | 403,323 |
| Diseases of the oral soft tissues, excluding lesions specific for gingiva and tongue | digestive | 528 | 1,699 | 3,939 | 403,323 |
| Diseases of lips | digestive | 528.5 | 4,954 | 684 | 403,323 |
| Diseases and other conditions of the tongue | digestive | 529 | 4,402 | 1,236 | 403,323 |
| Diseases of esophagus | digestive | 530 | 3,834 | 35,852 | 369,275 |
| Esophagitis, GERD and related diseases | digestive | 530.1 | 7,578 | 32,108 | 369,275 |
| GERD | digestive | 530.11 | 25,463 | 14,223 | 369,275 |
| Ulcer of esophagus | digestive | 530.12 | 34,443 | 5,243 | 369,275 |
| Reflux esophagitis | digestive | 530.14 | 29,135 | 10,551 | 369,275 |
| Esophageal bleeding (varices/hemorrhage) | digestive | 530.2 | 38,014 | 1,672 | 369,275 |
| Disorders of esophageal motility | digestive | 530.5 | 39,047 | 639 | 369,275 |
| Heartburn | digestive | 530.9 | 37,586 | 2,100 | 369,275 |
| Peptic ulcer (excl. esophageal) | digestive | 531 | 0 | 7,436 | 401,525 |
| Hemorrhage from gastrointestinal ulcer | digestive | 531.1 | 6,819 | 617 | 401,525 |
| Gastric ulcer | digestive | 531.2 | 3,327 | 4,109 | 401,525 |
| Duodenal ulcer | digestive | 531.3 | 4,434 | 3,002 | 401,525 |
| Dysphagia | digestive | 532 | 33,204 | 6,482 | 369,275 |
| Gastritis and duodenitis | digestive | 535 | 1,896 | 28,941 | 378,124 |
| Acute gastritis | digestive | 535.1 | 29,653 | 1,184 | 378,124 |
| Duodenitis | digestive | 535.6 | 23,182 | 7,655 | 378,124 |
| Other specified gastritis | digestive | 535.8 | 22,690 | 8,147 | 378,124 |
| Other disorders of stomach and duodenum | digestive | 537 | 27,433 | 3,404 | 378,124 |
| Appendiceal conditions | digestive | 540 | 0 | 3,409 | 405,552 |
| Appendicitis | digestive | 540.1 | 192 | 3,217 | 405,552 |
| Acute appendicitis | digestive | 540.11 | 801 | 2,608 | 405,552 |
| Abdominal hernia | digestive | 550 | 0 | 47,344 | 361,617 |
| Inguinal hernia | digestive | 550.1 | 31,349 | 15,995 | 361,617 |
| Diaphragmatic hernia | digestive | 550.2 | 20,218 | 27,126 | 361,617 |
| Femoral hernia | digestive | 550.3 | 46,693 | 651 | 361,617 |
| Umbilical hernia | digestive | 550.4 | 43,617 | 3,727 | 361,617 |
| Ventral hernia | digestive | 550.5 | 43,896 | 3,448 | 361,617 |
| Inflammatory bowel disease and other gastroenteritis and colitis | digestive | 555 | 69,650 | 4,528 | 334,783 |
| Regional enteritis | digestive | 555.1 | 72,435 | 1,743 | 334,783 |
| Ulcerative colitis | digestive | 555.2 | 70,983 | 3,195 | 334,783 |
| Ulcerative colitis (chronic) | digestive | 555.21 | 73,639 | 539 | 334,783 |
| Ulceration of the lower GI tract | digestive | 556 | 73,115 | 1,063 | 334,783 |
| Ulceration of intestine | digestive | 556.1 | 73,504 | 674 | 334,783 |
| Intestinal malabsorption (non-celiac) | digestive | 557 | 72,075 | 2,103 | 334,783 |
| Noninfectious gastroenteritis | digestive | 558 | 58,431 | 15,747 | 334,783 |
| Ileostomy status | digestive | 559 | 72,518 | 1,660 | 334,783 |
| Intestinal obstruction without mention of hernia | digestive | 560 | 70,184 | 3,994 | 334,783 |
| Paralytic ileus | digestive | 560.1 | 73,656 | 522 | 334,783 |
| Peritoneal or intestinal adhesions | digestive | 560.3 | 73,346 | 832 | 334,783 |
| Other intestinal obstruction | digestive | 560.4 | 70,832 | 3,346 | 334,783 |
| Symptoms involving digestive system | digestive | 561 | 58,201 | 15,977 | 334,783 |
| Flatulence | digestive | 561.2 | 72,489 | 1,689 | 334,783 |
| Diverticulosis and diverticulitis | digestive | 562 | 46,867 | 27,311 | 334,783 |
| Diverticulosis | digestive | 562.1 | 46,910 | 27,268 | 334,783 |
| Constipation | digestive | 563 | 63,736 | 10,442 | 334,783 |
| Functional digestive disorders | digestive | 564 | 52,040 | 22,138 | 334,783 |
| Irritable Bowel Syndrome | digestive | 564.1 | 68,630 | 5,548 | 334,783 |
| Abnormal findings on exam of gastrointestinal tract/ abdominal area | digestive | 564.8 | 72,528 | 1,650 | 334,783 |
| Personal history of diseases of digestive system | digestive | 564.9 | 58,786 | 15,392 | 334,783 |
| Anal and rectal conditions | digestive | 565 | 6,626 | 14,997 | 387,338 |
| Anal and rectal polyp | digestive | 565.1 | 14,215 | 7,408 | 387,338 |
| Peritonitis and retroperitoneal infections | digestive | 567 | 20,736 | 887 | 387,338 |
| Other disorders of peritoneum | digestive | 568 | 18,315 | 3,308 | 387,338 |
| Peritoneal adhesions (postoperative) (postinfection) | digestive | 568.1 | 18,515 | 3,108 | 387,338 |
| Other disorders of intestine | digestive | 569 | 17,423 | 4,200 | 387,338 |
| Chronic liver disease and cirrhosis | digestive | 571 | 6,011 | 2,895 | 400,055 |
| Other chronic nonalcoholic liver disease | digestive | 571.5 | 7,242 | 1,664 | 400,055 |
| Liver abscess and sequelae of chronic liver disease | digestive | 571.8 | 7,964 | 942 | 400,055 |
| Portal hypertension | digestive | 571.81 | 8,377 | 529 | 400,055 |
| Ascites (non malignant) | digestive | 572 | 7,359 | 1,547 | 400,055 |
| Other disorders of liver | digestive | 573 | 3,059 | 5,847 | 400,055 |
| Jaundice (not of newborn) | digestive | 573.5 | 8,009 | 897 | 400,055 |
| Abnormal results of function study of liver | digestive | 573.7 | 5,427 | 3,479 | 400,055 |
| Cholelithiasis and cholecystitis | digestive | 574 | 1,429 | 16,225 | 391,307 |
| Cholelithiasis | digestive | 574.1 | 3,877 | 13,777 | 391,307 |
| Cholelithiasis with acute cholecystitis | digestive | 574.11 | 16,141 | 1,513 | 391,307 |
| Cholelithiasis with other cholecystitis | digestive | 574.12 | 12,182 | 5,472 | 391,307 |
| Calculus of bile duct | digestive | 574.2 | 15,020 | 2,634 | 391,307 |
| Cholecystitis without cholelithiasis | digestive | 574.3 | 14,893 | 2,761 | 391,307 |
| Other biliary tract disease | digestive | 575 | 13,762 | 3,892 | 391,307 |
| Obstruction of bile duct | digestive | 575.2 | 16,877 | 777 | 391,307 |
| Other disorders of gallbladder | digestive | 575.7 | 16,242 | 1,412 | 391,307 |
| Other disorders of biliary tract | digestive | 575.8 | 16,614 | 1,040 | 391,307 |
| Diseases of pancreas | digestive | 577 | 0 | 2,690 | 406,271 |
| Chronic pancreatitis | digestive | 577.2 | 2,176 | 514 | 406,271 |
| Gastrointestinal hemorrhage | digestive | 578 | 2,667 | 21,137 | 385,157 |
| Blood in stool | digestive | 578.2 | 21,165 | 2,639 | 385,157 |
| Hemorrhage of rectum and anus | digestive | 578.8 | 10,582 | 13,222 | 385,157 |
| Hemorrhage of gastrointestinal tract | digestive | 578.9 | 18,575 | 5,229 | 385,157 |
| Other symptoms involving abdomen and pelvis | digestive | 579 | 20,609 | 3,195 | 385,157 |
| Nonspecific abnormal findings in stool contents | digestive | 579.8 | 22,145 | 1,659 | 385,157 |
| Nephritis; nephrosis; renal sclerosis | genitourinary | 580 | 9,837 | 1,522 | 397,602 |
| Glomerulonephritis | genitourinary | 580.1 | 10,326 | 1,033 | 397,602 |
| Chronic glomerulonephritis, NOS | genitourinary | 580.14 | 10,514 | 845 | 397,602 |
| Renal failure | genitourinary | 585 | 4,374 | 6,985 | 397,602 |
| Acute renal failure | genitourinary | 585.1 | 6,838 | 4,521 | 397,602 |
| Renal failure NOS | genitourinary | 585.2 | 9,947 | 1,412 | 397,602 |
| Chronic renal failure [CKD] | genitourinary | 585.3 | 8,730 | 2,629 | 397,602 |
| Other disorders of the kidney and ureters | genitourinary | 586 | 7,997 | 3,362 | 397,602 |
| Cyst of kidney, acquired | genitourinary | 586.2 | 10,099 | 1,260 | 397,602 |
| Stricture/obstruction of ureter | genitourinary | 586.4 | 10,446 | 913 | 397,602 |
| Pyelonephritis | genitourinary | 590 | 27,701 | 1,324 | 379,936 |
| Urinary tract infection | genitourinary | 591 | 16,534 | 12,491 | 379,936 |
| Cystitis and urethritis | genitourinary | 592 | 25,937 | 3,088 | 379,936 |
| Cystitis | genitourinary | 592.1 | 26,077 | 2,948 | 379,936 |
| Chronic cystitis | genitourinary | 592.12 | 28,133 | 892 | 379,936 |
| Hematuria | genitourinary | 593 | 12,616 | 16,409 | 379,936 |
| Urinary calculus | genitourinary | 594 | 1,313 | 6,643 | 401,005 |
| Calculus of kidney | genitourinary | 594.1 | 4,765 | 3,191 | 401,005 |
| Calculus of lower urinary tract | genitourinary | 594.2 | 7,178 | 778 | 401,005 |
| Calculus of ureter | genitourinary | 594.3 | 5,539 | 2,417 | 401,005 |
| Other disorders of bladder | genitourinary | 596 | 4,329 | 9,933 | 394,699 |
| Functional disorders of bladder | genitourinary | 596.5 | 12,598 | 1,664 | 394,699 |
| Other disorders of urethra and urinary tract | genitourinary | 597 | 10,193 | 4,069 | 394,699 |
| Urethral stricture (not specified as infectious) | genitourinary | 597.1 | 10,931 | 3,331 | 394,699 |
| Abnormal findings on examination of urine | genitourinary | 598 | 0 | 3,547 | 405,414 |
| Other symptoms/disorders or the urinary system | genitourinary | 599 | 0 | 24,031 | 384,930 |
| Retention of urine | genitourinary | 599.2 | 17,276 | 6,755 | 384,930 |
| Dysuria | genitourinary | 599.3 | 22,832 | 1,199 | 384,930 |
| Urinary incontinence | genitourinary | 599.4 | 15,175 | 8,856 | 384,930 |
| Frequency of urination and polyuria | genitourinary | 599.5 | 19,994 | 4,037 | 384,930 |
| Abnormal findings on mammogram or breast exam | genitourinary | 611 | 5,635 | 1,580 | 401,746 |
| Lump or mass in breast | genitourinary | 611.3 | 5,690 | 1,525 | 401,746 |
| Breast conditions, congenital or relating to hormones | genitourinary | 612 | 6,273 | 942 | 401,746 |
| Hypertrophy of breast (Gynecomastia) | genitourinary | 612.2 | 6,361 | 854 | 401,746 |
| Inflammatory disease of breast | genitourinary | 613.1 | 1,260 | 723 | 406,978 |
| Other signs and symptoms in breast | genitourinary | 613.7 | 1,279 | 704 | 406,978 |
| Noninflammatory disorders of ovary, fallopian tube, and broad ligament | genitourinary | 619.1 | 8,602 | 730 | 399,629 |
| Superficial cellulitis and abscess | dermatologic | 681 | 3,875 | 7,451 | 397,635 |
| Cellulitis and abscess of fingers/toes | dermatologic | 681.1 | 10,760 | 566 | 397,635 |
| Cellulitis and abscess of face/neck | dermatologic | 681.2 | 10,802 | 524 | 397,635 |
| Cellulitis and abscess of arm/hand | dermatologic | 681.3 | 5,787 | 5,539 | 397,635 |
| Cellulitis and abscess of leg, except foot | dermatologic | 681.5 | 5,779 | 5,547 | 397,635 |
| Cellulitis and abscess of foot, toe | dermatologic | 681.6 | 5,824 | 5,502 | 397,635 |
| Cellulitis and abscess of trunk | dermatologic | 681.7 | 10,724 | 602 | 397,635 |
| Other local infections of skin and subcutaneous tissue | dermatologic | 686 | 6,806 | 4,520 | 397,635 |
| Carbuncle and furuncle | dermatologic | 686.1 | 9,024 | 2,302 | 397,635 |
| Pilonidal cyst | dermatologic | 686.3 | 10,739 | 587 | 397,635 |
| Symptoms affecting skin | dermatologic | 687 | 0 | 5,554 | 403,407 |
| Rash and other nonspecific skin eruption | dermatologic | 687.1 | 3,397 | 2,157 | 403,407 |
| Disturbance of skin sensation | dermatologic | 687.4 | 2,654 | 2,900 | 403,407 |
| Disorder of skin and subcutaneous tissue NOS | dermatologic | 689 | 0 | 5,782 | 403,179 |
| Dyschromia and Vitiligo | dermatologic | 694 | 5,302 | 987 | 402,672 |
| Other dyschromia | dermatologic | 694.2 | 5,506 | 783 | 402,672 |
| Erythematous conditions | dermatologic | 695 | 3,869 | 2,420 | 402,672 |
| Prurigo and Lichen | dermatologic | 695.7 | 5,510 | 779 | 402,672 |
| Psoriasis and related disorders | dermatologic | 696 | 8,469 | 2,293 | 398,199 |
| Psoriasis | dermatologic | 696.4 | 8,525 | 2,237 | 398,199 |
| Psoriasis vulgaris | dermatologic | 696.41 | 9,078 | 1,684 | 398,199 |
| Psoriatic arthropathy | dermatologic | 696.42 | 10,054 | 708 | 398,199 |
| Sarcoidosis | dermatologic | 697 | 5,741 | 548 | 402,672 |
| Pruritus and related conditions | dermatologic | 698 | 0 | 783 | 408,178 |
| Other hypertrophic and atrophic conditions of skin | dermatologic | 701 | 282 | 4,804 | 403,875 |
| Scar conditions and fibrosis of skin | dermatologic | 701.2 | 2,730 | 2,356 | 403,875 |
| Degenerative skin conditions and other dermatoses | dermatologic | 702 | 4,693 | 5,522 | 398,746 |
| Actinic keratosis | dermatologic | 702.1 | 2,928 | 2,594 | 403,439 |
| Seborrheic keratosis | dermatologic | 702.2 | 2,430 | 3,092 | 403,439 |
| Diseases of nail, NOS | dermatologic | 703 | 5,317 | 1,287 | 402,357 |
| Ingrowing nail | dermatologic | 703.1 | 5,634 | 970 | 402,357 |
| Diseases of hair and hair follicles | dermatologic | 704 | 1,260 | 5,344 | 402,357 |
| Disorders of sweat glands | dermatologic | 705 | 8,889 | 817 | 399,255 |
| Hyperhidrosis | dermatologic | 705.8 | 9,114 | 592 | 399,255 |
| Diseases of sebaceous glands | dermatologic | 706 | 758 | 8,948 | 399,255 |
| Sebaceous cyst | dermatologic | 706.2 | 830 | 8,876 | 399,255 |
| Decubitus ulcer | dermatologic | 707.1 | 1,075 | 913 | 406,973 |
| Diffuse diseases of connective tissue | dermatologic | 709 | 6,094 | 3,463 | 399,404 |
| Sicca syndrome | dermatologic | 709.2 | 9,044 | 513 | 399,404 |
| Unspecified diffuse connective tissue disease | dermatologic | 709.7 | 6,837 | 2,720 | 399,404 |
| Osteomyelitis, periostitis, and other infections involving bone | musculoskeletal | 710 | 42,530 | 612 | 365,819 |
| Osteomyelitis | musculoskeletal | 710.1 | 42,577 | 565 | 365,819 |
| Rheumatoid arthritis and other inflammatory polyarthropathies | musculoskeletal | 714 | 38,997 | 4,879 | 365,085 |
| Rheumatoid arthritis | musculoskeletal | 714.1 | 39,464 | 4,412 | 365,085 |
| Other inflammatory spondylopathies | musculoskeletal | 715 | 42,205 | 1,671 | 365,085 |
| Ankylosing spondylitis | musculoskeletal | 715.2 | 43,256 | 620 | 365,085 |
| Other arthropathies | musculoskeletal | 716 | 4,427 | 38,715 | 365,819 |
| Unspecified polyarthropathy or polyarthritis | musculoskeletal | 716.1 | 39,607 | 3,535 | 365,819 |
| Unspecified monoarthritis | musculoskeletal | 716.2 | 27,352 | 15,790 | 365,819 |
| Arthropathy NOS | musculoskeletal | 716.9 | 6,099 | 37,043 | 365,819 |
| Polymyalgia Rheumatica | musculoskeletal | 717 | 0 | 1,152 | 407,809 |
| Spinal stenosis | musculoskeletal | 720 | 13,311 | 3,733 | 391,917 |
| Spondylosis and allied disorders | musculoskeletal | 721 | 9,114 | 7,930 | 391,917 |
| Spondylosis without myelopathy | musculoskeletal | 721.1 | 11,967 | 5,077 | 391,917 |
| Intervertebral disc disorders | musculoskeletal | 722 | 7,803 | 9,241 | 391,917 |
| Displacement of intervertebral disc | musculoskeletal | 722.1 | 16,531 | 513 | 391,917 |
| Degeneration of intervertebral disc | musculoskeletal | 722.6 | 14,198 | 2,846 | 391,917 |
| Other and unspecified disc disorder | musculoskeletal | 722.9 | 12,610 | 4,434 | 391,917 |
| Other unspecified back disorders | musculoskeletal | 724.9 | 15,427 | 1,617 | 391,917 |
| Peripheral enthesopathies and allied syndromes | musculoskeletal | 726 | 15,267 | 14,983 | 378,711 |
| Enthesopathy | musculoskeletal | 726.1 | 20,582 | 9,668 | 378,711 |
| Bursitis | musculoskeletal | 726.3 | 29,392 | 858 | 378,711 |
| Other disorders of synovium, tendon, and bursa | musculoskeletal | 727 | 22,621 | 7,629 | 378,711 |
| Synovitis and tenosynovitis | musculoskeletal | 727.1 | 27,496 | 2,754 | 378,711 |
| Ganglion and cyst of synovium, tendon, and bursa | musculoskeletal | 727.4 | 27,065 | 3,185 | 378,711 |
| Rupture of synovium | musculoskeletal | 727.5 | 29,657 | 593 | 378,711 |
| Disorders of muscle, ligament, and fascia | musculoskeletal | 728 | 25,762 | 4,488 | 378,711 |
| Fasciitis | musculoskeletal | 728.7 | 26,407 | 3,843 | 378,711 |
| Contracture of palmar fascia [Dupuytren's disease] | musculoskeletal | 728.71 | 26,747 | 3,503 | 378,711 |
| Other disorders of soft tissues | musculoskeletal | 729 | 24,080 | 6,170 | 378,711 |
| Rheumatism, unspecified and fibrositis | musculoskeletal | 729.1 | 29,240 | 1,010 | 378,711 |
| Other disorders of bone and cartilage | musculoskeletal | 733 | 14,147 | 3,773 | 391,041 |
| Aseptic necrosis of bone | musculoskeletal | 733.4 | 17,378 | 542 | 391,041 |
| Malunion and nonunion of fracture | musculoskeletal | 733.8 | 16,652 | 1,268 | 391,041 |
| Acquired foot deformities | musculoskeletal | 735 | 4,182 | 9,865 | 394,914 |
| Acquired toe deformities | musculoskeletal | 735.2 | 8,903 | 5,144 | 394,914 |
| Hallux rigidus | musculoskeletal | 735.23 | 12,486 | 1,561 | 394,914 |
| Hallux valgus (Bunion) | musculoskeletal | 735.3 | 7,348 | 6,699 | 394,914 |
| Other acquired deformities of limbs | musculoskeletal | 736 | 12,398 | 1,649 | 394,914 |
| Curvature of spine | musculoskeletal | 737 | 12,913 | 1,134 | 394,914 |
| Kyphoscoliosis and scoliosis | musculoskeletal | 737.3 | 12,984 | 1,063 | 394,914 |
| Acquired spondylolisthesis | musculoskeletal | 738.4 | 12,526 | 1,521 | 394,914 |
| Osteoarthrosis | musculoskeletal | 740 | 0 | 28,439 | 380,522 |
| Osteoarthritis; localized | musculoskeletal | 740.1 | 10,748 | 17,691 | 380,522 |
| Osteoarthrosis, localized, primary | musculoskeletal | 740.11 | 19,370 | 9,069 | 380,522 |
| Osteoarthrosis, generalized | musculoskeletal | 740.2 | 27,840 | 599 | 380,522 |
| Osteoarthrosis NOS | musculoskeletal | 740.9 | 16,003 | 12,436 | 380,522 |
| Symptoms and disorders of the joints | musculoskeletal | 741 | 2,694 | 3,634 | 402,633 |
| Stiffness of joint | musculoskeletal | 741.2 | 5,713 | 615 | 402,633 |
| Joint effusions | musculoskeletal | 741.4 | 4,903 | 1,425 | 402,633 |
| Derangement of joint, nontraumatic | musculoskeletal | 742 | 3,415 | 2,913 | 402,633 |
| Articular cartilage disorder | musculoskeletal | 742.8 | 5,828 | 500 | 402,633 |
| Osteoporosis, osteopenia and pathological fracture | musculoskeletal | 743 | 0 | 7,682 | 401,279 |
| Osteoporosis | musculoskeletal | 743.1 | 1,198 | 6,484 | 401,279 |
| Osteoporosis NOS | musculoskeletal | 743.11 | 2,060 | 5,622 | 401,279 |
| Other specified osteoporosis | musculoskeletal | 743.13 | 7,150 | 532 | 401,279 |
| Pathologic fracture | musculoskeletal | 743.2 | 7,168 | 514 | 401,279 |
| Osteopenia or other disorder of bone and cartilage | musculoskeletal | 743.9 | 6,862 | 820 | 401,279 |
| Pain in joint | musculoskeletal | 745 | 0 | 8,037 | 400,924 |
| Cardiac and circulatory congenital anomalies | congenital anomalies | 747 | 0 | 2,796 | 406,165 |
| Cardiac congenital anomalies | congenital anomalies | 747.1 | 178 | 2,618 | 406,165 |
| Cardiac shunt/ heart septal defect | congenital anomalies | 747.11 | 2,210 | 586 | 406,165 |
| Congenital anomalies of great vessels | congenital anomalies | 747.13 | 997 | 1,799 | 406,165 |
| Digestive congenital anomalies | congenital anomalies | 750 | 1,528 | 703 | 406,730 |
| Genitourinary congenital anomalies | congenital anomalies | 751 | 635 | 1,596 | 406,730 |
| Congenital anomalies of genital organs | congenital anomalies | 751.1 | 1,499 | 732 | 406,730 |
| Congenital anomalies of urinary system | congenital anomalies | 751.2 | 1,376 | 855 | 406,730 |
| Other congenital musculoskeletal anomalies | congenital anomalies | 756 | 536 | 594 | 407,831 |
| Back pain | symptoms | 760 | 0 | 11,274 | 397,687 |
| Cervicalgia | symptoms | 761 | 0 | 1,796 | 407,165 |
| Sciatica | symptoms | 764 | 1,097 | 2,383 | 405,481 |
| Neuralgia, neuritis, and radiculitis NOS | symptoms | 766 | 2,299 | 1,181 | 405,481 |
| Myalgia and myositis unspecified | symptoms | 770 | 0 | 642 | 408,319 |
| Musculoskeletal symptoms referable to limbs | symptoms | 771 | 0 | 5,493 | 403,468 |
| Swelling of limb | symptoms | 771.1 | 830 | 4,663 | 403,468 |
| Symptoms of the muscles | symptoms | 772 | 0 | 698 | 408,263 |
| Pain in limb | symptoms | 773 | 0 | 6,327 | 402,634 |
| Symptoms involving skin and other integumentary tissue | symptoms | 782 | 0 | 1,816 | 407,145 |
| Edema | symptoms | 782.3 | 168 | 1,648 | 407,145 |
| Fever of unknown origin | symptoms | 783 | 0 | 3,940 | 405,021 |
| Abdominal pain | symptoms | 785 | 0 | 41,316 | 367,645 |
| Syncope and collapse | symptoms | 788 | 0 | 9,163 | 399,798 |
| Nausea and vomiting | symptoms | 789 | 0 | 11,706 | 397,255 |
| Nonspecific findings on examination of blood | symptoms | 790 | 0 | 6,619 | 402,342 |
| Other abnormal blood chemistry | symptoms | 790.6 | 206 | 6,413 | 402,342 |
| Gangrene | symptoms | 791 | 0 | 550 | 408,411 |
| Nonspecific abnormal findings on radiological and other examination of musculoskeletal system | injuries & poisonings | 793 | 0 | 698 | 408,263 |
| Nonspecific abnormal findings on radiological and other examination of other intrathoracic organs (echocardiogram, etc) | circulatory system | 793.2 | 131 | 567 | 408,263 |
| Malaise and fatigue | symptoms | 798 | 0 | 3,429 | 405,532 |
| Chronic fatigue syndrome | symptoms | 798.1 | 2,836 | 593 | 405,532 |
| Fracture of lower limb | injuries & poisonings | 800 | 13,945 | 7,251 | 387,765 |
| Fracture of neck of femur | injuries & poisonings | 800.1 | 19,382 | 1,814 | 387,765 |
| Fracture of tibia and fibula | injuries & poisonings | 800.3 | 19,034 | 2,162 | 387,765 |
| Fracture of patella | injuries & poisonings | 800.4 | 20,428 | 768 | 387,765 |
| Fracture of ankle and foot | injuries & poisonings | 801 | 18,857 | 2,339 | 387,765 |
| Fracture of pelvis | injuries & poisonings | 802 | 20,474 | 722 | 387,765 |
| Fracture of upper limb | injuries & poisonings | 803 | 12,675 | 8,521 | 387,765 |
| Fracture of humerus | injuries & poisonings | 803.1 | 19,504 | 1,692 | 387,765 |
| Fracture of radius and ulna | injuries & poisonings | 803.2 | 15,950 | 5,246 | 387,765 |
| Fracture of clavicle or scapula | injuries & poisonings | 803.3 | 19,675 | 1,521 | 387,765 |
| Fracture of hand or wrist | injuries & poisonings | 804 | 17,839 | 3,357 | 387,765 |
| Fracture of vertebral column without mention of spinal cord injury | injuries & poisonings | 805 | 19,844 | 1,352 | 387,765 |
| Fracture of ribs | injuries & poisonings | 807 | 20,421 | 775 | 387,765 |
| Fracture of unspecified bones | injuries & poisonings | 809 | 19,892 | 1,304 | 387,765 |
| Intracranial hemorrhage (injury) | injuries & poisonings | 818 | 2,899 | 508 | 405,554 |
| Skull and face fracture and other intercranial injury | injuries & poisonings | 819 | 450 | 2,957 | 405,554 |
| Torus fracture | injuries & poisonings | 823 | 22,130 | 1,674 | 385,157 |
| Internal derangement of knee | injuries & poisonings | 835 | 2,074 | 15,430 | 391,457 |
| Traumatic arthropathy | injuries & poisonings | 836 | 16,976 | 528 | 391,457 |
| Hemorrhage or hematoma complicating a procedure | injuries & poisonings | 850 | 8,703 | 5,329 | 394,929 |
| Complication of colostomy or enterostomy | injuries & poisonings | 853 | 13,530 | 502 | 394,929 |
| Complications of cardiac/vascular device, implant, and graft | injuries & poisonings | 854 | 12,243 | 1,789 | 394,929 |
| Mechanical complication of unspecified genitourinary device, implant, and graft | injuries & poisonings | 857 | 12,791 | 1,241 | 394,929 |
| Complication of internal orthopedic device | injuries & poisonings | 858 | 10,865 | 3,167 | 394,929 |
| Complication due to other implant and internal device | injuries & poisonings | 859 | 10,767 | 3,265 | 394,929 |
| Open wounds of head; neck; and trunk | injuries & poisonings | 870 | 4,698 | 3,837 | 400,426 |
| Other open wound of head and face | injuries & poisonings | 870.3 | 5,616 | 2,919 | 400,426 |
| Open wounds of extremities | injuries & poisonings | 871 | 4,316 | 4,219 | 400,426 |
| Injuries to the nervous system | injuries & poisonings | 907 | 150 | 1,315 | 407,496 |
| Superficial injury without mention of infection | injuries & poisonings | 915 | 1,077 | 4,289 | 403,595 |
| Contusion | injuries & poisonings | 916 | 0 | 1,486 | 407,475 |
| Atopic/contact dermatitis due to other or unspecified | dermatologic | 939 | 2,034 | 2,110 | 404,817 |
| Anaphylactic shock NOS | injuries & poisonings | 946 | 3,596 | 548 | 404,817 |
| Poisoning by antibiotics | injuries & poisonings | 960 | 8,734 | 18,430 | 381,797 |
| Allergy/adverse effect of penicillin | injuries & poisonings | 960.2 | 11,074 | 16,090 | 381,797 |
| Poisoning by other antiinfectives | injuries & poisonings | 961 | 25,908 | 1,256 | 381,797 |
| Poisoning/allergy of sulfonamides | injuries & poisonings | 961.1 | 26,290 | 874 | 381,797 |
| Poisoning by analgesics, antipyretics, and antirheumatics | injuries & poisonings | 965 | 20,254 | 6,910 | 381,797 |
| Opiates and related narcotics causing adverse effects in therapeutic use | injuries & poisonings | 965.1 | 26,014 | 1,150 | 381,797 |
| Poisoning by anticonvulsants and anti-Parkinsonism drugs | injuries & poisonings | 966 | 26,655 | 509 | 381,797 |
| Adverse effects of sedatives or other central nervous system depressants and anesthetics | injuries & poisonings | 967 | 26,576 | 588 | 381,797 |
| Adverse drug events and drug allergies | injuries & poisonings | 979 | 26,469 | 695 | 381,797 |
| Effects radiation NOS | injuries & poisonings | 990 | 2,536 | 3,130 | 403,295 |
| Sepsis and SIRS | infectious diseases | 994 | 0 | 2,811 | 406,150 |
| Sepsis | infectious diseases | 994.2 | 0 | 2,811 | 406,150 |
| Burns | injuries & poisonings | 1000 | 0 | 579 | 408,382 |
| Foreign body injury | injuries & poisonings | 1001 | 0 | 1,210 | 407,751 |
| Symptoms concerning nutrition, metabolism, and development | symptoms | 1002 | 0 | 5,512 | 403,449 |
| Other symptoms | symptoms | 1005 | 0 | 1,222 | 407,739 |
| Crushing or internal injury to organs | injuries & poisonings | 1008 | 0 | 1,007 | 407,954 |
| Injury, NOS | injuries & poisonings | 1009 | 0 | 8,440 | 400,521 |
| Other tests | symptoms | 1010 | 0 | 5,972 | 402,989 |
| Complications of surgical and medical procedures | injuries & poisonings | 1011 | 0 | 9,140 | 399,821 |
| Effects of other external causes | symptoms | 1015 | 0 | 4,230 | 404,731 |
| Other ill-defined and unknown causes of morbidity and mortality | symptoms | 1019 | 0 | 16,398 | 392,563 |

Abbreviation: Phe-MR, phenome-wide Mendelian randomization.

**Table S5. Inverse-variance weighted MR analysis for the associations between blood metabolites and delirium.**

| **Metabolites** | **Super-pathway** | **SNPs** | **Q estimate** | ***P* value for Q estimate** | **IVW model** | **OR** | **95% CI** | ***P* value** |
| --- | --- | --- | --- | --- | --- | --- | --- | --- |
| 2-hydroxyisobutyrate | Amino acid | 4 | 2.14 | 0.54 | Fixed-effect model | 0.45 | 0.13 - 1.64 | 0.23 |
| 3-methyl-2-oxovalerate | Amino acid | 3 | 1.04 | 0.60 | Fixed-effect model | 1.48 | 0.11 - 19.03 | 0.77 |
| 4-acetamidobutanoate | Amino acid | 6 | 1.38 | 0.93 | Fixed-effect model | 0.90 | 0.25 - 3.25 | 0.87 |
| alpha-hydroxyisovalerate | Amino acid | 3 | 0.06 | 0.97 | Fixed-effect model | 0.67 | 0.25 - 1.82 | 0.43 |
| asparagine | Amino acid | 3 | 0.30 | 0.86 | Fixed-effect model | 0.61 | 0.11 - 3.39 | 0.57 |
| betaine | Amino acid | 5 | 0.56 | 0.97 | Fixed-effect model | 0.59 | 0.17 - 2.08 | 0.41 |
| citrulline | Amino acid | 3 | 5.42 | 0.07 | Fixed-effect model | 0.67 | 0.03 - 15.84 | 0.81 |
| glutaroyl carnitine | Amino acid | 9 | 3.80 | 0.87 | Fixed-effect model | 1.16 | 0.56 - 2.41 | 0.69 |
| glycine | Amino acid | 5 | 5.70 | 0.22 | Fixed-effect model | 1.13 | 0.61 - 2.11 | 0.69 |
| isobutyrylcarnitine | Amino acid | 6 | 1.37 | 0.93 | Fixed-effect model | 1.37 | 0.70 - 2.69 | 0.36 |
| isovalerylcarnitine | Amino acid | 7 | 7.78 | 0.25 | Fixed-effect model | 1.04 | 0.48 - 2.29 | 0.91 |
| kynurenine | Amino acid | 5 | 2.80 | 0.59 | Fixed-effect model | 0.86 | 0.19 - 3.89 | 0.85 |
| leucine | Amino acid | 11 | 23.81 | 0.01 | Random effect model | 1.29 | 0.02 - 101.98 | 0.91 |
| N-acetylglycine | Amino acid | 6 | 8.33 | 0.14 | Fixed-effect model | 1.26 | 0.78 - 2.05 | 0.35 |
| N-acetylornithine | Amino acid | 9 | 5.56 | 0.70 | Fixed-effect model | 0.75 | 0.59 - 0.95 | 0.02 |
| proline | Amino acid | 4 | 1.76 | 0.62 | Fixed-effect model | 2.22 | 0.64 - 7.64 | 0.21 |
| pyroglutamine | Amino acid | 4 | 0.84 | 0.84 | Fixed-effect model | 0.99 | 0.44 - 2.20 | 0.98 |
| serine | Amino acid | 3 | 0.54 | 0.76 | Fixed-effect model | 1.80 | 0.35 - 9.32 | 0.48 |
| tryptophan | Amino acid | 17 | 12.28 | 0.72 | Fixed-effect model | 1.18 | 0.11 - 13.03 | 0.89 |
| tryptophan betaine | Amino acid | 3 | 1.34 | 0.51 | Fixed-effect model | 1.52 | 1.01 - 2.29 | 0.05 |
| tyrosine | Amino acid | 4 | 1.49 | 0.68 | Fixed-effect model | 0.14 | 0.01 - 2.03 | 0.15 |
| X-03056--N-[3-(2-Oxopyrrolidin-1-yl)propyl]acetamide | Amino acid | 5 | 3.73 | 0.44 | Fixed-effect model | 1.50 | 0.38 - 5.83 | 0.56 |
| X-12510--2-aminooctanoic acid | Amino acid | 6 | 3.92 | 0.56 | Fixed-effect model | 0.79 | 0.49 - 1.28 | 0.34 |
| 1,5-anhydroglucitol (1,5-AG) | Carbohydrate | 6 | 2.30 | 0.81 | Fixed-effect model | 0.44 | 0.20 - 0.97 | 0.04 |
| erythronate | Carbohydrate | 3 | 3.18 | 0.20 | Fixed-effect model | 0.61 | 0.03 - 10.95 | 0.74 |
| mannose | Carbohydrate | 5 | 0.45 | 0.98 | Fixed-effect model | 0.96 | 0.37 - 2.55 | 0.94 |
| Acetate | Cofactors and vitamins | 18 | 9.79 | 0.91 | Fixed-effect model | 1.07 | 0.70 - 1.63 | 0.76 |
| Acetoacetate | Cofactors and vitamins | 6 | 2.94 | 0.71 | Fixed-effect model | 0.76 | 0.36 - 1.59 | 0.46 |
| bilirubin (E,E) | Cofactors and vitamins | 6 | 4.13 | 0.53 | Fixed-effect model | 1.05 | 0.75 - 1.48 | 0.78 |
| bilirubin (E,Z or Z,E) | Cofactors and vitamins | 4 | 3.10 | 0.38 | Fixed-effect model | 0.83 | 0.47 - 1.46 | 0.52 |
| bilirubin (Z,Z) | Cofactors and vitamins | 8 | 8.46 | 0.29 | Fixed-effect model | 1.00 | 0.78 - 1.29 | 0.99 |
| biliverdin | Cofactors and vitamins | 8 | 7.17 | 0.41 | Fixed-effect model | 1.15 | 0.79 - 1.67 | 0.48 |
| X-11593--O-methylascorbate | Cofactors and vitamins | 13 | 10.90 | 0.54 | Fixed-effect model | 0.21 | 0.10 - 0.43 | 0.00 |
| X-11793--oxidized bilirubin | Cofactors and vitamins | 10 | 5.07 | 0.83 | Fixed-effect model | 0.71 | 0.46 - 1.11 | 0.13 |
| Acetone | Energy | 15 | 6.80 | 0.94 | Fixed-effect model | 0.94 | 0.59 - 1.51 | 0.81 |
| citrate | Energy | 7 | 9.62 | 0.14 | Fixed-effect model | 0.12 | 0.02 - 0.82 | 0.03 |
| succinylcarnitine | Energy | 10 | 9.14 | 0.42 | Fixed-effect model | 1.90 | 0.69 - 5.22 | 0.21 |
| 10-undecenoate (11:1n1) | Lipid | 3 | 1.47 | 0.48 | Fixed-effect model | 1.17 | 0.44 - 3.08 | 0.75 |
| 18:2, linoleic acid (LA) | Lipid | 19 | 199.96 | 1.02E-32 | Random effect model | 1.29 | 0.89 - 1.89 | 0.18 |
| 1-arachidonoylglycerophosphocholine | Lipid | 5 | 3.96 | 0.41 | Fixed-effect model | 0.68 | 0.32 - 1.45 | 0.32 |
| 1-arachidonoylglycerophosphoethanolamine | Lipid | 3 | 0.53 | 0.77 | Fixed-effect model | 0.20 | 0.04 - 1.07 | 0.06 |
| 1-arachidonoylglycerophosphoinositol | Lipid | 3 | 1.64 | 0.44 | Fixed-effect model | 0.93 | 0.29 - 2.95 | 0.90 |
| 22:6, docosahexaenoic acid | Lipid | 5 | 7.75 | 0.10 | Fixed-effect model | 1.02 | 0.79 - 1.32 | 0.89 |
| 3-dehydrocarnitine | Lipid | 5 | 5.19 | 0.27 | Fixed-effect model | 4.49 | 1.14 - 17.74 | 0.03 |
| 4-androsten-3beta,17beta-diol disulfate 1 | Lipid | 6 | 6.16 | 0.29 | Fixed-effect model | 0.79 | 0.59 - 1.08 | 0.14 |
| 5alpha-androstan-3beta,17beta-diol disulfate | Lipid | 6 | 6.73 | 0.24 | Fixed-effect model | 0.82 | 0.62 - 1.08 | 0.16 |
| androsterone sulfate | Lipid | 8 | 7.52 | 0.38 | Fixed-effect model | 0.91 | 0.78 - 1.07 | 0.28 |
| Apolipoprotein A1 | Lipid | 201 | 438.03 | 7.56E-20 | Random effect model | 1.01 | 0.86 - 1.18 | 0.93 |
| Apolipoprotein B | Lipid | 139 | 434.55 | 2.10E-32 | Random effect model | 1.28 | 1.08 - 1.51 | 4.68E-03 |
| arachidonate (20:4n6) | Lipid | 3 | 4.46 | 0.11 | Fixed-effect model | 0.35 | 0.08 - 1.45 | 0.15 |
| butyrylcarnitine | Lipid | 17 | 19.58 | 0.24 | Fixed-effect model | 0.90 | 0.73 - 1.13 | 0.36 |
| carnitine | Lipid | 20 | 21.13 | 0.33 | Fixed-effect model | 2.04 | 0.44 - 9.46 | 0.36 |
| cis-4-decenoyl carnitine | Lipid | 5 | 2.40 | 0.66 | Fixed-effect model | 0.52 | 0.26 - 1.07 | 0.07 |
| Clinical LDL cholesterol | Lipid | 129 | 385.72 | 1.22E-27 | Random effect model | 1.47 | 1.25 - 1.73 | 3.92E-06 |
| decanoylcarnitine | Lipid | 5 | 3.69 | 0.45 | Fixed-effect model | 0.74 | 0.39 - 1.41 | 0.36 |
| dihomo-linolenate (20:3n3 or n6) | Lipid | 3 | 1.82 | 0.40 | Fixed-effect model | 0.85 | 0.15 - 4.73 | 0.85 |
| Docosahexaenoic acid | Lipid | 138 | 198.20 | 4.91E-04 | Random effect model | 1.03 | 0.93 - 1.15 | 0.54 |
| clinical low density lipoprotein cholesterol | Lipid | 7 | 6.73 | 0.35 | Fixed-effect model | 0.86 | 0.67 - 1.09 | 0.21 |
| Free cholesterol | Lipid | 34 | 228.12 | 4.98E-31 | Random effect model | 1.12 | 0.91 - 1.38 | 0.27 |
| HDL cholesterol | Lipid | 228 | 448.28 | 1.17E-16 | Random effect model | 1.15 | 1.01 - 1.31 | 0.03 |
| hexadecanedioate | Lipid | 3 | 0.09 | 0.95 | Fixed-effect model | 0.86 | 0.50 - 1.46 | 0.56 |
| hexanoylcarnitine | Lipid | 7 | 6.36 | 0.38 | Fixed-effect model | 0.62 | 0.34 - 1.12 | 0.11 |
| LDL cholesterol | Lipid | 130 | 411.12 | 3.61E-31 | Random effect model | 1.50 | 1.26 - 1.79 | 3.74E-06 |
| Mono-unsaturated fatty acids | Lipid | 180 | 451.04 | 1.94E-25 | Random effect model | 0.96 | 0.80 - 1.15 | 0.65 |
| octadecanedioate | Lipid | 3 | 0.04 | 0.98 | Fixed-effect model | 0.60 | 0.19 - 1.91 | 0.39 |
| octanoylcarnitine | Lipid | 5 | 3.11 | 0.54 | Fixed-effect model | 0.70 | 0.40 - 1.21 | 0.20 |
| Omega-3 fatty acids | Lipid | 201 | 464.60 | 4.18E-23 | Random effect model | 1.07 | 0.96 - 1.20 | 0.22 |
| Omega-6 fatty acids | Lipid | 146 | 430.40 | 4.51E-30 | Random effect model | 1.19 | 0.97 - 1.46 | 0.10 |
| Omega-7, omega-9 and saturated fatty acids | Lipid | 6 | 6.67 | 0.25 | Fixed-effect model | 0.91 | 0.70 - 1.18 | 0.46 |
| Other polyunsaturated fatty acids than 18:2 | Lipid | 42 | 59.85 | 0.03 | Random effect model | 0.99 | 0.91 - 1.08 | 0.87 |
| Phosphatidylcholine and other cholines | Lipid | 11 | 10.42 | 0.40 | Fixed-effect model | 0.96 | 0.84 - 1.11 | 0.60 |
| Polyunsaturated fatty acids | Lipid | 183 | 357.22 | 1.70E-13 | Random effect model | 1.12 | 0.97 - 1.30 | 0.11 |
| propionylcarnitine | Lipid | 4 | 2.85 | 0.42 | Fixed-effect model | 1.86 | 0.31 - 11.23 | 0.50 |
| Remnant cholesterol (non-HDL, non-LDL -cholesterol) | Lipid | 138 | 423.18 | 5.84E-31 | Random effect model | 1.30 | 1.08 - 1.56 | 0.01 |
| Saturated fatty acids | Lipid | 134 | 362.98 | 3.17E-23 | Random effect model | 1.02 | 0.82 - 1.27 | 0.89 |
| Serum total cholesterol | Lipid | 51 | 279.06 | 1.46E-33 | Random effect model | 1.25 | 1.04 - 1.50 | 0.02 |
| Serum total triglycerides | Lipid | 16 | 222.39 | 5.78E-39 | Random effect model | 1.43 | 0.76 - 2.69 | 0.26 |
| Sphingomyelins | Lipid | 163 | 430.53 | 3.13E-26 | Random effect model | 1.47 | 1.25 - 1.74 | 5.97E-06 |
| tetradecanedioate | Lipid | 3 | 0.13 | 0.94 | Fixed-effect model | 0.87 | 0.53 - 1.41 | 0.57 |
| Total cholesterol minus HDL-C | Lipid | 135 | 404.89 | 5.36E-29 | Random effect model | 1.33 | 1.12 - 1.58 | 1.07E-03 |
| Total esterified cholesterol | Lipid | 142 | 431.54 | 3.29E-31 | Random effect model | 1.59 | 1.31 - 1.92 | 1.98E-06 |
| Total fatty acids | Lipid | 11 | 205.57 | 1.11E-38 | Random effect model | 1.25 | 0.58 - 2.68 | 0.57 |
| Total phosphoglycerides | Lipid | 11 | 8.20 | 0.61 | Fixed-effect model | 0.97 | 0.84 - 1.12 | 0.67 |
| VLDL cholesterol | Lipid | 158 | 321.67 | 2.08E-13 | Random effect model | 1.09 | 0.93 - 1.28 | 0.29 |
| X-11445--5-alpha-pregnan-3beta,20alpha-disulfate | Lipid | 3 | 1.41 | 0.49 | Fixed-effect model | 0.92 | 0.54 - 1.58 | 0.77 |
| X-13431--nonanoylcarnitine | Lipid | 7 | 3.05 | 0.80 | Fixed-effect model | 0.71 | 0.49 - 1.04 | 0.08 |
| urate | Nucleotide | 3 | 7.90 | 0.02 | Random effect model | 0.56 | 0.003 - 90.17 | 0.82 |
| uridine | Nucleotide | 3 | 1.61 | 0.45 | Fixed-effect model | 2.04 | 0.09 - 44.77 | 0.65 |
| Albumin | Peptide | 38 | 45.91 | 0.15 | Fixed-effect model | 0.95 | 0.75 - 1.20 | 0.65 |
| bradykinin, des-arg(9) | Peptide | 5 | 1.95 | 0.75 | Fixed-effect model | 0.98 | 0.78 - 1.23 | 0.88 |
| gamma-glutamylglutamine | Peptide | 3 | 0.24 | 0.88 | Fixed-effect model | 1.35 | 0.16 - 11.48 | 0.79 |
| gamma-glutamyltyrosine | Peptide | 3 | 0.97 | 0.62 | Fixed-effect model | 0.88 | 0.04 - 17.90 | 0.94 |
| Glycoprotein acetyls | Peptide | 131 | 173.80 | 0.01 | Random effect model | 1.05 | 0.92 - 1.21 | 0.44 |
| Glycoproteins | Peptide | 72 | 59.20 | 0.84 | Fixed-effect model | 0.95 | 0.90 - 1.00 | 0.06 |
| HWESASXX | Peptide | 3 | 0.21 | 0.90 | Fixed-effect model | 1.82 | 0.81 - 4.10 | 0.15 |
| X-12244--N-acetylcarnosine | Peptide | 6 | 6.02 | 0.30 | Fixed-effect model | 1.17 | 0.37 - 3.69 | 0.79 |
| X-02269 | Unknown | 3 | 0.72 | 0.70 | Fixed-effect model | 2.90 | 1.47 - 5.73 | 0.00 |
| X-03094 | Unknown | 3 | 0.06 | 0.97 | Fixed-effect model | 0.75 | 0.10 - 5.84 | 0.79 |
| X-08402 | Unknown | 6 | 8.54 | 0.13 | Fixed-effect model | 1.36 | 0.57 - 3.27 | 0.49 |
| X-08988 | Unknown | 3 | 2.30 | 0.32 | Fixed-effect model | 0.86 | 0.19 - 3.90 | 0.85 |
| X-10510 | Unknown | 3 | 4.43 | 0.11 | Fixed-effect model | 1.51 | 0.36 - 6.37 | 0.58 |
| X-11204 | Unknown | 3 | 1.89 | 0.39 | Fixed-effect model | 0.48 | 0.02 - 9.82 | 0.63 |
| X-11261 | Unknown | 5 | 1.16 | 0.89 | Fixed-effect model | 1.81 | 0.79 - 4.14 | 0.16 |
| X-11315 | Unknown | 3 | 0.55 | 0.76 | Fixed-effect model | 0.37 | 0.07 - 1.99 | 0.25 |
| X-11440 | Unknown | 5 | 4.42 | 0.35 | Fixed-effect model | 0.87 | 0.60 - 1.24 | 0.43 |
| X-11441 | Unknown | 4 | 7.03 | 0.07 | Fixed-effect model | 1.16 | 0.77 - 1.76 | 0.48 |
| X-11442 | Unknown | 6 | 4.08 | 0.54 | Fixed-effect model | 1.09 | 0.73 - 1.63 | 0.67 |
| X-11444 | Unknown | 3 | 1.23 | 0.54 | Fixed-effect model | 0.92 | 0.27 - 3.18 | 0.90 |
| X-11469 | Unknown | 4 | 1.05 | 0.79 | Fixed-effect model | 2.88 | 1.55 - 5.32 | 0.00 |
| X-11491 | Unknown | 4 | 1.58 | 0.66 | Fixed-effect model | 0.86 | 0.49 - 1.48 | 0.58 |
| X-11529 | Unknown | 11 | 4.42 | 0.93 | Fixed-effect model | 0.95 | 0.81 - 1.12 | 0.55 |
| X-11530 | Unknown | 8 | 6.86 | 0.44 | Fixed-effect model | 1.04 | 0.71 - 1.51 | 0.85 |
| X-11538 | Unknown | 7 | 0.19 | 1.00 | Fixed-effect model | 0.86 | 0.58 - 1.28 | 0.46 |
| X-11787 | Unknown | 7 | 6.50 | 0.37 | Fixed-effect model | 1.23 | 0.24 - 6.21 | 0.80 |
| X-11792 | Unknown | 3 | 2.04 | 0.36 | Fixed-effect model | 0.98 | 0.69 - 1.38 | 0.89 |
| X-11905 | Unknown | 3 | 0.09 | 0.95 | Fixed-effect model | 0.69 | 0.36 - 1.30 | 0.25 |
| X-12063 | Unknown | 14 | 9.77 | 0.71 | Fixed-effect model | 0.73 | 0.57 - 0.93 | 0.01 |
| X-12092 | Unknown | 16 | 10.42 | 0.79 | Fixed-effect model | 0.78 | 0.67 - 0.92 | 0.00 |
| X-12093 | Unknown | 3 | 1.77 | 0.41 | Fixed-effect model | 1.44 | 0.85 - 2.42 | 0.17 |
| X-12456 | Unknown | 3 | 1.14 | 0.56 | Fixed-effect model | 0.76 | 0.41 - 1.43 | 0.40 |
| X-12556 | Unknown | 4 | 20.55 | 1.30E-04 | Random effect model | 0.66 | 0.02 - 28.79 | 0.83 |
| X-12644 | Unknown | 3 | 2.82 | 0.24 | Fixed-effect model | 0.36 | 0.07 - 1.91 | 0.23 |
| X-12696 | Unknown | 5 | 2.06 | 0.72 | Fixed-effect model | 0.36 | 0.13 - 1.02 | 0.06 |
| X-12728 | Unknown | 8 | 3.11 | 0.87 | Fixed-effect model | 1.01 | 0.96 - 1.07 | 0.64 |
| X-12798 | Unknown | 12 | 13.47 | 0.26 | Fixed-effect model | 0.71 | 0.47 - 1.07 | 0.10 |
| X-12844 | Unknown | 4 | 2.35 | 0.50 | Fixed-effect model | 0.59 | 0.16 - 2.15 | 0.42 |
| X-13429 | Unknown | 4 | 0.27 | 0.96 | Fixed-effect model | 0.93 | 0.67 - 1.31 | 0.69 |
| X-13435 | Unknown | 3 | 3.95 | 0.14 | Fixed-effect model | 4.18 | 1.37 - 12.78 | 0.01 |
| X-14626 | Unknown | 3 | 1.39 | 0.50 | Fixed-effect model | 0.86 | 0.34 - 2.19 | 0.75 |

Odds ratios (ORs) with their 95% confidence intervals (CIs) represent the association estimates with the risks of delirium of per 1–standard deviation increase in blood metabolite levels. Significant threshold was set at *P*<3.88 x 10-4 (Bonferroni-corrected significance threshold calculated as 0.05 divided by 129 [for 119 blood metabolites]). The threshold of significance for heterogeneity was set at *P*<0.05. E signifies the exponent of 10 in the table.

Abbreviations: MR, Mendelian randomization; SNPs, single nucleotide polymorphisms.

**Table S6. Sensitivity analyses for significant blood metabolites.**

| **Metabolites** | **Super-pathway** | **SNPs** | **Weighted median** | | **MR-Egger** | | **MR-Egger Intercept** | |
| --- | --- | --- | --- | --- | --- | --- | --- | --- |
|  |  |  | **OR (95% CI)** | ***P* value** | **OR (95% CI)** | ***P* value** | **Estimate /Standard error** | ***P* value** |
| Clinical LDL cholesterol | Lipid | 129 | 1.27 (1.07-1.51) | 0.01 | 1.76 (1.36-2.27) | 3.11E-05 | -0.015/0.008 | 0.08 |
| LDL cholesterol | Lipid | 130 | 1.28 (1.07-1.53) | 0.01 | 1.98 (1.51-2.59) | 2.47E-06 | -0.022/0.009 | 0.01 |
| Sphingomyelins | Lipid | 163 | 1.41 (1.16-1.70) | 4.14E-04 | 1.69 (1.26-2.27) | 5.55E-04 | -0.009/0.008 | 0.26 |
| Total esterified cholesterol | Lipid | 142 | 1.46 (1.19-1.78) | 2.17E-04 | 2.15 (1.55-2.98) | 8.51E-06 | -0.019/0.009 | 0.03 |
| O-methylascorbate | Cofactors and vitamins | 13 | 0.40 (0.15-1.05) | 0.06 | 0.31 (0.07-1.28) | 0.13 | -0.012/0.019 | 0.55 |

Odds ratios (ORs) with their 95% confidence intervals (CIs) represent the association estimates with the risk of delirium of per 1-SD increase in clinical low density lipoprotein (LDL) cholesterol, low density lipoprotein cholesterol, sphingomyelins, total esterified cholesterol, and O-methylascorbate levels, respectively.

Significant threshold was set at *P*<3.88 x 10^-4^ (Bonferroni-corrected significance threshold calculated as 0.05 divided by 129 [for 119 blood metabolites]). E signifies the exponent of 10 in the table.

Abbreviations: SNPs, single nucleotide polymorphisms.

**Table S7. Phe-MR analyses for the associations between Clinical LDL cholesterol and 678 diseases using the inverse-variance weighted method.**

| **PheCode** | **Phenotype Description** | **Disease Chapter** | **Q estimate** | ***P* value for Q estimate** | **IVW model** | **OR** | **95% CI** | ***P* value** |
| --- | --- | --- | --- | --- | --- | --- | --- | --- |
| 8 | Intestinal infection | infectious diseases | 114.05 | 0.64 | Fixed-effect model | 1.02 | 0.97 - 1.08 | 0.41 |
| 8.5 | Bacterial enteritis | infectious diseases | 126.55 | 0.32 | Fixed-effect model | 0.99 | 0.90 - 1.09 | 0.85 |
| 8.52 | Intestinal infection due to C. difficile | infectious diseases | 124.73 | 0.37 | Fixed-effect model | 1.01 | 0.82 - 1.24 | 0.92 |
| 8.6 | Viral Enteritis | infectious diseases | 137.25 | 0.13 | Fixed-effect model | 1.08 | 0.90 - 1.28 | 0.41 |
| 38 | Septicemia | infectious diseases | 114.21 | 0.63 | Fixed-effect model | 0.85 | 0.78 - 0.92 | 1.00E-04 |
| 38.1 | Gram negative septicemia | infectious diseases | 129.14 | 0.27 | Fixed-effect model | 0.93 | 0.78 - 1.11 | 0.41 |
| 41 | Bacterial infection NOS | infectious diseases | 132.53 | 0.20 | Fixed-effect model | 0.99 | 0.95 - 1.04 | 0.81 |
| 41.1 | Staphylococcus infections | infectious diseases | 159.32 | 0.01 | Random effect model | 0.97 | 0.87 - 1.08 | 0.61 |
| 41.2 | Streptococcus infection | infectious diseases | 115.07 | 0.61 | Fixed-effect model | 0.92 | 0.81 - 1.04 | 0.17 |
| 41.4 | E. coli | infectious diseases | 131.11 | 0.23 | Fixed-effect model | 0.97 | 0.88 - 1.07 | 0.50 |
| 70 | Viral hepatitis | infectious diseases | 145.40 | 0.06 | Fixed-effect model | 1.11 | 0.96 - 1.29 | 0.16 |
| 78 | Viral warts & HPV | infectious diseases | 99.96 | 0.91 | Fixed-effect model | 0.97 | 0.82 - 1.14 | 0.70 |
| 79 | Viral infection | infectious diseases | 99.43 | 0.91 | Fixed-effect model | 0.99 | 0.90 - 1.09 | 0.82 |
| 80 | Postoperative infection | infectious diseases | 155.71 | 0.02 | Random effect model | 1.02 | 0.94 - 1.12 | 0.60 |
| 81 | Infection/inflammation of internal prosthetic device; implant; and graft | infectious diseases | 108.81 | 0.76 | Fixed-effect model | 0.93 | 0.84 - 1.04 | 0.19 |
| 112 | Candidiasis | infectious diseases | 117.01 | 0.56 | Fixed-effect model | 0.95 | 0.85 - 1.06 | 0.34 |
| 145 | Cancer of mouth | neoplasms | 137.75 | 0.13 | Fixed-effect model | 0.96 | 0.78 - 1.18 | 0.70 |
| 149 | Cancer of larynx, pharynx, nasal cavities | neoplasms | 135.68 | 0.16 | Fixed-effect model | 0.95 | 0.78 - 1.17 | 0.66 |
| 150 | Cancer of esophagus | neoplasms | 126.09 | 0.33 | Fixed-effect model | 0.77 | 0.64 - 0.94 | 0.01 |
| 151 | Cancer of stomach | neoplasms | 96.70 | 0.94 | Fixed-effect model | 0.74 | 0.59 - 0.92 | 0.01 |
| 153 | Colorectal cancer | neoplasms | 121.25 | 0.45 | Fixed-effect model | 1.12 | 1.04 - 1.21 | 4.51E-03 |
| 153.2 | Colon cancer | neoplasms | 140.33 | 0.10 | Fixed-effect model | 1.06 | 0.97 - 1.17 | 0.20 |
| 153.3 | Malignant neoplasm of rectum, rectosigmoid junction, and anus | neoplasms | 108.98 | 0.76 | Fixed-effect model | 1.14 | 1.02 - 1.28 | 0.02 |
| 157 | Pancreatic cancer | neoplasms | 118.79 | 0.51 | Fixed-effect model | 0.74 | 0.60 - 0.92 | 0.01 |
| 158 | Neoplasm of unspecified nature of digestive system | neoplasms | 125.74 | 0.34 | Fixed-effect model | 1.00 | 0.85 - 1.18 | 0.98 |
| 159 | Malignant neoplasm of other and ill-defined sites within the digestive organs and peritoneum | neoplasms | 135.53 | 0.16 | Fixed-effect model | 1.02 | 0.95 - 1.10 | 0.54 |
| 165 | Cancer within the respiratory system | neoplasms | 150.10 | 0.03 | Random effect model | 0.93 | 0.84 - 1.05 | 0.24 |
| 165.1 | Cancer of bronchus; lung | neoplasms | 145.57 | 0.06 | Fixed-effect model | 0.91 | 0.81 - 1.01 | 0.09 |
| 172 | Skin cancer | neoplasms | 219.11 | 0.00 | Random effect model | 1.02 | 0.96 - 1.09 | 0.53 |
| 172.1 | Melanomas of skin, dx or hx | neoplasms | 149.59 | 0.03 | Random effect model | 1.04 | 0.93 - 1.16 | 0.52 |
| 172.11 | Melanomas of skin | neoplasms | 149.59 | 0.03 | Random effect model | 1.04 | 0.93 - 1.16 | 0.52 |
| 172.2 | Other non-epithelial cancer of skin | neoplasms | 200.40 | 0.00 | Random effect model | 1.01 | 0.95 - 1.08 | 0.77 |
| 172.3 | Carcinoma in situ of skin | neoplasms | 114.33 | 0.63 | Fixed-effect model | 1.26 | 1.03 - 1.54 | 0.02 |
| 174 | Breast cancer | neoplasms | 143.54 | 0.07 | Fixed-effect model | 1.06 | 1.01 - 1.12 | 0.01 |
| 189 | Cancer of urinary organs (incl. kidney and bladder) | neoplasms | 118.44 | 0.52 | Fixed-effect model | 1.05 | 0.97 - 1.14 | 0.21 |
| 189.1 | Cancer of kidney and renal pelvis | neoplasms | 139.80 | 0.10 | Fixed-effect model | 1.14 | 0.97 - 1.34 | 0.10 |
| 189.11 | Malignant neoplasm of kidney, except pelvis | neoplasms | 135.55 | 0.16 | Fixed-effect model | 1.15 | 0.98 - 1.36 | 0.08 |
| 189.2 | Cancer of bladder | neoplasms | 87.44 | 0.99 | Fixed-effect model | 1.06 | 0.96 - 1.18 | 0.26 |
| 189.21 | Malignant neoplasm of bladder | neoplasms | 87.14 | 0.99 | Fixed-effect model | 1.12 | 1.00 - 1.26 | 0.04 |
| 191 | Manlignant and unknown neoplasms of brain and nervous system | neoplasms | 106.48 | 0.81 | Fixed-effect model | 1.38 | 1.13 - 1.69 | 1.71E-03 |
| 191.1 | Cancer of brain and nervous system | neoplasms | 99.27 | 0.92 | Fixed-effect model | 1.41 | 1.13 - 1.77 | 2.71E-03 |
| 195 | Cancer, suspected or other | neoplasms | 123.03 | 0.41 | Fixed-effect model | 1.03 | 0.99 - 1.08 | 0.10 |
| 195.1 | Malignant neoplasm, other | neoplasms | 118.15 | 0.53 | Fixed-effect model | 1.04 | 1.00 - 1.09 | 0.04 |
| 197 | Chemotherapy | neoplasms | 104.29 | 0.85 | Fixed-effect model | 0.96 | 0.93 - 1.00 | 0.03 |
| 198 | Secondary malignant neoplasm | neoplasms | 119.50 | 0.50 | Fixed-effect model | 0.99 | 0.94 - 1.05 | 0.75 |
| 198.1 | Secondary malignancy of lymph nodes | neoplasms | 129.99 | 0.25 | Fixed-effect model | 0.98 | 0.91 - 1.05 | 0.49 |
| 198.2 | Secondary malignancy of respiratory organs | neoplasms | 119.53 | 0.49 | Fixed-effect model | 0.98 | 0.88 - 1.09 | 0.73 |
| 198.3 | Secondary malignant neoplasm of digestive systems | neoplasms | 119.65 | 0.49 | Fixed-effect model | 1.02 | 0.89 - 1.16 | 0.82 |
| 198.4 | Secondary malignant neoplasm of liver | neoplasms | 89.37 | 0.98 | Fixed-effect model | 0.94 | 0.85 - 1.04 | 0.25 |
| 198.5 | Secondary malignancy of brain/spine | neoplasms | 128.83 | 0.27 | Fixed-effect model | 0.94 | 0.79 - 1.13 | 0.53 |
| 198.6 | Secondary malignancy of bone | neoplasms | 94.74 | 0.96 | Fixed-effect model | 1.10 | 0.98 - 1.23 | 0.09 |
| 199 | Neoplasm of uncertain behavior | neoplasms | 73.84 | 1.00 | Fixed-effect model | 1.08 | 0.95 - 1.24 | 0.25 |
| 200 | Myeloproliferative disease | neoplasms | 183.27 | 1.78E-04 | Random effect model | 0.73 | 0.60 - 0.90 | 2.45E-03 |
| 202 | Cancer of other lymphoid, histiocytic tissue | neoplasms | 156.21 | 0.01 | Random effect model | 1.03 | 0.91 - 1.17 | 0.61 |
| 202.2 | Non-Hodgkins lymphoma | neoplasms | 151.96 | 0.03 | Random effect model | 1.03 | 0.90 - 1.18 | 0.67 |
| 202.24 | Large cell lymphoma | neoplasms | 127.71 | 0.30 | Fixed-effect model | 0.81 | 0.65 - 1.00 | 0.05 |
| 204 | Leukemia | neoplasms | 110.77 | 0.72 | Fixed-effect model | 1.01 | 0.89 - 1.14 | 0.91 |
| 204.1 | Lymphoid leukemia | neoplasms | 127.03 | 0.31 | Fixed-effect model | 0.97 | 0.78 - 1.20 | 0.79 |
| 204.12 | Lymphoid leukemia, chronic | neoplasms | 128.94 | 0.27 | Fixed-effect model | 0.94 | 0.75 - 1.18 | 0.60 |
| 204.4 | Multiple myeloma | neoplasms | 133.22 | 0.19 | Fixed-effect model | 0.97 | 0.78 - 1.21 | 0.78 |
| 208 | Benign neoplasm of colon | neoplasms | 228.25 | 0.00 | Random effect model | 1.05 | 1.00 - 1.11 | 0.05 |
| 210 | Benign neoplasm of lip, oral cavity, and pharynx | neoplasms | 95.89 | 0.95 | Fixed-effect model | 0.91 | 0.77 - 1.08 | 0.28 |
| 211 | Benign neoplasm of other parts of digestive system | neoplasms | 141.62 | 0.09 | Fixed-effect model | 0.98 | 0.92 - 1.06 | 0.66 |
| 214 | Lipoma | neoplasms | 138.48 | 0.12 | Fixed-effect model | 0.99 | 0.93 - 1.06 | 0.88 |
| 214.1 | Lipoma of skin and subcutaneous tissue | neoplasms | 143.94 | 0.07 | Fixed-effect model | 0.99 | 0.92 - 1.07 | 0.81 |
| 215 | Other benign neoplasm of connective and other soft tissue | neoplasms | 131.58 | 0.22 | Fixed-effect model | 0.97 | 0.83 - 1.13 | 0.72 |
| 216 | Benign neoplasm of skin | neoplasms | 109.72 | 0.74 | Fixed-effect model | 1.03 | 0.97 - 1.09 | 0.38 |
| 217 | Vascular hamartomas and non-neoplastic nevi | neoplasms | 109.11 | 0.75 | Fixed-effect model | 0.95 | 0.78 - 1.17 | 0.65 |
| 217.1 | Nevus, non-neoplastic | neoplasms | 105.10 | 0.83 | Fixed-effect model | 1.00 | 0.81 - 1.23 | 0.98 |
| 225 | Benign neoplasm of brain and other parts of nervous system | neoplasms | 117.47 | 0.55 | Fixed-effect model | 1.11 | 0.92 - 1.32 | 0.27 |
| 225.1 | Benign neoplasm of brain, cranial nerves, meninges | neoplasms | 116.98 | 0.56 | Fixed-effect model | 1.14 | 0.95 - 1.37 | 0.17 |
| 227 | Benign neoplasm of other endocrine glands and related structures | neoplasms | 114.52 | 0.62 | Fixed-effect model | 1.17 | 0.98 - 1.39 | 0.08 |
| 228 | Hemangioma and lymphangioma, any site | neoplasms | 136.75 | 0.14 | Fixed-effect model | 0.97 | 0.85 - 1.11 | 0.67 |
| 229 | Benign neoplasm of unspecified sites | neoplasms | 118.14 | 0.53 | Fixed-effect model | 1.06 | 0.96 - 1.18 | 0.27 |
| 240 | Simple and unspecified goiter | endocrine/metabolic | 119.00 | 0.51 | Fixed-effect model | 0.89 | 0.72 - 1.10 | 0.26 |
| 241 | Nontoxic nodular goiter | endocrine/metabolic | 107.16 | 0.79 | Fixed-effect model | 0.90 | 0.77 - 1.05 | 0.18 |
| 241.2 | Nontoxic multinodular goiter | endocrine/metabolic | 120.42 | 0.47 | Fixed-effect model | 0.70 | 0.57 - 0.85 | 4.21E-04 |
| 244 | Hypothyroidism | endocrine/metabolic | 328.62 | 0.00 | Random effect model | 0.97 | 0.90 - 1.05 | 0.47 |
| 244.1 | Secondary hypothyroidism | endocrine/metabolic | 124.53 | 0.37 | Fixed-effect model | 0.95 | 0.81 - 1.11 | 0.49 |
| 244.4 | Hypothyroidism NOS | endocrine/metabolic | 330.30 | 0.00 | Random effect model | 0.98 | 0.91 - 1.06 | 0.57 |
| 250 | Diabetes mellitus | endocrine/metabolic | 223.57 | 3.04E-08 | Random effect model | 0.92 | 0.87 - 0.97 | 1.22E-03 |
| 250.1 | Type 1 diabetes | endocrine/metabolic | 185.05 | 0.00 | Random effect model | 0.93 | 0.82 - 1.05 | 0.25 |
| 250.2 | Type 2 diabetes | endocrine/metabolic | 223.63 | 3.00E-08 | Random effect model | 0.91 | 0.86 - 0.96 | 6.69E-04 |
| 250.23 | Type 2 diabetes with ophthalmic manifestations | endocrine/metabolic | 122.51 | 0.42 | Fixed-effect model | 0.84 | 0.73 - 0.97 | 0.02 |
| 250.24 | Type 2 diabetes with neurological manifestations | endocrine/metabolic | 115.77 | 0.59 | Fixed-effect model | 1.02 | 0.82 - 1.26 | 0.86 |
| 250.4 | Abnormal glucose | endocrine/metabolic | 114.27 | 0.63 | Fixed-effect model | 0.85 | 0.70 - 1.03 | 0.10 |
| 250.7 | Diabetic retinopathy | endocrine/metabolic | 129.58 | 0.26 | Fixed-effect model | 0.85 | 0.74 - 0.98 | 0.03 |
| 251 | Other disorders of pancreatic internal secretion | endocrine/metabolic | 127.07 | 0.31 | Fixed-effect model | 0.87 | 0.74 - 1.03 | 0.12 |
| 251.1 | Hypoglycemia | endocrine/metabolic | 127.80 | 0.30 | Fixed-effect model | 0.87 | 0.73 - 1.03 | 0.10 |
| 252 | Disorders of parathyroid gland | endocrine/metabolic | 125.32 | 0.35 | Fixed-effect model | 1.43 | 1.20 - 1.71 | 6.24E-05 |
| 252.1 | Hyperparathyroidism | endocrine/metabolic | 119.47 | 0.50 | Fixed-effect model | 1.34 | 1.12 - 1.61 | 1.61E-03 |
| 253 | Disorders of the pituitary gland and its hypothalamic control | endocrine/metabolic | 115.70 | 0.59 | Fixed-effect model | 1.30 | 1.07 - 1.58 | 0.01 |
| 255 | Disorders of adrenal glands | endocrine/metabolic | 112.78 | 0.67 | Fixed-effect model | 0.88 | 0.71 - 1.07 | 0.20 |
| 260 | Protein-calorie malnutrition | endocrine/metabolic | 100.47 | 0.90 | Fixed-effect model | 0.83 | 0.70 - 0.97 | 0.02 |
| 260.6 | Anorexia | endocrine/metabolic | 105.97 | 0.82 | Fixed-effect model | 0.81 | 0.68 - 0.97 | 0.02 |
| 261 | Vitamin deficiency | endocrine/metabolic | 120.91 | 0.46 | Fixed-effect model | 1.12 | 0.97 - 1.31 | 0.12 |
| 261.2 | Vitamin B-complex deficiencies | endocrine/metabolic | 122.88 | 0.41 | Fixed-effect model | 1.10 | 0.91 - 1.33 | 0.32 |
| 272 | Disorders of lipid metabolism | endocrine/metabolic | 659.99 | 1.66E-75 | Random effect model | 2.18 | 2.03 - 2.34 | 1.68E-100 |
| 272.1 | Hyperlipidemia | endocrine/metabolic | 654.73 | 1.44E-74 | Random effect model | 2.18 | 2.03 - 2.34 | 6.68E-101 |
| 272.11 | Hypercholesterolemia | endocrine/metabolic | 627.83 | 8.46E-70 | Random effect model | 2.18 | 2.03 - 2.35 | 1.03E-100 |
| 274 | Gout and other crystal arthropathies | endocrine/metabolic | 142.18 | 0.08 | Fixed-effect model | 0.93 | 0.86 - 1.02 | 0.11 |
| 274.1 | Gout | endocrine/metabolic | 139.65 | 0.11 | Fixed-effect model | 0.89 | 0.81 - 0.98 | 0.02 |
| 274.2 | Crystal arthropathies | endocrine/metabolic | 97.49 | 0.93 | Fixed-effect model | 1.14 | 0.92 - 1.40 | 0.23 |
| 274.21 | Chondrocalcinosis | endocrine/metabolic | 96.15 | 0.95 | Fixed-effect model | 1.16 | 0.93 - 1.44 | 0.19 |
| 275 | Disorders of mineral metabolism | endocrine/metabolic | 2185.50 | 0.00 | Random effect model | 0.86 | 0.53 - 1.39 | 0.54 |
| 275.1 | Disorders of iron metabolism | hematopoietic | 534.99 | 0.00 | Random effect model | 0.79 | 0.52 - 1.21 | 0.28 |
| 275.5 | Disorders of calcium/phosphorus metabolism | endocrine/metabolic | 119.69 | 0.49 | Fixed-effect model | 1.15 | 0.99 - 1.34 | 0.06 |
| 276 | Disorders of fluid, electrolyte, and acid-base balance | endocrine/metabolic | 121.78 | 0.44 | Fixed-effect model | 1.05 | 0.99 - 1.11 | 0.13 |
| 276.1 | Electrolyte imbalance | endocrine/metabolic | 132.53 | 0.20 | Fixed-effect model | 1.09 | 1.00 - 1.18 | 0.04 |
| 276.13 | Hyperpotassemia | endocrine/metabolic | 127.68 | 0.30 | Fixed-effect model | 1.04 | 0.88 - 1.23 | 0.63 |
| 276.14 | Hypopotassemia | endocrine/metabolic | 96.89 | 0.94 | Fixed-effect model | 1.08 | 0.94 - 1.24 | 0.26 |
| 276.4 | Acid-base balance disorder | endocrine/metabolic | 107.09 | 0.79 | Fixed-effect model | 0.98 | 0.85 - 1.14 | 0.83 |
| 276.41 | Acidosis | endocrine/metabolic | 103.55 | 0.86 | Fixed-effect model | 0.95 | 0.81 - 1.11 | 0.50 |
| 276.5 | Hypovolemia | endocrine/metabolic | 118.73 | 0.52 | Fixed-effect model | 1.08 | 0.98 - 1.19 | 0.11 |
| 277 | Other disorders of metabolism | endocrine/metabolic | 100.30 | 0.90 | Fixed-effect model | 0.91 | 0.80 - 1.05 | 0.19 |
| 278 | Overweight, obesity and other hyperalimentation | endocrine/metabolic | 168.97 | 2.17E-03 | Random effect model | 0.91 | 0.85 - 0.96 | 1.68E-03 |
| 278.1 | Obesity | endocrine/metabolic | 168.05 | 2.51E-03 | Random effect model | 0.89 | 0.84 - 0.95 | 3.20E-04 |
| 280 | Iron deficiency anemias | hematopoietic | 173.86 | 9.60E-04 | Random effect model | 1.13 | 1.05 - 1.21 | 8.46E-04 |
| 280.1 | Iron deficiency anemias, unspecified or not due to blood loss | hematopoietic | 176.11 | 6.50E-04 | Random effect model | 1.15 | 1.06 - 1.23 | 3.09E-04 |
| 281 | Other deficiency anemia | hematopoietic | 165.58 | 0.00 | Random effect model | 1.23 | 1.03 - 1.47 | 0.02 |
| 281.1 | Megaloblastic anemia | hematopoietic | 159.28 | 0.01 | Random effect model | 1.23 | 1.02 - 1.47 | 0.03 |
| 281.11 | Pernicious anemia | hematopoietic | 185.02 | 0.00 | Random effect model | 1.22 | 0.97 - 1.55 | 0.09 |
| 285 | Other anemias | hematopoietic | 168.27 | 2.43E-03 | Random effect model | 1.10 | 1.04 - 1.16 | 1.19E-03 |
| 285.2 | Anemia of chronic disease | hematopoietic | 125.72 | 0.34 | Fixed-effect model | 1.16 | 0.95 - 1.41 | 0.14 |
| 286 | Coagulation defects | hematopoietic | 136.18 | 0.15 | Fixed-effect model | 0.96 | 0.81 - 1.14 | 0.66 |
| 287 | Purpura and other hemorrhagic conditions | hematopoietic | 118.97 | 0.51 | Fixed-effect model | 1.00 | 0.89 - 1.13 | 0.99 |
| 287.3 | Thrombocytopenia | hematopoietic | 144.39 | 0.06 | Fixed-effect model | 1.02 | 0.90 - 1.17 | 0.73 |
| 288 | Diseases of white blood cells | hematopoietic | 107.62 | 0.78 | Fixed-effect model | 1.03 | 0.95 - 1.12 | 0.49 |
| 288.1 | Decreased white blood cell count | hematopoietic | 117.14 | 0.56 | Fixed-effect model | 1.02 | 0.93 - 1.11 | 0.75 |
| 288.11 | Neutropenia | hematopoietic | 117.14 | 0.56 | Fixed-effect model | 1.02 | 0.93 - 1.11 | 0.75 |
| 289 | Other diseases of blood and blood-forming organs | hematopoietic | 122.40 | 0.42 | Fixed-effect model | 0.86 | 0.79 - 0.93 | 2.46E-04 |
| 289.4 | Lymphadenitis | hematopoietic | 124.72 | 0.37 | Fixed-effect model | 0.89 | 0.81 - 0.99 | 0.03 |
| 289.5 | Diseases of spleen | hematopoietic | 106.07 | 0.81 | Fixed-effect model | 0.82 | 0.65 - 1.02 | 0.08 |
| 290.1 | Dementias | mental disorders | 623.12 | 5.75E-69 | Random effect model | 3.02 | 2.06 - 4.43 | 1.59E-08 |
| 291 | Other specified nonpsychotic and/or transient mental disorders | mental disorders | 127.31 | 0.31 | Fixed-effect model | 0.82 | 0.67 - 1.01 | 0.06 |
| 292 | Neurological disorders | mental disorders | 198.24 | 9.18E-06 | Random effect model | 1.20 | 1.09 - 1.32 | 2.47E-04 |
| 292.1 | Aphasia/speech disturbance | mental disorders | 138.81 | 0.12 | Fixed-effect model | 1.27 | 1.11 - 1.45 | 4.38E-04 |
| 292.3 | Memory loss | mental disorders | 131.29 | 0.23 | Fixed-effect model | 0.76 | 0.63 - 0.93 | 0.01 |
| 292.4 | Altered mental status | mental disorders | 163.87 | 4.83E-03 | Random effect model | 1.39 | 1.22 - 1.58 | 7.63E-07 |
| 293 | Symptoms involving head and neck | mental disorders | 106.21 | 0.81 | Fixed-effect model | 1.04 | 0.95 - 1.14 | 0.41 |
| 293.1 | Swelling, mass, or lump in head and neck [Space occupying lesion, intracranial NOS] | mental disorders | 109.24 | 0.75 | Fixed-effect model | 1.16 | 0.97 - 1.37 | 0.10 |
| 295 | Schizophrenia and other psychotic disorders | mental disorders | 116.65 | 0.57 | Fixed-effect model | 1.09 | 0.92 - 1.31 | 0.32 |
| 295.1 | Schizophrenia | mental disorders | 98.58 | 0.92 | Fixed-effect model | 1.01 | 0.81 - 1.25 | 0.92 |
| 296 | Mood disorders | mental disorders | 135.45 | 0.16 | Fixed-effect model | 1.01 | 0.97 - 1.06 | 0.60 |
| 296.1 | Bipolar | mental disorders | 133.68 | 0.19 | Fixed-effect model | 0.95 | 0.81 - 1.12 | 0.54 |
| 296.2 | Depression | mental disorders | 140.22 | 0.10 | Fixed-effect model | 1.02 | 0.97 - 1.07 | 0.38 |
| 300 | Anxiety disorders | mental disorders | 165.55 | 0.00 | Random effect model | 1.04 | 0.97 - 1.12 | 0.30 |
| 300.1 | Anxiety disorder | mental disorders | 159.53 | 0.01 | Random effect model | 1.03 | 0.95 - 1.11 | 0.50 |
| 300.12 | Agorophobia, social phobia, and panic disorder | mental disorders | 123.28 | 0.40 | Fixed-effect model | 1.04 | 0.86 - 1.26 | 0.70 |
| 300.13 | Phobia | mental disorders | 112.32 | 0.68 | Fixed-effect model | 1.47 | 1.17 - 1.84 | 1.05E-03 |
| 303 | Psychogenic and somatoform disorders | mental disorders | 118.90 | 0.51 | Fixed-effect model | 0.90 | 0.72 - 1.12 | 0.35 |
| 306 | Other mental disorder | mental disorders | 160.51 | 0.01 | Random effect model | 1.02 | 0.98 - 1.06 | 0.31 |
| 317 | Alcohol-related disorders | mental disorders | 162.20 | 0.01 | Random effect model | 1.00 | 0.95 - 1.06 | 0.99 |
| 317.1 | Alcoholism | mental disorders | 167.24 | 0.00 | Random effect model | 1.01 | 0.94 - 1.07 | 0.85 |
| 317.11 | Alcoholic liver damage | mental disorders | 147.15 | 0.05 | Random effect model | 1.10 | 0.90 - 1.34 | 0.38 |
| 318 | Tobacco use disorder | mental disorders | 154.06 | 0.02 | Random effect model | 0.95 | 0.91 - 0.99 | 0.03 |
| 327 | Sleep disorders | neurological | 111.08 | 0.71 | Fixed-effect model | 0.95 | 0.88 - 1.02 | 0.13 |
| 327.3 | Sleep apnea | neurological | 112.19 | 0.68 | Fixed-effect model | 0.97 | 0.90 - 1.05 | 0.44 |
| 331 | Other cerebral degenerations | neurological | 148.56 | 0.04 | Random effect model | 1.48 | 1.25 - 1.76 | 6.79E-06 |
| 332 | Parkinson's disease | neurological | 112.86 | 0.67 | Fixed-effect model | 0.98 | 0.84 - 1.15 | 0.83 |
| 333 | Extrapyramidal disease and abnormal movement disorders | neurological | 141.09 | 0.09 | Fixed-effect model | 1.00 | 0.84 - 1.19 | 0.97 |
| 334 | Degenerative disease of the spinal cord | neurological | 121.94 | 0.43 | Fixed-effect model | 0.99 | 0.87 - 1.12 | 0.85 |
| 335 | Multiple sclerosis | neurological | 166.23 | 0.00 | Random effect model | 0.90 | 0.76 - 1.06 | 0.22 |
| 338 | Pain | symptoms | 118.01 | 0.53 | Fixed-effect model | 0.84 | 0.70 - 1.01 | 0.07 |
| 339 | Other headache syndromes | neurological | 134.37 | 0.17 | Fixed-effect model | 0.97 | 0.92 - 1.03 | 0.33 |
| 340 | Migraine | neurological | 185.69 | 0.00 | Random effect model | 1.03 | 0.91 - 1.16 | 0.66 |
| 342 | Hemiplegia | neurological | 149.86 | 0.03 | Random effect model | 1.05 | 0.90 - 1.21 | 0.57 |
| 344 | Other paralytic syndromes | neurological | 98.79 | 0.92 | Fixed-effect model | 1.00 | 0.82 - 1.22 | 0.98 |
| 345 | Epilepsy, recurrent seizures, convulsions | neurological | 148.49 | 0.04 | Random effect model | 1.05 | 0.96 - 1.13 | 0.28 |
| 345.1 | Epilepsy | neurological | 115.84 | 0.59 | Fixed-effect model | 1.13 | 0.95 - 1.34 | 0.17 |
| 345.3 | Convulsions | neurological | 153.47 | 0.02 | Random effect model | 1.15 | 1.01 - 1.30 | 0.03 |
| 348 | Other conditions of brain | neurological | 105.89 | 0.82 | Fixed-effect model | 1.04 | 0.91 - 1.20 | 0.54 |
| 350 | Abnormal movement | neurological | 132.23 | 0.21 | Fixed-effect model | 0.99 | 0.89 - 1.09 | 0.79 |
| 350.1 | Abnormal involuntary movements | neurological | 110.09 | 0.73 | Fixed-effect model | 1.09 | 0.91 - 1.30 | 0.34 |
| 350.2 | Abnormality of gait | neurological | 128.72 | 0.28 | Fixed-effect model | 0.96 | 0.85 - 1.10 | 0.58 |
| 351 | Other peripheral nerve disorders | neurological | 118.08 | 0.53 | Fixed-effect model | 0.97 | 0.92 - 1.01 | 0.16 |
| 352 | Disorders of other cranial nerves | neurological | 130.10 | 0.25 | Fixed-effect model | 1.04 | 0.91 - 1.20 | 0.57 |
| 352.2 | Facial nerve disorders [CN7] | neurological | 113.90 | 0.64 | Fixed-effect model | 1.00 | 0.84 - 1.19 | 0.98 |
| 353 | Nerve root and plexus disorders | neurological | 117.26 | 0.55 | Fixed-effect model | 0.85 | 0.73 - 0.99 | 0.04 |
| 357 | Inflammatory and toxic neuropathy | neurological | 105.17 | 0.83 | Fixed-effect model | 1.03 | 0.89 - 1.18 | 0.69 |
| 361 | Retinal detachments and defects | sense organs | 139.94 | 0.10 | Fixed-effect model | 1.09 | 0.99 - 1.19 | 0.06 |
| 361.1 | Retinal detachment with retinal defect | sense organs | 105.00 | 0.83 | Fixed-effect model | 1.17 | 1.02 - 1.34 | 0.03 |
| 362 | Other retinal disorders | sense organs | 107.95 | 0.78 | Fixed-effect model | 1.03 | 0.95 - 1.12 | 0.50 |
| 362.2 | Degeneration of macula and posterior pole of retina | sense organs | 128.32 | 0.29 | Fixed-effect model | 1.04 | 0.93 - 1.16 | 0.52 |
| 362.29 | Macular degeneration (senile) of retina NOS | sense organs | 128.73 | 0.28 | Fixed-effect model | 1.03 | 0.92 - 1.15 | 0.57 |
| 362.4 | Retinal vascular changes and abnomalities | sense organs | 101.32 | 0.89 | Fixed-effect model | 0.91 | 0.76 - 1.08 | 0.27 |
| 364 | Corneal opacity and other disorders of cornea | sense organs | 120.88 | 0.46 | Fixed-effect model | 1.02 | 0.84 - 1.24 | 0.83 |
| 365 | Glaucoma | sense organs | 166.99 | 0.00 | Random effect model | 0.98 | 0.89 - 1.08 | 0.69 |
| 365.1 | Open-angle glaucoma | sense organs | 104.92 | 0.83 | Fixed-effect model | 0.87 | 0.74 - 1.03 | 0.10 |
| 365.11 | Primary open angle glaucoma | sense organs | 102.19 | 0.88 | Fixed-effect model | 0.87 | 0.74 - 1.02 | 0.09 |
| 365.2 | Primary angle-closure glaucoma | sense organs | 113.45 | 0.65 | Fixed-effect model | 1.10 | 0.91 - 1.34 | 0.33 |
| 366 | Cataract | sense organs | 134.78 | 0.17 | Fixed-effect model | 1.08 | 1.03 - 1.12 | 2.17E-04 |
| 366.2 | Senile cataract | sense organs | 122.86 | 0.41 | Fixed-effect model | 1.05 | 0.99 - 1.11 | 0.10 |
| 367 | Disorders of refraction and accommodation; blindness and low vision | sense organs | 103.65 | 0.86 | Fixed-effect model | 1.05 | 0.94 - 1.16 | 0.40 |
| 367.1 | Myopia | sense organs | 99.23 | 0.92 | Fixed-effect model | 0.93 | 0.80 - 1.07 | 0.30 |
| 367.9 | Blindness and low vision | sense organs | 127.07 | 0.31 | Fixed-effect model | 1.17 | 0.97 - 1.42 | 0.10 |
| 368 | Visual disturbances | sense organs | 133.51 | 0.19 | Fixed-effect model | 0.92 | 0.84 - 1.01 | 0.07 |
| 368.1 | Amblyopia | sense organs | 105.04 | 0.83 | Fixed-effect model | 1.07 | 0.86 - 1.34 | 0.55 |
| 368.2 | Diplopia and disorders of binocular vision | sense organs | 117.21 | 0.56 | Fixed-effect model | 0.69 | 0.57 - 0.84 | 1.58E-04 |
| 368.9 | Subjective visual disturbances | sense organs | 116.81 | 0.57 | Fixed-effect model | 0.98 | 0.80 - 1.21 | 0.87 |
| 369 | Infection of the eye | sense organs | 141.88 | 0.08 | Fixed-effect model | 1.06 | 0.85 - 1.32 | 0.60 |
| 371 | Inflammation of the eye | sense organs | 128.53 | 0.28 | Fixed-effect model | 1.00 | 0.92 - 1.10 | 0.95 |
| 371.3 | Inflammation of eyelids | sense organs | 107.16 | 0.79 | Fixed-effect model | 1.10 | 0.99 - 1.23 | 0.07 |
| 372 | Disorders of conjunctiva | sense organs | 112.31 | 0.68 | Fixed-effect model | 0.80 | 0.66 - 0.97 | 0.02 |
| 374 | Other disorders of eyelids | sense organs | 118.70 | 0.52 | Fixed-effect model | 1.01 | 0.94 - 1.08 | 0.79 |
| 374.1 | Ectropion or entropion | sense organs | 162.02 | 0.01 | Random effect model | 0.99 | 0.83 - 1.20 | 0.95 |
| 375 | Disorders of lacrimal system | sense organs | 91.78 | 0.97 | Fixed-effect model | 0.92 | 0.83 - 1.03 | 0.16 |
| 375.2 | Epiphora | sense organs | 115.97 | 0.59 | Fixed-effect model | 0.69 | 0.58 - 0.82 | 3.27E-05 |
| 378 | Strabismus and other disorders of binocular eye movements | sense organs | 133.89 | 0.18 | Fixed-effect model | 0.87 | 0.76 - 1.00 | 0.05 |
| 378.1 | Strabismus (not specified as paralytic) | sense organs | 127.39 | 0.30 | Fixed-effect model | 0.92 | 0.78 - 1.09 | 0.35 |
| 379 | Other disorders of eye | sense organs | 131.20 | 0.23 | Fixed-effect model | 0.95 | 0.87 - 1.03 | 0.21 |
| 379.2 | Disorders of vitreous body | sense organs | 165.25 | 0.00 | Random effect model | 0.96 | 0.82 - 1.14 | 0.66 |
| 379.3 | Aphakia and other disorders of lens | sense organs | 93.74 | 0.96 | Fixed-effect model | 0.93 | 0.83 - 1.06 | 0.28 |
| 380 | Disorders of external ear | sense organs | 137.78 | 0.13 | Fixed-effect model | 1.09 | 0.96 - 1.25 | 0.19 |
| 380.1 | Otitis externa | sense organs | 142.42 | 0.08 | Fixed-effect model | 1.28 | 1.02 - 1.60 | 0.03 |
| 381 | Otitis media and Eustachian tube disorders | sense organs | 127.92 | 0.29 | Fixed-effect model | 0.86 | 0.77 - 0.96 | 0.01 |
| 381.1 | Otitis media | sense organs | 121.43 | 0.45 | Fixed-effect model | 0.86 | 0.76 - 0.97 | 0.02 |
| 381.11 | Suppurative and unspecified otitis media | sense organs | 114.73 | 0.62 | Fixed-effect model | 0.88 | 0.74 - 1.05 | 0.15 |
| 384 | Other disorders of tympanic membrane | sense organs | 118.54 | 0.52 | Fixed-effect model | 0.85 | 0.74 - 0.98 | 0.02 |
| 384.4 | Perforation of tympanic membrane | sense organs | 124.99 | 0.36 | Fixed-effect model | 0.87 | 0.74 - 1.02 | 0.09 |
| 385 | Other disorders of middle ear and mastoid | sense organs | 119.58 | 0.49 | Fixed-effect model | 0.91 | 0.76 - 1.09 | 0.33 |
| 385.3 | Cholesteatoma | sense organs | 108.91 | 0.76 | Fixed-effect model | 0.74 | 0.60 - 0.92 | 0.01 |
| 386 | Vertiginous syndromes and other disorders of vestibular system | sense organs | 141.04 | 0.09 | Fixed-effect model | 0.94 | 0.88 - 1.00 | 0.05 |
| 386.1 | Meniere's disease | sense organs | 124.41 | 0.37 | Fixed-effect model | 0.90 | 0.73 - 1.12 | 0.34 |
| 386.3 | Labyrinthitis | sense organs | 99.47 | 0.91 | Fixed-effect model | 0.88 | 0.73 - 1.06 | 0.17 |
| 386.9 | Dizziness and giddiness (Light-headedness and vertigo) | sense organs | 129.50 | 0.26 | Fixed-effect model | 0.96 | 0.89 - 1.04 | 0.29 |
| 389 | Hearing loss | sense organs | 127.17 | 0.31 | Fixed-effect model | 1.02 | 0.94 - 1.11 | 0.59 |
| 389.4 | Tinnitus | sense organs | 123.76 | 0.39 | Fixed-effect model | 1.02 | 0.81 - 1.28 | 0.87 |
| 394 | Rheumatic disease of the heart valves | circulatory system | 155.08 | 0.02 | Random effect model | 1.04 | 0.96 - 1.14 | 0.31 |
| 394.2 | Mitral valve disease | circulatory system | 142.08 | 0.08 | Fixed-effect model | 1.05 | 0.95 - 1.15 | 0.35 |
| 394.3 | Aortic valve disease | circulatory system | 137.15 | 0.14 | Fixed-effect model | 1.02 | 0.88 - 1.18 | 0.83 |
| 394.7 | Disease of tricuspid valve | circulatory system | 87.09 | 0.99 | Fixed-effect model | 1.19 | 1.01 - 1.39 | 0.03 |
| 395 | Heart valve disorders | circulatory system | 144.43 | 0.06 | Fixed-effect model | 1.02 | 0.94 - 1.11 | 0.57 |
| 395.1 | Nonrheumatic mitral valve disorders | circulatory system | 138.80 | 0.12 | Fixed-effect model | 1.08 | 0.98 - 1.19 | 0.14 |
| 395.6 | Heart valve replaced | circulatory system | 135.75 | 0.15 | Fixed-effect model | 0.93 | 0.81 - 1.06 | 0.28 |
| 396 | Abnormal heart sounds | circulatory system | 146.22 | 0.05 | Fixed-effect model | 0.96 | 0.82 - 1.13 | 0.64 |
| 401 | Hypertension | circulatory system | 436.17 | 0.00 | Random effect model | 1.04 | 0.99 - 1.08 | 0.11 |
| 401.1 | Essential hypertension | circulatory system | 436.37 | 0.00 | Random effect model | 1.04 | 0.99 - 1.08 | 0.11 |
| 401.2 | Hypertensive heart and/or renal disease | circulatory system | 132.55 | 0.20 | Fixed-effect model | 1.00 | 0.88 - 1.13 | 0.97 |
| 401.22 | Hypertensive chronic kidney disease | circulatory system | 139.19 | 0.11 | Fixed-effect model | 0.99 | 0.87 - 1.13 | 0.91 |
| 402 | Elevated blood pressure reading without diagnosis of hypertension | circulatory system | 124.28 | 0.38 | Fixed-effect model | 1.10 | 0.97 - 1.26 | 0.15 |
| 411 | Ischemic Heart Disease | circulatory system | 340.08 | 6.26E-23 | Random effect model | 1.46 | 1.38 - 1.55 | 7.82E-42 |
| 411.1 | Unstable angina (intermediate coronary syndrome) | circulatory system | 168.01 | 2.53E-03 | Random effect model | 1.50 | 1.37 - 1.63 | 5.29E-20 |
| 411.2 | Myocardial infarction | circulatory system | 256.65 | 6.03E-12 | Random effect model | 1.58 | 1.47 - 1.70 | 1.17E-33 |
| 411.3 | Angina pectoris | circulatory system | 254.88 | 9.76E-12 | Random effect model | 1.52 | 1.43 - 1.62 | 2.41E-38 |
| 411.4 | Coronary atherosclerosis | circulatory system | 395.98 | 3.36E-31 | Random effect model | 1.65 | 1.53 - 1.77 | 4.25E-41 |
| 411.41 | Aneurysm and dissection of heart | circulatory system | 121.50 | 0.44 | Fixed-effect model | 1.16 | 0.95 - 1.41 | 0.14 |
| 411.8 | Other chronic ischemic heart disease, unspecified | circulatory system | 231.59 | 4.25E-09 | Random effect model | 1.52 | 1.43 - 1.62 | 6.87E-39 |
| 411.9 | Other acute and subacute forms of ischemic heart disease | circulatory system | 132.71 | 0.20 | Fixed-effect model | 1.32 | 1.13 - 1.53 | 3.80E-04 |
| 414 | Other forms of chronic heart disease | circulatory system | 111.65 | 0.69 | Fixed-effect model | 1.31 | 1.16 - 1.48 | 1.25E-05 |
| 415 | Pulmonary heart disease | circulatory system | 121.57 | 0.44 | Fixed-effect model | 0.88 | 0.81 - 0.95 | 1.96E-03 |
| 415.2 | Chronic pulmonary heart disease | circulatory system | 105.51 | 0.82 | Fixed-effect model | 0.90 | 0.73 - 1.12 | 0.35 |
| 416 | Cardiomegaly | circulatory system | 117.53 | 0.55 | Fixed-effect model | 1.04 | 0.94 - 1.15 | 0.45 |
| 418 | Nonspecific chest pain | circulatory system | 150.39 | 0.03 | Random effect model | 1.10 | 1.07 - 1.14 | 3.82E-08 |
| 418.1 | Precordial pain | circulatory system | 122.51 | 0.42 | Fixed-effect model | 1.01 | 0.93 - 1.10 | 0.83 |
| 420.2 | Pericarditis | circulatory system | 100.88 | 0.90 | Fixed-effect model | 1.11 | 0.96 - 1.28 | 0.17 |
| 420.3 | Endocarditis | circulatory system | 154.91 | 0.02 | Random effect model | 0.99 | 0.79 - 1.24 | 0.93 |
| 425 | Cardiomyopathy | circulatory system | 120.40 | 0.47 | Fixed-effect model | 0.82 | 0.71 - 0.95 | 0.01 |
| 425.1 | Primary/intrinsic cardiomyopathies | circulatory system | 116.10 | 0.58 | Fixed-effect model | 0.81 | 0.70 - 0.94 | 0.01 |
| 426 | Cardiac conduction disorders | circulatory system | 152.34 | 0.02 | Random effect model | 1.11 | 1.03 - 1.19 | 0.01 |
| 426.2 | Atrioventricular [AV] block | circulatory system | 125.72 | 0.34 | Fixed-effect model | 1.12 | 1.00 - 1.25 | 0.05 |
| 426.21 | First degree AV block | circulatory system | 145.60 | 0.06 | Fixed-effect model | 1.27 | 1.07 - 1.49 | 4.83E-03 |
| 426.24 | Atrioventricular block, complete | circulatory system | 105.36 | 0.83 | Fixed-effect model | 0.95 | 0.77 - 1.18 | 0.67 |
| 426.3 | Bundle branch block | circulatory system | 177.20 | 0.00 | Random effect model | 1.06 | 0.95 - 1.19 | 0.27 |
| 426.31 | Right bundle branch block | circulatory system | 160.16 | 0.01 | Random effect model | 1.10 | 0.94 - 1.28 | 0.24 |
| 426.32 | Left bundle branch block | circulatory system | 141.07 | 0.09 | Fixed-effect model | 1.03 | 0.91 - 1.16 | 0.69 |
| 426.9 | Cardiac pacemaker/device in situ | circulatory system | 136.23 | 0.15 | Fixed-effect model | 1.17 | 1.06 - 1.30 | 2.77E-03 |
| 426.91 | Cardiac pacemaker in situ | circulatory system | 129.26 | 0.27 | Fixed-effect model | 1.21 | 1.09 - 1.35 | 5.03E-04 |
| 427 | Cardiac dysrhythmias | circulatory system | 118.83 | 0.51 | Fixed-effect model | 1.07 | 1.03 - 1.11 | 1.36E-04 |
| 427.1 | Paroxysmal tachycardia, unspecified | circulatory system | 110.36 | 0.72 | Fixed-effect model | 1.08 | 0.99 - 1.19 | 0.10 |
| 427.11 | Paroxysmal supraventricular tachycardia | circulatory system | 122.93 | 0.41 | Fixed-effect model | 1.02 | 0.92 - 1.14 | 0.69 |
| 427.12 | Paroxysmal ventricular tachycardia | circulatory system | 100.44 | 0.90 | Fixed-effect model | 1.23 | 1.04 - 1.46 | 0.01 |
| 427.2 | Atrial fibrillation and flutter | circulatory system | 136.24 | 0.15 | Fixed-effect model | 1.10 | 1.05 - 1.15 | 6.00E-05 |
| 427.3 | Other specified cardiac dysrhythmias | circulatory system | 139.98 | 0.10 | Fixed-effect model | 1.22 | 1.12 - 1.34 | 1.54E-05 |
| 427.4 | Cardiac arrest and ventricular fibrillation | circulatory system | 132.84 | 0.20 | Fixed-effect model | 1.22 | 1.05 - 1.42 | 0.01 |
| 427.42 | Cardiac arrest | circulatory system | 135.06 | 0.16 | Fixed-effect model | 1.17 | 0.99 - 1.39 | 0.06 |
| 427.5 | Arrhythmia (cardiac) NOS | circulatory system | 117.25 | 0.55 | Fixed-effect model | 0.90 | 0.76 - 1.06 | 0.21 |
| 427.6 | Premature beats | circulatory system | 121.70 | 0.44 | Fixed-effect model | 1.16 | 0.93 - 1.46 | 0.18 |
| 427.7 | Tachycardia NOS | circulatory system | 144.14 | 0.07 | Fixed-effect model | 0.86 | 0.77 - 0.96 | 0.01 |
| 427.9 | Palpitations | circulatory system | 138.18 | 0.12 | Fixed-effect model | 1.10 | 1.01 - 1.19 | 0.03 |
| 428 | Congestive heart failure; nonhypertensive | circulatory system | 199.55 | 0.00 | Random effect model | 1.11 | 1.01 - 1.22 | 0.03 |
| 428.2 | Heart failure NOS | circulatory system | 204.05 | 2.66E-06 | Random effect model | 1.18 | 1.07 - 1.31 | 1.58E-03 |
| 429 | Ill-defined descriptions and complications of heart disease | circulatory system | 95.04 | 0.95 | Fixed-effect model | 1.36 | 1.14 - 1.63 | 8.62E-04 |
| 429.2 | Abnormal function study of cardiovascular system | circulatory system | 115.86 | 0.59 | Fixed-effect model | 1.32 | 1.05 - 1.65 | 0.02 |
| 430 | Intracranial hemorrhage | circulatory system | 132.91 | 0.20 | Fixed-effect model | 0.93 | 0.82 - 1.05 | 0.24 |
| 430.1 | Subarachnoid hemorrhage | circulatory system | 123.01 | 0.41 | Fixed-effect model | 0.96 | 0.80 - 1.15 | 0.66 |
| 430.2 | Intracerebral hemorrhage | circulatory system | 99.09 | 0.92 | Fixed-effect model | 0.95 | 0.78 - 1.15 | 0.60 |
| 433 | Cerebrovascular disease | circulatory system | 148.71 | 0.04 | Random effect model | 1.17 | 1.10 - 1.24 | 1.33E-06 |
| 433.1 | Occlusion and stenosis of precerebral arteries | circulatory system | 146.84 | 0.05 | Random effect model | 1.33 | 1.13 - 1.57 | 7.19E-04 |
| 433.2 | Occlusion of cerebral arteries | circulatory system | 119.23 | 0.50 | Fixed-effect model | 1.08 | 0.99 - 1.17 | 0.07 |
| 433.21 | Cerebral artery occlusion, with cerebral infarction | circulatory system | 104.13 | 0.85 | Fixed-effect model | 0.82 | 0.71 - 0.94 | 3.55E-03 |
| 433.3 | Cerebral ischemia | circulatory system | 154.80 | 0.02 | Random effect model | 1.19 | 1.07 - 1.33 | 1.67E-03 |
| 433.31 | Transient cerebral ischemia | circulatory system | 149.38 | 0.04 | Random effect model | 1.31 | 1.15 - 1.48 | 2.58E-05 |
| 433.8 | Late effects of cerebrovascular disease | circulatory system | 147.92 | 0.04 | Random effect model | 0.95 | 0.81 - 1.12 | 0.57 |
| 440 | Atherosclerosis | circulatory system | 154.61 | 0.02 | Random effect model | 1.28 | 1.09 - 1.51 | 2.51E-03 |
| 440.2 | Atherosclerosis of the extremities | circulatory system | 167.79 | 0.00 | Random effect model | 1.17 | 0.94 - 1.45 | 0.15 |
| 441 | Vascular insufficiency of intestine | circulatory system | 102.36 | 0.88 | Fixed-effect model | 0.71 | 0.58 - 0.88 | 2.05E-03 |
| 442 | Other aneurysm | circulatory system | 174.37 | 0.00 | Random effect model | 1.12 | 0.96 - 1.29 | 0.14 |
| 442.1 | Aortic aneurysm | circulatory system | 162.11 | 0.01 | Random effect model | 1.27 | 1.08 - 1.49 | 4.13E-03 |
| 442.11 | Abdominal aortic aneurysm | circulatory system | 192.49 | 2.98E-05 | Random effect model | 1.50 | 1.20 - 1.87 | 3.24E-04 |
| 443 | Peripheral vascular disease | circulatory system | 184.68 | 1.37E-04 | Random effect model | 1.23 | 1.11 - 1.36 | 1.08E-04 |
| 443.1 | Raynaud's syndrome | circulatory system | 125.51 | 0.35 | Fixed-effect model | 1.07 | 0.92 - 1.24 | 0.41 |
| 443.9 | Peripheral vascular disease, unspecified | circulatory system | 202.84 | 3.46E-06 | Random effect model | 1.32 | 1.15 - 1.50 | 5.81E-05 |
| 444 | Arterial embolism and thrombosis | circulatory system | 160.02 | 0.01 | Random effect model | 1.11 | 0.91 - 1.35 | 0.30 |
| 444.1 | Arterial embolism and thrombosis of lower extremity artery | circulatory system | 145.59 | 0.06 | Fixed-effect model | 0.98 | 0.79 - 1.22 | 0.85 |
| 446 | Polyarteritis nodosa and allied conditions | circulatory system | 130.22 | 0.25 | Fixed-effect model | 1.33 | 1.11 - 1.59 | 1.96E-03 |
| 447 | Other disorders of arteries and arterioles | circulatory system | 97.75 | 0.93 | Fixed-effect model | 1.06 | 0.92 - 1.22 | 0.41 |
| 447.1 | Stricture of artery | circulatory system | 110.70 | 0.72 | Fixed-effect model | 1.09 | 0.91 - 1.29 | 0.36 |
| 450 | Noninfectious disorders of lymphatic channels | circulatory system | 103.73 | 0.86 | Fixed-effect model | 1.07 | 0.88 - 1.30 | 0.47 |
| 451 | Phlebitis and thrombophlebitis | circulatory system | 182.51 | 0.00 | Random effect model | 0.88 | 0.79 - 0.98 | 0.02 |
| 451.2 | Phlebitis and thrombophlebitis of lower extremities | circulatory system | 179.24 | 3.73E-04 | Random effect model | 0.85 | 0.77 - 0.95 | 3.31E-03 |
| 452 | Other venous embolism and thrombosis | circulatory system | 136.93 | 0.14 | Fixed-effect model | 0.67 | 0.54 - 0.83 | 3.38E-04 |
| 454 | Varicose veins | circulatory system | 299.43 | 0.00 | Random effect model | 0.95 | 0.88 - 1.03 | 0.21 |
| 454.1 | Varicose veins of lower extremity | circulatory system | 278.97 | 0.00 | Random effect model | 0.96 | 0.89 - 1.04 | 0.36 |
| 454.11 | Varicose veins of lower extremity, symptomtic | circulatory system | 90.10 | 0.98 | Fixed-effect model | 0.84 | 0.69 - 1.04 | 0.10 |
| 455 | Hemorrhoids | circulatory system | 158.73 | 0.01 | Random effect model | 1.02 | 0.98 - 1.06 | 0.31 |
| 458 | Hypotension | circulatory system | 100.62 | 0.90 | Fixed-effect model | 1.09 | 1.02 - 1.17 | 0.01 |
| 458.1 | Orthostatic hypotension | circulatory system | 121.60 | 0.44 | Fixed-effect model | 1.33 | 1.16 - 1.54 | 5.92E-05 |
| 458.9 | Hypotension NOS | circulatory system | 102.23 | 0.88 | Fixed-effect model | 1.01 | 0.93 - 1.11 | 0.75 |
| 459 | Other disorders of circulatory system | circulatory system | 213.76 | 0.00 | Random effect model | 1.05 | 0.99 - 1.11 | 0.09 |
| 459.9 | Circulatory disease NEC | circulatory system | 208.07 | 0.00 | Random effect model | 1.05 | 0.99 - 1.11 | 0.10 |
| 465 | Acute upper respiratory infections of multiple or unspecified sites | respiratory | 96.83 | 0.94 | Fixed-effect model | 1.09 | 0.98 - 1.21 | 0.12 |
| 465.2 | Acute pharyngitis | respiratory | 106.82 | 0.80 | Fixed-effect model | 1.19 | 0.99 - 1.41 | 0.06 |
| 470 | Septal Deviations/Turbinate Hypertrophy | respiratory | 124.80 | 0.36 | Fixed-effect model | 1.02 | 0.95 - 1.10 | 0.62 |
| 471 | Nasal polyps | respiratory | 145.65 | 0.06 | Fixed-effect model | 1.14 | 1.04 - 1.25 | 4.83E-03 |
| 472 | Chronic pharyngitis and nasopharyngitis | respiratory | 110.29 | 0.73 | Fixed-effect model | 1.10 | 0.94 - 1.30 | 0.24 |
| 473 | Diseases of the larynx and vocal cords | respiratory | 140.99 | 0.09 | Fixed-effect model | 0.98 | 0.88 - 1.08 | 0.67 |
| 473.4 | Voice disturbance | respiratory | 148.89 | 0.04 | Random effect model | 0.97 | 0.82 - 1.15 | 0.72 |
| 474.1 | Acute tonsillitis | respiratory | 118.30 | 0.53 | Fixed-effect model | 1.09 | 0.88 - 1.34 | 0.44 |
| 474.2 | Chronic tonsillitis and adenoiditis | respiratory | 126.95 | 0.31 | Fixed-effect model | 0.91 | 0.78 - 1.06 | 0.21 |
| 475 | Chronic sinusitis | respiratory | 125.18 | 0.35 | Fixed-effect model | 0.95 | 0.86 - 1.05 | 0.32 |
| 476 | Allergic rhinitis | respiratory | 109.42 | 0.75 | Fixed-effect model | 1.09 | 0.93 - 1.28 | 0.29 |
| 477 | Epistaxis or throat hemorrhage | respiratory | 122.51 | 0.42 | Fixed-effect model | 1.00 | 0.90 - 1.11 | 0.99 |
| 479 | Other upper respiratory disease | respiratory | 133.60 | 0.19 | Fixed-effect model | 0.99 | 0.91 - 1.07 | 0.82 |
| 480 | Pneumonia | respiratory | 151.19 | 0.03 | Random effect model | 1.07 | 1.01 - 1.14 | 0.02 |
| 480.1 | Bacterial pneumonia | respiratory | 155.28 | 0.02 | Random effect model | 1.05 | 0.98 - 1.13 | 0.17 |
| 480.11 | Pneumococcal pneumonia | respiratory | 152.78 | 0.02 | Random effect model | 1.04 | 0.96 - 1.13 | 0.30 |
| 495 | Asthma | respiratory | 272.63 | 0.00 | Random effect model | 0.98 | 0.93 - 1.03 | 0.48 |
| 496 | Chronic airway obstruction | respiratory | 158.70 | 0.01 | Random effect model | 0.91 | 0.85 - 0.96 | 1.65E-03 |
| 496.1 | Emphysema | respiratory | 140.44 | 0.10 | Fixed-effect model | 0.97 | 0.85 - 1.10 | 0.61 |
| 496.2 | Chronic bronchitis | respiratory | 154.30 | 0.02 | Random effect model | 0.92 | 0.83 - 1.03 | 0.16 |
| 496.21 | Obstructive chronic bronchitis | respiratory | 165.25 | 0.00 | Random effect model | 0.95 | 0.85 - 1.07 | 0.43 |
| 497 | Bronchitis | respiratory | 133.13 | 0.19 | Fixed-effect model | 0.83 | 0.67 - 1.01 | 0.07 |
| 501 | Pneumonitis due to inhalation of food or vomitus | respiratory | 126.79 | 0.32 | Fixed-effect model | 1.64 | 1.32 - 2.03 | 5.69E-06 |
| 502 | Postinflammatory pulmonary fibrosis | respiratory | 107.02 | 0.80 | Fixed-effect model | 0.98 | 0.83 - 1.17 | 0.85 |
| 506 | Empyema and pneumothorax | respiratory | 137.79 | 0.13 | Fixed-effect model | 1.10 | 0.95 - 1.28 | 0.21 |
| 507 | Pleurisy; pleural effusion | respiratory | 140.78 | 0.09 | Fixed-effect model | 1.06 | 0.99 - 1.13 | 0.10 |
| 509 | Respiratory failure, insufficiency, arrest | respiratory | 124.40 | 0.37 | Fixed-effect model | 1.09 | 0.98 - 1.21 | 0.11 |
| 509.2 | Respiratory insufficiency | respiratory | 125.73 | 0.34 | Fixed-effect model | 1.05 | 0.93 - 1.19 | 0.44 |
| 510 | Other diseases of lung | respiratory | 144.82 | 0.06 | Fixed-effect model | 0.91 | 0.75 - 1.09 | 0.30 |
| 512 | Other symptoms of respiratory system | respiratory | 111.08 | 0.71 | Fixed-effect model | 1.05 | 1.00 - 1.11 | 0.07 |
| 512.7 | Shortness of breath | respiratory | 95.80 | 0.95 | Fixed-effect model | 1.06 | 0.99 - 1.13 | 0.12 |
| 512.8 | Cough | respiratory | 138.73 | 0.12 | Fixed-effect model | 1.06 | 0.96 - 1.17 | 0.26 |
| 512.9 | Other dyspnea | respiratory | 85.83 | 0.99 | Fixed-effect model | 0.96 | 0.81 - 1.15 | 0.68 |
| 513 | Respiratory abnormalities | respiratory | 157.48 | 0.01 | Random effect model | 0.77 | 0.61 - 0.98 | 0.04 |
| 514 | Abnormal findings examination of lungs | respiratory | 102.24 | 0.88 | Fixed-effect model | 1.07 | 0.97 - 1.19 | 0.20 |
| 516 | Abnormal sputum | respiratory | 125.60 | 0.34 | Fixed-effect model | 0.88 | 0.79 - 0.99 | 0.03 |
| 519 | Other diseases of respiratory system, not elsewhere classified | respiratory | 132.78 | 0.20 | Fixed-effect model | 1.02 | 0.96 - 1.07 | 0.52 |
| 519.8 | Other diseases of respiratory system, NEC | respiratory | 123.50 | 0.39 | Fixed-effect model | 1.02 | 0.97 - 1.08 | 0.43 |
| 520 | Disorders of tooth development | digestive | 100.22 | 0.91 | Fixed-effect model | 1.08 | 0.97 - 1.20 | 0.14 |
| 520.2 | Disturbances in tooth eruption | digestive | 99.09 | 0.92 | Fixed-effect model | 1.07 | 0.96 - 1.19 | 0.21 |
| 521 | Diseases of hard tissues of teeth | digestive | 90.43 | 0.98 | Fixed-effect model | 1.03 | 0.94 - 1.13 | 0.51 |
| 521.1 | Dental caries | digestive | 88.62 | 0.99 | Fixed-effect model | 1.02 | 0.93 - 1.12 | 0.65 |
| 522 | Diseases of pulp and periapical tissues | digestive | 108.28 | 0.77 | Fixed-effect model | 0.94 | 0.84 - 1.07 | 0.36 |
| 522.5 | Periapical abscess | digestive | 100.67 | 0.90 | Fixed-effect model | 1.06 | 0.91 - 1.24 | 0.43 |
| 523 | Gingival and periodontal diseases | digestive | 126.75 | 0.32 | Fixed-effect model | 0.90 | 0.79 - 1.01 | 0.08 |
| 523.3 | Periodontitis (acute or chronic) | digestive | 125.13 | 0.36 | Fixed-effect model | 0.92 | 0.79 - 1.06 | 0.25 |
| 523.31 | Acute periodontitis | digestive | 133.50 | 0.19 | Fixed-effect model | 0.98 | 0.81 - 1.20 | 0.87 |
| 523.32 | Chronic periodontitis | digestive | 117.11 | 0.56 | Fixed-effect model | 0.83 | 0.67 - 1.04 | 0.10 |
| 525 | Other diseases of the teeth and supporting structures | digestive | 108.26 | 0.77 | Fixed-effect model | 0.96 | 0.87 - 1.07 | 0.48 |
| 526 | Diseases of the jaws | digestive | 131.59 | 0.22 | Fixed-effect model | 0.99 | 0.84 - 1.17 | 0.89 |
| 527 | Diseases of the salivary glands | digestive | 121.30 | 0.45 | Fixed-effect model | 1.44 | 1.19 - 1.75 | 1.94E-04 |
| 528 | Diseases of the oral soft tissues, excluding lesions specific for gingiva and tongue | digestive | 126.90 | 0.32 | Fixed-effect model | 1.05 | 0.97 - 1.15 | 0.21 |
| 528.5 | Diseases of lips | digestive | 90.52 | 0.98 | Fixed-effect model | 1.13 | 0.93 - 1.38 | 0.22 |
| 529 | Diseases and other conditions of the tongue | digestive | 120.54 | 0.47 | Fixed-effect model | 1.15 | 0.99 - 1.33 | 0.07 |
| 530 | Diseases of esophagus | digestive | 172.21 | 0.00 | Random effect model | 0.97 | 0.93 - 1.00 | 0.05 |
| 530.1 | Esophagitis, GERD and related diseases | digestive | 168.03 | 0.00 | Random effect model | 0.95 | 0.92 - 0.99 | 0.01 |
| 530.11 | GERD | digestive | 154.96 | 0.02 | Random effect model | 1.00 | 0.95 - 1.05 | 0.85 |
| 530.12 | Ulcer of esophagus | digestive | 157.03 | 0.01 | Random effect model | 0.90 | 0.83 - 0.98 | 0.02 |
| 530.14 | Reflux esophagitis | digestive | 164.20 | 0.00 | Random effect model | 0.92 | 0.87 - 0.98 | 0.01 |
| 530.2 | Esophageal bleeding (varices/hemorrhage) | digestive | 107.54 | 0.79 | Fixed-effect model | 0.90 | 0.79 - 1.02 | 0.10 |
| 530.5 | Disorders of esophageal motility | digestive | 127.50 | 0.30 | Fixed-effect model | 0.83 | 0.68 - 1.02 | 0.08 |
| 530.9 | Heartburn | digestive | 114.12 | 0.63 | Fixed-effect model | 1.02 | 0.91 - 1.14 | 0.76 |
| 531 | Peptic ulcer (excl. esophageal) | digestive | 150.52 | 0.03 | Random effect model | 0.97 | 0.91 - 1.04 | 0.39 |
| 531.1 | Hemorrhage from gastrointestinal ulcer | digestive | 127.35 | 0.31 | Fixed-effect model | 1.06 | 0.86 - 1.31 | 0.57 |
| 531.2 | Gastric ulcer | digestive | 113.48 | 0.65 | Fixed-effect model | 0.94 | 0.87 - 1.02 | 0.14 |
| 531.3 | Duodenal ulcer | digestive | 143.31 | 0.07 | Fixed-effect model | 1.00 | 0.91 - 1.10 | 0.99 |
| 532 | Dysphagia | digestive | 123.71 | 0.39 | Fixed-effect model | 1.02 | 0.95 - 1.09 | 0.62 |
| 535 | Gastritis and duodenitis | digestive | 165.61 | 3.70E-03 | Random effect model | 0.94 | 0.90 - 0.97 | 9.31E-04 |
| 535.1 | Acute gastritis | digestive | 90.37 | 0.98 | Fixed-effect model | 0.89 | 0.76 - 1.03 | 0.12 |
| 535.6 | Duodenitis | digestive | 120.79 | 0.46 | Fixed-effect model | 0.91 | 0.85 - 0.96 | 1.76E-03 |
| 535.8 | Other specified gastritis | digestive | 103.45 | 0.86 | Fixed-effect model | 0.94 | 0.89 - 1.00 | 0.04 |
| 537 | Other disorders of stomach and duodenum | digestive | 121.42 | 0.45 | Fixed-effect model | 0.83 | 0.76 - 0.91 | 5.38E-05 |
| 540 | Appendiceal conditions | digestive | 106.19 | 0.81 | Fixed-effect model | 1.03 | 0.94 - 1.13 | 0.51 |
| 540.1 | Appendicitis | digestive | 101.81 | 0.88 | Fixed-effect model | 1.03 | 0.94 - 1.13 | 0.47 |
| 540.11 | Acute appendicitis | digestive | 112.32 | 0.68 | Fixed-effect model | 1.03 | 0.93 - 1.14 | 0.58 |
| 550 | Abdominal hernia | digestive | 133.56 | 0.19 | Fixed-effect model | 0.96 | 0.94 - 0.99 | 0.01 |
| 550.1 | Inguinal hernia | digestive | 221.09 | 0.00 | Random effect model | 1.05 | 0.99 - 1.11 | 0.11 |
| 550.2 | Diaphragmatic hernia | digestive | 163.20 | 0.01 | Random effect model | 0.91 | 0.88 - 0.95 | 5.40E-06 |
| 550.3 | Femoral hernia | digestive | 119.65 | 0.49 | Fixed-effect model | 1.23 | 1.01 - 1.51 | 0.04 |
| 550.4 | Umbilical hernia | digestive | 105.13 | 0.83 | Fixed-effect model | 0.92 | 0.85 - 1.01 | 0.07 |
| 550.5 | Ventral hernia | digestive | 105.46 | 0.83 | Fixed-effect model | 0.94 | 0.86 - 1.02 | 0.14 |
| 555 | Inflammatory bowel disease and other gastroenteritis and colitis | digestive | 162.95 | 0.01 | Random effect model | 1.03 | 0.94 - 1.13 | 0.52 |
| 555.1 | Regional enteritis | digestive | 98.45 | 0.93 | Fixed-effect model | 0.94 | 0.83 - 1.06 | 0.29 |
| 555.2 | Ulcerative colitis | digestive | 164.38 | 0.00 | Random effect model | 1.06 | 0.95 - 1.18 | 0.27 |
| 555.21 | Ulcerative colitis (chronic) | digestive | 143.05 | 0.07 | Fixed-effect model | 0.98 | 0.79 - 1.23 | 0.89 |
| 556 | Ulceration of the lower GI tract | digestive | 91.00 | 0.98 | Fixed-effect model | 1.08 | 0.92 - 1.27 | 0.33 |
| 556.1 | Ulceration of intestine | digestive | 101.36 | 0.89 | Fixed-effect model | 1.05 | 0.86 - 1.29 | 0.61 |
| 557 | Intestinal malabsorption (non-celiac) | digestive | 633.25 | 0.00 | Random effect model | 1.02 | 0.79 - 1.33 | 0.87 |
| 558 | Noninfectious gastroenteritis | digestive | 102.76 | 0.87 | Fixed-effect model | 0.99 | 0.95 - 1.04 | 0.80 |
| 559 | Ileostomy status | digestive | 104.77 | 0.84 | Fixed-effect model | 1.14 | 1.00 - 1.30 | 0.04 |
| 560 | Intestinal obstruction without mention of hernia | digestive | 106.95 | 0.80 | Fixed-effect model | 1.01 | 0.93 - 1.09 | 0.89 |
| 560.1 | Paralytic ileus | digestive | 123.74 | 0.39 | Fixed-effect model | 1.14 | 0.91 - 1.43 | 0.26 |
| 560.3 | Peritoneal or intestinal adhesions | digestive | 114.69 | 0.62 | Fixed-effect model | 0.98 | 0.82 - 1.17 | 0.79 |
| 560.4 | Other intestinal obstruction | digestive | 113.87 | 0.64 | Fixed-effect model | 0.97 | 0.88 - 1.06 | 0.44 |
| 561 | Symptoms involving digestive system | digestive | 111.65 | 0.69 | Fixed-effect model | 0.95 | 0.91 - 0.99 | 0.01 |
| 561.2 | Flatulence | digestive | 113.65 | 0.65 | Fixed-effect model | 0.88 | 0.77 - 1.00 | 0.04 |
| 562 | Diverticulosis and diverticulitis | digestive | 180.67 | 0.00 | Random effect model | 0.98 | 0.94 - 1.02 | 0.34 |
| 562.1 | Diverticulosis | digestive | 180.53 | 0.00 | Random effect model | 0.98 | 0.94 - 1.02 | 0.36 |
| 563 | Constipation | digestive | 122.69 | 0.41 | Fixed-effect model | 0.99 | 0.94 - 1.04 | 0.68 |
| 564 | Functional digestive disorders | digestive | 126.43 | 0.33 | Fixed-effect model | 0.96 | 0.92 - 0.99 | 0.02 |
| 564.1 | Irritable Bowel Syndrome | digestive | 135.48 | 0.16 | Fixed-effect model | 0.85 | 0.80 - 0.92 | 1.33E-05 |
| 564.8 | Abnormal findings on exam of gastrointestinal tract/ abdominal area | digestive | 125.27 | 0.35 | Fixed-effect model | 1.13 | 1.00 - 1.28 | 0.06 |
| 564.9 | Personal history of diseases of digestive system | digestive | 131.45 | 0.22 | Fixed-effect model | 0.97 | 0.93 - 1.02 | 0.21 |
| 565 | Anal and rectal conditions | digestive | 120.65 | 0.47 | Fixed-effect model | 1.13 | 1.08 - 1.18 | 1.59E-08 |
| 565.1 | Anal and rectal polyp | digestive | 117.83 | 0.54 | Fixed-effect model | 1.08 | 1.02 - 1.15 | 0.01 |
| 567 | Peritonitis and retroperitoneal infections | digestive | 105.38 | 0.83 | Fixed-effect model | 0.91 | 0.77 - 1.09 | 0.31 |
| 568 | Other disorders of peritoneum | digestive | 139.18 | 0.11 | Fixed-effect model | 0.89 | 0.81 - 0.97 | 0.01 |
| 568.1 | Peritoneal adhesions (postoperative) (postinfection) | digestive | 129.40 | 0.26 | Fixed-effect model | 0.89 | 0.81 - 0.98 | 0.01 |
| 569 | Other disorders of intestine | digestive | 113.84 | 0.64 | Fixed-effect model | 1.06 | 0.98 - 1.15 | 0.15 |
| 571 | Chronic liver disease and cirrhosis | digestive | 207.59 | 1.22E-06 | Random effect model | 0.81 | 0.71 - 0.92 | 1.38E-03 |
| 571.5 | Other chronic nonalcoholic liver disease | digestive | 217.53 | 0.00 | Random effect model | 0.79 | 0.66 - 0.93 | 0.01 |
| 571.8 | Liver abscess and sequelae of chronic liver disease | digestive | 102.91 | 0.87 | Fixed-effect model | 0.78 | 0.66 - 0.92 | 3.71E-03 |
| 571.81 | Portal hypertension | digestive | 141.96 | 0.08 | Fixed-effect model | 0.80 | 0.64 - 1.01 | 0.06 |
| 572 | Ascites (non malignant) | digestive | 142.76 | 0.08 | Fixed-effect model | 0.90 | 0.79 - 1.03 | 0.13 |
| 573 | Other disorders of liver | digestive | 169.72 | 0.00 | Random effect model | 1.01 | 0.93 - 1.10 | 0.82 |
| 573.5 | Jaundice (not of newborn) | digestive | 132.60 | 0.20 | Fixed-effect model | 0.70 | 0.59 - 0.84 | 7.35E-05 |
| 573.7 | Abnormal results of function study of liver | digestive | 142.19 | 0.08 | Fixed-effect model | 0.98 | 0.89 - 1.07 | 0.60 |
| 574 | Cholelithiasis and cholecystitis | digestive | 571.02 | 6.98E-60 | Random effect model | 0.84 | 0.77 - 0.93 | 3.40E-04 |
| 574.1 | Cholelithiasis | digestive | 550.08 | 2.74E-56 | Random effect model | 0.85 | 0.77 - 0.93 | 9.21E-04 |
| 574.11 | Cholelithiasis with acute cholecystitis | digestive | 182.34 | 0.00 | Random effect model | 0.83 | 0.70 - 0.97 | 0.02 |
| 574.12 | Cholelithiasis with other cholecystitis | digestive | 345.22 | 0.00 | Random effect model | 0.88 | 0.78 - 1.00 | 0.04 |
| 574.2 | Calculus of bile duct | digestive | 181.22 | 2.61E-04 | Random effect model | 0.80 | 0.70 - 0.90 | 3.18E-04 |
| 574.3 | Cholecystitis without cholelithiasis | digestive | 160.37 | 0.01 | Random effect model | 0.76 | 0.68 - 0.86 | 3.85E-06 |
| 575 | Other biliary tract disease | digestive | 155.41 | 0.02 | Random effect model | 0.86 | 0.78 - 0.95 | 2.24E-03 |
| 575.2 | Obstruction of bile duct | digestive | 151.24 | 0.03 | Random effect model | 0.92 | 0.75 - 1.14 | 0.44 |
| 575.7 | Other disorders of gallbladder | digestive | 160.32 | 0.01 | Random effect model | 1.00 | 0.85 - 1.17 | 0.98 |
| 575.8 | Other disorders of biliary tract | digestive | 122.39 | 0.42 | Fixed-effect model | 0.75 | 0.64 - 0.88 | 4.52E-04 |
| 577 | Diseases of pancreas | digestive | 182.13 | 0.00 | Random effect model | 0.90 | 0.80 - 1.02 | 0.11 |
| 577.2 | Chronic pancreatitis | digestive | 115.30 | 0.60 | Fixed-effect model | 1.00 | 0.80 - 1.26 | 0.97 |
| 578 | Gastrointestinal hemorrhage | digestive | 142.86 | 0.08 | Fixed-effect model | 1.04 | 1.00 - 1.08 | 0.04 |
| 578.2 | Blood in stool | digestive | 134.42 | 0.17 | Fixed-effect model | 1.07 | 0.96 - 1.18 | 0.22 |
| 578.8 | Hemorrhage of rectum and anus | digestive | 133.42 | 0.19 | Fixed-effect model | 1.03 | 0.99 - 1.08 | 0.17 |
| 578.9 | Hemorrhage of gastrointestinal tract | digestive | 107.70 | 0.78 | Fixed-effect model | 1.05 | 0.98 - 1.13 | 0.15 |
| 579 | Other symptoms involving abdomen and pelvis | digestive | 106.58 | 0.80 | Fixed-effect model | 1.00 | 0.92 - 1.10 | 0.95 |
| 579.8 | Nonspecific abnormal findings in stool contents | digestive | 119.96 | 0.48 | Fixed-effect model | 1.02 | 0.90 - 1.15 | 0.80 |
| 580 | Nephritis; nephrosis; renal sclerosis | genitourinary | 124.68 | 0.37 | Fixed-effect model | 1.08 | 0.95 - 1.24 | 0.23 |
| 580.1 | Glomerulonephritis | genitourinary | 111.58 | 0.70 | Fixed-effect model | 1.03 | 0.88 - 1.21 | 0.72 |
| 580.14 | Chronic glomerulonephritis, NOS | genitourinary | 128.87 | 0.27 | Fixed-effect model | 1.06 | 0.89 - 1.27 | 0.52 |
| 585 | Renal failure | genitourinary | 121.11 | 0.45 | Fixed-effect model | 1.03 | 0.96 - 1.09 | 0.42 |
| 585.1 | Acute renal failure | genitourinary | 138.63 | 0.12 | Fixed-effect model | 1.05 | 0.98 - 1.14 | 0.19 |
| 585.2 | Renal failure NOS | genitourinary | 82.46 | 1.00 | Fixed-effect model | 1.12 | 0.97 - 1.28 | 0.11 |
| 585.3 | Chronic renal failure [CKD] | genitourinary | 140.71 | 0.10 | Fixed-effect model | 0.98 | 0.88 - 1.08 | 0.63 |
| 586 | Other disorders of the kidney and ureters | genitourinary | 109.13 | 0.75 | Fixed-effect model | 1.01 | 0.92 - 1.11 | 0.82 |
| 586.2 | Cyst of kidney, acquired | genitourinary | 115.18 | 0.61 | Fixed-effect model | 0.99 | 0.85 - 1.14 | 0.87 |
| 586.4 | Stricture/obstruction of ureter | genitourinary | 112.10 | 0.68 | Fixed-effect model | 1.05 | 0.88 - 1.25 | 0.59 |
| 590 | Pyelonephritis | genitourinary | 123.19 | 0.40 | Fixed-effect model | 0.98 | 0.85 - 1.13 | 0.82 |
| 591 | Urinary tract infection | genitourinary | 115.80 | 0.59 | Fixed-effect model | 0.99 | 0.94 - 1.04 | 0.66 |
| 592 | Cystitis and urethritis | genitourinary | 90.33 | 0.98 | Fixed-effect model | 0.99 | 0.90 - 1.09 | 0.83 |
| 592.1 | Cystitis | genitourinary | 87.80 | 0.99 | Fixed-effect model | 0.98 | 0.89 - 1.08 | 0.71 |
| 592.12 | Chronic cystitis | genitourinary | 93.29 | 0.97 | Fixed-effect model | 1.01 | 0.85 - 1.20 | 0.90 |
| 593 | Hematuria | genitourinary | 189.78 | 0.00 | Random effect model | 1.00 | 0.95 - 1.05 | 0.93 |
| 594 | Urinary calculus | genitourinary | 156.83 | 0.01 | Random effect model | 0.93 | 0.86 - 1.00 | 0.04 |
| 594.1 | Calculus of kidney | genitourinary | 127.21 | 0.31 | Fixed-effect model | 0.92 | 0.84 - 1.01 | 0.08 |
| 594.2 | Calculus of lower urinary tract | genitourinary | 120.42 | 0.47 | Fixed-effect model | 0.88 | 0.73 - 1.06 | 0.17 |
| 594.3 | Calculus of ureter | genitourinary | 104.61 | 0.84 | Fixed-effect model | 1.01 | 0.91 - 1.12 | 0.84 |
| 596 | Other disorders of bladder | genitourinary | 103.90 | 0.85 | Fixed-effect model | 0.98 | 0.93 - 1.03 | 0.45 |
| 596.5 | Functional disorders of bladder | genitourinary | 112.57 | 0.67 | Fixed-effect model | 0.79 | 0.69 - 0.89 | 1.96E-04 |
| 597 | Other disorders of urethra and urinary tract | genitourinary | 123.79 | 0.39 | Fixed-effect model | 1.08 | 0.99 - 1.17 | 0.08 |
| 597.1 | Urethral stricture (not specified as infectious) | genitourinary | 106.50 | 0.81 | Fixed-effect model | 1.05 | 0.96 - 1.15 | 0.31 |
| 598 | Abnormal findings on examination of urine | genitourinary | 129.08 | 0.27 | Fixed-effect model | 0.88 | 0.80 - 0.96 | 3.01E-03 |
| 599 | Other symptoms/disorders or the urinary system | genitourinary | 119.60 | 0.49 | Fixed-effect model | 0.98 | 0.94 - 1.01 | 0.15 |
| 599.2 | Retention of urine | genitourinary | 108.39 | 0.77 | Fixed-effect model | 1.09 | 1.02 - 1.16 | 0.01 |
| 599.3 | Dysuria | genitourinary | 128.09 | 0.29 | Fixed-effect model | 0.92 | 0.79 - 1.07 | 0.26 |
| 599.4 | Urinary incontinence | genitourinary | 118.30 | 0.53 | Fixed-effect model | 0.86 | 0.82 - 0.91 | 2.94E-07 |
| 599.5 | Frequency of urination and polyuria | genitourinary | 117.64 | 0.54 | Fixed-effect model | 1.03 | 0.95 - 1.12 | 0.48 |
| 611 | Abnormal findings on mammogram or breast exam | genitourinary | 121.56 | 0.44 | Fixed-effect model | 1.26 | 1.10 - 1.43 | 5.82E-04 |
| 611.3 | Lump or mass in breast | genitourinary | 115.83 | 0.59 | Fixed-effect model | 1.27 | 1.11 - 1.45 | 4.65E-04 |
| 612 | Breast conditions, congenital or relating to hormones | genitourinary | 104.96 | 0.83 | Fixed-effect model | 0.90 | 0.76 - 1.07 | 0.23 |
| 612.2 | Hypertrophy of breast (Gynecomastia) | genitourinary | 109.82 | 0.74 | Fixed-effect model | 0.88 | 0.74 - 1.06 | 0.17 |
| 613.1 | Inflammatory disease of breast | genitourinary | 110.92 | 0.71 | Fixed-effect model | 1.00 | 0.83 - 1.21 | 0.99 |
| 613.7 | Other signs and symptoms in breast | genitourinary | 116.88 | 0.56 | Fixed-effect model | 1.03 | 0.85 - 1.25 | 0.77 |
| 619.1 | Noninflammatory disorders of ovary, fallopian tube, and broad ligament | genitourinary | 95.13 | 0.95 | Fixed-effect model | 1.17 | 0.97 - 1.42 | 0.11 |
| 681 | Superficial cellulitis and abscess | dermatologic | 143.56 | 0.07 | Fixed-effect model | 0.98 | 0.92 - 1.04 | 0.43 |
| 681.1 | Cellulitis and abscess of fingers/toes | dermatologic | 92.92 | 0.97 | Fixed-effect model | 1.10 | 0.89 - 1.37 | 0.37 |
| 681.2 | Cellulitis and abscess of face/neck | dermatologic | 153.13 | 0.02 | Random effect model | 1.00 | 0.77 - 1.29 | 0.99 |
| 681.3 | Cellulitis and abscess of arm/hand | dermatologic | 144.80 | 0.06 | Fixed-effect model | 0.96 | 0.90 - 1.03 | 0.29 |
| 681.5 | Cellulitis and abscess of leg, except foot | dermatologic | 140.87 | 0.09 | Fixed-effect model | 0.96 | 0.90 - 1.03 | 0.30 |
| 681.6 | Cellulitis and abscess of foot, toe | dermatologic | 142.91 | 0.08 | Fixed-effect model | 0.97 | 0.90 - 1.04 | 0.34 |
| 681.7 | Cellulitis and abscess of trunk | dermatologic | 127.82 | 0.30 | Fixed-effect model | 1.11 | 0.90 - 1.37 | 0.34 |
| 686 | Other local infections of skin and subcutaneous tissue | dermatologic | 127.19 | 0.31 | Fixed-effect model | 0.92 | 0.85 - 0.99 | 0.04 |
| 686.1 | Carbuncle and furuncle | dermatologic | 111.20 | 0.71 | Fixed-effect model | 0.88 | 0.79 - 0.98 | 0.02 |
| 686.3 | Pilonidal cyst | dermatologic | 116.20 | 0.58 | Fixed-effect model | 1.08 | 0.88 - 1.34 | 0.46 |
| 687 | Symptoms affecting skin | dermatologic | 128.32 | 0.28 | Fixed-effect model | 1.07 | 1.00 - 1.15 | 0.06 |
| 687.1 | Rash and other nonspecific skin eruption | dermatologic | 116.09 | 0.58 | Fixed-effect model | 1.05 | 0.94 - 1.18 | 0.35 |
| 687.4 | Disturbance of skin sensation | dermatologic | 109.91 | 0.73 | Fixed-effect model | 1.08 | 0.98 - 1.19 | 0.13 |
| 689 | Disorder of skin and subcutaneous tissue NOS | dermatologic | 86.07 | 0.99 | Fixed-effect model | 0.96 | 0.89 - 1.02 | 0.19 |
| 694 | Dyschromia and Vitiligo | dermatologic | 114.62 | 0.62 | Fixed-effect model | 1.09 | 0.93 - 1.29 | 0.30 |
| 694.2 | Other dyschromia | dermatologic | 112.57 | 0.67 | Fixed-effect model | 1.09 | 0.91 - 1.31 | 0.37 |
| 695 | Erythematous conditions | dermatologic | 111.33 | 0.70 | Fixed-effect model | 1.02 | 0.92 - 1.14 | 0.69 |
| 695.7 | Prurigo and Lichen | dermatologic | 110.87 | 0.71 | Fixed-effect model | 1.11 | 0.92 - 1.33 | 0.27 |
| 696 | Psoriasis and related disorders | dermatologic | 236.37 | 0.00 | Random effect model | 1.03 | 0.88 - 1.20 | 0.72 |
| 696.4 | Psoriasis | dermatologic | 234.36 | 0.00 | Random effect model | 1.05 | 0.90 - 1.22 | 0.52 |
| 696.41 | Psoriasis vulgaris | dermatologic | 208.86 | 0.00 | Random effect model | 1.02 | 0.87 - 1.21 | 0.80 |
| 696.42 | Psoriatic arthropathy | dermatologic | 192.71 | 0.00 | Random effect model | 1.21 | 0.95 - 1.55 | 0.12 |
| 697 | Sarcoidosis | dermatologic | 128.07 | 0.29 | Fixed-effect model | 0.78 | 0.62 - 0.97 | 0.03 |
| 698 | Pruritus and related conditions | dermatologic | 112.46 | 0.67 | Fixed-effect model | 1.14 | 0.95 - 1.37 | 0.17 |
| 701 | Other hypertrophic and atrophic conditions of skin | dermatologic | 136.33 | 0.15 | Fixed-effect model | 1.06 | 0.98 - 1.14 | 0.15 |
| 701.2 | Scar conditions and fibrosis of skin | dermatologic | 118.18 | 0.53 | Fixed-effect model | 1.23 | 1.11 - 1.37 | 1.34E-04 |
| 702 | Degenerative skin conditions and other dermatoses | dermatologic | 147.57 | 0.04 | Random effect model | 1.04 | 0.97 - 1.13 | 0.28 |
| 702.1 | Actinic keratosis | dermatologic | 155.01 | 0.02 | Random effect model | 0.99 | 0.88 - 1.11 | 0.80 |
| 702.2 | Seborrheic keratosis | dermatologic | 132.55 | 0.20 | Fixed-effect model | 1.11 | 1.01 - 1.22 | 0.03 |
| 703 | Diseases of nail, NOS | dermatologic | 126.69 | 0.32 | Fixed-effect model | 1.05 | 0.91 - 1.21 | 0.51 |
| 703.1 | Ingrowing nail | dermatologic | 119.67 | 0.49 | Fixed-effect model | 1.05 | 0.89 - 1.24 | 0.57 |
| 704 | Diseases of hair and hair follicles | dermatologic | 109.42 | 0.75 | Fixed-effect model | 1.03 | 0.96 - 1.11 | 0.40 |
| 705 | Disorders of sweat glands | dermatologic | 145.37 | 0.06 | Fixed-effect model | 0.91 | 0.76 - 1.09 | 0.29 |
| 705.8 | Hyperhidrosis | dermatologic | 138.97 | 0.11 | Fixed-effect model | 0.90 | 0.73 - 1.11 | 0.34 |
| 706 | Diseases of sebaceous glands | dermatologic | 164.46 | 0.00 | Random effect model | 1.03 | 0.96 - 1.09 | 0.45 |
| 706.2 | Sebaceous cyst | dermatologic | 159.41 | 0.01 | Random effect model | 1.03 | 0.97 - 1.10 | 0.38 |
| 707.1 | Decubitus ulcer | dermatologic | 112.56 | 0.67 | Fixed-effect model | 1.16 | 0.97 - 1.37 | 0.10 |
| 709 | Diffuse diseases of connective tissue | dermatologic | 126.09 | 0.33 | Fixed-effect model | 0.98 | 0.90 - 1.07 | 0.66 |
| 709.2 | Sicca syndrome | dermatologic | 156.99 | 0.01 | Random effect model | 1.23 | 0.95 - 1.59 | 0.12 |
| 709.7 | Unspecified diffuse connective tissue disease | dermatologic | 112.26 | 0.68 | Fixed-effect model | 0.96 | 0.87 - 1.06 | 0.38 |
| 710 | Osteomyelitis, periostitis, and other infections involving bone | musculoskeletal | 121.30 | 0.45 | Fixed-effect model | 1.10 | 0.89 - 1.36 | 0.37 |
| 710.1 | Osteomyelitis | musculoskeletal | 121.97 | 0.43 | Fixed-effect model | 1.10 | 0.88 - 1.36 | 0.41 |
| 714 | Rheumatoid arthritis and other inflammatory polyarthropathies | musculoskeletal | 111.82 | 0.69 | Fixed-effect model | 0.95 | 0.88 - 1.03 | 0.20 |
| 714.1 | Rheumatoid arthritis | musculoskeletal | 122.82 | 0.41 | Fixed-effect model | 0.98 | 0.91 - 1.06 | 0.67 |
| 715 | Other inflammatory spondylopathies | musculoskeletal | 115.85 | 0.59 | Fixed-effect model | 1.05 | 0.92 - 1.19 | 0.47 |
| 715.2 | Ankylosing spondylitis | musculoskeletal | 143.03 | 0.07 | Fixed-effect model | 1.02 | 0.83 - 1.25 | 0.87 |
| 716 | Other arthropathies | musculoskeletal | 178.82 | 4.02E-04 | Random effect model | 0.92 | 0.89 - 0.95 | 6.06E-06 |
| 716.1 | Unspecified polyarthropathy or polyarthritis | musculoskeletal | 150.66 | 0.03 | Random effect model | 1.03 | 0.93 - 1.13 | 0.59 |
| 716.2 | Unspecified monoarthritis | musculoskeletal | 125.29 | 0.35 | Fixed-effect model | 0.89 | 0.85 - 0.93 | 4.68E-08 |
| 716.9 | Arthropathy NOS | musculoskeletal | 178.72 | 4.10E-04 | Random effect model | 0.91 | 0.88 - 0.95 | 9.03E-07 |
| 717 | Polymyalgia Rheumatica | musculoskeletal | 97.22 | 0.94 | Fixed-effect model | 0.94 | 0.81 - 1.10 | 0.44 |
| 720 | Spinal stenosis | musculoskeletal | 146.12 | 0.05 | Fixed-effect model | 0.92 | 0.84 - 1.00 | 0.04 |
| 721 | Spondylosis and allied disorders | musculoskeletal | 127.42 | 0.30 | Fixed-effect model | 0.92 | 0.87 - 0.98 | 0.01 |
| 721.1 | Spondylosis without myelopathy | musculoskeletal | 118.31 | 0.53 | Fixed-effect model | 0.91 | 0.84 - 0.97 | 0.01 |
| 722 | Intervertebral disc disorders | musculoskeletal | 139.47 | 0.11 | Fixed-effect model | 0.90 | 0.85 - 0.95 | 1.36E-04 |
| 722.1 | Displacement of intervertebral disc | musculoskeletal | 135.82 | 0.15 | Fixed-effect model | 1.08 | 0.86 - 1.36 | 0.49 |
| 722.6 | Degeneration of intervertebral disc | musculoskeletal | 127.50 | 0.30 | Fixed-effect model | 0.85 | 0.77 - 0.94 | 9.94E-04 |
| 722.9 | Other and unspecified disc disorder | musculoskeletal | 139.70 | 0.11 | Fixed-effect model | 0.85 | 0.78 - 0.92 | 3.42E-05 |
| 724.9 | Other unspecified back disorders | musculoskeletal | 113.60 | 0.65 | Fixed-effect model | 0.95 | 0.84 - 1.09 | 0.48 |
| 726 | Peripheral enthesopathies and allied syndromes | musculoskeletal | 124.24 | 0.38 | Fixed-effect model | 0.89 | 0.85 - 0.93 | 1.56E-07 |
| 726.1 | Enthesopathy | musculoskeletal | 108.20 | 0.77 | Fixed-effect model | 0.92 | 0.87 - 0.97 | 2.19E-03 |
| 726.3 | Bursitis | musculoskeletal | 95.53 | 0.95 | Fixed-effect model | 0.74 | 0.62 - 0.88 | 7.66E-04 |
| 727 | Other disorders of synovium, tendon, and bursa | musculoskeletal | 132.60 | 0.20 | Fixed-effect model | 0.92 | 0.86 - 0.97 | 4.68E-03 |
| 727.1 | Synovitis and tenosynovitis | musculoskeletal | 104.98 | 0.83 | Fixed-effect model | 0.88 | 0.80 - 0.97 | 0.01 |
| 727.4 | Ganglion and cyst of synovium, tendon, and bursa | musculoskeletal | 122.54 | 0.42 | Fixed-effect model | 0.97 | 0.88 - 1.06 | 0.50 |
| 727.5 | Rupture of synovium | musculoskeletal | 98.94 | 0.92 | Fixed-effect model | 0.91 | 0.73 - 1.12 | 0.37 |
| 728 | Disorders of muscle, ligament, and fascia | musculoskeletal | 127.29 | 0.31 | Fixed-effect model | 0.94 | 0.87 - 1.02 | 0.11 |
| 728.7 | Fasciitis | musculoskeletal | 126.51 | 0.32 | Fixed-effect model | 0.98 | 0.90 - 1.06 | 0.57 |
| 728.71 | Contracture of palmar fascia [Dupuytren's disease] | musculoskeletal | 125.44 | 0.35 | Fixed-effect model | 1.04 | 0.95 - 1.14 | 0.38 |
| 729 | Other disorders of soft tissues | musculoskeletal | 130.39 | 0.24 | Fixed-effect model | 0.89 | 0.83 - 0.95 | 3.14E-04 |
| 729.1 | Rheumatism, unspecified and fibrositis | musculoskeletal | 92.62 | 0.97 | Fixed-effect model | 0.83 | 0.71 - 0.98 | 0.03 |
| 733 | Other disorders of bone and cartilage | musculoskeletal | 89.66 | 0.98 | Fixed-effect model | 0.90 | 0.83 - 0.98 | 0.01 |
| 733.4 | Aseptic necrosis of bone | musculoskeletal | 108.26 | 0.77 | Fixed-effect model | 0.87 | 0.70 - 1.09 | 0.23 |
| 733.8 | Malunion and nonunion of fracture | musculoskeletal | 108.60 | 0.76 | Fixed-effect model | 0.97 | 0.84 - 1.12 | 0.66 |
| 735 | Acquired foot deformities | musculoskeletal | 145.32 | 0.06 | Fixed-effect model | 0.92 | 0.87 - 0.97 | 1.39E-03 |
| 735.2 | Acquired toe deformities | musculoskeletal | 162.83 | 0.01 | Random effect model | 0.91 | 0.84 - 0.99 | 0.03 |
| 735.23 | Hallux rigidus | musculoskeletal | 98.91 | 0.92 | Fixed-effect model | 0.97 | 0.85 - 1.10 | 0.63 |
| 735.3 | Hallux valgus (Bunion) | musculoskeletal | 122.15 | 0.43 | Fixed-effect model | 0.89 | 0.83 - 0.95 | 4.69E-04 |
| 736 | Other acquired deformities of limbs | musculoskeletal | 160.26 | 0.01 | Random effect model | 0.85 | 0.73 - 0.99 | 0.03 |
| 737 | Curvature of spine | musculoskeletal | 123.21 | 0.40 | Fixed-effect model | 0.83 | 0.72 - 0.97 | 0.02 |
| 737.3 | Kyphoscoliosis and scoliosis | musculoskeletal | 115.44 | 0.60 | Fixed-effect model | 0.89 | 0.76 - 1.04 | 0.16 |
| 738.4 | Acquired spondylolisthesis | musculoskeletal | 104.05 | 0.85 | Fixed-effect model | 0.96 | 0.84 - 1.10 | 0.57 |
| 740 | Osteoarthrosis | musculoskeletal | 199.95 | 6.40E-06 | Random effect model | 0.89 | 0.85 - 0.93 | 1.30E-07 |
| 740.1 | Osteoarthritis; localized | musculoskeletal | 198.31 | 9.04E-06 | Random effect model | 0.86 | 0.81 - 0.90 | 5.28E-09 |
| 740.11 | Osteoarthrosis, localized, primary | musculoskeletal | 135.19 | 0.16 | Fixed-effect model | 0.85 | 0.81 - 0.90 | 2.66E-08 |
| 740.2 | Osteoarthrosis, generalized | musculoskeletal | 118.53 | 0.52 | Fixed-effect model | 0.97 | 0.79 - 1.20 | 0.79 |
| 740.9 | Osteoarthrosis NOS | musculoskeletal | 158.32 | 0.01 | Random effect model | 0.95 | 0.90 - 1.01 | 0.09 |
| 741 | Symptoms and disorders of the joints | musculoskeletal | 94.88 | 0.96 | Fixed-effect model | 0.95 | 0.87 - 1.03 | 0.22 |
| 741.2 | Stiffness of joint | musculoskeletal | 94.40 | 0.96 | Fixed-effect model | 0.86 | 0.70 - 1.06 | 0.15 |
| 741.4 | Joint effusions | musculoskeletal | 102.09 | 0.88 | Fixed-effect model | 1.04 | 0.91 - 1.19 | 0.59 |
| 742 | Derangement of joint, nontraumatic | musculoskeletal | 92.88 | 0.97 | Fixed-effect model | 0.80 | 0.73 - 0.88 | 6.57E-06 |
| 742.8 | Articular cartilage disorder | musculoskeletal | 102.86 | 0.87 | Fixed-effect model | 0.79 | 0.63 - 0.99 | 0.04 |
| 743 | Osteoporosis, osteopenia and pathological fracture | musculoskeletal | 132.82 | 0.20 | Fixed-effect model | 1.00 | 0.95 - 1.07 | 0.89 |
| 743.1 | Osteoporosis | musculoskeletal | 143.07 | 0.07 | Fixed-effect model | 0.99 | 0.93 - 1.06 | 0.86 |
| 743.11 | Osteoporosis NOS | musculoskeletal | 142.28 | 0.08 | Fixed-effect model | 0.97 | 0.90 - 1.04 | 0.36 |
| 743.13 | Other specified osteoporosis | musculoskeletal | 104.42 | 0.84 | Fixed-effect model | 0.97 | 0.78 - 1.22 | 0.82 |
| 743.2 | Pathologic fracture | musculoskeletal | 166.18 | 0.00 | Random effect model | 0.97 | 0.74 - 1.27 | 0.83 |
| 743.9 | Osteopenia or other disorder of bone and cartilage | musculoskeletal | 111.37 | 0.70 | Fixed-effect model | 1.06 | 0.88 - 1.27 | 0.55 |
| 745 | Pain in joint | musculoskeletal | 146.51 | 0.05 | Fixed-effect model | 0.97 | 0.92 - 1.03 | 0.33 |
| 747 | Cardiac and circulatory congenital anomalies | congenital anomalies | 154.77 | 0.02 | Random effect model | 1.20 | 1.08 - 1.35 | 1.10E-03 |
| 747.1 | Cardiac congenital anomalies | congenital anomalies | 171.30 | 1.48E-03 | Random effect model | 1.22 | 1.08 - 1.38 | 1.08E-03 |
| 747.11 | Cardiac shunt/ heart septal defect | congenital anomalies | 116.93 | 0.56 | Fixed-effect model | 0.93 | 0.75 - 1.15 | 0.48 |
| 747.13 | Congenital anomalies of great vessels | congenital anomalies | 162.80 | 0.01 | Random effect model | 1.38 | 1.20 - 1.59 | 8.23E-06 |
| 750 | Digestive congenital anomalies | congenital anomalies | 120.86 | 0.46 | Fixed-effect model | 0.80 | 0.66 - 0.97 | 0.02 |
| 751 | Genitourinary congenital anomalies | congenital anomalies | 120.43 | 0.47 | Fixed-effect model | 0.75 | 0.66 - 0.86 | 1.42E-05 |
| 751.1 | Congenital anomalies of genital organs | congenital anomalies | 114.71 | 0.62 | Fixed-effect model | 0.82 | 0.68 - 1.00 | 0.05 |
| 751.2 | Congenital anomalies of urinary system | congenital anomalies | 107.79 | 0.78 | Fixed-effect model | 0.70 | 0.59 - 0.84 | 7.92E-05 |
| 756 | Other congenital musculoskeletal anomalies | congenital anomalies | 123.06 | 0.41 | Fixed-effect model | 0.67 | 0.54 - 0.83 | 2.40E-04 |
| 760 | Back pain | symptoms | 123.46 | 0.40 | Fixed-effect model | 0.94 | 0.90 - 0.99 | 0.02 |
| 761 | Cervicalgia | symptoms | 116.76 | 0.57 | Fixed-effect model | 1.06 | 0.94 - 1.20 | 0.33 |
| 764 | Sciatica | symptoms | 137.58 | 0.13 | Fixed-effect model | 0.93 | 0.84 - 1.04 | 0.20 |
| 766 | Neuralgia, neuritis, and radiculitis NOS | symptoms | 118.88 | 0.51 | Fixed-effect model | 0.84 | 0.72 - 0.98 | 0.02 |
| 770 | Myalgia and myositis unspecified | symptoms | 119.17 | 0.50 | Fixed-effect model | 1.27 | 1.04 - 1.56 | 0.02 |
| 771 | Musculoskeletal symptoms referable to limbs | symptoms | 124.96 | 0.36 | Fixed-effect model | 0.87 | 0.81 - 0.93 | 6.81E-05 |
| 771.1 | Swelling of limb | symptoms | 125.08 | 0.36 | Fixed-effect model | 0.87 | 0.81 - 0.94 | 4.56E-04 |
| 772 | Symptoms of the muscles | symptoms | 107.80 | 0.78 | Fixed-effect model | 0.94 | 0.77 - 1.14 | 0.54 |
| 773 | Pain in limb | symptoms | 129.47 | 0.26 | Fixed-effect model | 0.98 | 0.92 - 1.05 | 0.53 |
| 782 | Symptoms involving skin and other integumentary tissue | symptoms | 129.21 | 0.27 | Fixed-effect model | 0.99 | 0.87 - 1.11 | 0.81 |
| 782.3 | Edema | symptoms | 126.63 | 0.32 | Fixed-effect model | 0.96 | 0.84 - 1.09 | 0.51 |
| 783 | Fever of unknown origin | symptoms | 106.13 | 0.81 | Fixed-effect model | 0.99 | 0.91 - 1.08 | 0.86 |
| 785 | Abdominal pain | symptoms | 106.71 | 0.80 | Fixed-effect model | 0.96 | 0.94 - 0.99 | 0.01 |
| 788 | Syncope and collapse | symptoms | 112.28 | 0.68 | Fixed-effect model | 1.15 | 1.08 - 1.21 | 1.13E-06 |
| 789 | Nausea and vomiting | symptoms | 137.37 | 0.13 | Fixed-effect model | 0.95 | 0.90 - 0.99 | 0.02 |
| 790 | Nonspecific findings on examination of blood | symptoms | 154.06 | 0.02 | Random effect model | 0.99 | 0.92 - 1.07 | 0.82 |
| 790.6 | Other abnormal blood chemistry | symptoms | 166.78 | 0.00 | Random effect model | 0.99 | 0.92 - 1.07 | 0.87 |
| 791 | Gangrene | symptoms | 133.62 | 0.19 | Fixed-effect model | 1.00 | 0.80 - 1.24 | 0.97 |
| 793 | Nonspecific abnormal findings on radiological and other examination of musculoskeletal system | injuries & poisonings | 101.84 | 0.88 | Fixed-effect model | 1.18 | 0.97 - 1.44 | 0.09 |
| 793.2 | Nonspecific abnormal findings on radiological and other examination of other intrathoracic organs (echocardiogram, etc) | circulatory system | 101.33 | 0.89 | Fixed-effect model | 1.21 | 0.98 - 1.51 | 0.08 |
| 798 | Malaise and fatigue | symptoms | 112.26 | 0.68 | Fixed-effect model | 1.01 | 0.92 - 1.10 | 0.85 |
| 798.1 | Chronic fatigue syndrome | symptoms | 122.28 | 0.42 | Fixed-effect model | 0.79 | 0.64 - 0.98 | 0.03 |
| 800 | Fracture of lower limb | injuries & poisonings | 123.65 | 0.39 | Fixed-effect model | 0.93 | 0.87 - 0.99 | 0.02 |
| 800.1 | Fracture of neck of femur | injuries & poisonings | 142.64 | 0.08 | Fixed-effect model | 0.78 | 0.69 - 0.88 | 6.27E-05 |
| 800.3 | Fracture of tibia and fibula | injuries & poisonings | 93.06 | 0.97 | Fixed-effect model | 0.99 | 0.88 - 1.10 | 0.82 |
| 800.4 | Fracture of patella | injuries & poisonings | 119.99 | 0.48 | Fixed-effect model | 1.10 | 0.91 - 1.32 | 0.31 |
| 801 | Fracture of ankle and foot | injuries & poisonings | 119.88 | 0.49 | Fixed-effect model | 0.99 | 0.89 - 1.10 | 0.86 |
| 802 | Fracture of pelvis | injuries & poisonings | 152.64 | 0.02 | Random effect model | 0.92 | 0.74 - 1.15 | 0.46 |
| 803 | Fracture of upper limb | injuries & poisonings | 135.86 | 0.15 | Fixed-effect model | 1.01 | 0.95 - 1.07 | 0.72 |
| 803.1 | Fracture of humerus | injuries & poisonings | 136.86 | 0.14 | Fixed-effect model | 1.02 | 0.90 - 1.16 | 0.76 |
| 803.2 | Fracture of radius and ulna | injuries & poisonings | 132.17 | 0.21 | Fixed-effect model | 1.01 | 0.94 - 1.08 | 0.86 |
| 803.3 | Fracture of clavicle or scapula | injuries & poisonings | 120.34 | 0.47 | Fixed-effect model | 0.97 | 0.85 - 1.11 | 0.70 |
| 804 | Fracture of hand or wrist | injuries & poisonings | 118.40 | 0.52 | Fixed-effect model | 1.11 | 1.02 - 1.21 | 0.02 |
| 805 | Fracture of vertebral column without mention of spinal cord injury | injuries & poisonings | 131.27 | 0.23 | Fixed-effect model | 0.97 | 0.84 - 1.11 | 0.64 |
| 807 | Fracture of ribs | injuries & poisonings | 111.29 | 0.70 | Fixed-effect model | 0.86 | 0.72 - 1.04 | 0.11 |
| 809 | Fracture of unspecified bones | injuries & poisonings | 115.16 | 0.61 | Fixed-effect model | 0.98 | 0.85 - 1.13 | 0.80 |
| 818 | Intracranial hemorrhage (injury) | injuries & poisonings | 131.85 | 0.22 | Fixed-effect model | 1.08 | 0.86 - 1.36 | 0.49 |
| 819 | Skull and face fracture and other intercranial injury | injuries & poisonings | 135.16 | 0.16 | Fixed-effect model | 0.99 | 0.90 - 1.09 | 0.88 |
| 823 | Torus fracture | injuries & poisonings | 98.32 | 0.93 | Fixed-effect model | 0.95 | 0.84 - 1.08 | 0.47 |
| 835 | Internal derangement of knee | injuries & poisonings | 113.43 | 0.65 | Fixed-effect model | 0.96 | 0.92 - 1.00 | 0.04 |
| 836 | Traumatic arthropathy | injuries & poisonings | 110.03 | 0.73 | Fixed-effect model | 1.18 | 0.95 - 1.48 | 0.14 |
| 850 | Hemorrhage or hematoma complicating a procedure | injuries & poisonings | 106.44 | 0.81 | Fixed-effect model | 1.15 | 1.07 - 1.23 | 1.60E-04 |
| 853 | Complication of colostomy or enterostomy | injuries & poisonings | 110.90 | 0.71 | Fixed-effect model | 0.91 | 0.72 - 1.14 | 0.40 |
| 854 | Complications of cardiac/vascular device, implant, and graft | injuries & poisonings | 141.76 | 0.09 | Fixed-effect model | 1.39 | 1.23 - 1.57 | 1.71E-07 |
| 857 | Mechanical complication of unspecified genitourinary device, implant, and graft | injuries & poisonings | 132.23 | 0.21 | Fixed-effect model | 1.35 | 1.16 - 1.56 | 7.57E-05 |
| 858 | Complication of internal orthopedic device | injuries & poisonings | 120.53 | 0.47 | Fixed-effect model | 0.76 | 0.70 - 0.84 | 8.90E-09 |
| 859 | Complication due to other implant and internal device | injuries & poisonings | 98.97 | 0.92 | Fixed-effect model | 0.86 | 0.78 - 0.94 | 1.18E-03 |
| 870 | Open wounds of head; neck; and trunk | injuries & poisonings | 109.74 | 0.74 | Fixed-effect model | 1.17 | 1.08 - 1.27 | 2.39E-04 |
| 870.3 | Other open wound of head and face | injuries & poisonings | 101.31 | 0.89 | Fixed-effect model | 1.13 | 1.03 - 1.25 | 0.01 |
| 871 | Open wounds of extremities | injuries & poisonings | 100.37 | 0.90 | Fixed-effect model | 1.06 | 0.98 - 1.15 | 0.13 |
| 907 | Injuries to the nervous system | injuries & poisonings | 124.56 | 0.37 | Fixed-effect model | 1.03 | 0.90 - 1.19 | 0.65 |
| 915 | Superficial injury without mention of infection | injuries & poisonings | 108.51 | 0.77 | Fixed-effect model | 1.11 | 1.02 - 1.20 | 0.01 |
| 916 | Contusion | injuries & poisonings | 102.56 | 0.87 | Fixed-effect model | 1.25 | 1.09 - 1.43 | 1.09E-03 |
| 939 | Atopic/contact dermatitis due to other or unspecified | dermatologic | 113.18 | 0.66 | Fixed-effect model | 1.09 | 0.97 - 1.22 | 0.15 |
| 946 | Anaphylactic shock NOS | injuries & poisonings | 69.97 | 1.00 | Fixed-effect model | 1.15 | 0.92 - 1.43 | 0.22 |
| 960 | Poisoning by antibiotics | injuries & poisonings | 129.70 | 0.26 | Fixed-effect model | 0.93 | 0.90 - 0.97 | 8.27E-04 |
| 960.2 | Allergy/adverse effect of penicillin | injuries & poisonings | 132.03 | 0.21 | Fixed-effect model | 0.93 | 0.89 - 0.97 | 9.45E-04 |
| 961 | Poisoning by other antiinfectives | injuries & poisonings | 155.55 | 0.02 | Random effect model | 1.12 | 0.94 - 1.32 | 0.20 |
| 961.1 | Poisoning/allergy of sulfonamides | injuries & poisonings | 133.39 | 0.19 | Fixed-effect model | 1.17 | 0.98 - 1.40 | 0.07 |
| 965 | Poisoning by analgesics, antipyretics, and antirheumatics | injuries & poisonings | 128.51 | 0.28 | Fixed-effect model | 1.07 | 1.01 - 1.14 | 0.03 |
| 965.1 | Opiates and related narcotics causing adverse effects in therapeutic use | injuries & poisonings | 87.37 | 0.99 | Fixed-effect model | 1.10 | 0.95 - 1.28 | 0.22 |
| 966 | Poisoning by anticonvulsants and anti-Parkinsonism drugs | injuries & poisonings | 136.50 | 0.14 | Fixed-effect model | 0.83 | 0.66 - 1.04 | 0.11 |
| 967 | Adverse effects of sedatives or other central nervous system depressants and anesthetics | injuries & poisonings | 92.24 | 0.97 | Fixed-effect model | 0.87 | 0.71 - 1.08 | 0.21 |
| 979 | Adverse drug events and drug allergies | injuries & poisonings | 109.34 | 0.75 | Fixed-effect model | 1.16 | 0.96 - 1.42 | 0.13 |
| 990 | Effects radiation NOS | injuries & poisonings | 133.89 | 0.18 | Fixed-effect model | 1.24 | 1.13 - 1.36 | 5.88E-06 |
| 994 | Sepsis and SIRS | infectious diseases | 119.27 | 0.50 | Fixed-effect model | 0.85 | 0.77 - 0.94 | 1.20E-03 |
| 994.2 | Sepsis | infectious diseases | 119.27 | 0.50 | Fixed-effect model | 0.85 | 0.77 - 0.94 | 1.20E-03 |
| 1000 | Burns | injuries & poisonings | 113.18 | 0.66 | Fixed-effect model | 1.11 | 0.90 - 1.37 | 0.34 |
| 1001 | Foreign body injury | injuries & poisonings | 128.27 | 0.29 | Fixed-effect model | 0.84 | 0.72 - 0.97 | 0.02 |
| 1002 | Symptoms concerning nutrition, metabolism, and development | symptoms | 154.28 | 0.02 | Random effect model | 1.02 | 0.94 - 1.10 | 0.69 |
| 1005 | Other symptoms | symptoms | 105.43 | 0.83 | Fixed-effect model | 1.00 | 0.86 - 1.16 | 0.99 |
| 1008 | Crushing or internal injury to organs | injuries & poisonings | 116.31 | 0.58 | Fixed-effect model | 1.10 | 0.93 - 1.29 | 0.27 |
| 1009 | Injury, NOS | injuries & poisonings | 132.32 | 0.21 | Fixed-effect model | 1.05 | 0.99 - 1.11 | 0.10 |
| 1010 | Other tests | symptoms | 138.15 | 0.12 | Fixed-effect model | 1.04 | 0.97 - 1.11 | 0.26 |
| 1011 | Complications of surgical and medical procedures | injuries & poisonings | 78.60 | 1.00 | Fixed-effect model | 0.90 | 0.85 - 0.95 | 1.68E-04 |
| 1015 | Effects of other external causes | symptoms | 122.64 | 0.42 | Fixed-effect model | 1.04 | 0.96 - 1.13 | 0.33 |
| 1019 | Other ill-defined and unknown causes of morbidity and mortality | symptoms | 138.55 | 0.12 | Fixed-effect model | 1.03 | 0.99 - 1.08 | 0.12 |

Odds ratios (ORs) with their 95% confidence intervals (CIs) represent the effect estimates on the risk of multiple non-delirium of per 10% reduction in risk for delirium by targeting clinical low density lipoprotein cholesterol.

Significant threshold was set at *P*<2.46 x 10^-5^ (Bonferroni-corrected significance threshold calculated as 0.05 divided by 2034 [3 metabolites×678 diseases]). E signifies the exponent of 10 in the table.

Abbreviations: Phe-MR, phenome-wide Mendelian randomization; SNPs, single nucleotide polymorphisms.

**Table S8. Phe-MR analyses for the associations between Sphingomyelin and 678 diseases using the inverse-variance weighted method.**

| **PheCode** | **Phenotype Description** | **Disease Chapter** | **Q estimate** | ***P* value for Q estimate** | **IVW model** | **OR** | **95% CI** | ***P* value** |
| --- | --- | --- | --- | --- | --- | --- | --- | --- |
| 8 | Intestinal infection | infectious diseases | 131.07 | 0.94 | Fixed-effect model | 0.95 | 0.89 - 1.00 | 0.06 |
| 8.5 | Bacterial enteritis | infectious diseases | 158.57 | 0.45 | Fixed-effect model | 0.95 | 0.85 - 1.05 | 0.31 |
| 8.52 | Intestinal infection due to C. difficile | infectious diseases | 164.86 | 0.32 | Fixed-effect model | 1.11 | 0.90 - 1.38 | 0.33 |
| 8.6 | Viral Enteritis | infectious diseases | 145.84 | 0.73 | Fixed-effect model | 1.01 | 0.83 - 1.22 | 0.94 |
| 38 | Septicemia | infectious diseases | 136.67 | 0.88 | Fixed-effect model | 0.82 | 0.75 - 0.89 | 7.80E-06 |
| 38.1 | Gram negative septicemia | infectious diseases | 157.82 | 0.47 | Fixed-effect model | 0.96 | 0.79 - 1.16 | 0.68 |
| 41 | Bacterial infection NOS | infectious diseases | 154.82 | 0.53 | Fixed-effect model | 0.94 | 0.90 - 0.99 | 0.03 |
| 41.1 | Staphylococcus infections | infectious diseases | 162.53 | 0.36 | Fixed-effect model | 0.92 | 0.84 - 1.02 | 0.12 |
| 41.2 | Streptococcus infection | infectious diseases | 148.54 | 0.67 | Fixed-effect model | 0.91 | 0.80 - 1.04 | 0.18 |
| 41.4 | E. coli | infectious diseases | 160.27 | 0.41 | Fixed-effect model | 0.89 | 0.80 - 0.99 | 0.03 |
| 70 | Viral hepatitis | infectious diseases | 142.19 | 0.80 | Fixed-effect model | 0.98 | 0.84 - 1.15 | 0.85 |
| 78 | Viral warts & HPV | infectious diseases | 152.45 | 0.59 | Fixed-effect model | 0.93 | 0.78 - 1.10 | 0.40 |
| 79 | Viral infection | infectious diseases | 135.40 | 0.89 | Fixed-effect model | 0.96 | 0.87 - 1.07 | 0.45 |
| 80 | Postoperative infection | infectious diseases | 186.14 | 0.06 | Fixed-effect model | 0.91 | 0.84 - 0.99 | 0.03 |
| 81 | Infection/inflammation of internal prosthetic device; implant; and graft | infectious diseases | 148.01 | 0.68 | Fixed-effect model | 0.98 | 0.88 - 1.10 | 0.74 |
| 112 | Candidiasis | infectious diseases | 130.54 | 0.94 | Fixed-effect model | 0.86 | 0.76 - 0.97 | 0.01 |
| 145 | Cancer of mouth | neoplasms | 160.14 | 0.42 | Fixed-effect model | 0.99 | 0.80 - 1.23 | 0.94 |
| 149 | Cancer of larynx, pharynx, nasal cavities | neoplasms | 137.04 | 0.87 | Fixed-effect model | 1.08 | 0.86 - 1.34 | 0.52 |
| 150 | Cancer of esophagus | neoplasms | 189.92 | 0.04 | Random effect model | 0.80 | 0.64 - 1.01 | 0.06 |
| 151 | Cancer of stomach | neoplasms | 162.65 | 0.36 | Fixed-effect model | 0.82 | 0.65 - 1.04 | 0.10 |
| 153 | Colorectal cancer | neoplasms | 182.98 | 0.08 | Fixed-effect model | 1.14 | 1.05 - 1.24 | 1.99E-03 |
| 153.2 | Colon cancer | neoplasms | 175.84 | 0.14 | Fixed-effect model | 1.04 | 0.94 - 1.15 | 0.47 |
| 153.3 | Malignant neoplasm of rectum, rectosigmoid junction, and anus | neoplasms | 160.58 | 0.41 | Fixed-effect model | 1.19 | 1.06 - 1.34 | 4.67E-03 |
| 157 | Pancreatic cancer | neoplasms | 144.43 | 0.76 | Fixed-effect model | 0.63 | 0.50 - 0.79 | 5.59E-05 |
| 158 | Neoplasm of unspecified nature of digestive system | neoplasms | 162.39 | 0.37 | Fixed-effect model | 0.93 | 0.78 - 1.10 | 0.39 |
| 159 | Malignant neoplasm of other and ill-defined sites within the digestive organs and peritoneum | neoplasms | 214.33 | 1.61E-03 | Random effect model | 1.08 | 0.99 - 1.18 | 0.07 |
| 165 | Cancer within the respiratory system | neoplasms | 173.18 | 0.18 | Fixed-effect model | 1.02 | 0.92 - 1.13 | 0.72 |
| 165.1 | Cancer of bronchus; lung | neoplasms | 186.48 | 0.05 | Fixed-effect model | 1.02 | 0.90 - 1.15 | 0.76 |
| 172 | Skin cancer | neoplasms | 261.16 | 3.49E-07 | Random effect model | 1.07 | 1.01 - 1.14 | 0.03 |
| 172.1 | Melanomas of skin, dx or hx | neoplasms | 173.19 | 0.18 | Fixed-effect model | 1.05 | 0.95 - 1.17 | 0.33 |
| 172.11 | Melanomas of skin | neoplasms | 173.19 | 0.18 | Fixed-effect model | 1.05 | 0.95 - 1.17 | 0.33 |
| 172.2 | Other non-epithelial cancer of skin | neoplasms | 241.72 | 1.62E-05 | Random effect model | 1.07 | 1.00 - 1.14 | 0.06 |
| 172.3 | Carcinoma in situ of skin | neoplasms | 166.82 | 0.28 | Fixed-effect model | 1.22 | 0.99 - 1.52 | 0.06 |
| 174 | Breast cancer | neoplasms | 223.45 | 3.91E-04 | Random effect model | 1.06 | 1.00 - 1.13 | 0.06 |
| 189 | Cancer of urinary organs (incl. kidney and bladder) | neoplasms | 182.75 | 0.08 | Fixed-effect model | 1.05 | 0.96 - 1.14 | 0.27 |
| 189.1 | Cancer of kidney and renal pelvis | neoplasms | 167.25 | 0.27 | Fixed-effect model | 1.11 | 0.94 - 1.32 | 0.22 |
| 189.11 | Malignant neoplasm of kidney, except pelvis | neoplasms | 168.16 | 0.26 | Fixed-effect model | 1.14 | 0.95 - 1.35 | 0.15 |
| 189.2 | Cancer of bladder | neoplasms | 133.87 | 0.91 | Fixed-effect model | 1.09 | 0.98 - 1.22 | 0.12 |
| 189.21 | Malignant neoplasm of bladder | neoplasms | 128.84 | 0.95 | Fixed-effect model | 1.13 | 1.01 - 1.28 | 0.04 |
| 191 | Manlignant and unknown neoplasms of brain and nervous system | neoplasms | 150.39 | 0.63 | Fixed-effect model | 1.37 | 1.10 - 1.69 | 4.32E-03 |
| 191.1 | Cancer of brain and nervous system | neoplasms | 160.58 | 0.41 | Fixed-effect model | 1.28 | 1.01 - 1.63 | 0.04 |
| 195 | Cancer, suspected or other | neoplasms | 198.79 | 0.01 | Random effect model | 1.03 | 0.98 - 1.08 | 0.30 |
| 195.1 | Malignant neoplasm, other | neoplasms | 188.10 | 0.05 | Random effect model | 1.04 | 0.99 - 1.09 | 0.12 |
| 197 | Chemotherapy | neoplasms | 147.95 | 0.69 | Fixed-effect model | 0.95 | 0.91 - 0.98 | 0.01 |
| 198 | Secondary malignant neoplasm | neoplasms | 179.58 | 0.10 | Fixed-effect model | 1.00 | 0.94 - 1.05 | 0.87 |
| 198.1 | Secondary malignancy of lymph nodes | neoplasms | 214.93 | 1.48E-03 | Random effect model | 1.02 | 0.93 - 1.11 | 0.73 |
| 198.2 | Secondary malignancy of respiratory organs | neoplasms | 162.01 | 0.38 | Fixed-effect model | 1.07 | 0.95 - 1.20 | 0.29 |
| 198.3 | Secondary malignant neoplasm of digestive systems | neoplasms | 157.16 | 0.48 | Fixed-effect model | 1.05 | 0.91 - 1.21 | 0.49 |
| 198.4 | Secondary malignant neoplasm of liver | neoplasms | 142.62 | 0.79 | Fixed-effect model | 0.90 | 0.81 - 1.00 | 0.05 |
| 198.5 | Secondary malignancy of brain/spine | neoplasms | 169.21 | 0.24 | Fixed-effect model | 1.06 | 0.87 - 1.28 | 0.59 |
| 198.6 | Secondary malignancy of bone | neoplasms | 177.26 | 0.13 | Fixed-effect model | 1.04 | 0.92 - 1.17 | 0.54 |
| 199 | Neoplasm of uncertain behavior | neoplasms | 121.39 | 0.98 | Fixed-effect model | 1.26 | 1.08 - 1.45 | 2.31E-03 |
| 200 | Myeloproliferative disease | neoplasms | 238.17 | 3.11E-05 | Random effect model | 0.77 | 0.62 - 0.95 | 0.02 |
| 202 | Cancer of other lymphoid, histiocytic tissue | neoplasms | 158.93 | 0.44 | Fixed-effect model | 1.01 | 0.90 - 1.13 | 0.89 |
| 202.2 | Non-Hodgkins lymphoma | neoplasms | 181.72 | 0.09 | Fixed-effect model | 1.01 | 0.88 - 1.15 | 0.91 |
| 202.24 | Large cell lymphoma | neoplasms | 154.99 | 0.53 | Fixed-effect model | 0.81 | 0.64 - 1.01 | 0.07 |
| 204 | Leukemia | neoplasms | 173.46 | 0.17 | Fixed-effect model | 1.14 | 0.99 - 1.30 | 0.06 |
| 204.1 | Lymphoid leukemia | neoplasms | 178.30 | 0.12 | Fixed-effect model | 1.04 | 0.83 - 1.31 | 0.74 |
| 204.12 | Lymphoid leukemia, chronic | neoplasms | 175.64 | 0.15 | Fixed-effect model | 1.00 | 0.78 - 1.28 | 0.99 |
| 204.4 | Multiple myeloma | neoplasms | 146.62 | 0.71 | Fixed-effect model | 1.10 | 0.87 - 1.39 | 0.44 |
| 208 | Benign neoplasm of colon | neoplasms | 307.64 | 7.61E-12 | Random effect model | 1.04 | 0.98 - 1.10 | 0.23 |
| 210 | Benign neoplasm of lip, oral cavity, and pharynx | neoplasms | 135.48 | 0.89 | Fixed-effect model | 0.81 | 0.68 - 0.96 | 0.02 |
| 211 | Benign neoplasm of other parts of digestive system | neoplasms | 170.79 | 0.21 | Fixed-effect model | 0.97 | 0.90 - 1.04 | 0.39 |
| 214 | Lipoma | neoplasms | 178.72 | 0.11 | Fixed-effect model | 0.99 | 0.92 - 1.06 | 0.74 |
| 214.1 | Lipoma of skin and subcutaneous tissue | neoplasms | 184.07 | 0.07 | Fixed-effect model | 0.99 | 0.91 - 1.08 | 0.84 |
| 215 | Other benign neoplasm of connective and other soft tissue | neoplasms | 164.68 | 0.32 | Fixed-effect model | 0.89 | 0.76 - 1.05 | 0.17 |
| 216 | Benign neoplasm of skin | neoplasms | 136.43 | 0.88 | Fixed-effect model | 1.10 | 1.03 - 1.17 | 3.23E-03 |
| 217 | Vascular hamartomas and non-neoplastic nevi | neoplasms | 178.42 | 0.12 | Fixed-effect model | 1.06 | 0.86 - 1.32 | 0.58 |
| 217.1 | Nevus, non-neoplastic | neoplasms | 177.76 | 0.12 | Fixed-effect model | 1.08 | 0.86 - 1.36 | 0.49 |
| 225 | Benign neoplasm of brain and other parts of nervous system | neoplasms | 189.88 | 0.04 | Random effect model | 1.22 | 0.99 - 1.50 | 0.07 |
| 225.1 | Benign neoplasm of brain, cranial nerves, meninges | neoplasms | 194.74 | 0.02 | Random effect model | 1.22 | 0.98 - 1.52 | 0.08 |
| 227 | Benign neoplasm of other endocrine glands and related structures | neoplasms | 149.19 | 0.66 | Fixed-effect model | 1.17 | 0.97 - 1.41 | 0.09 |
| 228 | Hemangioma and lymphangioma, any site | neoplasms | 174.97 | 0.15 | Fixed-effect model | 0.91 | 0.79 - 1.04 | 0.16 |
| 229 | Benign neoplasm of unspecified sites | neoplasms | 134.33 | 0.90 | Fixed-effect model | 1.13 | 1.01 - 1.26 | 0.04 |
| 240 | Simple and unspecified goiter | endocrine/metabolic | 154.99 | 0.53 | Fixed-effect model | 0.92 | 0.73 - 1.15 | 0.46 |
| 241 | Nontoxic nodular goiter | endocrine/metabolic | 188.14 | 0.05 | Random effect model | 0.95 | 0.80 - 1.14 | 0.60 |
| 241.2 | Nontoxic multinodular goiter | endocrine/metabolic | 178.09 | 0.12 | Fixed-effect model | 0.73 | 0.59 - 0.90 | 3.81E-03 |
| 244 | Hypothyroidism | endocrine/metabolic | 635.38 | 8.88E-59 | Random effect model | 0.96 | 0.87 - 1.05 | 0.35 |
| 244.1 | Secondary hypothyroidism | endocrine/metabolic | 185.60 | 0.06 | Fixed-effect model | 0.95 | 0.81 - 1.13 | 0.58 |
| 244.4 | Hypothyroidism NOS | endocrine/metabolic | 602.03 | 2.41E-53 | Random effect model | 0.96 | 0.87 - 1.05 | 0.36 |
| 250 | Diabetes mellitus | endocrine/metabolic | 437.55 | 2.62E-28 | Random effect model | 0.85 | 0.79 - 0.91 | 7.03E-06 |
| 250.1 | Type 1 diabetes | endocrine/metabolic | 168.77 | 0.25 | Fixed-effect model | 0.87 | 0.78 - 0.97 | 0.01 |
| 250.2 | Type 2 diabetes | endocrine/metabolic | 430.60 | 2.47E-27 | Random effect model | 0.84 | 0.78 - 0.90 | 2.27E-06 |
| 250.23 | Type 2 diabetes with ophthalmic manifestations | endocrine/metabolic | 201.95 | 0.01 | Random effect model | 0.85 | 0.71 - 1.01 | 0.06 |
| 250.24 | Type 2 diabetes with neurological manifestations | endocrine/metabolic | 176.34 | 0.14 | Fixed-effect model | 0.89 | 0.71 - 1.12 | 0.33 |
| 250.4 | Abnormal glucose | endocrine/metabolic | 185.41 | 0.06 | Fixed-effect model | 0.83 | 0.68 - 1.03 | 0.09 |
| 250.7 | Diabetic retinopathy | endocrine/metabolic | 208.87 | 3.55E-03 | Random effect model | 0.82 | 0.68 - 0.97 | 0.02 |
| 251 | Other disorders of pancreatic internal secretion | endocrine/metabolic | 161.74 | 0.38 | Fixed-effect model | 0.72 | 0.60 - 0.86 | 2.91E-04 |
| 251.1 | Hypoglycemia | endocrine/metabolic | 161.51 | 0.39 | Fixed-effect model | 0.70 | 0.59 - 0.84 | 1.26E-04 |
| 252 | Disorders of parathyroid gland | endocrine/metabolic | 136.84 | 0.88 | Fixed-effect model | 1.23 | 1.02 - 1.48 | 0.03 |
| 252.1 | Hyperparathyroidism | endocrine/metabolic | 146.39 | 0.72 | Fixed-effect model | 1.13 | 0.93 - 1.38 | 0.22 |
| 253 | Disorders of the pituitary gland and its hypothalamic control | endocrine/metabolic | 179.58 | 0.10 | Fixed-effect model | 1.18 | 0.96 - 1.46 | 0.12 |
| 255 | Disorders of adrenal glands | endocrine/metabolic | 147.75 | 0.69 | Fixed-effect model | 1.06 | 0.85 - 1.32 | 0.59 |
| 260 | Protein-calorie malnutrition | endocrine/metabolic | 151.97 | 0.60 | Fixed-effect model | 0.77 | 0.65 - 0.91 | 2.39E-03 |
| 260.6 | Anorexia | endocrine/metabolic | 146.95 | 0.71 | Fixed-effect model | 0.76 | 0.63 - 0.92 | 4.28E-03 |
| 261 | Vitamin deficiency | endocrine/metabolic | 156.52 | 0.50 | Fixed-effect model | 1.00 | 0.85 - 1.17 | 0.99 |
| 261.2 | Vitamin B-complex deficiencies | endocrine/metabolic | 154.06 | 0.55 | Fixed-effect model | 0.98 | 0.80 - 1.20 | 0.84 |
| 272 | Disorders of lipid metabolism | endocrine/metabolic | 1740.34 | 6.96E-265 | Random effect model | 1.83 | 1.65 - 2.04 | 7.98E-28 |
| 272.1 | Hyperlipidemia | endocrine/metabolic | 1733.26 | 1.75E-263 | Random effect model | 1.83 | 1.64 - 2.04 | 7.78E-28 |
| 272.11 | Hypercholesterolemia | endocrine/metabolic | 1672.29 | 1.90E-251 | Random effect model | 1.85 | 1.66 - 2.07 | 1.42E-28 |
| 274 | Gout and other crystal arthropathies | endocrine/metabolic | 235.94 | 4.65E-05 | Random effect model | 0.90 | 0.80 - 1.00 | 0.06 |
| 274.1 | Gout | endocrine/metabolic | 257.78 | 7.02E-07 | Random effect model | 0.89 | 0.78 - 1.01 | 0.07 |
| 274.2 | Crystal arthropathies | endocrine/metabolic | 122.19 | 0.98 | Fixed-effect model | 0.94 | 0.75 - 1.17 | 0.56 |
| 274.21 | Chondrocalcinosis | endocrine/metabolic | 132.22 | 0.93 | Fixed-effect model | 0.93 | 0.74 - 1.18 | 0.56 |
| 275 | Disorders of mineral metabolism | endocrine/metabolic | 2217.50 | 0.00E+00 | Random effect model | 0.89 | 0.57 - 1.40 | 0.61 |
| 275.1 | Disorders of iron metabolism | hematopoietic | 584.67 | 3.93E-51 | Random effect model | 0.81 | 0.53 - 1.22 | 0.31 |
| 275.5 | Disorders of calcium/phosphorus metabolism | endocrine/metabolic | 163.71 | 0.34 | Fixed-effect model | 1.19 | 1.01 - 1.39 | 0.03 |
| 276 | Disorders of fluid, electrolyte, and acid-base balance | endocrine/metabolic | 200.21 | 0.01 | Random effect model | 1.02 | 0.95 - 1.10 | 0.59 |
| 276.1 | Electrolyte imbalance | endocrine/metabolic | 154.45 | 0.54 | Fixed-effect model | 1.02 | 0.94 - 1.11 | 0.61 |
| 276.13 | Hyperpotassemia | endocrine/metabolic | 153.78 | 0.56 | Fixed-effect model | 0.90 | 0.75 - 1.07 | 0.24 |
| 276.14 | Hypopotassemia | endocrine/metabolic | 149.95 | 0.64 | Fixed-effect model | 0.97 | 0.84 - 1.13 | 0.71 |
| 276.4 | Acid-base balance disorder | endocrine/metabolic | 145.49 | 0.74 | Fixed-effect model | 1.04 | 0.88 - 1.22 | 0.65 |
| 276.41 | Acidosis | endocrine/metabolic | 140.70 | 0.82 | Fixed-effect model | 1.05 | 0.89 - 1.24 | 0.58 |
| 276.5 | Hypovolemia | endocrine/metabolic | 181.08 | 0.09 | Fixed-effect model | 0.99 | 0.89 - 1.09 | 0.80 |
| 277 | Other disorders of metabolism | endocrine/metabolic | 173.03 | 0.18 | Fixed-effect model | 0.99 | 0.85 - 1.14 | 0.88 |
| 278 | Overweight, obesity and other hyperalimentation | endocrine/metabolic | 200.98 | 0.01 | Random effect model | 0.88 | 0.83 - 0.94 | 9.26E-05 |
| 278.1 | Obesity | endocrine/metabolic | 205.05 | 0.01 | Random effect model | 0.88 | 0.82 - 0.93 | 4.33E-05 |
| 280 | Iron deficiency anemias | hematopoietic | 175.23 | 0.15 | Fixed-effect model | 1.10 | 1.03 - 1.17 | 2.86E-03 |
| 280.1 | Iron deficiency anemias, unspecified or not due to blood loss | hematopoietic | 189.65 | 0.04 | Random effect model | 1.11 | 1.04 - 1.20 | 3.32E-03 |
| 281 | Other deficiency anemia | hematopoietic | 176.91 | 0.13 | Fixed-effect model | 1.24 | 1.05 - 1.46 | 0.01 |
| 281.1 | Megaloblastic anemia | hematopoietic | 177.45 | 0.13 | Fixed-effect model | 1.23 | 1.04 - 1.46 | 0.01 |
| 281.11 | Pernicious anemia | hematopoietic | 186.18 | 0.06 | Fixed-effect model | 1.24 | 1.02 - 1.52 | 0.03 |
| 285 | Other anemias | hematopoietic | 232.02 | 9.29E-05 | Random effect model | 1.04 | 0.98 - 1.11 | 0.19 |
| 285.2 | Anemia of chronic disease | hematopoietic | 143.17 | 0.78 | Fixed-effect model | 1.01 | 0.82 - 1.25 | 0.90 |
| 286 | Coagulation defects | hematopoietic | 160.38 | 0.41 | Fixed-effect model | 1.02 | 0.85 - 1.22 | 0.83 |
| 287 | Purpura and other hemorrhagic conditions | hematopoietic | 158.78 | 0.45 | Fixed-effect model | 1.02 | 0.90 - 1.17 | 0.72 |
| 287.3 | Thrombocytopenia | hematopoietic | 163.76 | 0.34 | Fixed-effect model | 1.04 | 0.90 - 1.19 | 0.62 |
| 288 | Diseases of white blood cells | hematopoietic | 175.46 | 0.15 | Fixed-effect model | 1.06 | 0.97 - 1.16 | 0.18 |
| 288.1 | Decreased white blood cell count | hematopoietic | 167.03 | 0.28 | Fixed-effect model | 1.03 | 0.93 - 1.13 | 0.61 |
| 288.11 | Neutropenia | hematopoietic | 167.03 | 0.28 | Fixed-effect model | 1.03 | 0.93 - 1.13 | 0.61 |
| 289 | Other diseases of blood and blood-forming organs | hematopoietic | 147.25 | 0.70 | Fixed-effect model | 0.90 | 0.82 - 0.98 | 0.01 |
| 289.4 | Lymphadenitis | hematopoietic | 163.41 | 0.35 | Fixed-effect model | 0.91 | 0.82 - 1.02 | 0.09 |
| 289.5 | Diseases of spleen | hematopoietic | 118.07 | 0.99 | Fixed-effect model | 0.80 | 0.63 - 1.02 | 0.07 |
| 290.1 | Dementias | mental disorders | 746.07 | 1.98E-77 | Random effect model | 2.06 | 1.39 - 3.04 | 3.07E-04 |
| 291 | Other specified nonpsychotic and/or transient mental disorders | mental disorders | 173.07 | 0.18 | Fixed-effect model | 0.97 | 0.78 - 1.20 | 0.75 |
| 292 | Neurological disorders | mental disorders | 261.43 | 3.30E-07 | Random effect model | 1.13 | 1.02 - 1.25 | 0.02 |
| 292.1 | Aphasia/speech disturbance | mental disorders | 192.99 | 0.03 | Random effect model | 1.28 | 1.09 - 1.50 | 2.13E-03 |
| 292.3 | Memory loss | mental disorders | 156.48 | 0.50 | Fixed-effect model | 0.96 | 0.78 - 1.18 | 0.69 |
| 292.4 | Altered mental status | mental disorders | 272.53 | 3.05E-08 | Random effect model | 1.15 | 0.98 - 1.34 | 0.08 |
| 293 | Symptoms involving head and neck | mental disorders | 159.70 | 0.42 | Fixed-effect model | 1.03 | 0.93 - 1.14 | 0.60 |
| 293.1 | Swelling, mass, or lump in head and neck [Space occupying lesion, intracranial NOS] | mental disorders | 157.98 | 0.46 | Fixed-effect model | 1.15 | 0.96 - 1.38 | 0.14 |
| 295 | Schizophrenia and other psychotic disorders | mental disorders | 166.11 | 0.29 | Fixed-effect model | 1.08 | 0.90 - 1.31 | 0.40 |
| 295.1 | Schizophrenia | mental disorders | 161.35 | 0.39 | Fixed-effect model | 1.01 | 0.81 - 1.28 | 0.90 |
| 296 | Mood disorders | mental disorders | 170.63 | 0.22 | Fixed-effect model | 0.97 | 0.92 - 1.02 | 0.29 |
| 296.1 | Bipolar | mental disorders | 175.21 | 0.15 | Fixed-effect model | 0.87 | 0.73 - 1.03 | 0.10 |
| 296.2 | Depression | mental disorders | 166.69 | 0.28 | Fixed-effect model | 0.97 | 0.92 - 1.02 | 0.29 |
| 300 | Anxiety disorders | mental disorders | 191.14 | 0.03 | Random effect model | 0.96 | 0.89 - 1.04 | 0.32 |
| 300.1 | Anxiety disorder | mental disorders | 197.27 | 0.02 | Random effect model | 0.96 | 0.89 - 1.04 | 0.33 |
| 300.12 | Agorophobia, social phobia, and panic disorder | mental disorders | 160.81 | 0.40 | Fixed-effect model | 1.04 | 0.84 - 1.27 | 0.74 |
| 300.13 | Phobia | mental disorders | 159.27 | 0.43 | Fixed-effect model | 1.39 | 1.09 - 1.78 | 0.01 |
| 303 | Psychogenic and somatoform disorders | mental disorders | 141.17 | 0.81 | Fixed-effect model | 1.27 | 1.00 - 1.61 | 0.05 |
| 306 | Other mental disorder | mental disorders | 195.15 | 0.02 | Random effect model | 0.99 | 0.96 - 1.03 | 0.74 |
| 317 | Alcohol-related disorders | mental disorders | 236.48 | 4.23E-05 | Random effect model | 1.00 | 0.94 - 1.06 | 0.92 |
| 317.1 | Alcoholism | mental disorders | 232.55 | 8.48E-05 | Random effect model | 1.01 | 0.94 - 1.08 | 0.82 |
| 317.11 | Alcoholic liver damage | mental disorders | 171.66 | 0.20 | Fixed-effect model | 1.06 | 0.88 - 1.29 | 0.53 |
| 318 | Tobacco use disorder | mental disorders | 195.78 | 0.02 | Random effect model | 0.98 | 0.93 - 1.02 | 0.30 |
| 327 | Sleep disorders | neurological | 185.23 | 0.06 | Fixed-effect model | 0.90 | 0.84 - 0.98 | 0.01 |
| 327.3 | Sleep apnea | neurological | 182.78 | 0.08 | Fixed-effect model | 0.94 | 0.86 - 1.02 | 0.12 |
| 331 | Other cerebral degenerations | neurological | 174.93 | 0.16 | Fixed-effect model | 1.20 | 1.02 - 1.42 | 0.03 |
| 332 | Parkinson's disease | neurological | 147.22 | 0.70 | Fixed-effect model | 0.93 | 0.79 - 1.09 | 0.38 |
| 333 | Extrapyramidal disease and abnormal movement disorders | neurological | 188.79 | 0.04 | Random effect model | 0.87 | 0.71 - 1.06 | 0.17 |
| 334 | Degenerative disease of the spinal cord | neurological | 145.64 | 0.73 | Fixed-effect model | 0.97 | 0.86 - 1.11 | 0.69 |
| 335 | Multiple sclerosis | neurological | 180.75 | 0.09 | Fixed-effect model | 0.90 | 0.77 - 1.04 | 0.16 |
| 338 | Pain | symptoms | 151.73 | 0.60 | Fixed-effect model | 0.83 | 0.68 - 1.01 | 0.06 |
| 339 | Other headache syndromes | neurological | 169.11 | 0.24 | Fixed-effect model | 0.93 | 0.87 - 0.99 | 0.03 |
| 340 | Migraine | neurological | 198.24 | 0.01 | Random effect model | 0.92 | 0.82 - 1.03 | 0.16 |
| 342 | Hemiplegia | neurological | 195.80 | 0.02 | Random effect model | 1.01 | 0.86 - 1.19 | 0.89 |
| 344 | Other paralytic syndromes | neurological | 172.61 | 0.19 | Fixed-effect model | 0.89 | 0.72 - 1.11 | 0.30 |
| 345 | Epilepsy, recurrent seizures, convulsions | neurological | 171.12 | 0.21 | Fixed-effect model | 1.01 | 0.93 - 1.09 | 0.89 |
| 345.1 | Epilepsy | neurological | 175.52 | 0.15 | Fixed-effect model | 0.92 | 0.77 - 1.11 | 0.39 |
| 345.3 | Convulsions | neurological | 176.75 | 0.13 | Fixed-effect model | 1.05 | 0.93 - 1.17 | 0.46 |
| 348 | Other conditions of brain | neurological | 135.05 | 0.90 | Fixed-effect model | 1.03 | 0.89 - 1.20 | 0.65 |
| 350 | Abnormal movement | neurological | 170.24 | 0.22 | Fixed-effect model | 0.94 | 0.85 - 1.04 | 0.25 |
| 350.1 | Abnormal involuntary movements | neurological | 156.24 | 0.50 | Fixed-effect model | 1.07 | 0.89 - 1.29 | 0.45 |
| 350.2 | Abnormality of gait | neurological | 133.85 | 0.91 | Fixed-effect model | 0.90 | 0.78 - 1.03 | 0.13 |
| 351 | Other peripheral nerve disorders | neurological | 196.99 | 0.02 | Random effect model | 0.99 | 0.93 - 1.04 | 0.64 |
| 352 | Disorders of other cranial nerves | neurological | 165.15 | 0.31 | Fixed-effect model | 0.97 | 0.84 - 1.13 | 0.69 |
| 352.2 | Facial nerve disorders [CN7] | neurological | 138.80 | 0.85 | Fixed-effect model | 0.98 | 0.82 - 1.18 | 0.85 |
| 353 | Nerve root and plexus disorders | neurological | 174.82 | 0.16 | Fixed-effect model | 0.88 | 0.74 - 1.03 | 0.11 |
| 357 | Inflammatory and toxic neuropathy | neurological | 148.47 | 0.67 | Fixed-effect model | 0.86 | 0.74 - 1.00 | 0.05 |
| 361 | Retinal detachments and defects | sense organs | 177.14 | 0.13 | Fixed-effect model | 0.99 | 0.89 - 1.09 | 0.78 |
| 361.1 | Retinal detachment with retinal defect | sense organs | 140.07 | 0.83 | Fixed-effect model | 1.05 | 0.90 - 1.21 | 0.56 |
| 362 | Other retinal disorders | sense organs | 154.06 | 0.55 | Fixed-effect model | 1.01 | 0.92 - 1.10 | 0.91 |
| 362.2 | Degeneration of macula and posterior pole of retina | sense organs | 162.88 | 0.36 | Fixed-effect model | 1.04 | 0.93 - 1.17 | 0.50 |
| 362.29 | Macular degeneration (senile) of retina NOS | sense organs | 164.35 | 0.33 | Fixed-effect model | 1.04 | 0.92 - 1.17 | 0.55 |
| 362.4 | Retinal vascular changes and abnomalities | sense organs | 150.83 | 0.62 | Fixed-effect model | 0.94 | 0.78 - 1.13 | 0.50 |
| 364 | Corneal opacity and other disorders of cornea | sense organs | 153.29 | 0.57 | Fixed-effect model | 0.98 | 0.80 - 1.20 | 0.84 |
| 365 | Glaucoma | sense organs | 210.24 | 2.92E-03 | Random effect model | 0.97 | 0.88 - 1.07 | 0.49 |
| 365.1 | Open-angle glaucoma | sense organs | 164.10 | 0.33 | Fixed-effect model | 0.91 | 0.77 - 1.08 | 0.30 |
| 365.11 | Primary open angle glaucoma | sense organs | 164.50 | 0.32 | Fixed-effect model | 0.91 | 0.77 - 1.08 | 0.29 |
| 365.2 | Primary angle-closure glaucoma | sense organs | 188.85 | 0.04 | Random effect model | 1.12 | 0.90 - 1.41 | 0.31 |
| 366 | Cataract | sense organs | 187.85 | 0.05 | Random effect model | 1.03 | 0.99 - 1.08 | 0.17 |
| 366.2 | Senile cataract | sense organs | 174.99 | 0.15 | Fixed-effect model | 1.00 | 0.94 - 1.06 | 0.92 |
| 367 | Disorders of refraction and accommodation; blindness and low vision | sense organs | 139.98 | 0.83 | Fixed-effect model | 0.96 | 0.86 - 1.07 | 0.45 |
| 367.1 | Myopia | sense organs | 141.06 | 0.81 | Fixed-effect model | 0.88 | 0.75 - 1.02 | 0.10 |
| 367.9 | Blindness and low vision | sense organs | 157.76 | 0.47 | Fixed-effect model | 1.07 | 0.87 - 1.32 | 0.50 |
| 368 | Visual disturbances | sense organs | 138.73 | 0.85 | Fixed-effect model | 0.86 | 0.79 - 0.95 | 2.97E-03 |
| 368.1 | Amblyopia | sense organs | 127.91 | 0.96 | Fixed-effect model | 0.98 | 0.77 - 1.24 | 0.85 |
| 368.2 | Diplopia and disorders of binocular vision | sense organs | 145.64 | 0.73 | Fixed-effect model | 0.78 | 0.64 - 0.96 | 0.02 |
| 368.9 | Subjective visual disturbances | sense organs | 180.31 | 0.10 | Fixed-effect model | 0.84 | 0.68 - 1.05 | 0.12 |
| 369 | Infection of the eye | sense organs | 146.41 | 0.72 | Fixed-effect model | 1.12 | 0.89 - 1.41 | 0.34 |
| 371 | Inflammation of the eye | sense organs | 188.15 | 0.05 | Random effect model | 0.96 | 0.86 - 1.07 | 0.44 |
| 371.3 | Inflammation of eyelids | sense organs | 202.31 | 0.01 | Random effect model | 1.04 | 0.92 - 1.18 | 0.53 |
| 372 | Disorders of conjunctiva | sense organs | 144.49 | 0.75 | Fixed-effect model | 0.80 | 0.65 - 0.97 | 0.03 |
| 374 | Other disorders of eyelids | sense organs | 140.11 | 0.83 | Fixed-effect model | 1.07 | 0.99 - 1.15 | 0.09 |
| 374.1 | Ectropion or entropion | sense organs | 161.45 | 0.39 | Fixed-effect model | 0.99 | 0.84 - 1.17 | 0.90 |
| 375 | Disorders of lacrimal system | sense organs | 150.71 | 0.63 | Fixed-effect model | 0.90 | 0.80 - 1.02 | 0.09 |
| 375.2 | Epiphora | sense organs | 149.67 | 0.65 | Fixed-effect model | 0.75 | 0.63 - 0.90 | 2.41E-03 |
| 378 | Strabismus and other disorders of binocular eye movements | sense organs | 139.83 | 0.83 | Fixed-effect model | 0.93 | 0.81 - 1.08 | 0.36 |
| 378.1 | Strabismus (not specified as paralytic) | sense organs | 135.61 | 0.89 | Fixed-effect model | 0.89 | 0.74 - 1.06 | 0.20 |
| 379 | Other disorders of eye | sense organs | 181.11 | 0.09 | Fixed-effect model | 0.99 | 0.91 - 1.09 | 0.89 |
| 379.2 | Disorders of vitreous body | sense organs | 170.58 | 0.22 | Fixed-effect model | 1.13 | 0.98 - 1.31 | 0.10 |
| 379.3 | Aphakia and other disorders of lens | sense organs | 148.10 | 0.68 | Fixed-effect model | 0.86 | 0.76 - 0.98 | 0.03 |
| 380 | Disorders of external ear | sense organs | 167.02 | 0.28 | Fixed-effect model | 0.87 | 0.75 - 1.00 | 0.05 |
| 380.1 | Otitis externa | sense organs | 168.94 | 0.24 | Fixed-effect model | 1.04 | 0.82 - 1.33 | 0.72 |
| 381 | Otitis media and Eustachian tube disorders | sense organs | 141.33 | 0.81 | Fixed-effect model | 0.74 | 0.66 - 0.84 | 5.94E-07 |
| 381.1 | Otitis media | sense organs | 161.63 | 0.38 | Fixed-effect model | 0.73 | 0.64 - 0.83 | 2.10E-06 |
| 381.11 | Suppurative and unspecified otitis media | sense organs | 145.60 | 0.73 | Fixed-effect model | 0.69 | 0.57 - 0.83 | 9.29E-05 |
| 384 | Other disorders of tympanic membrane | sense organs | 156.07 | 0.51 | Fixed-effect model | 0.75 | 0.65 - 0.87 | 1.56E-04 |
| 384.4 | Perforation of tympanic membrane | sense organs | 144.75 | 0.75 | Fixed-effect model | 0.78 | 0.65 - 0.92 | 3.41E-03 |
| 385 | Other disorders of middle ear and mastoid | sense organs | 144.66 | 0.75 | Fixed-effect model | 0.80 | 0.66 - 0.97 | 0.02 |
| 385.3 | Cholesteatoma | sense organs | 144.09 | 0.76 | Fixed-effect model | 0.71 | 0.56 - 0.88 | 2.33E-03 |
| 386 | Vertiginous syndromes and other disorders of vestibular system | sense organs | 186.59 | 0.05 | Fixed-effect model | 0.91 | 0.85 - 0.98 | 0.01 |
| 386.1 | Meniere's disease | sense organs | 173.34 | 0.18 | Fixed-effect model | 1.06 | 0.84 - 1.33 | 0.62 |
| 386.3 | Labyrinthitis | sense organs | 118.58 | 0.99 | Fixed-effect model | 0.84 | 0.69 - 1.03 | 0.10 |
| 386.9 | Dizziness and giddiness (Light-headedness and vertigo) | sense organs | 187.57 | 0.05 | Random effect model | 0.92 | 0.84 - 1.00 | 0.06 |
| 389 | Hearing loss | sense organs | 177.77 | 0.12 | Fixed-effect model | 0.97 | 0.89 - 1.05 | 0.46 |
| 389.4 | Tinnitus | sense organs | 160.43 | 0.41 | Fixed-effect model | 1.08 | 0.85 - 1.38 | 0.52 |
| 394 | Rheumatic disease of the heart valves | circulatory system | 211.99 | 2.27E-03 | Random effect model | 0.95 | 0.87 - 1.04 | 0.28 |
| 394.2 | Mitral valve disease | circulatory system | 168.08 | 0.26 | Fixed-effect model | 0.99 | 0.89 - 1.09 | 0.81 |
| 394.3 | Aortic valve disease | circulatory system | 187.37 | 0.05 | Random effect model | 0.82 | 0.69 - 0.97 | 0.02 |
| 394.7 | Disease of tricuspid valve | circulatory system | 126.13 | 0.97 | Fixed-effect model | 1.11 | 0.94 - 1.31 | 0.23 |
| 395 | Heart valve disorders | circulatory system | 194.36 | 0.02 | Random effect model | 1.00 | 0.91 - 1.10 | 0.95 |
| 395.1 | Nonrheumatic mitral valve disorders | circulatory system | 170.14 | 0.22 | Fixed-effect model | 1.01 | 0.91 - 1.12 | 0.85 |
| 395.6 | Heart valve replaced | circulatory system | 205.48 | 0.01 | Random effect model | 1.01 | 0.86 - 1.19 | 0.88 |
| 396 | Abnormal heart sounds | circulatory system | 202.20 | 0.01 | Random effect model | 0.99 | 0.82 - 1.20 | 0.94 |
| 401 | Hypertension | circulatory system | 659.26 | 9.99E-63 | Random effect model | 1.01 | 0.96 - 1.06 | 0.72 |
| 401.1 | Essential hypertension | circulatory system | 655.43 | 4.32E-62 | Random effect model | 1.01 | 0.96 - 1.06 | 0.76 |
| 401.2 | Hypertensive heart and/or renal disease | circulatory system | 161.41 | 0.39 | Fixed-effect model | 0.91 | 0.79 - 1.04 | 0.15 |
| 401.22 | Hypertensive chronic kidney disease | circulatory system | 165.34 | 0.31 | Fixed-effect model | 0.90 | 0.79 - 1.04 | 0.16 |
| 402 | Elevated blood pressure reading without diagnosis of hypertension | circulatory system | 164.69 | 0.32 | Fixed-effect model | 1.16 | 1.00 - 1.34 | 0.04 |
| 411 | Ischemic Heart Disease | circulatory system | 927.20 | 1.82E-109 | Random effect model | 1.28 | 1.18 - 1.40 | 9.17E-09 |
| 411.1 | Unstable angina (intermediate coronary syndrome) | circulatory system | 349.36 | 1.13E-16 | Random effect model | 1.32 | 1.18 - 1.49 | 2.26E-06 |
| 411.2 | Myocardial infarction | circulatory system | 668.66 | 2.71E-64 | Random effect model | 1.36 | 1.21 - 1.51 | 7.38E-08 |
| 411.3 | Angina pectoris | circulatory system | 633.38 | 1.89E-58 | Random effect model | 1.35 | 1.23 - 1.49 | 2.10E-10 |
| 411.4 | Coronary atherosclerosis | circulatory system | 990.07 | 6.47E-121 | Random effect model | 1.40 | 1.26 - 1.56 | 5.75E-10 |
| 411.41 | Aneurysm and dissection of heart | circulatory system | 171.94 | 0.20 | Fixed-effect model | 0.98 | 0.80 - 1.21 | 0.85 |
| 411.8 | Other chronic ischemic heart disease, unspecified | circulatory system | 577.32 | 2.21E-49 | Random effect model | 1.35 | 1.23 - 1.48 | 2.69E-10 |
| 411.9 | Other acute and subacute forms of ischemic heart disease | circulatory system | 180.33 | 0.10 | Fixed-effect model | 1.20 | 1.02 - 1.41 | 0.03 |
| 414 | Other forms of chronic heart disease | circulatory system | 135.31 | 0.89 | Fixed-effect model | 1.22 | 1.07 - 1.39 | 2.52E-03 |
| 415 | Pulmonary heart disease | circulatory system | 200.17 | 0.01 | Random effect model | 0.84 | 0.76 - 0.92 | 2.82E-04 |
| 415.2 | Chronic pulmonary heart disease | circulatory system | 154.98 | 0.53 | Fixed-effect model | 0.76 | 0.61 - 0.96 | 0.02 |
| 416 | Cardiomegaly | circulatory system | 171.17 | 0.21 | Fixed-effect model | 1.05 | 0.94 - 1.17 | 0.41 |
| 418 | Nonspecific chest pain | circulatory system | 226.54 | 2.36E-04 | Random effect model | 1.05 | 1.01 - 1.09 | 0.01 |
| 418.1 | Precordial pain | circulatory system | 169.05 | 0.24 | Fixed-effect model | 1.00 | 0.91 - 1.10 | 0.96 |
| 420.2 | Pericarditis | circulatory system | 166.19 | 0.29 | Fixed-effect model | 1.23 | 1.05 - 1.44 | 0.01 |
| 420.3 | Endocarditis | circulatory system | 164.62 | 0.32 | Fixed-effect model | 0.87 | 0.71 - 1.07 | 0.19 |
| 425 | Cardiomyopathy | circulatory system | 163.94 | 0.34 | Fixed-effect model | 0.90 | 0.77 - 1.05 | 0.20 |
| 425.1 | Primary/intrinsic cardiomyopathies | circulatory system | 150.48 | 0.63 | Fixed-effect model | 0.88 | 0.75 - 1.03 | 0.10 |
| 426 | Cardiac conduction disorders | circulatory system | 229.38 | 1.46E-04 | Random effect model | 0.99 | 0.91 - 1.08 | 0.84 |
| 426.2 | Atrioventricular [AV] block | circulatory system | 148.66 | 0.67 | Fixed-effect model | 0.99 | 0.88 - 1.12 | 0.93 |
| 426.21 | First degree AV block | circulatory system | 161.87 | 0.38 | Fixed-effect model | 1.11 | 0.93 - 1.33 | 0.23 |
| 426.24 | Atrioventricular block, complete | circulatory system | 183.55 | 0.07 | Fixed-effect model | 0.94 | 0.75 - 1.18 | 0.58 |
| 426.3 | Bundle branch block | circulatory system | 210.89 | 2.67E-03 | Random effect model | 0.99 | 0.89 - 1.11 | 0.92 |
| 426.31 | Right bundle branch block | circulatory system | 187.79 | 0.05 | Random effect model | 1.01 | 0.86 - 1.18 | 0.93 |
| 426.32 | Left bundle branch block | circulatory system | 154.41 | 0.54 | Fixed-effect model | 0.97 | 0.85 - 1.10 | 0.61 |
| 426.9 | Cardiac pacemaker/device in situ | circulatory system | 183.72 | 0.07 | Fixed-effect model | 1.00 | 0.90 - 1.12 | 0.96 |
| 426.91 | Cardiac pacemaker in situ | circulatory system | 191.02 | 0.03 | Random effect model | 1.04 | 0.92 - 1.18 | 0.54 |
| 427 | Cardiac dysrhythmias | circulatory system | 219.05 | 7.87E-04 | Random effect model | 1.04 | 1.00 - 1.09 | 0.06 |
| 427.1 | Paroxysmal tachycardia, unspecified | circulatory system | 147.22 | 0.70 | Fixed-effect model | 0.97 | 0.88 - 1.07 | 0.58 |
| 427.11 | Paroxysmal supraventricular tachycardia | circulatory system | 160.77 | 0.40 | Fixed-effect model | 0.97 | 0.86 - 1.08 | 0.55 |
| 427.12 | Paroxysmal ventricular tachycardia | circulatory system | 131.43 | 0.93 | Fixed-effect model | 1.05 | 0.88 - 1.26 | 0.60 |
| 427.2 | Atrial fibrillation and flutter | circulatory system | 275.29 | 1.66E-08 | Random effect model | 1.06 | 1.00 - 1.13 | 0.06 |
| 427.3 | Other specified cardiac dysrhythmias | circulatory system | 192.53 | 0.03 | Random effect model | 1.18 | 1.06 - 1.31 | 3.14E-03 |
| 427.4 | Cardiac arrest and ventricular fibrillation | circulatory system | 157.34 | 0.48 | Fixed-effect model | 1.06 | 0.90 - 1.25 | 0.46 |
| 427.42 | Cardiac arrest | circulatory system | 187.40 | 0.05 | Random effect model | 1.08 | 0.89 - 1.31 | 0.45 |
| 427.5 | Arrhythmia (cardiac) NOS | circulatory system | 165.38 | 0.31 | Fixed-effect model | 0.91 | 0.76 - 1.09 | 0.28 |
| 427.6 | Premature beats | circulatory system | 157.68 | 0.47 | Fixed-effect model | 1.17 | 0.92 - 1.48 | 0.21 |
| 427.7 | Tachycardia NOS | circulatory system | 174.04 | 0.17 | Fixed-effect model | 0.89 | 0.79 - 1.00 | 0.05 |
| 427.9 | Palpitations | circulatory system | 151.63 | 0.61 | Fixed-effect model | 1.09 | 1.00 - 1.19 | 0.06 |
| 428 | Congestive heart failure; nonhypertensive | circulatory system | 184.87 | 0.06 | Fixed-effect model | 1.05 | 0.97 - 1.13 | 0.21 |
| 428.2 | Heart failure NOS | circulatory system | 210.49 | 2.82E-03 | Random effect model | 1.10 | 0.99 - 1.21 | 0.07 |
| 429 | Ill-defined descriptions and complications of heart disease | circulatory system | 155.32 | 0.52 | Fixed-effect model | 1.22 | 1.00 - 1.48 | 0.05 |
| 429.2 | Abnormal function study of cardiovascular system | circulatory system | 154.36 | 0.54 | Fixed-effect model | 1.06 | 0.83 - 1.35 | 0.63 |
| 430 | Intracranial hemorrhage | circulatory system | 150.79 | 0.62 | Fixed-effect model | 0.88 | 0.78 - 1.01 | 0.06 |
| 430.1 | Subarachnoid hemorrhage | circulatory system | 145.90 | 0.73 | Fixed-effect model | 0.93 | 0.77 - 1.13 | 0.47 |
| 430.2 | Intracerebral hemorrhage | circulatory system | 160.90 | 0.40 | Fixed-effect model | 0.74 | 0.60 - 0.91 | 4.57E-03 |
| 433 | Cerebrovascular disease | circulatory system | 214.96 | 1.47E-03 | Random effect model | 1.13 | 1.05 - 1.21 | 7.40E-04 |
| 433.1 | Occlusion and stenosis of precerebral arteries | circulatory system | 231.25 | 1.06E-04 | Random effect model | 1.18 | 0.97 - 1.43 | 0.10 |
| 433.2 | Occlusion of cerebral arteries | circulatory system | 219.99 | 6.79E-04 | Random effect model | 1.19 | 1.07 - 1.32 | 1.01E-03 |
| 433.21 | Cerebral artery occlusion, with cerebral infarction | circulatory system | 152.38 | 0.59 | Fixed-effect model | 0.88 | 0.76 - 1.01 | 0.08 |
| 433.3 | Cerebral ischemia | circulatory system | 181.23 | 0.09 | Fixed-effect model | 1.05 | 0.95 - 1.16 | 0.38 |
| 433.31 | Transient cerebral ischemia | circulatory system | 175.97 | 0.14 | Fixed-effect model | 1.18 | 1.05 - 1.33 | 0.01 |
| 433.8 | Late effects of cerebrovascular disease | circulatory system | 190.22 | 0.04 | Random effect model | 1.01 | 0.85 - 1.19 | 0.95 |
| 440 | Atherosclerosis | circulatory system | 203.26 | 0.01 | Random effect model | 1.13 | 0.95 - 1.34 | 0.16 |
| 440.2 | Atherosclerosis of the extremities | circulatory system | 202.06 | 0.01 | Random effect model | 0.97 | 0.78 - 1.21 | 0.78 |
| 441 | Vascular insufficiency of intestine | circulatory system | 165.00 | 0.32 | Fixed-effect model | 0.68 | 0.54 - 0.86 | 1.12E-03 |
| 442 | Other aneurysm | circulatory system | 222.53 | 4.54E-04 | Random effect model | 1.02 | 0.87 - 1.19 | 0.83 |
| 442.1 | Aortic aneurysm | circulatory system | 221.60 | 5.27E-04 | Random effect model | 1.08 | 0.90 - 1.28 | 0.42 |
| 442.11 | Abdominal aortic aneurysm | circulatory system | 256.20 | 9.69E-07 | Random effect model | 1.12 | 0.89 - 1.42 | 0.33 |
| 443 | Peripheral vascular disease | circulatory system | 269.23 | 6.27E-08 | Random effect model | 1.10 | 0.98 - 1.23 | 0.12 |
| 443.1 | Raynaud's syndrome | circulatory system | 202.24 | 0.01 | Random effect model | 1.10 | 0.91 - 1.32 | 0.33 |
| 443.9 | Peripheral vascular disease, unspecified | circulatory system | 266.36 | 1.17E-07 | Random effect model | 1.13 | 0.98 - 1.30 | 0.10 |
| 444 | Arterial embolism and thrombosis | circulatory system | 204.14 | 0.01 | Random effect model | 0.90 | 0.73 - 1.11 | 0.32 |
| 444.1 | Arterial embolism and thrombosis of lower extremity artery | circulatory system | 186.49 | 0.05 | Fixed-effect model | 0.81 | 0.64 - 1.03 | 0.08 |
| 446 | Polyarteritis nodosa and allied conditions | circulatory system | 174.00 | 0.17 | Fixed-effect model | 1.31 | 1.08 - 1.58 | 0.01 |
| 447 | Other disorders of arteries and arterioles | circulatory system | 146.12 | 0.72 | Fixed-effect model | 1.03 | 0.89 - 1.20 | 0.67 |
| 447.1 | Stricture of artery | circulatory system | 149.13 | 0.66 | Fixed-effect model | 1.07 | 0.89 - 1.29 | 0.47 |
| 450 | Noninfectious disorders of lymphatic channels | circulatory system | 134.35 | 0.90 | Fixed-effect model | 1.04 | 0.84 - 1.28 | 0.73 |
| 451 | Phlebitis and thrombophlebitis | circulatory system | 234.03 | 6.54E-05 | Random effect model | 0.87 | 0.78 - 0.97 | 0.01 |
| 451.2 | Phlebitis and thrombophlebitis of lower extremities | circulatory system | 230.36 | 1.24E-04 | Random effect model | 0.83 | 0.74 - 0.93 | 1.38E-03 |
| 452 | Other venous embolism and thrombosis | circulatory system | 176.96 | 0.13 | Fixed-effect model | 0.61 | 0.48 - 0.77 | 3.38E-05 |
| 454 | Varicose veins | circulatory system | 282.28 | 3.41E-09 | Random effect model | 0.98 | 0.91 - 1.05 | 0.55 |
| 454.1 | Varicose veins of lower extremity | circulatory system | 271.01 | 4.26E-08 | Random effect model | 0.98 | 0.92 - 1.05 | 0.64 |
| 454.11 | Varicose veins of lower extremity, symptomtic | circulatory system | 159.82 | 0.42 | Fixed-effect model | 1.00 | 0.81 - 1.25 | 0.98 |
| 455 | Hemorrhoids | circulatory system | 230.68 | 1.17E-04 | Random effect model | 1.01 | 0.96 - 1.06 | 0.71 |
| 458 | Hypotension | circulatory system | 146.57 | 0.71 | Fixed-effect model | 1.03 | 0.96 - 1.11 | 0.45 |
| 458.1 | Orthostatic hypotension | circulatory system | 156.10 | 0.51 | Fixed-effect model | 1.14 | 0.98 - 1.32 | 0.09 |
| 458.9 | Hypotension NOS | circulatory system | 158.53 | 0.45 | Fixed-effect model | 1.03 | 0.94 - 1.13 | 0.52 |
| 459 | Other disorders of circulatory system | circulatory system | 286.87 | 1.18E-09 | Random effect model | 1.01 | 0.95 - 1.07 | 0.74 |
| 459.9 | Circulatory disease NEC | circulatory system | 283.15 | 2.79E-09 | Random effect model | 1.01 | 0.95 - 1.07 | 0.77 |
| 465 | Acute upper respiratory infections of multiple or unspecified sites | respiratory | 156.63 | 0.49 | Fixed-effect model | 1.09 | 0.97 - 1.22 | 0.14 |
| 465.2 | Acute pharyngitis | respiratory | 172.40 | 0.19 | Fixed-effect model | 1.24 | 1.03 - 1.49 | 0.02 |
| 470 | Septal Deviations/Turbinate Hypertrophy | respiratory | 179.30 | 0.11 | Fixed-effect model | 1.07 | 0.99 - 1.15 | 0.11 |
| 471 | Nasal polyps | respiratory | 164.37 | 0.33 | Fixed-effect model | 1.23 | 1.12 - 1.36 | 1.98E-05 |
| 472 | Chronic pharyngitis and nasopharyngitis | respiratory | 171.11 | 0.21 | Fixed-effect model | 1.10 | 0.92 - 1.32 | 0.28 |
| 473 | Diseases of the larynx and vocal cords | respiratory | 168.52 | 0.25 | Fixed-effect model | 0.96 | 0.87 - 1.07 | 0.51 |
| 473.4 | Voice disturbance | respiratory | 160.30 | 0.41 | Fixed-effect model | 1.01 | 0.86 - 1.20 | 0.89 |
| 474.1 | Acute tonsillitis | respiratory | 174.29 | 0.16 | Fixed-effect model | 0.86 | 0.69 - 1.07 | 0.17 |
| 474.2 | Chronic tonsillitis and adenoiditis | respiratory | 167.08 | 0.28 | Fixed-effect model | 0.81 | 0.69 - 0.96 | 0.01 |
| 475 | Chronic sinusitis | respiratory | 175.36 | 0.15 | Fixed-effect model | 0.98 | 0.88 - 1.09 | 0.66 |
| 476 | Allergic rhinitis | respiratory | 126.89 | 0.96 | Fixed-effect model | 0.86 | 0.72 - 1.01 | 0.07 |
| 477 | Epistaxis or throat hemorrhage | respiratory | 190.50 | 0.04 | Random effect model | 0.97 | 0.86 - 1.10 | 0.67 |
| 479 | Other upper respiratory disease | respiratory | 156.64 | 0.49 | Fixed-effect model | 0.95 | 0.87 - 1.04 | 0.27 |
| 480 | Pneumonia | respiratory | 192.43 | 0.03 | Random effect model | 1.04 | 0.98 - 1.11 | 0.21 |
| 480.1 | Bacterial pneumonia | respiratory | 198.14 | 0.01 | Random effect model | 1.07 | 0.99 - 1.16 | 0.08 |
| 480.11 | Pneumococcal pneumonia | respiratory | 183.27 | 0.07 | Fixed-effect model | 1.07 | 1.00 - 1.15 | 0.05 |
| 495 | Asthma | respiratory | 280.20 | 5.48E-09 | Random effect model | 0.98 | 0.94 - 1.03 | 0.54 |
| 496 | Chronic airway obstruction | respiratory | 205.42 | 0.01 | Random effect model | 0.92 | 0.87 - 0.98 | 0.01 |
| 496.1 | Emphysema | respiratory | 154.11 | 0.55 | Fixed-effect model | 0.89 | 0.78 - 1.02 | 0.10 |
| 496.2 | Chronic bronchitis | respiratory | 202.78 | 0.01 | Random effect model | 0.91 | 0.81 - 1.02 | 0.12 |
| 496.21 | Obstructive chronic bronchitis | respiratory | 212.44 | 2.13E-03 | Random effect model | 0.92 | 0.81 - 1.04 | 0.17 |
| 497 | Bronchitis | respiratory | 162.12 | 0.37 | Fixed-effect model | 1.05 | 0.85 - 1.31 | 0.65 |
| 501 | Pneumonitis due to inhalation of food or vomitus | respiratory | 156.55 | 0.50 | Fixed-effect model | 1.15 | 0.92 - 1.45 | 0.23 |
| 502 | Postinflammatory pulmonary fibrosis | respiratory | 136.91 | 0.87 | Fixed-effect model | 1.23 | 1.02 - 1.48 | 0.03 |
| 506 | Empyema and pneumothorax | respiratory | 136.72 | 0.88 | Fixed-effect model | 1.20 | 1.02 - 1.41 | 0.02 |
| 507 | Pleurisy; pleural effusion | respiratory | 173.22 | 0.18 | Fixed-effect model | 1.05 | 0.98 - 1.13 | 0.14 |
| 509 | Respiratory failure, insufficiency, arrest | respiratory | 162.87 | 0.36 | Fixed-effect model | 1.00 | 0.89 - 1.11 | 0.95 |
| 509.2 | Respiratory insufficiency | respiratory | 162.15 | 0.37 | Fixed-effect model | 1.05 | 0.92 - 1.20 | 0.48 |
| 510 | Other diseases of lung | respiratory | 189.47 | 0.04 | Random effect model | 0.75 | 0.60 - 0.93 | 0.01 |
| 512 | Other symptoms of respiratory system | respiratory | 138.67 | 0.85 | Fixed-effect model | 1.04 | 0.98 - 1.10 | 0.21 |
| 512.7 | Shortness of breath | respiratory | 121.66 | 0.98 | Fixed-effect model | 1.03 | 0.96 - 1.10 | 0.47 |
| 512.8 | Cough | respiratory | 152.76 | 0.58 | Fixed-effect model | 1.08 | 0.97 - 1.20 | 0.14 |
| 512.9 | Other dyspnea | respiratory | 137.19 | 0.87 | Fixed-effect model | 1.09 | 0.91 - 1.31 | 0.33 |
| 513 | Respiratory abnormalities | respiratory | 158.68 | 0.45 | Fixed-effect model | 0.92 | 0.74 - 1.16 | 0.49 |
| 514 | Abnormal findings examination of lungs | respiratory | 164.67 | 0.32 | Fixed-effect model | 1.07 | 0.96 - 1.20 | 0.21 |
| 516 | Abnormal sputum | respiratory | 177.99 | 0.12 | Fixed-effect model | 0.93 | 0.83 - 1.05 | 0.24 |
| 519 | Other diseases of respiratory system, not elsewhere classified | respiratory | 148.26 | 0.68 | Fixed-effect model | 0.97 | 0.92 - 1.03 | 0.35 |
| 519.8 | Other diseases of respiratory system, NEC | respiratory | 152.47 | 0.59 | Fixed-effect model | 0.99 | 0.94 - 1.05 | 0.83 |
| 520 | Disorders of tooth development | digestive | 141.70 | 0.80 | Fixed-effect model | 1.14 | 1.02 - 1.27 | 0.02 |
| 520.2 | Disturbances in tooth eruption | digestive | 140.19 | 0.83 | Fixed-effect model | 1.13 | 1.01 - 1.26 | 0.04 |
| 521 | Diseases of hard tissues of teeth | digestive | 136.65 | 0.88 | Fixed-effect model | 1.08 | 0.97 - 1.19 | 0.14 |
| 521.1 | Dental caries | digestive | 137.84 | 0.86 | Fixed-effect model | 1.06 | 0.96 - 1.18 | 0.22 |
| 522 | Diseases of pulp and periapical tissues | digestive | 146.94 | 0.71 | Fixed-effect model | 0.93 | 0.82 - 1.06 | 0.30 |
| 522.5 | Periapical abscess | digestive | 154.44 | 0.54 | Fixed-effect model | 1.01 | 0.86 - 1.19 | 0.87 |
| 523 | Gingival and periodontal diseases | digestive | 169.35 | 0.24 | Fixed-effect model | 1.00 | 0.87 - 1.13 | 0.94 |
| 523.3 | Periodontitis (acute or chronic) | digestive | 154.47 | 0.54 | Fixed-effect model | 1.03 | 0.88 - 1.20 | 0.74 |
| 523.31 | Acute periodontitis | digestive | 171.94 | 0.20 | Fixed-effect model | 1.14 | 0.92 - 1.41 | 0.22 |
| 523.32 | Chronic periodontitis | digestive | 141.37 | 0.81 | Fixed-effect model | 0.91 | 0.72 - 1.15 | 0.43 |
| 525 | Other diseases of the teeth and supporting structures | digestive | 174.28 | 0.16 | Fixed-effect model | 0.93 | 0.83 - 1.03 | 0.17 |
| 526 | Diseases of the jaws | digestive | 174.00 | 0.17 | Fixed-effect model | 0.94 | 0.79 - 1.12 | 0.51 |
| 527 | Diseases of the salivary glands | digestive | 151.71 | 0.60 | Fixed-effect model | 1.36 | 1.10 - 1.67 | 3.66E-03 |
| 528 | Diseases of the oral soft tissues, excluding lesions specific for gingiva and tongue | digestive | 159.42 | 0.43 | Fixed-effect model | 0.97 | 0.89 - 1.06 | 0.49 |
| 528.5 | Diseases of lips | digestive | 152.07 | 0.60 | Fixed-effect model | 1.09 | 0.88 - 1.35 | 0.41 |
| 529 | Diseases and other conditions of the tongue | digestive | 174.25 | 0.16 | Fixed-effect model | 0.89 | 0.76 - 1.04 | 0.14 |
| 530 | Diseases of esophagus | digestive | 200.82 | 0.01 | Random effect model | 0.93 | 0.90 - 0.97 | 1.26E-04 |
| 530.1 | Esophagitis, GERD and related diseases | digestive | 179.56 | 0.10 | Fixed-effect model | 0.93 | 0.90 - 0.96 | 1.40E-05 |
| 530.11 | GERD | digestive | 194.49 | 0.02 | Random effect model | 0.95 | 0.91 - 1.01 | 0.09 |
| 530.12 | Ulcer of esophagus | digestive | 196.41 | 0.02 | Random effect model | 0.89 | 0.82 - 0.97 | 0.01 |
| 530.14 | Reflux esophagitis | digestive | 162.27 | 0.37 | Fixed-effect model | 0.91 | 0.86 - 0.96 | 9.58E-04 |
| 530.2 | Esophageal bleeding (varices/hemorrhage) | digestive | 177.35 | 0.13 | Fixed-effect model | 0.94 | 0.82 - 1.07 | 0.34 |
| 530.5 | Disorders of esophageal motility | digestive | 126.36 | 0.97 | Fixed-effect model | 0.82 | 0.66 - 1.02 | 0.07 |
| 530.9 | Heartburn | digestive | 162.51 | 0.37 | Fixed-effect model | 0.81 | 0.72 - 0.91 | 5.37E-04 |
| 531 | Peptic ulcer (excl. esophageal) | digestive | 189.13 | 0.04 | Random effect model | 1.02 | 0.95 - 1.09 | 0.63 |
| 531.1 | Hemorrhage from gastrointestinal ulcer | digestive | 193.33 | 0.03 | Random effect model | 0.86 | 0.67 - 1.09 | 0.21 |
| 531.2 | Gastric ulcer | digestive | 158.03 | 0.46 | Fixed-effect model | 1.04 | 0.95 - 1.14 | 0.36 |
| 531.3 | Duodenal ulcer | digestive | 167.85 | 0.26 | Fixed-effect model | 1.03 | 0.93 - 1.14 | 0.52 |
| 532 | Dysphagia | digestive | 172.08 | 0.19 | Fixed-effect model | 0.97 | 0.91 - 1.04 | 0.47 |
| 535 | Gastritis and duodenitis | digestive | 195.45 | 0.02 | Random effect model | 0.96 | 0.93 - 1.00 | 0.05 |
| 535.1 | Acute gastritis | digestive | 146.73 | 0.71 | Fixed-effect model | 0.94 | 0.80 - 1.10 | 0.45 |
| 535.6 | Duodenitis | digestive | 208.59 | 3.69E-03 | Random effect model | 0.95 | 0.88 - 1.03 | 0.19 |
| 535.8 | Other specified gastritis | digestive | 151.67 | 0.61 | Fixed-effect model | 0.96 | 0.90 - 1.02 | 0.23 |
| 537 | Other disorders of stomach and duodenum | digestive | 171.78 | 0.20 | Fixed-effect model | 0.90 | 0.81 - 0.99 | 0.02 |
| 540 | Appendiceal conditions | digestive | 121.44 | 0.98 | Fixed-effect model | 1.15 | 1.04 - 1.26 | 0.01 |
| 540.1 | Appendicitis | digestive | 118.21 | 0.99 | Fixed-effect model | 1.14 | 1.03 - 1.25 | 0.01 |
| 540.11 | Acute appendicitis | digestive | 131.33 | 0.93 | Fixed-effect model | 1.12 | 1.01 - 1.25 | 0.03 |
| 550 | Abdominal hernia | digestive | 164.33 | 0.33 | Fixed-effect model | 0.96 | 0.94 - 0.99 | 0.01 |
| 550.1 | Inguinal hernia | digestive | 247.02 | 5.95E-06 | Random effect model | 1.10 | 1.03 - 1.16 | 2.26E-03 |
| 550.2 | Diaphragmatic hernia | digestive | 183.66 | 0.07 | Fixed-effect model | 0.90 | 0.87 - 0.93 | 8.77E-09 |
| 550.3 | Femoral hernia | digestive | 164.69 | 0.32 | Fixed-effect model | 1.11 | 0.89 - 1.37 | 0.35 |
| 550.4 | Umbilical hernia | digestive | 138.44 | 0.85 | Fixed-effect model | 0.88 | 0.80 - 0.96 | 0.01 |
| 550.5 | Ventral hernia | digestive | 163.99 | 0.34 | Fixed-effect model | 0.89 | 0.81 - 0.98 | 0.02 |
| 555 | Inflammatory bowel disease and other gastroenteritis and colitis | digestive | 173.65 | 0.17 | Fixed-effect model | 0.93 | 0.85 - 1.01 | 0.08 |
| 555.1 | Regional enteritis | digestive | 113.18 | 1.00 | Fixed-effect model | 0.86 | 0.76 - 0.99 | 0.03 |
| 555.2 | Ulcerative colitis | digestive | 197.58 | 0.02 | Random effect model | 0.96 | 0.86 - 1.07 | 0.43 |
| 555.21 | Ulcerative colitis (chronic) | digestive | 168.49 | 0.25 | Fixed-effect model | 0.99 | 0.78 - 1.25 | 0.91 |
| 556 | Ulceration of the lower GI tract | digestive | 156.59 | 0.49 | Fixed-effect model | 0.99 | 0.83 - 1.17 | 0.89 |
| 556.1 | Ulceration of intestine | digestive | 194.24 | 0.02 | Random effect model | 0.86 | 0.68 - 1.09 | 0.22 |
| 557 | Intestinal malabsorption (non-celiac) | digestive | 246.63 | 6.41E-06 | Random effect model | 1.18 | 1.02 - 1.38 | 0.03 |
| 558 | Noninfectious gastroenteritis | digestive | 172.54 | 0.19 | Fixed-effect model | 0.96 | 0.91 - 1.00 | 0.05 |
| 559 | Ileostomy status | digestive | 149.03 | 0.66 | Fixed-effect model | 1.02 | 0.89 - 1.17 | 0.78 |
| 560 | Intestinal obstruction without mention of hernia | digestive | 161.41 | 0.39 | Fixed-effect model | 0.92 | 0.84 - 1.01 | 0.07 |
| 560.1 | Paralytic ileus | digestive | 157.73 | 0.47 | Fixed-effect model | 0.86 | 0.68 - 1.10 | 0.23 |
| 560.3 | Peritoneal or intestinal adhesions | digestive | 166.49 | 0.29 | Fixed-effect model | 0.81 | 0.67 - 0.98 | 0.03 |
| 560.4 | Other intestinal obstruction | digestive | 162.11 | 0.37 | Fixed-effect model | 0.89 | 0.81 - 0.98 | 0.02 |
| 561 | Symptoms involving digestive system | digestive | 137.78 | 0.86 | Fixed-effect model | 0.98 | 0.94 - 1.02 | 0.32 |
| 561.2 | Flatulence | digestive | 145.18 | 0.74 | Fixed-effect model | 0.88 | 0.77 - 1.01 | 0.06 |
| 562 | Diverticulosis and diverticulitis | digestive | 212.39 | 2.15E-03 | Random effect model | 0.95 | 0.91 - 0.99 | 0.02 |
| 562.1 | Diverticulosis | digestive | 212.20 | 2.21E-03 | Random effect model | 0.95 | 0.91 - 0.99 | 0.02 |
| 563 | Constipation | digestive | 172.69 | 0.19 | Fixed-effect model | 1.00 | 0.95 - 1.06 | 0.90 |
| 564 | Functional digestive disorders | digestive | 197.23 | 0.02 | Random effect model | 0.94 | 0.90 - 0.98 | 0.01 |
| 564.1 | Irritable Bowel Syndrome | digestive | 196.63 | 0.02 | Random effect model | 0.86 | 0.79 - 0.93 | 3.35E-04 |
| 564.8 | Abnormal findings on exam of gastrointestinal tract/ abdominal area | digestive | 166.56 | 0.29 | Fixed-effect model | 1.00 | 0.87 - 1.15 | 0.99 |
| 564.9 | Personal history of diseases of digestive system | digestive | 194.15 | 0.02 | Random effect model | 0.97 | 0.92 - 1.02 | 0.19 |
| 565 | Anal and rectal conditions | digestive | 186.75 | 0.05 | Fixed-effect model | 1.08 | 1.03 - 1.13 | 6.69E-04 |
| 565.1 | Anal and rectal polyp | digestive | 194.82 | 0.02 | Random effect model | 1.02 | 0.95 - 1.10 | 0.57 |
| 567 | Peritonitis and retroperitoneal infections | digestive | 133.41 | 0.91 | Fixed-effect model | 0.83 | 0.69 - 1.00 | 0.05 |
| 568 | Other disorders of peritoneum | digestive | 198.46 | 0.01 | Random effect model | 0.93 | 0.83 - 1.03 | 0.17 |
| 568.1 | Peritoneal adhesions (postoperative) (postinfection) | digestive | 182.15 | 0.08 | Fixed-effect model | 0.93 | 0.84 - 1.03 | 0.15 |
| 569 | Other disorders of intestine | digestive | 141.97 | 0.80 | Fixed-effect model | 1.00 | 0.92 - 1.09 | 0.96 |
| 571 | Chronic liver disease and cirrhosis | digestive | 268.09 | 8.03E-08 | Random effect model | 0.83 | 0.72 - 0.95 | 0.01 |
| 571.5 | Other chronic nonalcoholic liver disease | digestive | 263.12 | 2.32E-07 | Random effect model | 0.86 | 0.72 - 1.02 | 0.09 |
| 571.8 | Liver abscess and sequelae of chronic liver disease | digestive | 170.64 | 0.22 | Fixed-effect model | 0.79 | 0.66 - 0.95 | 0.01 |
| 571.81 | Portal hypertension | digestive | 167.22 | 0.27 | Fixed-effect model | 0.81 | 0.64 - 1.03 | 0.08 |
| 572 | Ascites (non malignant) | digestive | 187.79 | 0.05 | Random effect model | 0.80 | 0.69 - 0.93 | 4.59E-03 |
| 573 | Other disorders of liver | digestive | 199.49 | 0.01 | Random effect model | 1.02 | 0.94 - 1.10 | 0.72 |
| 573.5 | Jaundice (not of newborn) | digestive | 162.98 | 0.36 | Fixed-effect model | 0.69 | 0.57 - 0.83 | 7.67E-05 |
| 573.7 | Abnormal results of function study of liver | digestive | 181.88 | 0.08 | Fixed-effect model | 0.99 | 0.90 - 1.09 | 0.84 |
| 574 | Cholelithiasis and cholecystitis | digestive | 785.00 | 3.54E-84 | Random effect model | 0.79 | 0.72 - 0.88 | 7.12E-06 |
| 574.1 | Cholelithiasis | digestive | 776.11 | 1.26E-82 | Random effect model | 0.79 | 0.71 - 0.88 | 1.87E-05 |
| 574.11 | Cholelithiasis with acute cholecystitis | digestive | 244.24 | 1.01E-05 | Random effect model | 0.80 | 0.67 - 0.96 | 0.02 |
| 574.12 | Cholelithiasis with other cholecystitis | digestive | 475.05 | 1.06E-33 | Random effect model | 0.83 | 0.73 - 0.95 | 0.01 |
| 574.2 | Calculus of bile duct | digestive | 266.24 | 1.19E-07 | Random effect model | 0.74 | 0.65 - 0.86 | 3.86E-05 |
| 574.3 | Cholecystitis without cholelithiasis | digestive | 214.65 | 1.54E-03 | Random effect model | 0.70 | 0.62 - 0.79 | 1.71E-08 |
| 575 | Other biliary tract disease | digestive | 237.39 | 3.59E-05 | Random effect model | 0.78 | 0.70 - 0.87 | 6.44E-06 |
| 575.2 | Obstruction of bile duct | digestive | 165.12 | 0.31 | Fixed-effect model | 0.75 | 0.62 - 0.92 | 0.01 |
| 575.7 | Other disorders of gallbladder | digestive | 201.28 | 0.01 | Random effect model | 0.87 | 0.74 - 1.03 | 0.11 |
| 575.8 | Other disorders of biliary tract | digestive | 167.25 | 0.27 | Fixed-effect model | 0.70 | 0.59 - 0.83 | 3.31E-05 |
| 577 | Diseases of pancreas | digestive | 191.19 | 0.03 | Random effect model | 0.85 | 0.75 - 0.95 | 0.01 |
| 577.2 | Chronic pancreatitis | digestive | 129.10 | 0.95 | Fixed-effect model | 0.94 | 0.73 - 1.20 | 0.60 |
| 578 | Gastrointestinal hemorrhage | digestive | 171.57 | 0.20 | Fixed-effect model | 1.00 | 0.96 - 1.03 | 0.80 |
| 578.2 | Blood in stool | digestive | 152.65 | 0.58 | Fixed-effect model | 1.00 | 0.90 - 1.12 | 0.97 |
| 578.8 | Hemorrhage of rectum and anus | digestive | 155.79 | 0.51 | Fixed-effect model | 0.99 | 0.94 - 1.03 | 0.56 |
| 578.9 | Hemorrhage of gastrointestinal tract | digestive | 152.91 | 0.58 | Fixed-effect model | 0.96 | 0.89 - 1.04 | 0.33 |
| 579 | Other symptoms involving abdomen and pelvis | digestive | 166.85 | 0.28 | Fixed-effect model | 0.95 | 0.87 - 1.05 | 0.34 |
| 579.8 | Nonspecific abnormal findings in stool contents | digestive | 187.50 | 0.05 | Random effect model | 0.88 | 0.76 - 1.02 | 0.09 |
| 580 | Nephritis; nephrosis; renal sclerosis | genitourinary | 165.53 | 0.30 | Fixed-effect model | 0.95 | 0.83 - 1.10 | 0.51 |
| 580.1 | Glomerulonephritis | genitourinary | 149.95 | 0.64 | Fixed-effect model | 0.89 | 0.75 - 1.06 | 0.19 |
| 580.14 | Chronic glomerulonephritis, NOS | genitourinary | 145.99 | 0.73 | Fixed-effect model | 0.94 | 0.78 - 1.13 | 0.50 |
| 585 | Renal failure | genitourinary | 171.39 | 0.20 | Fixed-effect model | 0.94 | 0.88 - 1.01 | 0.08 |
| 585.1 | Acute renal failure | genitourinary | 159.20 | 0.44 | Fixed-effect model | 0.94 | 0.87 - 1.02 | 0.16 |
| 585.2 | Renal failure NOS | genitourinary | 155.26 | 0.52 | Fixed-effect model | 1.02 | 0.88 - 1.18 | 0.77 |
| 585.3 | Chronic renal failure [CKD] | genitourinary | 164.55 | 0.32 | Fixed-effect model | 0.92 | 0.83 - 1.03 | 0.14 |
| 586 | Other disorders of the kidney and ureters | genitourinary | 143.12 | 0.78 | Fixed-effect model | 1.01 | 0.92 - 1.11 | 0.88 |
| 586.2 | Cyst of kidney, acquired | genitourinary | 162.81 | 0.36 | Fixed-effect model | 0.97 | 0.83 - 1.14 | 0.74 |
| 586.4 | Stricture/obstruction of ureter | genitourinary | 132.64 | 0.92 | Fixed-effect model | 1.03 | 0.86 - 1.24 | 0.71 |
| 590 | Pyelonephritis | genitourinary | 214.53 | 1.57E-03 | Random effect model | 0.93 | 0.78 - 1.11 | 0.39 |
| 591 | Urinary tract infection | genitourinary | 172.38 | 0.19 | Fixed-effect model | 0.93 | 0.89 - 0.98 | 0.01 |
| 592 | Cystitis and urethritis | genitourinary | 137.07 | 0.87 | Fixed-effect model | 0.93 | 0.84 - 1.02 | 0.13 |
| 592.1 | Cystitis | genitourinary | 134.37 | 0.90 | Fixed-effect model | 0.92 | 0.83 - 1.02 | 0.12 |
| 592.12 | Chronic cystitis | genitourinary | 154.07 | 0.55 | Fixed-effect model | 1.00 | 0.83 - 1.20 | 0.97 |
| 593 | Hematuria | genitourinary | 207.57 | 4.25E-03 | Random effect model | 1.01 | 0.96 - 1.07 | 0.61 |
| 594 | Urinary calculus | genitourinary | 191.19 | 0.03 | Random effect model | 0.91 | 0.84 - 0.98 | 0.01 |
| 594.1 | Calculus of kidney | genitourinary | 176.12 | 0.14 | Fixed-effect model | 0.87 | 0.79 - 0.96 | 4.81E-03 |
| 594.2 | Calculus of lower urinary tract | genitourinary | 107.45 | 1.00 | Fixed-effect model | 0.84 | 0.69 - 1.02 | 0.08 |
| 594.3 | Calculus of ureter | genitourinary | 189.67 | 0.04 | Random effect model | 1.05 | 0.93 - 1.19 | 0.41 |
| 596 | Other disorders of bladder | genitourinary | 155.94 | 0.51 | Fixed-effect model | 0.94 | 0.89 - 1.00 | 0.05 |
| 596.5 | Functional disorders of bladder | genitourinary | 162.83 | 0.36 | Fixed-effect model | 0.76 | 0.66 - 0.87 | 6.21E-05 |
| 597 | Other disorders of urethra and urinary tract | genitourinary | 174.01 | 0.17 | Fixed-effect model | 1.00 | 0.92 - 1.09 | 0.95 |
| 597.1 | Urethral stricture (not specified as infectious) | genitourinary | 156.83 | 0.49 | Fixed-effect model | 0.97 | 0.88 - 1.07 | 0.58 |
| 598 | Abnormal findings on examination of urine | genitourinary | 157.54 | 0.47 | Fixed-effect model | 0.83 | 0.76 - 0.91 | 7.24E-05 |
| 599 | Other symptoms/disorders or the urinary system | genitourinary | 109.90 | 1.00 | Fixed-effect model | 0.95 | 0.91 - 0.98 | 3.74E-03 |
| 599.2 | Retention of urine | genitourinary | 135.50 | 0.89 | Fixed-effect model | 1.04 | 0.97 - 1.12 | 0.22 |
| 599.3 | Dysuria | genitourinary | 161.84 | 0.38 | Fixed-effect model | 0.81 | 0.70 - 0.95 | 0.01 |
| 599.4 | Urinary incontinence | genitourinary | 165.88 | 0.30 | Fixed-effect model | 0.86 | 0.81 - 0.91 | 3.32E-07 |
| 599.5 | Frequency of urination and polyuria | genitourinary | 123.52 | 0.98 | Fixed-effect model | 1.00 | 0.92 - 1.09 | 0.97 |
| 611 | Abnormal findings on mammogram or breast exam | genitourinary | 197.81 | 0.02 | Random effect model | 1.32 | 1.13 - 1.55 | 4.12E-04 |
| 611.3 | Lump or mass in breast | genitourinary | 187.84 | 0.05 | Random effect model | 1.32 | 1.13 - 1.54 | 4.73E-04 |
| 612 | Breast conditions, congenital or relating to hormones | genitourinary | 143.94 | 0.76 | Fixed-effect model | 0.85 | 0.71 - 1.02 | 0.09 |
| 612.2 | Hypertrophy of breast (Gynecomastia) | genitourinary | 151.92 | 0.60 | Fixed-effect model | 0.85 | 0.71 - 1.03 | 0.10 |
| 613.1 | Inflammatory disease of breast | genitourinary | 144.75 | 0.75 | Fixed-effect model | 0.86 | 0.70 - 1.06 | 0.15 |
| 613.7 | Other signs and symptoms in breast | genitourinary | 151.04 | 0.62 | Fixed-effect model | 0.95 | 0.77 - 1.17 | 0.65 |
| 619.1 | Noninflammatory disorders of ovary, fallopian tube, and broad ligament | genitourinary | 131.88 | 0.93 | Fixed-effect model | 1.04 | 0.85 - 1.28 | 0.67 |
| 681 | Superficial cellulitis and abscess | dermatologic | 179.25 | 0.11 | Fixed-effect model | 0.95 | 0.89 - 1.01 | 0.09 |
| 681.1 | Cellulitis and abscess of fingers/toes | dermatologic | 146.58 | 0.71 | Fixed-effect model | 1.06 | 0.84 - 1.34 | 0.61 |
| 681.2 | Cellulitis and abscess of face/neck | dermatologic | 148.99 | 0.66 | Fixed-effect model | 1.19 | 0.94 - 1.51 | 0.15 |
| 681.3 | Cellulitis and abscess of arm/hand | dermatologic | 156.12 | 0.50 | Fixed-effect model | 0.91 | 0.85 - 0.98 | 0.01 |
| 681.5 | Cellulitis and abscess of leg, except foot | dermatologic | 152.41 | 0.59 | Fixed-effect model | 0.90 | 0.84 - 0.97 | 0.01 |
| 681.6 | Cellulitis and abscess of foot, toe | dermatologic | 155.03 | 0.53 | Fixed-effect model | 0.91 | 0.85 - 0.98 | 0.02 |
| 681.7 | Cellulitis and abscess of trunk | dermatologic | 161.35 | 0.39 | Fixed-effect model | 1.05 | 0.84 - 1.32 | 0.67 |
| 686 | Other local infections of skin and subcutaneous tissue | dermatologic | 172.25 | 0.19 | Fixed-effect model | 0.90 | 0.83 - 0.97 | 0.01 |
| 686.1 | Carbuncle and furuncle | dermatologic | 178.45 | 0.12 | Fixed-effect model | 0.83 | 0.74 - 0.94 | 2.06E-03 |
| 686.3 | Pilonidal cyst | dermatologic | 160.22 | 0.41 | Fixed-effect model | 1.04 | 0.82 - 1.30 | 0.77 |
| 687 | Symptoms affecting skin | dermatologic | 204.39 | 0.01 | Random effect model | 1.06 | 0.98 - 1.16 | 0.16 |
| 687.1 | Rash and other nonspecific skin eruption | dermatologic | 168.68 | 0.25 | Fixed-effect model | 1.10 | 0.98 - 1.24 | 0.11 |
| 687.4 | Disturbance of skin sensation | dermatologic | 160.75 | 0.40 | Fixed-effect model | 1.04 | 0.94 - 1.15 | 0.49 |
| 689 | Disorder of skin and subcutaneous tissue NOS | dermatologic | 126.23 | 0.97 | Fixed-effect model | 0.97 | 0.90 - 1.04 | 0.38 |
| 694 | Dyschromia and Vitiligo | dermatologic | 166.98 | 0.28 | Fixed-effect model | 0.97 | 0.81 - 1.16 | 0.74 |
| 694.2 | Other dyschromia | dermatologic | 159.96 | 0.42 | Fixed-effect model | 1.05 | 0.87 - 1.28 | 0.59 |
| 695 | Erythematous conditions | dermatologic | 174.89 | 0.16 | Fixed-effect model | 1.00 | 0.90 - 1.12 | 0.95 |
| 695.7 | Prurigo and Lichen | dermatologic | 121.30 | 0.98 | Fixed-effect model | 1.05 | 0.86 - 1.27 | 0.65 |
| 696 | Psoriasis and related disorders | dermatologic | 182.00 | 0.08 | Fixed-effect model | 0.93 | 0.83 - 1.05 | 0.24 |
| 696.4 | Psoriasis | dermatologic | 180.95 | 0.09 | Fixed-effect model | 0.94 | 0.84 - 1.06 | 0.32 |
| 696.41 | Psoriasis vulgaris | dermatologic | 179.95 | 0.10 | Fixed-effect model | 0.90 | 0.79 - 1.03 | 0.13 |
| 696.42 | Psoriatic arthropathy | dermatologic | 175.65 | 0.15 | Fixed-effect model | 1.13 | 0.92 - 1.39 | 0.24 |
| 697 | Sarcoidosis | dermatologic | 176.46 | 0.14 | Fixed-effect model | 0.84 | 0.67 - 1.07 | 0.16 |
| 698 | Pruritus and related conditions | dermatologic | 157.30 | 0.48 | Fixed-effect model | 0.99 | 0.81 - 1.20 | 0.92 |
| 701 | Other hypertrophic and atrophic conditions of skin | dermatologic | 160.74 | 0.40 | Fixed-effect model | 1.10 | 1.01 - 1.19 | 0.02 |
| 701.2 | Scar conditions and fibrosis of skin | dermatologic | 143.22 | 0.78 | Fixed-effect model | 1.35 | 1.20 - 1.51 | 2.09E-07 |
| 702 | Degenerative skin conditions and other dermatoses | dermatologic | 177.12 | 0.13 | Fixed-effect model | 1.11 | 1.03 - 1.19 | 0.01 |
| 702.1 | Actinic keratosis | dermatologic | 169.71 | 0.23 | Fixed-effect model | 1.07 | 0.96 - 1.19 | 0.21 |
| 702.2 | Seborrheic keratosis | dermatologic | 196.22 | 0.02 | Random effect model | 1.18 | 1.06 - 1.32 | 3.24E-03 |
| 703 | Diseases of nail, NOS | dermatologic | 145.56 | 0.73 | Fixed-effect model | 1.03 | 0.89 - 1.21 | 0.67 |
| 703.1 | Ingrowing nail | dermatologic | 154.37 | 0.54 | Fixed-effect model | 0.99 | 0.83 - 1.19 | 0.95 |
| 704 | Diseases of hair and hair follicles | dermatologic | 167.15 | 0.27 | Fixed-effect model | 1.04 | 0.96 - 1.12 | 0.35 |
| 705 | Disorders of sweat glands | dermatologic | 184.90 | 0.06 | Fixed-effect model | 0.88 | 0.72 - 1.06 | 0.18 |
| 705.8 | Hyperhidrosis | dermatologic | 174.14 | 0.17 | Fixed-effect model | 0.96 | 0.76 - 1.20 | 0.70 |
| 706 | Diseases of sebaceous glands | dermatologic | 207.03 | 4.58E-03 | Random effect model | 1.03 | 0.96 - 1.10 | 0.36 |
| 706.2 | Sebaceous cyst | dermatologic | 206.09 | 0.01 | Random effect model | 1.03 | 0.97 - 1.11 | 0.33 |
| 707.1 | Decubitus ulcer | dermatologic | 149.33 | 0.66 | Fixed-effect model | 1.08 | 0.90 - 1.29 | 0.41 |
| 709 | Diffuse diseases of connective tissue | dermatologic | 155.79 | 0.51 | Fixed-effect model | 0.92 | 0.84 - 1.01 | 0.08 |
| 709.2 | Sicca syndrome | dermatologic | 170.48 | 0.22 | Fixed-effect model | 1.01 | 0.79 - 1.29 | 0.92 |
| 709.7 | Unspecified diffuse connective tissue disease | dermatologic | 139.40 | 0.84 | Fixed-effect model | 0.91 | 0.82 - 1.01 | 0.07 |
| 710 | Osteomyelitis, periostitis, and other infections involving bone | musculoskeletal | 156.72 | 0.49 | Fixed-effect model | 0.98 | 0.79 - 1.23 | 0.89 |
| 710.1 | Osteomyelitis | musculoskeletal | 160.66 | 0.40 | Fixed-effect model | 1.01 | 0.80 - 1.27 | 0.96 |
| 714 | Rheumatoid arthritis and other inflammatory polyarthropathies | musculoskeletal | 180.26 | 0.10 | Fixed-effect model | 1.01 | 0.93 - 1.09 | 0.81 |
| 714.1 | Rheumatoid arthritis | musculoskeletal | 172.30 | 0.19 | Fixed-effect model | 1.05 | 0.96 - 1.14 | 0.26 |
| 715 | Other inflammatory spondylopathies | musculoskeletal | 157.72 | 0.47 | Fixed-effect model | 1.07 | 0.93 - 1.22 | 0.36 |
| 715.2 | Ankylosing spondylitis | musculoskeletal | 219.99 | 6.79E-04 | Random effect model | 1.12 | 0.86 - 1.45 | 0.40 |
| 716 | Other arthropathies | musculoskeletal | 303.08 | 2.38E-11 | Random effect model | 0.93 | 0.89 - 0.97 | 1.07E-03 |
| 716.1 | Unspecified polyarthropathy or polyarthritis | musculoskeletal | 196.27 | 0.02 | Random effect model | 1.05 | 0.94 - 1.16 | 0.38 |
| 716.2 | Unspecified monoarthritis | musculoskeletal | 229.03 | 1.55E-04 | Random effect model | 0.90 | 0.85 - 0.95 | 1.69E-04 |
| 716.9 | Arthropathy NOS | musculoskeletal | 303.22 | 2.29E-11 | Random effect model | 0.92 | 0.88 - 0.96 | 3.75E-04 |
| 717 | Polymyalgia Rheumatica | musculoskeletal | 152.93 | 0.58 | Fixed-effect model | 0.91 | 0.77 - 1.07 | 0.25 |
| 720 | Spinal stenosis | musculoskeletal | 190.36 | 0.04 | Random effect model | 0.85 | 0.77 - 0.94 | 2.00E-03 |
| 721 | Spondylosis and allied disorders | musculoskeletal | 174.74 | 0.16 | Fixed-effect model | 0.93 | 0.87 - 0.99 | 0.03 |
| 721.1 | Spondylosis without myelopathy | musculoskeletal | 180.00 | 0.10 | Fixed-effect model | 0.90 | 0.84 - 0.98 | 0.01 |
| 722 | Intervertebral disc disorders | musculoskeletal | 167.20 | 0.27 | Fixed-effect model | 0.89 | 0.84 - 0.95 | 1.67E-04 |
| 722.1 | Displacement of intervertebral disc | musculoskeletal | 157.72 | 0.47 | Fixed-effect model | 0.94 | 0.73 - 1.20 | 0.61 |
| 722.6 | Degeneration of intervertebral disc | musculoskeletal | 225.30 | 2.90E-04 | Random effect model | 0.89 | 0.79 - 1.01 | 0.07 |
| 722.9 | Other and unspecified disc disorder | musculoskeletal | 173.33 | 0.18 | Fixed-effect model | 0.82 | 0.75 - 0.89 | 2.17E-06 |
| 724.9 | Other unspecified back disorders | musculoskeletal | 134.69 | 0.90 | Fixed-effect model | 0.89 | 0.77 - 1.02 | 0.09 |
| 726 | Peripheral enthesopathies and allied syndromes | musculoskeletal | 234.85 | 5.66E-05 | Random effect model | 0.92 | 0.87 - 0.98 | 4.79E-03 |
| 726.1 | Enthesopathy | musculoskeletal | 197.19 | 0.02 | Random effect model | 0.93 | 0.88 - 1.00 | 0.04 |
| 726.3 | Bursitis | musculoskeletal | 144.91 | 0.75 | Fixed-effect model | 0.80 | 0.66 - 0.96 | 0.02 |
| 727 | Other disorders of synovium, tendon, and bursa | musculoskeletal | 164.97 | 0.32 | Fixed-effect model | 0.93 | 0.87 - 0.99 | 0.03 |
| 727.1 | Synovitis and tenosynovitis | musculoskeletal | 141.14 | 0.81 | Fixed-effect model | 0.92 | 0.83 - 1.02 | 0.11 |
| 727.4 | Ganglion and cyst of synovium, tendon, and bursa | musculoskeletal | 180.52 | 0.10 | Fixed-effect model | 1.00 | 0.91 - 1.11 | 0.95 |
| 727.5 | Rupture of synovium | musculoskeletal | 148.70 | 0.67 | Fixed-effect model | 0.80 | 0.64 - 1.00 | 0.05 |
| 728 | Disorders of muscle, ligament, and fascia | musculoskeletal | 175.13 | 0.15 | Fixed-effect model | 0.98 | 0.90 - 1.07 | 0.63 |
| 728.7 | Fasciitis | musculoskeletal | 173.78 | 0.17 | Fixed-effect model | 1.02 | 0.93 - 1.12 | 0.64 |
| 728.71 | Contracture of palmar fascia [Dupuytren's disease] | musculoskeletal | 195.46 | 0.02 | Random effect model | 1.07 | 0.96 - 1.19 | 0.22 |
| 729 | Other disorders of soft tissues | musculoskeletal | 180.86 | 0.09 | Fixed-effect model | 0.89 | 0.83 - 0.96 | 1.86E-03 |
| 729.1 | Rheumatism, unspecified and fibrositis | musculoskeletal | 184.63 | 0.07 | Fixed-effect model | 0.99 | 0.83 - 1.18 | 0.90 |
| 733 | Other disorders of bone and cartilage | musculoskeletal | 137.30 | 0.87 | Fixed-effect model | 0.92 | 0.84 - 1.00 | 0.05 |
| 733.4 | Aseptic necrosis of bone | musculoskeletal | 149.75 | 0.65 | Fixed-effect model | 0.95 | 0.75 - 1.21 | 0.69 |
| 733.8 | Malunion and nonunion of fracture | musculoskeletal | 137.02 | 0.87 | Fixed-effect model | 1.01 | 0.87 - 1.18 | 0.86 |
| 735 | Acquired foot deformities | musculoskeletal | 177.99 | 0.12 | Fixed-effect model | 0.90 | 0.85 - 0.95 | 1.79E-04 |
| 735.2 | Acquired toe deformities | musculoskeletal | 201.55 | 0.01 | Random effect model | 0.93 | 0.85 - 1.02 | 0.11 |
| 735.23 | Hallux rigidus | musculoskeletal | 172.07 | 0.19 | Fixed-effect model | 0.95 | 0.83 - 1.10 | 0.49 |
| 735.3 | Hallux valgus (Bunion) | musculoskeletal | 164.78 | 0.32 | Fixed-effect model | 0.87 | 0.81 - 0.93 | 5.51E-05 |
| 736 | Other acquired deformities of limbs | musculoskeletal | 229.37 | 1.46E-04 | Random effect model | 0.84 | 0.71 - 0.99 | 0.04 |
| 737 | Curvature of spine | musculoskeletal | 163.80 | 0.34 | Fixed-effect model | 0.87 | 0.74 - 1.03 | 0.10 |
| 737.3 | Kyphoscoliosis and scoliosis | musculoskeletal | 156.63 | 0.49 | Fixed-effect model | 0.87 | 0.74 - 1.03 | 0.12 |
| 738.4 | Acquired spondylolisthesis | musculoskeletal | 155.09 | 0.53 | Fixed-effect model | 1.01 | 0.87 - 1.16 | 0.93 |
| 740 | Osteoarthrosis | musculoskeletal | 283.96 | 2.31E-09 | Random effect model | 0.91 | 0.87 - 0.95 | 8.55E-05 |
| 740.1 | Osteoarthritis; localized | musculoskeletal | 262.13 | 2.85E-07 | Random effect model | 0.89 | 0.84 - 0.94 | 2.54E-05 |
| 740.11 | Osteoarthrosis, localized, primary | musculoskeletal | 203.10 | 0.01 | Random effect model | 0.89 | 0.83 - 0.95 | 6.85E-04 |
| 740.2 | Osteoarthrosis, generalized | musculoskeletal | 146.40 | 0.72 | Fixed-effect model | 0.90 | 0.71 - 1.12 | 0.34 |
| 740.9 | Osteoarthrosis NOS | musculoskeletal | 243.07 | 1.26E-05 | Random effect model | 0.96 | 0.90 - 1.03 | 0.23 |
| 741 | Symptoms and disorders of the joints | musculoskeletal | 135.42 | 0.89 | Fixed-effect model | 0.98 | 0.89 - 1.07 | 0.61 |
| 741.2 | Stiffness of joint | musculoskeletal | 140.91 | 0.82 | Fixed-effect model | 0.88 | 0.70 - 1.09 | 0.24 |
| 741.4 | Joint effusions | musculoskeletal | 140.79 | 0.82 | Fixed-effect model | 0.90 | 0.78 - 1.04 | 0.16 |
| 742 | Derangement of joint, nontraumatic | musculoskeletal | 147.95 | 0.69 | Fixed-effect model | 0.82 | 0.74 - 0.91 | 2.18E-04 |
| 742.8 | Articular cartilage disorder | musculoskeletal | 154.84 | 0.53 | Fixed-effect model | 0.86 | 0.67 - 1.10 | 0.23 |
| 743 | Osteoporosis, osteopenia and pathological fracture | musculoskeletal | 198.79 | 0.01 | Random effect model | 1.01 | 0.94 - 1.09 | 0.69 |
| 743.1 | Osteoporosis | musculoskeletal | 216.49 | 1.17E-03 | Random effect model | 0.98 | 0.91 - 1.07 | 0.71 |
| 743.11 | Osteoporosis NOS | musculoskeletal | 224.64 | 3.22E-04 | Random effect model | 0.98 | 0.90 - 1.07 | 0.67 |
| 743.13 | Other specified osteoporosis | musculoskeletal | 147.01 | 0.70 | Fixed-effect model | 0.84 | 0.67 - 1.07 | 0.16 |
| 743.2 | Pathologic fracture | musculoskeletal | 197.47 | 0.02 | Random effect model | 1.47 | 1.12 - 1.94 | 0.01 |
| 743.9 | Osteopenia or other disorder of bone and cartilage | musculoskeletal | 161.30 | 0.39 | Fixed-effect model | 1.07 | 0.89 - 1.30 | 0.47 |
| 745 | Pain in joint | musculoskeletal | 197.28 | 0.02 | Random effect model | 0.94 | 0.87 - 1.00 | 0.06 |
| 747 | Cardiac and circulatory congenital anomalies | congenital anomalies | 196.53 | 0.02 | Random effect model | 1.11 | 0.99 - 1.25 | 0.08 |
| 747.1 | Cardiac congenital anomalies | congenital anomalies | 221.01 | 5.78E-04 | Random effect model | 1.13 | 0.99 - 1.28 | 0.07 |
| 747.11 | Cardiac shunt/ heart septal defect | congenital anomalies | 164.44 | 0.33 | Fixed-effect model | 0.76 | 0.61 - 0.96 | 0.02 |
| 747.13 | Congenital anomalies of great vessels | congenital anomalies | 214.25 | 1.63E-03 | Random effect model | 1.35 | 1.16 - 1.57 | 1.08E-04 |
| 750 | Digestive congenital anomalies | congenital anomalies | 154.91 | 0.53 | Fixed-effect model | 0.87 | 0.71 - 1.07 | 0.18 |
| 751 | Genitourinary congenital anomalies | congenital anomalies | 163.00 | 0.36 | Fixed-effect model | 0.77 | 0.67 - 0.88 | 1.42E-04 |
| 751.1 | Congenital anomalies of genital organs | congenital anomalies | 176.37 | 0.14 | Fixed-effect model | 0.85 | 0.69 - 1.04 | 0.11 |
| 751.2 | Congenital anomalies of urinary system | congenital anomalies | 134.47 | 0.90 | Fixed-effect model | 0.72 | 0.60 - 0.87 | 6.32E-04 |
| 756 | Other congenital musculoskeletal anomalies | congenital anomalies | 180.03 | 0.10 | Fixed-effect model | 0.77 | 0.61 - 0.97 | 0.02 |
| 760 | Back pain | symptoms | 179.53 | 0.11 | Fixed-effect model | 0.96 | 0.91 - 1.01 | 0.12 |
| 761 | Cervicalgia | symptoms | 149.08 | 0.66 | Fixed-effect model | 1.03 | 0.90 - 1.17 | 0.65 |
| 764 | Sciatica | symptoms | 132.37 | 0.92 | Fixed-effect model | 0.93 | 0.83 - 1.04 | 0.19 |
| 766 | Neuralgia, neuritis, and radiculitis NOS | symptoms | 157.42 | 0.48 | Fixed-effect model | 0.86 | 0.73 - 1.01 | 0.07 |
| 770 | Myalgia and myositis unspecified | symptoms | 193.22 | 0.03 | Random effect model | 1.21 | 0.95 - 1.53 | 0.13 |
| 771 | Musculoskeletal symptoms referable to limbs | symptoms | 188.83 | 0.04 | Random effect model | 0.85 | 0.79 - 0.93 | 1.51E-04 |
| 771.1 | Swelling of limb | symptoms | 190.96 | 0.03 | Random effect model | 0.85 | 0.78 - 0.93 | 4.05E-04 |
| 772 | Symptoms of the muscles | symptoms | 139.03 | 0.85 | Fixed-effect model | 1.22 | 0.99 - 1.50 | 0.06 |
| 773 | Pain in limb | symptoms | 197.99 | 0.01 | Random effect model | 0.99 | 0.91 - 1.07 | 0.79 |
| 782 | Symptoms involving skin and other integumentary tissue | symptoms | 162.87 | 0.36 | Fixed-effect model | 0.95 | 0.83 - 1.08 | 0.44 |
| 782.3 | Edema | symptoms | 162.63 | 0.36 | Fixed-effect model | 0.93 | 0.81 - 1.06 | 0.28 |
| 783 | Fever of unknown origin | symptoms | 132.53 | 0.92 | Fixed-effect model | 1.03 | 0.95 - 1.13 | 0.47 |
| 785 | Abdominal pain | symptoms | 120.71 | 0.99 | Fixed-effect model | 0.93 | 0.91 - 0.96 | 2.55E-06 |
| 788 | Syncope and collapse | symptoms | 166.30 | 0.29 | Fixed-effect model | 1.06 | 1.00 - 1.12 | 0.07 |
| 789 | Nausea and vomiting | symptoms | 158.58 | 0.45 | Fixed-effect model | 0.91 | 0.87 - 0.96 | 5.80E-04 |
| 790 | Nonspecific findings on examination of blood | symptoms | 202.37 | 0.01 | Random effect model | 1.01 | 0.93 - 1.09 | 0.84 |
| 790.6 | Other abnormal blood chemistry | symptoms | 211.97 | 2.28E-03 | Random effect model | 1.00 | 0.92 - 1.08 | 0.98 |
| 791 | Gangrene | symptoms | 150.25 | 0.64 | Fixed-effect model | 0.87 | 0.69 - 1.10 | 0.25 |
| 793 | Nonspecific abnormal findings on radiological and other examination of musculoskeletal system | injuries & poisonings | 135.05 | 0.90 | Fixed-effect model | 1.24 | 1.00 - 1.52 | 0.04 |
| 793.2 | Nonspecific abnormal findings on radiological and other examination of other intrathoracic organs (echocardiogram, etc) | circulatory system | 131.99 | 0.93 | Fixed-effect model | 1.27 | 1.01 - 1.60 | 0.04 |
| 798 | Malaise and fatigue | symptoms | 126.04 | 0.97 | Fixed-effect model | 1.03 | 0.93 - 1.13 | 0.59 |
| 798.1 | Chronic fatigue syndrome | symptoms | 139.52 | 0.84 | Fixed-effect model | 0.85 | 0.68 - 1.07 | 0.17 |
| 800 | Fracture of lower limb | injuries & poisonings | 190.52 | 0.04 | Random effect model | 0.96 | 0.90 - 1.04 | 0.31 |
| 800.1 | Fracture of neck of femur | injuries & poisonings | 173.44 | 0.18 | Fixed-effect model | 0.83 | 0.73 - 0.95 | 0.01 |
| 800.3 | Fracture of tibia and fibula | injuries & poisonings | 136.42 | 0.88 | Fixed-effect model | 1.11 | 0.98 - 1.25 | 0.09 |
| 800.4 | Fracture of patella | injuries & poisonings | 152.03 | 0.60 | Fixed-effect model | 1.13 | 0.93 - 1.38 | 0.22 |
| 801 | Fracture of ankle and foot | injuries & poisonings | 145.81 | 0.73 | Fixed-effect model | 0.98 | 0.87 - 1.10 | 0.69 |
| 802 | Fracture of pelvis | injuries & poisonings | 200.32 | 0.01 | Random effect model | 1.14 | 0.91 - 1.44 | 0.26 |
| 803 | Fracture of upper limb | injuries & poisonings | 186.21 | 0.06 | Fixed-effect model | 1.04 | 0.98 - 1.11 | 0.20 |
| 803.1 | Fracture of humerus | injuries & poisonings | 163.43 | 0.35 | Fixed-effect model | 0.98 | 0.86 - 1.12 | 0.78 |
| 803.2 | Fracture of radius and ulna | injuries & poisonings | 201.81 | 0.01 | Random effect model | 1.06 | 0.97 - 1.16 | 0.17 |
| 803.3 | Fracture of clavicle or scapula | injuries & poisonings | 162.18 | 0.37 | Fixed-effect model | 1.03 | 0.89 - 1.18 | 0.71 |
| 804 | Fracture of hand or wrist | injuries & poisonings | 184.90 | 0.06 | Fixed-effect model | 1.12 | 1.02 - 1.23 | 0.02 |
| 805 | Fracture of vertebral column without mention of spinal cord injury | injuries & poisonings | 139.03 | 0.85 | Fixed-effect model | 0.86 | 0.74 - 1.00 | 0.05 |
| 807 | Fracture of ribs | injuries & poisonings | 132.55 | 0.92 | Fixed-effect model | 0.76 | 0.63 - 0.93 | 0.01 |
| 809 | Fracture of unspecified bones | injuries & poisonings | 121.87 | 0.98 | Fixed-effect model | 0.92 | 0.79 - 1.07 | 0.27 |
| 818 | Intracranial hemorrhage (injury) | injuries & poisonings | 125.14 | 0.97 | Fixed-effect model | 0.96 | 0.75 - 1.23 | 0.76 |
| 819 | Skull and face fracture and other intercranial injury | injuries & poisonings | 173.23 | 0.18 | Fixed-effect model | 1.03 | 0.93 - 1.14 | 0.56 |
| 823 | Torus fracture | injuries & poisonings | 150.13 | 0.64 | Fixed-effect model | 1.13 | 0.99 - 1.30 | 0.07 |
| 835 | Internal derangement of knee | injuries & poisonings | 160.48 | 0.41 | Fixed-effect model | 0.98 | 0.94 - 1.03 | 0.43 |
| 836 | Traumatic arthropathy | injuries & poisonings | 139.14 | 0.84 | Fixed-effect model | 1.08 | 0.85 - 1.37 | 0.54 |
| 850 | Hemorrhage or hematoma complicating a procedure | injuries & poisonings | 181.35 | 0.09 | Fixed-effect model | 1.06 | 0.98 - 1.15 | 0.12 |
| 853 | Complication of colostomy or enterostomy | injuries & poisonings | 174.36 | 0.16 | Fixed-effect model | 0.76 | 0.60 - 0.98 | 0.03 |
| 854 | Complications of cardiac/vascular device, implant, and graft | injuries & poisonings | 235.57 | 4.97E-05 | Random effect model | 1.16 | 0.99 - 1.36 | 0.07 |
| 857 | Mechanical complication of unspecified genitourinary device, implant, and graft | injuries & poisonings | 169.24 | 0.24 | Fixed-effect model | 1.31 | 1.12 - 1.53 | 8.15E-04 |
| 858 | Complication of internal orthopedic device | injuries & poisonings | 180.41 | 0.10 | Fixed-effect model | 0.79 | 0.71 - 0.87 | 1.96E-06 |
| 859 | Complication due to other implant and internal device | injuries & poisonings | 143.41 | 0.77 | Fixed-effect model | 0.84 | 0.76 - 0.93 | 4.96E-04 |
| 870 | Open wounds of head; neck; and trunk | injuries & poisonings | 162.81 | 0.36 | Fixed-effect model | 1.19 | 1.09 - 1.31 | 1.02E-04 |
| 870.3 | Other open wound of head and face | injuries & poisonings | 153.35 | 0.57 | Fixed-effect model | 1.14 | 1.03 - 1.26 | 0.01 |
| 871 | Open wounds of extremities | injuries & poisonings | 153.44 | 0.57 | Fixed-effect model | 1.09 | 1.00 - 1.18 | 0.05 |
| 907 | Injuries to the nervous system | injuries & poisonings | 194.41 | 0.02 | Random effect model | 0.96 | 0.81 - 1.13 | 0.62 |
| 915 | Superficial injury without mention of infection | injuries & poisonings | 168.41 | 0.25 | Fixed-effect model | 1.08 | 0.99 - 1.17 | 0.08 |
| 916 | Contusion | injuries & poisonings | 160.23 | 0.41 | Fixed-effect model | 1.14 | 0.99 - 1.32 | 0.07 |
| 939 | Atopic/contact dermatitis due to other or unspecified | dermatologic | 160.61 | 0.41 | Fixed-effect model | 1.12 | 0.99 - 1.26 | 0.06 |
| 946 | Anaphylactic shock NOS | injuries & poisonings | 141.98 | 0.80 | Fixed-effect model | 1.00 | 0.79 - 1.27 | 0.99 |
| 960 | Poisoning by antibiotics | injuries & poisonings | 204.82 | 0.01 | Random effect model | 0.94 | 0.89 - 0.98 | 0.01 |
| 960.2 | Allergy/adverse effect of penicillin | injuries & poisonings | 189.16 | 0.04 | Random effect model | 0.92 | 0.87 - 0.96 | 5.92E-04 |
| 961 | Poisoning by other antiinfectives | injuries & poisonings | 176.17 | 0.14 | Fixed-effect model | 1.26 | 1.07 - 1.47 | 4.17E-03 |
| 961.1 | Poisoning/allergy of sulfonamides | injuries & poisonings | 164.42 | 0.33 | Fixed-effect model | 1.22 | 1.01 - 1.46 | 0.04 |
| 965 | Poisoning by analgesics, antipyretics, and antirheumatics | injuries & poisonings | 146.22 | 0.72 | Fixed-effect model | 1.05 | 0.98 - 1.12 | 0.15 |
| 965.1 | Opiates and related narcotics causing adverse effects in therapeutic use | injuries & poisonings | 118.76 | 0.99 | Fixed-effect model | 1.17 | 0.99 - 1.38 | 0.06 |
| 966 | Poisoning by anticonvulsants and anti-Parkinsonism drugs | injuries & poisonings | 173.54 | 0.17 | Fixed-effect model | 0.77 | 0.60 - 0.98 | 0.03 |
| 967 | Adverse effects of sedatives or other central nervous system depressants and anesthetics | injuries & poisonings | 122.28 | 0.98 | Fixed-effect model | 0.86 | 0.69 - 1.08 | 0.20 |
| 979 | Adverse drug events and drug allergies | injuries & poisonings | 159.84 | 0.42 | Fixed-effect model | 1.17 | 0.95 - 1.44 | 0.14 |
| 990 | Effects radiation NOS | injuries & poisonings | 160.35 | 0.41 | Fixed-effect model | 1.25 | 1.13 - 1.38 | 8.62E-06 |
| 994 | Sepsis and SIRS | infectious diseases | 130.42 | 0.94 | Fixed-effect model | 0.81 | 0.73 - 0.90 | 9.26E-05 |
| 994.2 | Sepsis | infectious diseases | 130.42 | 0.94 | Fixed-effect model | 0.81 | 0.73 - 0.90 | 9.26E-05 |
| 1000 | Burns | injuries & poisonings | 169.27 | 0.24 | Fixed-effect model | 0.96 | 0.77 - 1.21 | 0.76 |
| 1001 | Foreign body injury | injuries & poisonings | 141.39 | 0.81 | Fixed-effect model | 0.75 | 0.64 - 0.87 | 3.01E-04 |
| 1002 | Symptoms concerning nutrition, metabolism, and development | symptoms | 166.53 | 0.29 | Fixed-effect model | 0.93 | 0.86 - 1.00 | 0.05 |
| 1005 | Other symptoms | symptoms | 137.36 | 0.87 | Fixed-effect model | 0.97 | 0.82 - 1.13 | 0.66 |
| 1008 | Crushing or internal injury to organs | injuries & poisonings | 145.37 | 0.74 | Fixed-effect model | 1.14 | 0.96 - 1.36 | 0.13 |
| 1009 | Injury, NOS | injuries & poisonings | 170.41 | 0.22 | Fixed-effect model | 1.03 | 0.97 - 1.10 | 0.30 |
| 1010 | Other tests | symptoms | 157.02 | 0.48 | Fixed-effect model | 0.96 | 0.89 - 1.03 | 0.25 |
| 1011 | Complications of surgical and medical procedures | injuries & poisonings | 145.43 | 0.74 | Fixed-effect model | 0.89 | 0.84 - 0.94 | 4.74E-05 |
| 1015 | Effects of other external causes | symptoms | 166.63 | 0.28 | Fixed-effect model | 1.06 | 0.97 - 1.15 | 0.20 |
| 1019 | Other ill-defined and unknown causes of morbidity and mortality | symptoms | 169.48 | 0.23 | Fixed-effect model | 1.00 | 0.95 - 1.04 | 0.84 |

Odds ratios (ORs) with their 95% confidence intervals (CIs) represent the effect estimates on the risk of multiple non-delirium of per 10% reduction in risk for delirium by targeting sphingomyelin.

Significant threshold was set at *P*<2.46 x 10^-5^ (Bonferroni-corrected significance threshold calculated as 0.05 divided by 2034 [3 metabolites×678 diseases]). E signifies the exponent of 10 in the table.

Abbreviations: Phe-MR, phenome-wide Mendelian randomization.

**Table S9. Phe-MR analyses for the associations between O-methylascorbate and 678 diseases using the inverse-variance weighted method.**

| **PheCode** | **Phenotype Description** | **Disease Chapter** | **Q estimate** | ***P* value for Q estimate** | **IVW model** | **OR** | **95% CI** | ***P* value** |
| --- | --- | --- | --- | --- | --- | --- | --- | --- |
| 8 | Intestinal infection | infectious diseases | 8.22 | 0.77 | Fixed-effect model | 0.66 | 0.43 - 1.01 | 0.05 |
| 8.5 | Bacterial enteritis | infectious diseases | 9.76 | 0.64 | Fixed-effect model | 0.35 | 0.17 - 0.76 | 0.01 |
| 8.52 | Intestinal infection due to C. difficile | infectious diseases | 4.75 | 0.97 | Fixed-effect model | 0.05 | 0.01 - 0.25 | 1.94E-04 |
| 8.6 | Viral Enteritis | infectious diseases | 23.01 | 0.03 | Random effect model | 0.49 | 0.08 - 3.17 | 0.45 |
| 38 | Septicemia | infectious diseases | 9.64 | 0.65 | Fixed-effect model | 0.46 | 0.25 - 0.86 | 0.01 |
| 38.1 | Gram negative septicemia | infectious diseases | 5.07 | 0.96 | Fixed-effect model | 0.28 | 0.07 - 1.09 | 0.07 |
| 41 | Bacterial infection NOS | infectious diseases | 12.41 | 0.41 | Fixed-effect model | 0.82 | 0.57 - 1.18 | 0.28 |
| 41.1 | Staphylococcus infections | infectious diseases | 9.17 | 0.69 | Fixed-effect model | 0.58 | 0.29 - 1.18 | 0.13 |
| 41.2 | Streptococcus infection | infectious diseases | 11.49 | 0.49 | Fixed-effect model | 0.75 | 0.28 - 1.98 | 0.56 |
| 41.4 | E. coli | infectious diseases | 13.78 | 0.31 | Fixed-effect model | 0.53 | 0.25 - 1.13 | 0.10 |
| 70 | Viral hepatitis | infectious diseases | 8.44 | 0.75 | Fixed-effect model | 0.59 | 0.19 - 1.86 | 0.37 |
| 78 | Viral warts & HPV | infectious diseases | 6.56 | 0.89 | Fixed-effect model | 1.18 | 0.34 - 4.12 | 0.80 |
| 79 | Viral infection | infectious diseases | 19.46 | 0.08 | Fixed-effect model | 1.96 | 0.92 - 4.15 | 0.08 |
| 80 | Postoperative infection | infectious diseases | 23.17 | 0.03 | Random effect model | 1.07 | 0.47 - 2.45 | 0.87 |
| 81 | Infection/inflammation of internal prosthetic device; implant; and graft | infectious diseases | 11.33 | 0.50 | Fixed-effect model | 1.25 | 0.57 - 2.76 | 0.58 |
| 112 | Candidiasis | infectious diseases | 8.85 | 0.72 | Fixed-effect model | 0.95 | 0.40 - 2.25 | 0.90 |
| 145 | Cancer of mouth | neoplasms | 15.79 | 0.20 | Fixed-effect model | 1.04 | 0.22 - 5.01 | 0.96 |
| 149 | Cancer of larynx, pharynx, nasal cavities | neoplasms | 10.09 | 0.61 | Fixed-effect model | 0.55 | 0.11 - 2.68 | 0.45 |
| 150 | Cancer of esophagus | neoplasms | 8.96 | 0.71 | Fixed-effect model | 1.47 | 0.33 - 6.41 | 0.61 |
| 151 | Cancer of stomach | neoplasms | 14.53 | 0.27 | Fixed-effect model | 0.36 | 0.07 - 1.94 | 0.24 |
| 153 | Colorectal cancer | neoplasms | 12.83 | 0.38 | Fixed-effect model | 1.32 | 0.73 - 2.39 | 0.36 |
| 153.2 | Colon cancer | neoplasms | 11.70 | 0.47 | Fixed-effect model | 1.36 | 0.66 - 2.82 | 0.41 |
| 153.3 | Malignant neoplasm of rectum, rectosigmoid junction, and anus | neoplasms | 10.96 | 0.53 | Fixed-effect model | 1.60 | 0.67 - 3.81 | 0.29 |
| 157 | Pancreatic cancer | neoplasms | 8.11 | 0.78 | Fixed-effect model | 26.76 | 5.30 - 135.16 | 6.94E-05 |
| 158 | Neoplasm of unspecified nature of digestive system | neoplasms | 15.37 | 0.22 | Fixed-effect model | 4.04 | 1.19 - 13.77 | 0.03 |
| 159 | Malignant neoplasm of other and ill-defined sites within the digestive organs and peritoneum | neoplasms | 7.57 | 0.82 | Fixed-effect model | 0.95 | 0.56 - 1.62 | 0.85 |
| 165 | Cancer within the respiratory system | neoplasms | 7.09 | 0.85 | Fixed-effect model | 1.04 | 0.48 - 2.26 | 0.92 |
| 165.1 | Cancer of bronchus; lung | neoplasms | 2.79 | 1.00 | Fixed-effect model | 1.06 | 0.44 - 2.52 | 0.90 |
| 172 | Skin cancer | neoplasms | 12.81 | 0.38 | Fixed-effect model | 1.68 | 1.17 - 2.40 | 4.63E-03 |
| 172.1 | Melanomas of skin, dx or hx | neoplasms | 8.47 | 0.75 | Fixed-effect model | 2.75 | 1.26 - 5.97 | 0.01 |
| 172.11 | Melanomas of skin | neoplasms | 8.47 | 0.75 | Fixed-effect model | 2.75 | 1.26 - 5.97 | 0.01 |
| 172.2 | Other non-epithelial cancer of skin | neoplasms | 9.21 | 0.68 | Fixed-effect model | 1.82 | 1.24 - 2.69 | 2.48E-03 |
| 172.3 | Carcinoma in situ of skin | neoplasms | 16.65 | 0.16 | Fixed-effect model | 0.23 | 0.05 - 1.09 | 0.06 |
| 174 | Breast cancer | neoplasms | 9.21 | 0.68 | Fixed-effect model | 1.06 | 0.74 - 1.53 | 0.74 |
| 189 | Cancer of urinary organs (incl. kidney and bladder) | neoplasms | 8.80 | 0.72 | Fixed-effect model | 1.74 | 0.94 - 3.24 | 0.08 |
| 189.1 | Cancer of kidney and renal pelvis | neoplasms | 7.17 | 0.85 | Fixed-effect model | 2.06 | 0.60 - 7.06 | 0.25 |
| 189.11 | Malignant neoplasm of kidney, except pelvis | neoplasms | 6.78 | 0.87 | Fixed-effect model | 1.74 | 0.49 - 6.10 | 0.39 |
| 189.2 | Cancer of bladder | neoplasms | 9.38 | 0.67 | Fixed-effect model | 1.97 | 0.87 - 4.43 | 0.10 |
| 189.21 | Malignant neoplasm of bladder | neoplasms | 8.47 | 0.75 | Fixed-effect model | 1.71 | 0.72 - 4.08 | 0.22 |
| 191 | Manlignant and unknown neoplasms of brain and nervous system | neoplasms | 12.23 | 0.43 | Fixed-effect model | 1.51 | 0.32 - 7.01 | 0.60 |
| 191.1 | Cancer of brain and nervous system | neoplasms | 16.34 | 0.18 | Fixed-effect model | 1.30 | 0.23 - 7.25 | 0.76 |
| 195 | Cancer, suspected or other | neoplasms | 13.05 | 0.37 | Fixed-effect model | 1.30 | 0.95 - 1.77 | 0.10 |
| 195.1 | Malignant neoplasm, other | neoplasms | 12.86 | 0.38 | Fixed-effect model | 1.24 | 0.91 - 1.70 | 0.18 |
| 197 | Chemotherapy | neoplasms | 12.35 | 0.42 | Fixed-effect model | 0.92 | 0.70 - 1.21 | 0.56 |
| 198 | Secondary malignant neoplasm | neoplasms | 10.61 | 0.56 | Fixed-effect model | 0.72 | 0.47 - 1.09 | 0.12 |
| 198.1 | Secondary malignancy of lymph nodes | neoplasms | 15.35 | 0.22 | Fixed-effect model | 0.56 | 0.33 - 0.97 | 0.04 |
| 198.2 | Secondary malignancy of respiratory organs | neoplasms | 6.94 | 0.86 | Fixed-effect model | 0.33 | 0.14 - 0.77 | 0.01 |
| 198.3 | Secondary malignant neoplasm of digestive systems | neoplasms | 15.78 | 0.20 | Fixed-effect model | 0.85 | 0.31 - 2.34 | 0.75 |
| 198.4 | Secondary malignant neoplasm of liver | neoplasms | 10.65 | 0.56 | Fixed-effect model | 1.10 | 0.50 - 2.40 | 0.81 |
| 198.5 | Secondary malignancy of brain/spine | neoplasms | 11.75 | 0.47 | Fixed-effect model | 0.85 | 0.21 - 3.46 | 0.82 |
| 198.6 | Secondary malignancy of bone | neoplasms | 21.83 | 0.04 | Random effect model | 0.29 | 0.09 - 0.92 | 0.04 |
| 199 | Neoplasm of uncertain behavior | neoplasms | 14.74 | 0.26 | Fixed-effect model | 0.70 | 0.24 - 2.00 | 0.50 |
| 200 | Myeloproliferative disease | neoplasms | 22.57 | 0.03 | Random effect model | 0.59 | 0.10 - 3.30 | 0.55 |
| 202 | Cancer of other lymphoid, histiocytic tissue | neoplasms | 13.34 | 0.34 | Fixed-effect model | 1.45 | 0.63 - 3.35 | 0.39 |
| 202.2 | Non-Hodgkins lymphoma | neoplasms | 9.59 | 0.65 | Fixed-effect model | 1.55 | 0.60 - 3.98 | 0.37 |
| 202.24 | Large cell lymphoma | neoplasms | 7.17 | 0.85 | Fixed-effect model | 0.75 | 0.14 - 3.88 | 0.73 |
| 204 | Leukemia | neoplasms | 16.33 | 0.18 | Fixed-effect model | 0.64 | 0.24 - 1.70 | 0.37 |
| 204.1 | Lymphoid leukemia | neoplasms | 18.92 | 0.09 | Fixed-effect model | 4.10 | 0.79 - 21.28 | 0.09 |
| 204.12 | Lymphoid leukemia, chronic | neoplasms | 19.06 | 0.09 | Fixed-effect model | 4.14 | 0.71 - 24.12 | 0.11 |
| 204.4 | Multiple myeloma | neoplasms | 10.20 | 0.60 | Fixed-effect model | 0.70 | 0.13 - 3.76 | 0.68 |
| 208 | Benign neoplasm of colon | neoplasms | 15.22 | 0.23 | Fixed-effect model | 0.90 | 0.66 - 1.22 | 0.50 |
| 210 | Benign neoplasm of lip, oral cavity, and pharynx | neoplasms | 8.21 | 0.77 | Fixed-effect model | 0.52 | 0.15 - 1.83 | 0.31 |
| 211 | Benign neoplasm of other parts of digestive system | neoplasms | 19.09 | 0.09 | Fixed-effect model | 1.93 | 1.11 - 3.37 | 0.02 |
| 214 | Lipoma | neoplasms | 7.61 | 0.81 | Fixed-effect model | 0.66 | 0.40 - 1.09 | 0.10 |
| 214.1 | Lipoma of skin and subcutaneous tissue | neoplasms | 9.60 | 0.65 | Fixed-effect model | 0.86 | 0.48 - 1.55 | 0.63 |
| 215 | Other benign neoplasm of connective and other soft tissue | neoplasms | 7.23 | 0.84 | Fixed-effect model | 0.29 | 0.09 - 0.95 | 0.04 |
| 216 | Benign neoplasm of skin | neoplasms | 12.41 | 0.41 | Fixed-effect model | 1.44 | 0.91 - 2.26 | 0.12 |
| 217 | Vascular hamartomas and non-neoplastic nevi | neoplasms | 11.44 | 0.49 | Fixed-effect model | 2.28 | 0.48 - 10.96 | 0.30 |
| 217.1 | Nevus, non-neoplastic | neoplasms | 12.75 | 0.39 | Fixed-effect model | 3.37 | 0.67 - 16.90 | 0.14 |
| 225 | Benign neoplasm of brain and other parts of nervous system | neoplasms | 8.86 | 0.71 | Fixed-effect model | 0.63 | 0.16 - 2.50 | 0.51 |
| 225.1 | Benign neoplasm of brain, cranial nerves, meninges | neoplasms | 9.89 | 0.63 | Fixed-effect model | 0.58 | 0.14 - 2.42 | 0.46 |
| 227 | Benign neoplasm of other endocrine glands and related structures | neoplasms | 13.13 | 0.36 | Fixed-effect model | 0.56 | 0.15 - 2.12 | 0.39 |
| 228 | Hemangioma and lymphangioma, any site | neoplasms | 3.36 | 0.99 | Fixed-effect model | 2.01 | 0.75 - 5.38 | 0.16 |
| 229 | Benign neoplasm of unspecified sites | neoplasms | 5.11 | 0.95 | Fixed-effect model | 1.01 | 0.45 - 2.28 | 0.97 |
| 240 | Simple and unspecified goiter | endocrine/metabolic | 10.03 | 0.61 | Fixed-effect model | 0.48 | 0.10 - 2.40 | 0.37 |
| 241 | Nontoxic nodular goiter | endocrine/metabolic | 10.28 | 0.59 | Fixed-effect model | 1.20 | 0.37 - 3.88 | 0.76 |
| 241.2 | Nontoxic multinodular goiter | endocrine/metabolic | 8.08 | 0.78 | Fixed-effect model | 1.67 | 0.37 - 7.61 | 0.51 |
| 244 | Hypothyroidism | endocrine/metabolic | 9.47 | 0.66 | Fixed-effect model | 1.40 | 1.00 - 1.96 | 0.05 |
| 244.1 | Secondary hypothyroidism | endocrine/metabolic | 10.00 | 0.62 | Fixed-effect model | 2.96 | 0.89 - 9.80 | 0.08 |
| 244.4 | Hypothyroidism NOS | endocrine/metabolic | 10.92 | 0.54 | Fixed-effect model | 1.38 | 0.96 - 1.97 | 0.08 |
| 250 | Diabetes mellitus | endocrine/metabolic | 20.18 | 0.06 | Fixed-effect model | 0.61 | 0.45 - 0.83 | 1.48E-03 |
| 250.1 | Type 1 diabetes | endocrine/metabolic | 15.31 | 0.22 | Fixed-effect model | 1.21 | 0.55 - 2.64 | 0.63 |
| 250.2 | Type 2 diabetes | endocrine/metabolic | 18.28 | 0.11 | Fixed-effect model | 0.61 | 0.45 - 0.83 | 1.79E-03 |
| 250.23 | Type 2 diabetes with ophthalmic manifestations | endocrine/metabolic | 6.68 | 0.88 | Fixed-effect model | 0.58 | 0.19 - 1.77 | 0.34 |
| 250.24 | Type 2 diabetes with neurological manifestations | endocrine/metabolic | 9.29 | 0.68 | Fixed-effect model | 2.53 | 0.49 - 13.14 | 0.27 |
| 250.4 | Abnormal glucose | endocrine/metabolic | 8.22 | 0.77 | Fixed-effect model | 0.68 | 0.15 - 3.10 | 0.62 |
| 250.7 | Diabetic retinopathy | endocrine/metabolic | 5.80 | 0.93 | Fixed-effect model | 0.45 | 0.15 - 1.34 | 0.15 |
| 251 | Other disorders of pancreatic internal secretion | endocrine/metabolic | 9.08 | 0.70 | Fixed-effect model | 0.54 | 0.15 - 1.97 | 0.35 |
| 251.1 | Hypoglycemia | endocrine/metabolic | 8.85 | 0.72 | Fixed-effect model | 0.48 | 0.13 - 1.73 | 0.26 |
| 252 | Disorders of parathyroid gland | endocrine/metabolic | 7.36 | 0.83 | Fixed-effect model | 0.90 | 0.23 - 3.43 | 0.87 |
| 252.1 | Hyperparathyroidism | endocrine/metabolic | 6.25 | 0.90 | Fixed-effect model | 1.11 | 0.27 - 4.64 | 0.88 |
| 253 | Disorders of the pituitary gland and its hypothalamic control | endocrine/metabolic | 8.58 | 0.74 | Fixed-effect model | 0.13 | 0.03 - 0.60 | 0.01 |
| 255 | Disorders of adrenal glands | endocrine/metabolic | 12.29 | 0.42 | Fixed-effect model | 4.53 | 0.94 - 21.78 | 0.06 |
| 260 | Protein-calorie malnutrition | endocrine/metabolic | 6.01 | 0.92 | Fixed-effect model | 1.58 | 0.47 - 5.30 | 0.46 |
| 260.6 | Anorexia | endocrine/metabolic | 9.77 | 0.64 | Fixed-effect model | 1.17 | 0.30 - 4.59 | 0.83 |
| 261 | Vitamin deficiency | endocrine/metabolic | 3.55 | 0.99 | Fixed-effect model | 0.46 | 0.15 - 1.44 | 0.18 |
| 261.2 | Vitamin B-complex deficiencies | endocrine/metabolic | 4.87 | 0.96 | Fixed-effect model | 0.58 | 0.14 - 2.45 | 0.46 |
| 272 | Disorders of lipid metabolism | endocrine/metabolic | 25.31 | 0.01 | Random effect model | 0.60 | 0.43 - 0.84 | 3.02E-03 |
| 272.1 | Hyperlipidemia | endocrine/metabolic | 25.34 | 0.01 | Random effect model | 0.60 | 0.43 - 0.84 | 3.00E-03 |
| 272.11 | Hypercholesterolemia | endocrine/metabolic | 25.54 | 0.01 | Random effect model | 0.64 | 0.45 - 0.90 | 0.01 |
| 274 | Gout and other crystal arthropathies | endocrine/metabolic | 6.24 | 0.90 | Fixed-effect model | 0.49 | 0.26 - 0.94 | 0.03 |
| 274.1 | Gout | endocrine/metabolic | 5.92 | 0.92 | Fixed-effect model | 0.60 | 0.30 - 1.22 | 0.16 |
| 274.2 | Crystal arthropathies | endocrine/metabolic | 11.19 | 0.51 | Fixed-effect model | 0.14 | 0.03 - 0.71 | 0.02 |
| 274.21 | Chondrocalcinosis | endocrine/metabolic | 6.69 | 0.88 | Fixed-effect model | 0.10 | 0.02 - 0.51 | 0.01 |
| 275 | Disorders of mineral metabolism | endocrine/metabolic | 6.77 | 0.87 | Fixed-effect model | 0.44 | 0.19 - 1.05 | 0.06 |
| 275.1 | Disorders of iron metabolism | hematopoietic | 12.69 | 0.39 | Fixed-effect model | 0.83 | 0.18 - 3.83 | 0.81 |
| 275.5 | Disorders of calcium/phosphorus metabolism | endocrine/metabolic | 4.91 | 0.96 | Fixed-effect model | 0.18 | 0.06 - 0.56 | 3.28E-03 |
| 276 | Disorders of fluid, electrolyte, and acid-base balance | endocrine/metabolic | 8.57 | 0.74 | Fixed-effect model | 0.77 | 0.48 - 1.24 | 0.29 |
| 276.1 | Electrolyte imbalance | endocrine/metabolic | 6.24 | 0.90 | Fixed-effect model | 0.68 | 0.37 - 1.26 | 0.22 |
| 276.13 | Hyperpotassemia | endocrine/metabolic | 10.11 | 0.61 | Fixed-effect model | 0.42 | 0.12 - 1.48 | 0.17 |
| 276.14 | Hypopotassemia | endocrine/metabolic | 12.61 | 0.40 | Fixed-effect model | 0.62 | 0.22 - 1.76 | 0.37 |
| 276.4 | Acid-base balance disorder | endocrine/metabolic | 8.54 | 0.74 | Fixed-effect model | 2.23 | 0.69 - 7.19 | 0.18 |
| 276.41 | Acidosis | endocrine/metabolic | 9.47 | 0.66 | Fixed-effect model | 2.30 | 0.67 - 7.84 | 0.18 |
| 276.5 | Hypovolemia | endocrine/metabolic | 7.56 | 0.82 | Fixed-effect model | 0.47 | 0.22 - 0.99 | 0.05 |
| 277 | Other disorders of metabolism | endocrine/metabolic | 6.00 | 0.92 | Fixed-effect model | 0.36 | 0.13 - 1.05 | 0.06 |
| 278 | Overweight, obesity and other hyperalimentation | endocrine/metabolic | 11.64 | 0.48 | Fixed-effect model | 0.48 | 0.32 - 0.71 | 2.17E-04 |
| 278.1 | Obesity | endocrine/metabolic | 10.99 | 0.53 | Fixed-effect model | 0.49 | 0.33 - 0.73 | 3.73E-04 |
| 280 | Iron deficiency anemias | hematopoietic | 5.55 | 0.94 | Fixed-effect model | 0.96 | 0.61 - 1.51 | 0.85 |
| 280.1 | Iron deficiency anemias, unspecified or not due to blood loss | hematopoietic | 4.86 | 0.96 | Fixed-effect model | 1.01 | 0.63 - 1.63 | 0.95 |
| 281 | Other deficiency anemia | hematopoietic | 6.64 | 0.88 | Fixed-effect model | 0.28 | 0.09 - 0.89 | 0.03 |
| 281.1 | Megaloblastic anemia | hematopoietic | 6.38 | 0.90 | Fixed-effect model | 0.33 | 0.10 - 1.09 | 0.07 |
| 281.11 | Pernicious anemia | hematopoietic | 6.93 | 0.86 | Fixed-effect model | 0.15 | 0.03 - 0.62 | 0.01 |
| 285 | Other anemias | hematopoietic | 11.89 | 0.45 | Fixed-effect model | 1.04 | 0.72 - 1.49 | 0.84 |
| 285.2 | Anemia of chronic disease | hematopoietic | 6.90 | 0.86 | Fixed-effect model | 1.49 | 0.34 - 6.55 | 0.60 |
| 286 | Coagulation defects | hematopoietic | 11.13 | 0.52 | Fixed-effect model | 1.06 | 0.29 - 3.88 | 0.92 |
| 287 | Purpura and other hemorrhagic conditions | hematopoietic | 21.73 | 0.04 | Random effect model | 0.46 | 0.13 - 1.65 | 0.24 |
| 287.3 | Thrombocytopenia | hematopoietic | 20.93 | 0.05 | Fixed-effect model | 0.36 | 0.13 - 0.98 | 0.05 |
| 288 | Diseases of white blood cells | hematopoietic | 14.80 | 0.25 | Fixed-effect model | 0.45 | 0.24 - 0.86 | 0.02 |
| 288.1 | Decreased white blood cell count | hematopoietic | 11.27 | 0.51 | Fixed-effect model | 0.45 | 0.22 - 0.91 | 0.03 |
| 288.11 | Neutropenia | hematopoietic | 11.27 | 0.51 | Fixed-effect model | 0.45 | 0.22 - 0.91 | 0.03 |
| 289 | Other diseases of blood and blood-forming organs | hematopoietic | 9.20 | 0.69 | Fixed-effect model | 0.72 | 0.39 - 1.34 | 0.30 |
| 289.4 | Lymphadenitis | hematopoietic | 5.76 | 0.93 | Fixed-effect model | 0.72 | 0.33 - 1.57 | 0.41 |
| 289.5 | Diseases of spleen | hematopoietic | 9.34 | 0.67 | Fixed-effect model | 0.45 | 0.08 - 2.60 | 0.38 |
| 290.1 | Dementias | mental disorders | 6.18 | 0.91 | Fixed-effect model | 0.82 | 0.23 - 2.97 | 0.76 |
| 291 | Other specified nonpsychotic and/or transient mental disorders | mental disorders | 7.20 | 0.84 | Fixed-effect model | 1.21 | 0.25 - 5.81 | 0.81 |
| 292 | Neurological disorders | mental disorders | 4.87 | 0.96 | Fixed-effect model | 0.68 | 0.38 - 1.22 | 0.19 |
| 292.1 | Aphasia/speech disturbance | mental disorders | 6.50 | 0.89 | Fixed-effect model | 0.85 | 0.31 - 2.34 | 0.75 |
| 292.3 | Memory loss | mental disorders | 16.52 | 0.17 | Fixed-effect model | 1.77 | 0.39 - 7.95 | 0.46 |
| 292.4 | Altered mental status | mental disorders | 11.41 | 0.49 | Fixed-effect model | 0.28 | 0.12 - 0.64 | 2.73E-03 |
| 293 | Symptoms involving head and neck | mental disorders | 15.69 | 0.21 | Fixed-effect model | 1.78 | 0.86 - 3.68 | 0.12 |
| 293.1 | Swelling, mass, or lump in head and neck [Space occupying lesion, intracranial NOS] | mental disorders | 7.07 | 0.85 | Fixed-effect model | 3.03 | 0.81 - 11.29 | 0.10 |
| 295 | Schizophrenia and other psychotic disorders | mental disorders | 16.81 | 0.16 | Fixed-effect model | 2.65 | 0.68 - 10.35 | 0.16 |
| 295.1 | Schizophrenia | mental disorders | 14.19 | 0.29 | Fixed-effect model | 7.84 | 1.51 - 40.77 | 0.01 |
| 296 | Mood disorders | mental disorders | 13.35 | 0.34 | Fixed-effect model | 1.30 | 0.90 - 1.87 | 0.16 |
| 296.1 | Bipolar | mental disorders | 7.23 | 0.84 | Fixed-effect model | 4.05 | 1.19 - 13.82 | 0.03 |
| 296.2 | Depression | mental disorders | 14.23 | 0.29 | Fixed-effect model | 1.27 | 0.88 - 1.84 | 0.20 |
| 300 | Anxiety disorders | mental disorders | 7.59 | 0.82 | Fixed-effect model | 0.99 | 0.61 - 1.60 | 0.97 |
| 300.1 | Anxiety disorder | mental disorders | 6.52 | 0.89 | Fixed-effect model | 1.05 | 0.63 - 1.73 | 0.85 |
| 300.12 | Agorophobia, social phobia, and panic disorder | mental disorders | 17.44 | 0.13 | Fixed-effect model | 0.92 | 0.21 - 4.05 | 0.91 |
| 300.13 | Phobia | mental disorders | 7.09 | 0.85 | Fixed-effect model | 3.37 | 0.58 - 19.64 | 0.18 |
| 303 | Psychogenic and somatoform disorders | mental disorders | 9.00 | 0.70 | Fixed-effect model | 0.20 | 0.04 - 1.13 | 0.07 |
| 306 | Other mental disorder | mental disorders | 7.26 | 0.84 | Fixed-effect model | 0.52 | 0.40 - 0.66 | 2.78E-07 |
| 317 | Alcohol-related disorders | mental disorders | 9.27 | 0.68 | Fixed-effect model | 1.08 | 0.75 - 1.55 | 0.68 |
| 317.1 | Alcoholism | mental disorders | 12.25 | 0.43 | Fixed-effect model | 0.86 | 0.56 - 1.31 | 0.49 |
| 317.11 | Alcoholic liver damage | mental disorders | 16.23 | 0.18 | Fixed-effect model | 0.17 | 0.04 - 0.67 | 0.01 |
| 318 | Tobacco use disorder | mental disorders | 13.95 | 0.30 | Fixed-effect model | 0.88 | 0.65 - 1.19 | 0.41 |
| 327 | Sleep disorders | neurological | 6.36 | 0.90 | Fixed-effect model | 0.62 | 0.36 - 1.09 | 0.10 |
| 327.3 | Sleep apnea | neurological | 6.09 | 0.91 | Fixed-effect model | 0.53 | 0.29 - 0.95 | 0.03 |
| 331 | Other cerebral degenerations | neurological | 11.37 | 0.50 | Fixed-effect model | 0.74 | 0.22 - 2.43 | 0.61 |
| 332 | Parkinson's disease | neurological | 14.72 | 0.26 | Fixed-effect model | 0.46 | 0.14 - 1.48 | 0.19 |
| 333 | Extrapyramidal disease and abnormal movement disorders | neurological | 14.44 | 0.27 | Fixed-effect model | 3.28 | 0.87 - 12.30 | 0.08 |
| 334 | Degenerative disease of the spinal cord | neurological | 10.41 | 0.58 | Fixed-effect model | 0.48 | 0.19 - 1.24 | 0.13 |
| 335 | Multiple sclerosis | neurological | 8.65 | 0.73 | Fixed-effect model | 0.62 | 0.21 - 1.85 | 0.39 |
| 338 | Pain | symptoms | 14.06 | 0.30 | Fixed-effect model | 3.11 | 0.75 - 12.98 | 0.12 |
| 339 | Other headache syndromes | neurological | 10.76 | 0.55 | Fixed-effect model | 1.05 | 0.67 - 1.65 | 0.84 |
| 340 | Migraine | neurological | 10.68 | 0.56 | Fixed-effect model | 1.47 | 0.69 - 3.11 | 0.32 |
| 342 | Hemiplegia | neurological | 4.14 | 0.98 | Fixed-effect model | 1.05 | 0.38 - 2.96 | 0.92 |
| 344 | Other paralytic syndromes | neurological | 5.97 | 0.92 | Fixed-effect model | 0.53 | 0.11 - 2.44 | 0.41 |
| 345 | Epilepsy, recurrent seizures, convulsions | neurological | 12.87 | 0.38 | Fixed-effect model | 0.76 | 0.43 - 1.33 | 0.34 |
| 345.1 | Epilepsy | neurological | 18.67 | 0.10 | Fixed-effect model | 0.46 | 0.12 - 1.70 | 0.24 |
| 345.3 | Convulsions | neurological | 13.37 | 0.34 | Fixed-effect model | 0.77 | 0.33 - 1.79 | 0.55 |
| 348 | Other conditions of brain | neurological | 13.35 | 0.34 | Fixed-effect model | 0.86 | 0.30 - 2.46 | 0.77 |
| 350 | Abnormal movement | neurological | 7.46 | 0.83 | Fixed-effect model | 0.36 | 0.17 - 0.77 | 0.01 |
| 350.1 | Abnormal involuntary movements | neurological | 8.63 | 0.73 | Fixed-effect model | 0.86 | 0.22 - 3.25 | 0.82 |
| 350.2 | Abnormality of gait | neurological | 12.62 | 0.40 | Fixed-effect model | 0.23 | 0.09 - 0.61 | 3.25E-03 |
| 351 | Other peripheral nerve disorders | neurological | 11.79 | 0.46 | Fixed-effect model | 0.93 | 0.64 - 1.34 | 0.69 |
| 352 | Disorders of other cranial nerves | neurological | 4.32 | 0.98 | Fixed-effect model | 0.58 | 0.20 - 1.67 | 0.31 |
| 352.2 | Facial nerve disorders [CN7] | neurological | 7.30 | 0.84 | Fixed-effect model | 0.50 | 0.13 - 1.90 | 0.31 |
| 353 | Nerve root and plexus disorders | neurological | 21.42 | 0.04 | Random effect model | 1.52 | 0.32 - 7.29 | 0.60 |
| 357 | Inflammatory and toxic neuropathy | neurological | 12.19 | 0.43 | Fixed-effect model | 0.95 | 0.33 - 2.78 | 0.93 |
| 361 | Retinal detachments and defects | sense organs | 9.15 | 0.69 | Fixed-effect model | 1.49 | 0.74 - 3.00 | 0.27 |
| 361.1 | Retinal detachment with retinal defect | sense organs | 15.20 | 0.23 | Fixed-effect model | 0.65 | 0.22 - 1.88 | 0.43 |
| 362 | Other retinal disorders | sense organs | 12.84 | 0.38 | Fixed-effect model | 0.68 | 0.36 - 1.29 | 0.24 |
| 362.2 | Degeneration of macula and posterior pole of retina | sense organs | 10.41 | 0.58 | Fixed-effect model | 0.57 | 0.24 - 1.33 | 0.19 |
| 362.29 | Macular degeneration (senile) of retina NOS | sense organs | 10.28 | 0.59 | Fixed-effect model | 0.57 | 0.24 - 1.33 | 0.19 |
| 362.4 | Retinal vascular changes and abnomalities | sense organs | 10.49 | 0.57 | Fixed-effect model | 0.54 | 0.14 - 2.11 | 0.38 |
| 364 | Corneal opacity and other disorders of cornea | sense organs | 19.65 | 0.07 | Fixed-effect model | 0.98 | 0.23 - 4.20 | 0.98 |
| 365 | Glaucoma | sense organs | 12.00 | 0.45 | Fixed-effect model | 0.84 | 0.46 - 1.55 | 0.59 |
| 365.1 | Open-angle glaucoma | sense organs | 15.81 | 0.20 | Fixed-effect model | 0.32 | 0.09 - 1.09 | 0.07 |
| 365.11 | Primary open angle glaucoma | sense organs | 17.35 | 0.14 | Fixed-effect model | 0.30 | 0.09 - 1.02 | 0.05 |
| 365.2 | Primary angle-closure glaucoma | sense organs | 3.55 | 0.99 | Fixed-effect model | 0.33 | 0.07 - 1.44 | 0.14 |
| 366 | Cataract | sense organs | 14.86 | 0.25 | Fixed-effect model | 1.01 | 0.75 - 1.37 | 0.95 |
| 366.2 | Senile cataract | sense organs | 12.35 | 0.42 | Fixed-effect model | 1.05 | 0.67 - 1.64 | 0.83 |
| 367 | Disorders of refraction and accommodation; blindness and low vision | sense organs | 5.85 | 0.92 | Fixed-effect model | 2.53 | 1.13 - 5.68 | 0.02 |
| 367.1 | Myopia | sense organs | 7.06 | 0.85 | Fixed-effect model | 2.30 | 0.75 - 7.06 | 0.14 |
| 367.9 | Blindness and low vision | sense organs | 10.36 | 0.58 | Fixed-effect model | 1.34 | 0.31 - 5.87 | 0.70 |
| 368 | Visual disturbances | sense organs | 18.70 | 0.10 | Fixed-effect model | 1.19 | 0.59 - 2.40 | 0.62 |
| 368.1 | Amblyopia | sense organs | 6.41 | 0.89 | Fixed-effect model | 2.29 | 0.41 - 12.74 | 0.34 |
| 368.2 | Diplopia and disorders of binocular vision | sense organs | 15.80 | 0.20 | Fixed-effect model | 0.35 | 0.08 - 1.52 | 0.16 |
| 368.9 | Subjective visual disturbances | sense organs | 21.48 | 0.04 | Random effect model | 0.57 | 0.07 - 4.83 | 0.61 |
| 369 | Infection of the eye | sense organs | 7.06 | 0.85 | Fixed-effect model | 0.81 | 0.15 - 4.30 | 0.80 |
| 371 | Inflammation of the eye | sense organs | 8.52 | 0.74 | Fixed-effect model | 0.91 | 0.45 - 1.84 | 0.79 |
| 371.3 | Inflammation of eyelids | sense organs | 8.68 | 0.73 | Fixed-effect model | 1.37 | 0.61 - 3.07 | 0.45 |
| 372 | Disorders of conjunctiva | sense organs | 21.07 | 0.05 | Random effect model | 1.82 | 0.27 - 12.12 | 0.54 |
| 374 | Other disorders of eyelids | sense organs | 6.23 | 0.90 | Fixed-effect model | 0.43 | 0.25 - 0.73 | 1.67E-03 |
| 374.1 | Ectropion or entropion | sense organs | 7.85 | 0.80 | Fixed-effect model | 0.41 | 0.12 - 1.38 | 0.15 |
| 375 | Disorders of lacrimal system | sense organs | 16.53 | 0.17 | Fixed-effect model | 0.35 | 0.15 - 0.82 | 0.02 |
| 375.2 | Epiphora | sense organs | 18.82 | 0.09 | Fixed-effect model | 0.43 | 0.12 - 1.62 | 0.21 |
| 378 | Strabismus and other disorders of binocular eye movements | sense organs | 9.98 | 0.62 | Fixed-effect model | 1.68 | 0.60 - 4.76 | 0.33 |
| 378.1 | Strabismus (not specified as paralytic) | sense organs | 7.23 | 0.84 | Fixed-effect model | 1.23 | 0.33 - 4.58 | 0.75 |
| 379 | Other disorders of eye | sense organs | 27.40 | 0.01 | Random effect model | 1.53 | 0.58 - 4.02 | 0.39 |
| 379.2 | Disorders of vitreous body | sense organs | 5.34 | 0.95 | Fixed-effect model | 2.54 | 0.87 - 7.38 | 0.09 |
| 379.3 | Aphakia and other disorders of lens | sense organs | 15.92 | 0.19 | Fixed-effect model | 1.53 | 0.60 - 3.87 | 0.37 |
| 380 | Disorders of external ear | sense organs | 8.48 | 0.75 | Fixed-effect model | 1.16 | 0.41 - 3.27 | 0.78 |
| 380.1 | Otitis externa | sense organs | 5.57 | 0.94 | Fixed-effect model | 2.23 | 0.39 - 12.62 | 0.37 |
| 381 | Otitis media and Eustachian tube disorders | sense organs | 5.66 | 0.93 | Fixed-effect model | 0.46 | 0.20 - 1.06 | 0.07 |
| 381.1 | Otitis media | sense organs | 6.64 | 0.88 | Fixed-effect model | 0.56 | 0.22 - 1.42 | 0.22 |
| 381.11 | Suppurative and unspecified otitis media | sense organs | 15.12 | 0.24 | Fixed-effect model | 0.71 | 0.18 - 2.78 | 0.62 |
| 384 | Other disorders of tympanic membrane | sense organs | 15.36 | 0.22 | Fixed-effect model | 2.38 | 0.82 - 6.94 | 0.11 |
| 384.4 | Perforation of tympanic membrane | sense organs | 9.45 | 0.66 | Fixed-effect model | 5.61 | 1.64 - 19.20 | 0.01 |
| 385 | Other disorders of middle ear and mastoid | sense organs | 7.55 | 0.82 | Fixed-effect model | 0.47 | 0.12 - 1.86 | 0.28 |
| 385.3 | Cholesteatoma | sense organs | 7.57 | 0.82 | Fixed-effect model | 1.17 | 0.23 - 5.87 | 0.85 |
| 386 | Vertiginous syndromes and other disorders of vestibular system | sense organs | 16.86 | 0.15 | Fixed-effect model | 0.81 | 0.49 - 1.35 | 0.43 |
| 386.1 | Meniere's disease | sense organs | 13.38 | 0.34 | Fixed-effect model | 8.75 | 1.69 - 45.43 | 0.01 |
| 386.3 | Labyrinthitis | sense organs | 14.10 | 0.29 | Fixed-effect model | 0.89 | 0.21 - 3.72 | 0.87 |
| 386.9 | Dizziness and giddiness (Light-headedness and vertigo) | sense organs | 12.78 | 0.39 | Fixed-effect model | 0.63 | 0.35 - 1.14 | 0.13 |
| 389 | Hearing loss | sense organs | 10.17 | 0.60 | Fixed-effect model | 0.54 | 0.29 - 1.01 | 0.05 |
| 389.4 | Tinnitus | sense organs | 7.89 | 0.79 | Fixed-effect model | 4.34 | 0.76 - 24.90 | 0.10 |
| 394 | Rheumatic disease of the heart valves | circulatory system | 4.61 | 0.97 | Fixed-effect model | 0.43 | 0.24 - 0.77 | 4.20E-03 |
| 394.2 | Mitral valve disease | circulatory system | 7.00 | 0.86 | Fixed-effect model | 0.37 | 0.18 - 0.76 | 0.01 |
| 394.3 | Aortic valve disease | circulatory system | 7.07 | 0.85 | Fixed-effect model | 0.90 | 0.29 - 2.75 | 0.85 |
| 394.7 | Disease of tricuspid valve | circulatory system | 3.70 | 0.99 | Fixed-effect model | 0.53 | 0.16 - 1.77 | 0.30 |
| 395 | Heart valve disorders | circulatory system | 7.95 | 0.79 | Fixed-effect model | 0.42 | 0.23 - 0.79 | 0.01 |
| 395.1 | Nonrheumatic mitral valve disorders | circulatory system | 8.23 | 0.77 | Fixed-effect model | 0.39 | 0.19 - 0.81 | 0.01 |
| 395.6 | Heart valve replaced | circulatory system | 16.56 | 0.17 | Fixed-effect model | 0.74 | 0.26 - 2.08 | 0.57 |
| 396 | Abnormal heart sounds | circulatory system | 19.03 | 0.09 | Fixed-effect model | 0.98 | 0.29 - 3.34 | 0.97 |
| 401 | Hypertension | circulatory system | 16.26 | 0.18 | Fixed-effect model | 0.61 | 0.51 - 0.73 | 5.47E-08 |
| 401.1 | Essential hypertension | circulatory system | 16.78 | 0.16 | Fixed-effect model | 0.61 | 0.51 - 0.72 | 3.87E-08 |
| 401.2 | Hypertensive heart and/or renal disease | circulatory system | 13.19 | 0.36 | Fixed-effect model | 0.58 | 0.22 - 1.50 | 0.26 |
| 401.22 | Hypertensive chronic kidney disease | circulatory system | 10.38 | 0.58 | Fixed-effect model | 0.58 | 0.21 - 1.58 | 0.29 |
| 402 | Elevated blood pressure reading without diagnosis of hypertension | circulatory system | 5.79 | 0.93 | Fixed-effect model | 0.63 | 0.22 - 1.77 | 0.38 |
| 411 | Ischemic Heart Disease | circulatory system | 11.06 | 0.52 | Fixed-effect model | 0.75 | 0.58 - 0.96 | 0.02 |
| 411.1 | Unstable angina (intermediate coronary syndrome) | circulatory system | 12.95 | 0.37 | Fixed-effect model | 0.50 | 0.29 - 0.88 | 0.02 |
| 411.2 | Myocardial infarction | circulatory system | 9.24 | 0.68 | Fixed-effect model | 0.72 | 0.49 - 1.06 | 0.10 |
| 411.3 | Angina pectoris | circulatory system | 13.22 | 0.35 | Fixed-effect model | 0.68 | 0.49 - 0.96 | 0.03 |
| 411.4 | Coronary atherosclerosis | circulatory system | 7.92 | 0.79 | Fixed-effect model | 0.57 | 0.42 - 0.77 | 2.64E-04 |
| 411.41 | Aneurysm and dissection of heart | circulatory system | 9.24 | 0.68 | Fixed-effect model | 0.30 | 0.07 - 1.36 | 0.12 |
| 411.8 | Other chronic ischemic heart disease, unspecified | circulatory system | 16.38 | 0.17 | Fixed-effect model | 0.92 | 0.65 - 1.29 | 0.62 |
| 411.9 | Other acute and subacute forms of ischemic heart disease | circulatory system | 4.77 | 0.97 | Fixed-effect model | 0.85 | 0.26 - 2.73 | 0.78 |
| 414 | Other forms of chronic heart disease | circulatory system | 17.67 | 0.13 | Fixed-effect model | 0.29 | 0.11 - 0.74 | 0.01 |
| 415 | Pulmonary heart disease | circulatory system | 18.34 | 0.11 | Fixed-effect model | 1.03 | 0.56 - 1.91 | 0.92 |
| 415.2 | Chronic pulmonary heart disease | circulatory system | 6.44 | 0.89 | Fixed-effect model | 0.25 | 0.05 - 1.29 | 0.10 |
| 416 | Cardiomegaly | circulatory system | 12.20 | 0.43 | Fixed-effect model | 0.37 | 0.17 - 0.82 | 0.01 |
| 418 | Nonspecific chest pain | circulatory system | 20.84 | 0.05 | Fixed-effect model | 0.74 | 0.58 - 0.94 | 0.01 |
| 418.1 | Precordial pain | circulatory system | 15.17 | 0.23 | Fixed-effect model | 0.77 | 0.40 - 1.51 | 0.45 |
| 420.2 | Pericarditis | circulatory system | 9.60 | 0.65 | Fixed-effect model | 0.42 | 0.14 - 1.27 | 0.12 |
| 420.3 | Endocarditis | circulatory system | 8.02 | 0.78 | Fixed-effect model | 0.31 | 0.07 - 1.40 | 0.13 |
| 425 | Cardiomyopathy | circulatory system | 11.59 | 0.48 | Fixed-effect model | 0.31 | 0.10 - 0.94 | 0.04 |
| 425.1 | Primary/intrinsic cardiomyopathies | circulatory system | 12.07 | 0.44 | Fixed-effect model | 0.29 | 0.09 - 0.90 | 0.03 |
| 426 | Cardiac conduction disorders | circulatory system | 9.16 | 0.69 | Fixed-effect model | 0.56 | 0.35 - 0.91 | 0.02 |
| 426.2 | Atrioventricular [AV] block | circulatory system | 8.89 | 0.71 | Fixed-effect model | 0.30 | 0.13 - 0.71 | 0.01 |
| 426.21 | First degree AV block | circulatory system | 13.30 | 0.35 | Fixed-effect model | 0.24 | 0.07 - 0.84 | 0.03 |
| 426.24 | Atrioventricular block, complete | circulatory system | 9.97 | 0.62 | Fixed-effect model | 0.21 | 0.04 - 1.07 | 0.06 |
| 426.3 | Bundle branch block | circulatory system | 9.18 | 0.69 | Fixed-effect model | 0.41 | 0.21 - 0.82 | 0.01 |
| 426.31 | Right bundle branch block | circulatory system | 19.19 | 0.08 | Fixed-effect model | 0.68 | 0.24 - 1.91 | 0.46 |
| 426.32 | Left bundle branch block | circulatory system | 14.03 | 0.30 | Fixed-effect model | 0.23 | 0.09 - 0.61 | 2.76E-03 |
| 426.9 | Cardiac pacemaker/device in situ | circulatory system | 8.94 | 0.71 | Fixed-effect model | 0.74 | 0.33 - 1.66 | 0.47 |
| 426.91 | Cardiac pacemaker in situ | circulatory system | 8.54 | 0.74 | Fixed-effect model | 0.74 | 0.32 - 1.71 | 0.48 |
| 427 | Cardiac dysrhythmias | circulatory system | 6.30 | 0.90 | Fixed-effect model | 0.65 | 0.50 - 0.85 | 1.76E-03 |
| 427.1 | Paroxysmal tachycardia, unspecified | circulatory system | 16.61 | 0.16 | Fixed-effect model | 0.38 | 0.19 - 0.77 | 0.01 |
| 427.11 | Paroxysmal supraventricular tachycardia | circulatory system | 19.49 | 0.08 | Fixed-effect model | 0.40 | 0.18 - 0.90 | 0.03 |
| 427.12 | Paroxysmal ventricular tachycardia | circulatory system | 3.57 | 0.99 | Fixed-effect model | 0.42 | 0.12 - 1.54 | 0.19 |
| 427.2 | Atrial fibrillation and flutter | circulatory system | 8.45 | 0.75 | Fixed-effect model | 0.74 | 0.52 - 1.04 | 0.08 |
| 427.3 | Other specified cardiac dysrhythmias | circulatory system | 11.93 | 0.45 | Fixed-effect model | 0.30 | 0.15 - 0.60 | 6.71E-04 |
| 427.4 | Cardiac arrest and ventricular fibrillation | circulatory system | 11.95 | 0.45 | Fixed-effect model | 0.51 | 0.16 - 1.64 | 0.25 |
| 427.42 | Cardiac arrest | circulatory system | 17.11 | 0.15 | Fixed-effect model | 0.59 | 0.16 - 2.15 | 0.42 |
| 427.5 | Arrhythmia (cardiac) NOS | circulatory system | 15.82 | 0.20 | Fixed-effect model | 1.30 | 0.35 - 4.82 | 0.70 |
| 427.6 | Premature beats | circulatory system | 18.53 | 0.10 | Fixed-effect model | 0.38 | 0.07 - 2.13 | 0.27 |
| 427.7 | Tachycardia NOS | circulatory system | 16.98 | 0.15 | Fixed-effect model | 1.59 | 0.68 - 3.70 | 0.28 |
| 427.9 | Palpitations | circulatory system | 10.13 | 0.60 | Fixed-effect model | 0.76 | 0.40 - 1.45 | 0.40 |
| 428 | Congestive heart failure; nonhypertensive | circulatory system | 6.54 | 0.89 | Fixed-effect model | 0.36 | 0.21 - 0.62 | 2.90E-04 |
| 428.2 | Heart failure NOS | circulatory system | 7.65 | 0.81 | Fixed-effect model | 0.36 | 0.20 - 0.67 | 1.32E-03 |
| 429 | Ill-defined descriptions and complications of heart disease | circulatory system | 17.35 | 0.14 | Fixed-effect model | 9.45 | 2.32 - 38.43 | 1.71E-03 |
| 429.2 | Abnormal function study of cardiovascular system | circulatory system | 10.89 | 0.54 | Fixed-effect model | 5.42 | 0.96 - 30.74 | 0.06 |
| 430 | Intracranial hemorrhage | circulatory system | 9.38 | 0.67 | Fixed-effect model | 0.36 | 0.14 - 0.94 | 0.04 |
| 430.1 | Subarachnoid hemorrhage | circulatory system | 8.06 | 0.78 | Fixed-effect model | 0.49 | 0.12 - 1.99 | 0.32 |
| 430.2 | Intracerebral hemorrhage | circulatory system | 14.72 | 0.26 | Fixed-effect model | 0.58 | 0.13 - 2.63 | 0.48 |
| 433 | Cerebrovascular disease | circulatory system | 13.52 | 0.33 | Fixed-effect model | 0.87 | 0.57 - 1.33 | 0.52 |
| 433.1 | Occlusion and stenosis of precerebral arteries | circulatory system | 11.44 | 0.49 | Fixed-effect model | 0.87 | 0.28 - 2.74 | 0.81 |
| 433.2 | Occlusion of cerebral arteries | circulatory system | 6.73 | 0.87 | Fixed-effect model | 0.95 | 0.51 - 1.77 | 0.87 |
| 433.21 | Cerebral artery occlusion, with cerebral infarction | circulatory system | 5.68 | 0.93 | Fixed-effect model | 0.60 | 0.21 - 1.69 | 0.33 |
| 433.3 | Cerebral ischemia | circulatory system | 10.93 | 0.54 | Fixed-effect model | 0.59 | 0.29 - 1.23 | 0.16 |
| 433.31 | Transient cerebral ischemia | circulatory system | 5.68 | 0.93 | Fixed-effect model | 0.92 | 0.39 - 2.18 | 0.85 |
| 433.8 | Late effects of cerebrovascular disease | circulatory system | 10.87 | 0.54 | Fixed-effect model | 0.96 | 0.31 - 2.93 | 0.94 |
| 440 | Atherosclerosis | circulatory system | 11.42 | 0.49 | Fixed-effect model | 0.81 | 0.27 - 2.41 | 0.70 |
| 440.2 | Atherosclerosis of the extremities | circulatory system | 11.44 | 0.49 | Fixed-effect model | 0.55 | 0.14 - 2.24 | 0.41 |
| 441 | Vascular insufficiency of intestine | circulatory system | 15.87 | 0.20 | Fixed-effect model | 0.55 | 0.11 - 2.88 | 0.48 |
| 442 | Other aneurysm | circulatory system | 9.97 | 0.62 | Fixed-effect model | 0.32 | 0.12 - 0.82 | 0.02 |
| 442.1 | Aortic aneurysm | circulatory system | 13.50 | 0.33 | Fixed-effect model | 0.27 | 0.09 - 0.79 | 0.02 |
| 442.11 | Abdominal aortic aneurysm | circulatory system | 19.48 | 0.08 | Fixed-effect model | 0.35 | 0.09 - 1.35 | 0.13 |
| 443 | Peripheral vascular disease | circulatory system | 16.74 | 0.16 | Fixed-effect model | 0.51 | 0.27 - 0.97 | 0.04 |
| 443.1 | Raynaud's syndrome | circulatory system | 11.09 | 0.52 | Fixed-effect model | 0.40 | 0.12 - 1.28 | 0.12 |
| 443.9 | Peripheral vascular disease, unspecified | circulatory system | 19.53 | 0.08 | Fixed-effect model | 0.70 | 0.32 - 1.53 | 0.37 |
| 444 | Arterial embolism and thrombosis | circulatory system | 6.44 | 0.89 | Fixed-effect model | 1.47 | 0.39 - 5.45 | 0.57 |
| 444.1 | Arterial embolism and thrombosis of lower extremity artery | circulatory system | 6.22 | 0.90 | Fixed-effect model | 0.66 | 0.12 - 3.55 | 0.63 |
| 446 | Polyarteritis nodosa and allied conditions | circulatory system | 7.33 | 0.83 | Fixed-effect model | 0.47 | 0.12 - 1.85 | 0.28 |
| 447 | Other disorders of arteries and arterioles | circulatory system | 16.54 | 0.17 | Fixed-effect model | 0.54 | 0.18 - 1.60 | 0.26 |
| 447.1 | Stricture of artery | circulatory system | 14.57 | 0.27 | Fixed-effect model | 0.87 | 0.23 - 3.32 | 0.84 |
| 450 | Noninfectious disorders of lymphatic channels | circulatory system | 24.60 | 0.02 | Random effect model | 0.11 | 0.01 - 0.96 | 0.05 |
| 451 | Phlebitis and thrombophlebitis | circulatory system | 16.50 | 0.17 | Fixed-effect model | 1.08 | 0.57 - 2.06 | 0.81 |
| 451.2 | Phlebitis and thrombophlebitis of lower extremities | circulatory system | 17.00 | 0.15 | Fixed-effect model | 1.33 | 0.68 - 2.59 | 0.41 |
| 452 | Other venous embolism and thrombosis | circulatory system | 6.95 | 0.86 | Fixed-effect model | 0.66 | 0.12 - 3.53 | 0.63 |
| 454 | Varicose veins | circulatory system | 6.87 | 0.87 | Fixed-effect model | 1.06 | 0.73 - 1.53 | 0.75 |
| 454.1 | Varicose veins of lower extremity | circulatory system | 5.98 | 0.92 | Fixed-effect model | 1.06 | 0.72 - 1.56 | 0.76 |
| 454.11 | Varicose veins of lower extremity, symptomtic | circulatory system | 25.74 | 0.01 | Random effect model | 1.19 | 0.12 - 11.94 | 0.88 |
| 455 | Hemorrhoids | circulatory system | 3.36 | 0.99 | Fixed-effect model | 1.02 | 0.78 - 1.33 | 0.90 |
| 458 | Hypotension | circulatory system | 13.56 | 0.33 | Fixed-effect model | 0.56 | 0.33 - 0.96 | 0.03 |
| 458.1 | Orthostatic hypotension | circulatory system | 10.16 | 0.60 | Fixed-effect model | 0.18 | 0.06 - 0.55 | 2.33E-03 |
| 458.9 | Hypotension NOS | circulatory system | 13.67 | 0.32 | Fixed-effect model | 0.46 | 0.23 - 0.89 | 0.02 |
| 459 | Other disorders of circulatory system | circulatory system | 14.33 | 0.28 | Fixed-effect model | 0.94 | 0.69 - 1.29 | 0.72 |
| 459.9 | Circulatory disease NEC | circulatory system | 14.44 | 0.27 | Fixed-effect model | 0.96 | 0.70 - 1.32 | 0.81 |
| 465 | Acute upper respiratory infections of multiple or unspecified sites | respiratory | 15.52 | 0.21 | Fixed-effect model | 2.24 | 0.99 - 5.06 | 0.05 |
| 465.2 | Acute pharyngitis | respiratory | 4.00 | 0.98 | Fixed-effect model | 6.20 | 1.62 - 23.69 | 0.01 |
| 470 | Septal Deviations/Turbinate Hypertrophy | respiratory | 6.23 | 0.90 | Fixed-effect model | 0.73 | 0.42 - 1.30 | 0.29 |
| 471 | Nasal polyps | respiratory | 8.19 | 0.77 | Fixed-effect model | 0.91 | 0.45 - 1.82 | 0.78 |
| 472 | Chronic pharyngitis and nasopharyngitis | respiratory | 12.90 | 0.38 | Fixed-effect model | 0.81 | 0.22 - 2.90 | 0.74 |
| 473 | Diseases of the larynx and vocal cords | respiratory | 14.90 | 0.25 | Fixed-effect model | 0.72 | 0.33 - 1.56 | 0.40 |
| 473.4 | Voice disturbance | respiratory | 10.08 | 0.61 | Fixed-effect model | 0.42 | 0.13 - 1.40 | 0.16 |
| 474.1 | Acute tonsillitis | respiratory | 8.89 | 0.71 | Fixed-effect model | 0.63 | 0.13 - 3.12 | 0.57 |
| 474.2 | Chronic tonsillitis and adenoiditis | respiratory | 14.83 | 0.25 | Fixed-effect model | 0.12 | 0.04 - 0.40 | 5.06E-04 |
| 475 | Chronic sinusitis | respiratory | 11.68 | 0.47 | Fixed-effect model | 1.84 | 0.84 - 4.02 | 0.13 |
| 476 | Allergic rhinitis | respiratory | 13.60 | 0.33 | Fixed-effect model | 1.50 | 0.45 - 5.01 | 0.51 |
| 477 | Epistaxis or throat hemorrhage | respiratory | 4.88 | 0.96 | Fixed-effect model | 0.39 | 0.17 - 0.87 | 0.02 |
| 479 | Other upper respiratory disease | respiratory | 10.42 | 0.58 | Fixed-effect model | 0.60 | 0.32 - 1.11 | 0.11 |
| 480 | Pneumonia | respiratory | 10.22 | 0.60 | Fixed-effect model | 0.68 | 0.46 - 1.02 | 0.06 |
| 480.1 | Bacterial pneumonia | respiratory | 8.14 | 0.77 | Fixed-effect model | 0.58 | 0.35 - 0.96 | 0.03 |
| 480.11 | Pneumococcal pneumonia | respiratory | 10.43 | 0.58 | Fixed-effect model | 0.57 | 0.33 - 0.96 | 0.03 |
| 495 | Asthma | respiratory | 14.19 | 0.29 | Fixed-effect model | 0.69 | 0.53 - 0.90 | 0.01 |
| 496 | Chronic airway obstruction | respiratory | 7.46 | 0.83 | Fixed-effect model | 1.23 | 0.83 - 1.83 | 0.30 |
| 496.1 | Emphysema | respiratory | 4.89 | 0.96 | Fixed-effect model | 0.37 | 0.14 - 0.96 | 0.04 |
| 496.2 | Chronic bronchitis | respiratory | 7.10 | 0.85 | Fixed-effect model | 1.65 | 0.79 - 3.45 | 0.18 |
| 496.21 | Obstructive chronic bronchitis | respiratory | 7.20 | 0.84 | Fixed-effect model | 2.10 | 0.96 - 4.58 | 0.06 |
| 497 | Bronchitis | respiratory | 14.97 | 0.24 | Fixed-effect model | 1.53 | 0.32 - 7.37 | 0.60 |
| 501 | Pneumonitis due to inhalation of food or vomitus | respiratory | 4.91 | 0.96 | Fixed-effect model | 1.12 | 0.22 - 5.81 | 0.89 |
| 502 | Postinflammatory pulmonary fibrosis | respiratory | 13.57 | 0.33 | Fixed-effect model | 1.02 | 0.27 - 3.89 | 0.97 |
| 506 | Empyema and pneumothorax | respiratory | 7.06 | 0.85 | Fixed-effect model | 1.44 | 0.45 - 4.54 | 0.54 |
| 507 | Pleurisy; pleural effusion | respiratory | 6.72 | 0.88 | Fixed-effect model | 0.82 | 0.50 - 1.35 | 0.44 |
| 509 | Respiratory failure, insufficiency, arrest | respiratory | 7.35 | 0.83 | Fixed-effect model | 0.26 | 0.12 - 0.58 | 8.98E-04 |
| 509.2 | Respiratory insufficiency | respiratory | 5.12 | 0.95 | Fixed-effect model | 0.44 | 0.17 - 1.14 | 0.09 |
| 510 | Other diseases of lung | respiratory | 16.48 | 0.17 | Fixed-effect model | 0.11 | 0.03 - 0.48 | 2.84E-03 |
| 512 | Other symptoms of respiratory system | respiratory | 13.80 | 0.31 | Fixed-effect model | 0.68 | 0.45 - 1.04 | 0.07 |
| 512.7 | Shortness of breath | respiratory | 18.68 | 0.10 | Fixed-effect model | 0.47 | 0.28 - 0.80 | 0.01 |
| 512.8 | Cough | respiratory | 3.14 | 0.99 | Fixed-effect model | 1.42 | 0.67 - 3.01 | 0.35 |
| 512.9 | Other dyspnea | respiratory | 5.30 | 0.95 | Fixed-effect model | 0.23 | 0.06 - 0.84 | 0.03 |
| 513 | Respiratory abnormalities | respiratory | 10.46 | 0.58 | Fixed-effect model | 1.32 | 0.27 - 6.51 | 0.74 |
| 514 | Abnormal findings examination of lungs | respiratory | 8.96 | 0.71 | Fixed-effect model | 0.70 | 0.32 - 1.55 | 0.38 |
| 516 | Abnormal sputum | respiratory | 8.00 | 0.79 | Fixed-effect model | 0.69 | 0.30 - 1.62 | 0.40 |
| 519 | Other diseases of respiratory system, not elsewhere classified | respiratory | 3.18 | 0.99 | Fixed-effect model | 0.66 | 0.43 - 0.99 | 0.05 |
| 519.8 | Other diseases of respiratory system, NEC | respiratory | 4.18 | 0.98 | Fixed-effect model | 0.66 | 0.43 - 1.01 | 0.05 |
| 520 | Disorders of tooth development | digestive | 13.23 | 0.35 | Fixed-effect model | 1.28 | 0.57 - 2.86 | 0.55 |
| 520.2 | Disturbances in tooth eruption | digestive | 12.38 | 0.42 | Fixed-effect model | 1.30 | 0.58 - 2.93 | 0.53 |
| 521 | Diseases of hard tissues of teeth | digestive | 10.42 | 0.58 | Fixed-effect model | 0.71 | 0.34 - 1.46 | 0.35 |
| 521.1 | Dental caries | digestive | 10.77 | 0.55 | Fixed-effect model | 0.73 | 0.36 - 1.52 | 0.41 |
| 522 | Diseases of pulp and periapical tissues | digestive | 8.96 | 0.71 | Fixed-effect model | 2.08 | 0.82 - 5.27 | 0.12 |
| 522.5 | Periapical abscess | digestive | 14.70 | 0.26 | Fixed-effect model | 1.95 | 0.62 - 6.17 | 0.25 |
| 523 | Gingival and periodontal diseases | digestive | 19.72 | 0.07 | Fixed-effect model | 1.27 | 0.49 - 3.28 | 0.62 |
| 523.3 | Periodontitis (acute or chronic) | digestive | 19.67 | 0.07 | Fixed-effect model | 1.57 | 0.50 - 4.90 | 0.44 |
| 523.31 | Acute periodontitis | digestive | 11.98 | 0.45 | Fixed-effect model | 1.46 | 0.32 - 6.76 | 0.63 |
| 523.32 | Chronic periodontitis | digestive | 22.22 | 0.04 | Random effect model | 2.61 | 0.27 - 25.14 | 0.41 |
| 525 | Other diseases of the teeth and supporting structures | digestive | 4.16 | 0.98 | Fixed-effect model | 0.71 | 0.33 - 1.51 | 0.37 |
| 526 | Diseases of the jaws | digestive | 9.63 | 0.65 | Fixed-effect model | 2.38 | 0.66 - 8.57 | 0.19 |
| 527 | Diseases of the salivary glands | digestive | 5.22 | 0.95 | Fixed-effect model | 2.02 | 0.46 - 8.87 | 0.35 |
| 528 | Diseases of the oral soft tissues, excluding lesions specific for gingiva and tongue | digestive | 15.59 | 0.21 | Fixed-effect model | 0.67 | 0.35 - 1.27 | 0.22 |
| 528.5 | Diseases of lips | digestive | 6.51 | 0.89 | Fixed-effect model | 2.78 | 0.61 - 12.61 | 0.19 |
| 529 | Diseases and other conditions of the tongue | digestive | 9.47 | 0.66 | Fixed-effect model | 0.70 | 0.23 - 2.17 | 0.54 |
| 530 | Diseases of esophagus | digestive | 9.38 | 0.67 | Fixed-effect model | 1.13 | 0.90 - 1.42 | 0.28 |
| 530.1 | Esophagitis, GERD and related diseases | digestive | 9.65 | 0.65 | Fixed-effect model | 1.34 | 1.06 - 1.70 | 0.02 |
| 530.11 | GERD | digestive | 12.12 | 0.44 | Fixed-effect model | 1.27 | 0.91 - 1.78 | 0.17 |
| 530.12 | Ulcer of esophagus | digestive | 8.58 | 0.74 | Fixed-effect model | 1.44 | 0.82 - 2.51 | 0.20 |
| 530.14 | Reflux esophagitis | digestive | 10.98 | 0.53 | Fixed-effect model | 1.25 | 0.84 - 1.85 | 0.27 |
| 530.2 | Esophageal bleeding (varices/hemorrhage) | digestive | 7.98 | 0.79 | Fixed-effect model | 0.89 | 0.34 - 2.34 | 0.81 |
| 530.5 | Disorders of esophageal motility | digestive | 14.23 | 0.29 | Fixed-effect model | 2.99 | 0.62 - 14.38 | 0.17 |
| 530.9 | Heartburn | digestive | 15.89 | 0.20 | Fixed-effect model | 0.75 | 0.32 - 1.79 | 0.52 |
| 531 | Peptic ulcer (excl. esophageal) | digestive | 11.82 | 0.46 | Fixed-effect model | 1.34 | 0.84 - 2.15 | 0.22 |
| 531.1 | Hemorrhage from gastrointestinal ulcer | digestive | 10.53 | 0.57 | Fixed-effect model | 1.41 | 0.29 - 6.97 | 0.67 |
| 531.2 | Gastric ulcer | digestive | 11.86 | 0.46 | Fixed-effect model | 1.14 | 0.61 - 2.11 | 0.68 |
| 531.3 | Duodenal ulcer | digestive | 17.16 | 0.14 | Fixed-effect model | 1.49 | 0.72 - 3.09 | 0.28 |
| 532 | Dysphagia | digestive | 9.10 | 0.69 | Fixed-effect model | 1.63 | 0.99 - 2.70 | 0.06 |
| 535 | Gastritis and duodenitis | digestive | 8.67 | 0.73 | Fixed-effect model | 1.04 | 0.81 - 1.33 | 0.77 |
| 535.1 | Acute gastritis | digestive | 3.93 | 0.98 | Fixed-effect model | 1.33 | 0.42 - 4.20 | 0.63 |
| 535.6 | Duodenitis | digestive | 11.45 | 0.49 | Fixed-effect model | 1.49 | 0.94 - 2.39 | 0.09 |
| 535.8 | Other specified gastritis | digestive | 8.30 | 0.76 | Fixed-effect model | 0.78 | 0.50 - 1.21 | 0.27 |
| 537 | Other disorders of stomach and duodenum | digestive | 6.53 | 0.89 | Fixed-effect model | 0.83 | 0.41 - 1.66 | 0.60 |
| 540 | Appendiceal conditions | digestive | 8.38 | 0.75 | Fixed-effect model | 0.71 | 0.36 - 1.42 | 0.34 |
| 540.1 | Appendicitis | digestive | 9.59 | 0.65 | Fixed-effect model | 0.65 | 0.32 - 1.31 | 0.23 |
| 540.11 | Acute appendicitis | digestive | 9.83 | 0.63 | Fixed-effect model | 0.64 | 0.29 - 1.41 | 0.27 |
| 550 | Abdominal hernia | digestive | 6.42 | 0.89 | Fixed-effect model | 1.05 | 0.87 - 1.28 | 0.60 |
| 550.1 | Inguinal hernia | digestive | 13.99 | 0.30 | Fixed-effect model | 1.35 | 0.97 - 1.89 | 0.08 |
| 550.2 | Diaphragmatic hernia | digestive | 7.39 | 0.83 | Fixed-effect model | 1.03 | 0.79 - 1.33 | 0.85 |
| 550.3 | Femoral hernia | digestive | 31.61 | 1.59E-03 | Random effect model | 0.38 | 0.03 - 4.73 | 0.45 |
| 550.4 | Umbilical hernia | digestive | 4.83 | 0.96 | Fixed-effect model | 1.45 | 0.74 - 2.81 | 0.28 |
| 550.5 | Ventral hernia | digestive | 10.57 | 0.57 | Fixed-effect model | 0.78 | 0.40 - 1.53 | 0.47 |
| 555 | Inflammatory bowel disease and other gastroenteritis and colitis | digestive | 26.87 | 0.01 | Random effect model | 1.21 | 0.50 - 2.95 | 0.67 |
| 555.1 | Regional enteritis | digestive | 21.92 | 0.04 | Random effect model | 1.94 | 0.54 - 7.04 | 0.31 |
| 555.2 | Ulcerative colitis | digestive | 18.91 | 0.09 | Fixed-effect model | 0.86 | 0.43 - 1.75 | 0.69 |
| 555.21 | Ulcerative colitis (chronic) | digestive | 11.10 | 0.52 | Fixed-effect model | 0.49 | 0.09 - 2.72 | 0.42 |
| 556 | Ulceration of the lower GI tract | digestive | 14.29 | 0.28 | Fixed-effect model | 0.77 | 0.23 - 2.58 | 0.67 |
| 556.1 | Ulceration of intestine | digestive | 7.93 | 0.79 | Fixed-effect model | 0.67 | 0.15 - 3.11 | 0.61 |
| 557 | Intestinal malabsorption (non-celiac) | digestive | 12.70 | 0.39 | Fixed-effect model | 0.74 | 0.31 - 1.76 | 0.49 |
| 558 | Noninfectious gastroenteritis | digestive | 14.05 | 0.30 | Fixed-effect model | 0.58 | 0.42 - 0.81 | 1.32E-03 |
| 559 | Ileostomy status | digestive | 6.07 | 0.91 | Fixed-effect model | 0.84 | 0.31 - 2.23 | 0.72 |
| 560 | Intestinal obstruction without mention of hernia | digestive | 11.46 | 0.49 | Fixed-effect model | 1.09 | 0.59 - 2.04 | 0.78 |
| 560.1 | Paralytic ileus | digestive | 9.55 | 0.66 | Fixed-effect model | 1.19 | 0.21 - 6.78 | 0.84 |
| 560.3 | Peritoneal or intestinal adhesions | digestive | 6.23 | 0.90 | Fixed-effect model | 0.58 | 0.15 - 2.31 | 0.44 |
| 560.4 | Other intestinal obstruction | digestive | 16.01 | 0.19 | Fixed-effect model | 1.13 | 0.57 - 2.26 | 0.72 |
| 561 | Symptoms involving digestive system | digestive | 16.61 | 0.16 | Fixed-effect model | 0.64 | 0.46 - 0.87 | 4.56E-03 |
| 561.2 | Flatulence | digestive | 9.14 | 0.69 | Fixed-effect model | 1.19 | 0.45 - 3.15 | 0.73 |
| 562 | Diverticulosis and diverticulitis | digestive | 11.53 | 0.48 | Fixed-effect model | 0.76 | 0.59 - 0.99 | 0.04 |
| 562.1 | Diverticulosis | digestive | 11.53 | 0.48 | Fixed-effect model | 0.76 | 0.59 - 0.99 | 0.04 |
| 563 | Constipation | digestive | 17.48 | 0.13 | Fixed-effect model | 1.04 | 0.70 - 1.54 | 0.86 |
| 564 | Functional digestive disorders | digestive | 8.64 | 0.73 | Fixed-effect model | 0.79 | 0.60 - 1.04 | 0.10 |
| 564.1 | Irritable Bowel Syndrome | digestive | 12.18 | 0.43 | Fixed-effect model | 0.66 | 0.38 - 1.12 | 0.12 |
| 564.8 | Abnormal findings on exam of gastrointestinal tract/ abdominal area | digestive | 12.95 | 0.37 | Fixed-effect model | 0.88 | 0.33 - 2.34 | 0.80 |
| 564.9 | Personal history of diseases of digestive system | digestive | 7.57 | 0.82 | Fixed-effect model | 0.78 | 0.56 - 1.09 | 0.15 |
| 565 | Anal and rectal conditions | digestive | 15.40 | 0.22 | Fixed-effect model | 0.81 | 0.58 - 1.12 | 0.21 |
| 565.1 | Anal and rectal polyp | digestive | 15.91 | 0.20 | Fixed-effect model | 0.86 | 0.54 - 1.38 | 0.54 |
| 567 | Peritonitis and retroperitoneal infections | digestive | 10.61 | 0.56 | Fixed-effect model | 0.44 | 0.12 - 1.68 | 0.23 |
| 568 | Other disorders of peritoneum | digestive | 8.25 | 0.77 | Fixed-effect model | 0.86 | 0.43 - 1.73 | 0.67 |
| 568.1 | Peritoneal adhesions (postoperative) (postinfection) | digestive | 8.92 | 0.71 | Fixed-effect model | 0.96 | 0.48 - 1.94 | 0.91 |
| 569 | Other disorders of intestine | digestive | 11.40 | 0.49 | Fixed-effect model | 0.45 | 0.24 - 0.83 | 0.01 |
| 571 | Chronic liver disease and cirrhosis | digestive | 8.42 | 0.75 | Fixed-effect model | 0.44 | 0.21 - 0.92 | 0.03 |
| 571.5 | Other chronic nonalcoholic liver disease | digestive | 2.78 | 1.00 | Fixed-effect model | 0.32 | 0.12 - 0.84 | 0.02 |
| 571.8 | Liver abscess and sequelae of chronic liver disease | digestive | 16.66 | 0.16 | Fixed-effect model | 0.41 | 0.11 - 1.50 | 0.18 |
| 571.81 | Portal hypertension | digestive | 22.02 | 0.04 | Random effect model | 0.90 | 0.09 - 9.19 | 0.93 |
| 572 | Ascites (non malignant) | digestive | 18.17 | 0.11 | Fixed-effect model | 2.55 | 0.93 - 6.99 | 0.07 |
| 573 | Other disorders of liver | digestive | 9.55 | 0.66 | Fixed-effect model | 0.95 | 0.56 - 1.62 | 0.86 |
| 573.5 | Jaundice (not of newborn) | digestive | 9.53 | 0.66 | Fixed-effect model | 2.46 | 0.66 - 9.24 | 0.18 |
| 573.7 | Abnormal results of function study of liver | digestive | 7.24 | 0.84 | Fixed-effect model | 0.53 | 0.27 - 1.03 | 0.06 |
| 574 | Cholelithiasis and cholecystitis | digestive | 15.84 | 0.20 | Fixed-effect model | 0.99 | 0.71 - 1.38 | 0.96 |
| 574.1 | Cholelithiasis | digestive | 16.12 | 0.19 | Fixed-effect model | 1.07 | 0.75 - 1.54 | 0.70 |
| 574.11 | Cholelithiasis with acute cholecystitis | digestive | 9.27 | 0.68 | Fixed-effect model | 2.86 | 1.02 - 8.02 | 0.05 |
| 574.12 | Cholelithiasis with other cholecystitis | digestive | 11.39 | 0.50 | Fixed-effect model | 0.89 | 0.52 - 1.53 | 0.68 |
| 574.2 | Calculus of bile duct | digestive | 7.97 | 0.79 | Fixed-effect model | 1.00 | 0.46 - 2.17 | 0.99 |
| 574.3 | Cholecystitis without cholelithiasis | digestive | 16.95 | 0.15 | Fixed-effect model | 0.98 | 0.46 - 2.08 | 0.95 |
| 575 | Other biliary tract disease | digestive | 20.21 | 0.06 | Fixed-effect model | 1.35 | 0.71 - 2.57 | 0.36 |
| 575.2 | Obstruction of bile duct | digestive | 19.17 | 0.08 | Fixed-effect model | 0.41 | 0.10 - 1.73 | 0.23 |
| 575.7 | Other disorders of gallbladder | digestive | 14.73 | 0.26 | Fixed-effect model | 0.83 | 0.29 - 2.41 | 0.73 |
| 575.8 | Other disorders of biliary tract | digestive | 21.48 | 0.04 | Random effect model | 1.44 | 0.28 - 7.47 | 0.67 |
| 577 | Diseases of pancreas | digestive | 6.79 | 0.87 | Fixed-effect model | 0.99 | 0.46 - 2.15 | 0.98 |
| 577.2 | Chronic pancreatitis | digestive | 10.40 | 0.58 | Fixed-effect model | 1.06 | 0.18 - 6.14 | 0.95 |
| 578 | Gastrointestinal hemorrhage | digestive | 15.21 | 0.23 | Fixed-effect model | 0.97 | 0.73 - 1.28 | 0.83 |
| 578.2 | Blood in stool | digestive | 13.83 | 0.31 | Fixed-effect model | 0.92 | 0.42 - 2.01 | 0.84 |
| 578.8 | Hemorrhage of rectum and anus | digestive | 14.29 | 0.28 | Fixed-effect model | 0.90 | 0.64 - 1.26 | 0.54 |
| 578.9 | Hemorrhage of gastrointestinal tract | digestive | 15.72 | 0.20 | Fixed-effect model | 1.02 | 0.58 - 1.77 | 0.95 |
| 579 | Other symptoms involving abdomen and pelvis | digestive | 11.68 | 0.47 | Fixed-effect model | 0.53 | 0.26 - 1.06 | 0.07 |
| 579.8 | Nonspecific abnormal findings in stool contents | digestive | 12.62 | 0.40 | Fixed-effect model | 0.56 | 0.21 - 1.48 | 0.24 |
| 580 | Nephritis; nephrosis; renal sclerosis | genitourinary | 4.95 | 0.96 | Fixed-effect model | 0.33 | 0.12 - 0.91 | 0.03 |
| 580.1 | Glomerulonephritis | genitourinary | 8.36 | 0.76 | Fixed-effect model | 0.62 | 0.18 - 2.14 | 0.45 |
| 580.14 | Chronic glomerulonephritis, NOS | genitourinary | 14.99 | 0.24 | Fixed-effect model | 0.61 | 0.16 - 2.41 | 0.48 |
| 585 | Renal failure | genitourinary | 11.88 | 0.46 | Fixed-effect model | 0.38 | 0.24 - 0.62 | 8.02E-05 |
| 585.1 | Acute renal failure | genitourinary | 13.11 | 0.36 | Fixed-effect model | 0.29 | 0.16 - 0.53 | 4.87E-05 |
| 585.2 | Renal failure NOS | genitourinary | 10.72 | 0.55 | Fixed-effect model | 1.23 | 0.42 - 3.54 | 0.71 |
| 585.3 | Chronic renal failure [CKD] | genitourinary | 4.45 | 0.97 | Fixed-effect model | 0.29 | 0.13 - 0.63 | 1.92E-03 |
| 586 | Other disorders of the kidney and ureters | genitourinary | 9.95 | 0.62 | Fixed-effect model | 1.15 | 0.58 - 2.31 | 0.68 |
| 586.2 | Cyst of kidney, acquired | genitourinary | 8.57 | 0.74 | Fixed-effect model | 0.71 | 0.23 - 2.18 | 0.55 |
| 586.4 | Stricture/obstruction of ureter | genitourinary | 9.02 | 0.70 | Fixed-effect model | 3.66 | 0.98 - 13.60 | 0.05 |
| 590 | Pyelonephritis | genitourinary | 10.41 | 0.58 | Fixed-effect model | 1.09 | 0.37 - 3.23 | 0.88 |
| 591 | Urinary tract infection | genitourinary | 8.27 | 0.76 | Fixed-effect model | 0.79 | 0.55 - 1.13 | 0.19 |
| 592 | Cystitis and urethritis | genitourinary | 26.06 | 0.01 | Random effect model | 1.31 | 0.45 - 3.81 | 0.62 |
| 592.1 | Cystitis | genitourinary | 26.57 | 0.01 | Random effect model | 1.49 | 0.50 - 4.42 | 0.47 |
| 592.12 | Chronic cystitis | genitourinary | 18.88 | 0.09 | Fixed-effect model | 1.13 | 0.30 - 4.22 | 0.86 |
| 593 | Hematuria | genitourinary | 9.20 | 0.69 | Fixed-effect model | 1.09 | 0.80 - 1.49 | 0.60 |
| 594 | Urinary calculus | genitourinary | 8.60 | 0.74 | Fixed-effect model | 0.68 | 0.41 - 1.12 | 0.13 |
| 594.1 | Calculus of kidney | genitourinary | 5.59 | 0.94 | Fixed-effect model | 0.59 | 0.29 - 1.19 | 0.14 |
| 594.2 | Calculus of lower urinary tract | genitourinary | 8.70 | 0.73 | Fixed-effect model | 0.34 | 0.08 - 1.42 | 0.14 |
| 594.3 | Calculus of ureter | genitourinary | 12.62 | 0.40 | Fixed-effect model | 1.17 | 0.52 - 2.63 | 0.70 |
| 596 | Other disorders of bladder | genitourinary | 7.75 | 0.80 | Fixed-effect model | 1.25 | 0.83 - 1.90 | 0.29 |
| 596.5 | Functional disorders of bladder | genitourinary | 13.25 | 0.35 | Fixed-effect model | 0.84 | 0.32 - 2.24 | 0.73 |
| 597 | Other disorders of urethra and urinary tract | genitourinary | 14.14 | 0.29 | Fixed-effect model | 0.92 | 0.49 - 1.71 | 0.79 |
| 597.1 | Urethral stricture (not specified as infectious) | genitourinary | 15.28 | 0.23 | Fixed-effect model | 1.31 | 0.65 - 2.62 | 0.45 |
| 598 | Abnormal findings on examination of urine | genitourinary | 13.05 | 0.37 | Fixed-effect model | 1.19 | 0.61 - 2.32 | 0.61 |
| 599 | Other symptoms/disorders or the urinary system | genitourinary | 10.23 | 0.60 | Fixed-effect model | 1.07 | 0.82 - 1.39 | 0.63 |
| 599.2 | Retention of urine | genitourinary | 10.76 | 0.55 | Fixed-effect model | 0.90 | 0.55 - 1.48 | 0.67 |
| 599.3 | Dysuria | genitourinary | 10.86 | 0.54 | Fixed-effect model | 5.22 | 1.66 - 16.38 | 4.67E-03 |
| 599.4 | Urinary incontinence | genitourinary | 12.05 | 0.44 | Fixed-effect model | 0.89 | 0.59 - 1.37 | 0.60 |
| 599.5 | Frequency of urination and polyuria | genitourinary | 9.20 | 0.69 | Fixed-effect model | 1.96 | 1.05 - 3.65 | 0.03 |
| 611 | Abnormal findings on mammogram or breast exam | genitourinary | 6.83 | 0.87 | Fixed-effect model | 0.96 | 0.35 - 2.62 | 0.94 |
| 611.3 | Lump or mass in breast | genitourinary | 6.64 | 0.88 | Fixed-effect model | 1.04 | 0.38 - 2.85 | 0.94 |
| 612 | Breast conditions, congenital or relating to hormones | genitourinary | 4.32 | 0.98 | Fixed-effect model | 1.40 | 0.38 - 5.09 | 0.61 |
| 612.2 | Hypertrophy of breast (Gynecomastia) | genitourinary | 5.97 | 0.92 | Fixed-effect model | 1.76 | 0.45 - 6.85 | 0.42 |
| 613.1 | Inflammatory disease of breast | genitourinary | 8.60 | 0.74 | Fixed-effect model | 0.58 | 0.13 - 2.53 | 0.47 |
| 613.7 | Other signs and symptoms in breast | genitourinary | 10.90 | 0.54 | Fixed-effect model | 1.18 | 0.27 - 5.20 | 0.83 |
| 619.1 | Noninflammatory disorders of ovary, fallopian tube, and broad ligament | genitourinary | 4.60 | 0.97 | Fixed-effect model | 1.11 | 0.26 - 4.78 | 0.89 |
| 681 | Superficial cellulitis and abscess | dermatologic | 28.34 | 4.93E-03 | Random effect model | 0.66 | 0.32 - 1.37 | 0.27 |
| 681.1 | Cellulitis and abscess of fingers/toes | dermatologic | 17.19 | 0.14 | Fixed-effect model | 5.62 | 1.08 - 29.32 | 0.04 |
| 681.2 | Cellulitis and abscess of face/neck | dermatologic | 20.02 | 0.07 | Fixed-effect model | 0.76 | 0.13 - 4.30 | 0.75 |
| 681.3 | Cellulitis and abscess of arm/hand | dermatologic | 23.81 | 0.02 | Random effect model | 0.57 | 0.27 - 1.21 | 0.14 |
| 681.5 | Cellulitis and abscess of leg, except foot | dermatologic | 22.69 | 0.03 | Random effect model | 0.62 | 0.30 - 1.28 | 0.20 |
| 681.6 | Cellulitis and abscess of foot, toe | dermatologic | 24.47 | 0.02 | Random effect model | 0.58 | 0.27 - 1.24 | 0.16 |
| 681.7 | Cellulitis and abscess of trunk | dermatologic | 12.15 | 0.43 | Fixed-effect model | 0.60 | 0.12 - 2.99 | 0.53 |
| 686 | Other local infections of skin and subcutaneous tissue | dermatologic | 14.38 | 0.28 | Fixed-effect model | 0.80 | 0.44 - 1.43 | 0.45 |
| 686.1 | Carbuncle and furuncle | dermatologic | 17.41 | 0.13 | Fixed-effect model | 1.11 | 0.48 - 2.55 | 0.81 |
| 686.3 | Pilonidal cyst | dermatologic | 18.74 | 0.09 | Fixed-effect model | 0.89 | 0.17 - 4.56 | 0.89 |
| 687 | Symptoms affecting skin | dermatologic | 8.00 | 0.78 | Fixed-effect model | 0.57 | 0.34 - 0.97 | 0.04 |
| 687.1 | Rash and other nonspecific skin eruption | dermatologic | 12.27 | 0.42 | Fixed-effect model | 0.47 | 0.20 - 1.09 | 0.08 |
| 687.4 | Disturbance of skin sensation | dermatologic | 8.15 | 0.77 | Fixed-effect model | 0.74 | 0.36 - 1.53 | 0.41 |
| 689 | Disorder of skin and subcutaneous tissue NOS | dermatologic | 7.99 | 0.79 | Fixed-effect model | 2.01 | 1.19 - 3.40 | 0.01 |
| 694 | Dyschromia and Vitiligo | dermatologic | 14.58 | 0.27 | Fixed-effect model | 5.96 | 1.69 - 21.02 | 0.01 |
| 694.2 | Other dyschromia | dermatologic | 9.26 | 0.68 | Fixed-effect model | 4.85 | 1.17 - 20.09 | 0.03 |
| 695 | Erythematous conditions | dermatologic | 10.48 | 0.57 | Fixed-effect model | 1.95 | 0.87 - 4.37 | 0.11 |
| 695.7 | Prurigo and Lichen | dermatologic | 6.50 | 0.89 | Fixed-effect model | 1.19 | 0.29 - 4.96 | 0.81 |
| 696 | Psoriasis and related disorders | dermatologic | 18.35 | 0.11 | Fixed-effect model | 0.95 | 0.41 - 2.19 | 0.90 |
| 696.4 | Psoriasis | dermatologic | 18.42 | 0.10 | Fixed-effect model | 0.93 | 0.40 - 2.14 | 0.86 |
| 696.41 | Psoriasis vulgaris | dermatologic | 15.22 | 0.23 | Fixed-effect model | 1.43 | 0.54 - 3.78 | 0.47 |
| 696.42 | Psoriatic arthropathy | dermatologic | 11.01 | 0.53 | Fixed-effect model | 0.45 | 0.10 - 1.98 | 0.29 |
| 697 | Sarcoidosis | dermatologic | 5.94 | 0.92 | Fixed-effect model | 0.70 | 0.13 - 3.86 | 0.68 |
| 698 | Pruritus and related conditions | dermatologic | 13.80 | 0.31 | Fixed-effect model | 2.79 | 0.67 - 11.55 | 0.16 |
| 701 | Other hypertrophic and atrophic conditions of skin | dermatologic | 16.74 | 0.16 | Fixed-effect model | 1.02 | 0.57 - 1.82 | 0.96 |
| 701.2 | Scar conditions and fibrosis of skin | dermatologic | 11.52 | 0.48 | Fixed-effect model | 1.93 | 0.86 - 4.34 | 0.11 |
| 702 | Degenerative skin conditions and other dermatoses | dermatologic | 6.17 | 0.91 | Fixed-effect model | 0.57 | 0.33 - 0.97 | 0.04 |
| 702.1 | Actinic keratosis | dermatologic | 10.59 | 0.56 | Fixed-effect model | 0.81 | 0.37 - 1.78 | 0.61 |
| 702.2 | Seborrheic keratosis | dermatologic | 10.20 | 0.60 | Fixed-effect model | 0.46 | 0.22 - 0.95 | 0.03 |
| 703 | Diseases of nail, NOS | dermatologic | 5.06 | 0.96 | Fixed-effect model | 1.71 | 0.56 - 5.21 | 0.34 |
| 703.1 | Ingrowing nail | dermatologic | 5.76 | 0.93 | Fixed-effect model | 2.48 | 0.69 - 8.93 | 0.17 |
| 704 | Diseases of hair and hair follicles | dermatologic | 7.79 | 0.80 | Fixed-effect model | 0.89 | 0.51 - 1.55 | 0.68 |
| 705 | Disorders of sweat glands | dermatologic | 11.98 | 0.45 | Fixed-effect model | 0.89 | 0.22 - 3.58 | 0.87 |
| 705.8 | Hyperhidrosis | dermatologic | 9.70 | 0.64 | Fixed-effect model | 1.68 | 0.33 - 8.49 | 0.53 |
| 706 | Diseases of sebaceous glands | dermatologic | 5.31 | 0.95 | Fixed-effect model | 1.02 | 0.67 - 1.56 | 0.92 |
| 706.2 | Sebaceous cyst | dermatologic | 4.77 | 0.97 | Fixed-effect model | 1.04 | 0.68 - 1.58 | 0.87 |
| 707.1 | Decubitus ulcer | dermatologic | 12.22 | 0.43 | Fixed-effect model | 0.83 | 0.22 - 3.09 | 0.78 |
| 709 | Diffuse diseases of connective tissue | dermatologic | 9.52 | 0.66 | Fixed-effect model | 0.47 | 0.24 - 0.92 | 0.03 |
| 709.2 | Sicca syndrome | dermatologic | 11.60 | 0.48 | Fixed-effect model | 0.22 | 0.04 - 1.27 | 0.09 |
| 709.7 | Unspecified diffuse connective tissue disease | dermatologic | 6.07 | 0.91 | Fixed-effect model | 0.56 | 0.26 - 1.19 | 0.13 |
| 710 | Osteomyelitis, periostitis, and other infections involving bone | musculoskeletal | 9.89 | 0.63 | Fixed-effect model | 0.45 | 0.09 - 2.21 | 0.32 |
| 710.1 | Osteomyelitis | musculoskeletal | 10.14 | 0.60 | Fixed-effect model | 1.01 | 0.19 - 5.36 | 0.99 |
| 714 | Rheumatoid arthritis and other inflammatory polyarthropathies | musculoskeletal | 17.21 | 0.14 | Fixed-effect model | 0.40 | 0.22 - 0.72 | 2.04E-03 |
| 714.1 | Rheumatoid arthritis | musculoskeletal | 17.46 | 0.13 | Fixed-effect model | 0.28 | 0.15 - 0.52 | 4.70E-05 |
| 715 | Other inflammatory spondylopathies | musculoskeletal | 14.98 | 0.24 | Fixed-effect model | 1.53 | 0.58 - 4.07 | 0.39 |
| 715.2 | Ankylosing spondylitis | musculoskeletal | 23.80 | 0.02 | Random effect model | 1.09 | 0.12 - 10.29 | 0.94 |
| 716 | Other arthropathies | musculoskeletal | 9.18 | 0.69 | Fixed-effect model | 0.62 | 0.50 - 0.78 | 2.96E-05 |
| 716.1 | Unspecified polyarthropathy or polyarthritis | musculoskeletal | 14.31 | 0.28 | Fixed-effect model | 0.57 | 0.29 - 1.13 | 0.11 |
| 716.2 | Unspecified monoarthritis | musculoskeletal | 5.46 | 0.94 | Fixed-effect model | 0.57 | 0.41 - 0.79 | 8.29E-04 |
| 716.9 | Arthropathy NOS | musculoskeletal | 9.71 | 0.64 | Fixed-effect model | 0.63 | 0.50 - 0.79 | 6.33E-05 |
| 717 | Polymyalgia Rheumatica | musculoskeletal | 6.35 | 0.90 | Fixed-effect model | 0.41 | 0.13 - 1.34 | 0.14 |
| 720 | Spinal stenosis | musculoskeletal | 14.41 | 0.28 | Fixed-effect model | 0.51 | 0.26 - 1.00 | 0.05 |
| 721 | Spondylosis and allied disorders | musculoskeletal | 9.43 | 0.67 | Fixed-effect model | 1.01 | 0.64 - 1.58 | 0.97 |
| 721.1 | Spondylosis without myelopathy | musculoskeletal | 7.93 | 0.79 | Fixed-effect model | 1.23 | 0.70 - 2.16 | 0.47 |
| 722 | Intervertebral disc disorders | musculoskeletal | 11.23 | 0.51 | Fixed-effect model | 1.23 | 0.81 - 1.88 | 0.33 |
| 722.1 | Displacement of intervertebral disc | musculoskeletal | 13.36 | 0.34 | Fixed-effect model | 0.94 | 0.16 - 5.45 | 0.95 |
| 722.6 | Degeneration of intervertebral disc | musculoskeletal | 20.83 | 0.05 | Fixed-effect model | 1.98 | 0.94 - 4.21 | 0.07 |
| 722.9 | Other and unspecified disc disorder | musculoskeletal | 7.13 | 0.85 | Fixed-effect model | 1.20 | 0.66 - 2.18 | 0.54 |
| 724.9 | Other unspecified back disorders | musculoskeletal | 11.95 | 0.45 | Fixed-effect model | 0.32 | 0.12 - 0.86 | 0.02 |
| 726 | Peripheral enthesopathies and allied syndromes | musculoskeletal | 16.62 | 0.16 | Fixed-effect model | 1.47 | 1.05 - 2.05 | 0.03 |
| 726.1 | Enthesopathy | musculoskeletal | 9.70 | 0.64 | Fixed-effect model | 1.14 | 0.75 - 1.73 | 0.54 |
| 726.3 | Bursitis | musculoskeletal | 9.89 | 0.63 | Fixed-effect model | 0.77 | 0.20 - 2.99 | 0.70 |
| 727 | Other disorders of synovium, tendon, and bursa | musculoskeletal | 6.50 | 0.89 | Fixed-effect model | 0.79 | 0.49 - 1.26 | 0.32 |
| 727.1 | Synovitis and tenosynovitis | musculoskeletal | 5.21 | 0.95 | Fixed-effect model | 0.75 | 0.35 - 1.60 | 0.46 |
| 727.4 | Ganglion and cyst of synovium, tendon, and bursa | musculoskeletal | 9.42 | 0.67 | Fixed-effect model | 0.92 | 0.46 - 1.87 | 0.83 |
| 727.5 | Rupture of synovium | musculoskeletal | 18.62 | 0.10 | Fixed-effect model | 0.66 | 0.13 - 3.34 | 0.62 |
| 728 | Disorders of muscle, ligament, and fascia | musculoskeletal | 12.65 | 0.40 | Fixed-effect model | 0.55 | 0.30 - 1.02 | 0.06 |
| 728.7 | Fasciitis | musculoskeletal | 13.22 | 0.35 | Fixed-effect model | 0.66 | 0.35 - 1.26 | 0.21 |
| 728.71 | Contracture of palmar fascia [Dupuytren's disease] | musculoskeletal | 12.26 | 0.43 | Fixed-effect model | 0.76 | 0.38 - 1.52 | 0.44 |
| 729 | Other disorders of soft tissues | musculoskeletal | 8.78 | 0.72 | Fixed-effect model | 0.94 | 0.56 - 1.56 | 0.80 |
| 729.1 | Rheumatism, unspecified and fibrositis | musculoskeletal | 6.20 | 0.91 | Fixed-effect model | 1.49 | 0.43 - 5.24 | 0.53 |
| 733 | Other disorders of bone and cartilage | musculoskeletal | 6.55 | 0.89 | Fixed-effect model | 0.53 | 0.28 - 1.02 | 0.06 |
| 733.4 | Aseptic necrosis of bone | musculoskeletal | 4.23 | 0.98 | Fixed-effect model | 0.58 | 0.10 - 3.20 | 0.53 |
| 733.8 | Malunion and nonunion of fracture | musculoskeletal | 8.29 | 0.76 | Fixed-effect model | 0.06 | 0.02 - 0.19 | 9.89E-07 |
| 735 | Acquired foot deformities | musculoskeletal | 15.54 | 0.21 | Fixed-effect model | 0.76 | 0.50 - 1.15 | 0.20 |
| 735.2 | Acquired toe deformities | musculoskeletal | 12.71 | 0.39 | Fixed-effect model | 0.48 | 0.27 - 0.84 | 0.01 |
| 735.23 | Hallux rigidus | musculoskeletal | 7.91 | 0.79 | Fixed-effect model | 1.03 | 0.37 - 2.81 | 0.96 |
| 735.3 | Hallux valgus (Bunion) | musculoskeletal | 16.92 | 0.15 | Fixed-effect model | 1.02 | 0.62 - 1.68 | 0.94 |
| 736 | Other acquired deformities of limbs | musculoskeletal | 7.96 | 0.79 | Fixed-effect model | 0.28 | 0.11 - 0.75 | 0.01 |
| 737 | Curvature of spine | musculoskeletal | 7.53 | 0.82 | Fixed-effect model | 0.37 | 0.11 - 1.19 | 0.09 |
| 737.3 | Kyphoscoliosis and scoliosis | musculoskeletal | 8.64 | 0.73 | Fixed-effect model | 0.53 | 0.16 - 1.83 | 0.32 |
| 738.4 | Acquired spondylolisthesis | musculoskeletal | 13.89 | 0.31 | Fixed-effect model | 0.10 | 0.04 - 0.28 | 1.06E-05 |
| 740 | Osteoarthrosis | musculoskeletal | 30.03 | 2.77E-03 | Random effect model | 0.82 | 0.55 - 1.22 | 0.33 |
| 740.1 | Osteoarthritis; localized | musculoskeletal | 30.77 | 2.14E-03 | Random effect model | 0.72 | 0.44 - 1.19 | 0.20 |
| 740.11 | Osteoarthrosis, localized, primary | musculoskeletal | 25.89 | 0.01 | Random effect model | 0.81 | 0.43 - 1.51 | 0.50 |
| 740.2 | Osteoarthrosis, generalized | musculoskeletal | 10.16 | 0.60 | Fixed-effect model | 1.36 | 0.27 - 6.84 | 0.71 |
| 740.9 | Osteoarthrosis NOS | musculoskeletal | 11.74 | 0.47 | Fixed-effect model | 0.81 | 0.56 - 1.17 | 0.26 |
| 741 | Symptoms and disorders of the joints | musculoskeletal | 5.27 | 0.95 | Fixed-effect model | 0.93 | 0.48 - 1.81 | 0.83 |
| 741.2 | Stiffness of joint | musculoskeletal | 10.46 | 0.58 | Fixed-effect model | 27.75 | 5.64 - 136.54 | 4.35E-05 |
| 741.4 | Joint effusions | musculoskeletal | 8.83 | 0.72 | Fixed-effect model | 0.42 | 0.15 - 1.19 | 0.10 |
| 742 | Derangement of joint, nontraumatic | musculoskeletal | 10.60 | 0.56 | Fixed-effect model | 0.58 | 0.28 - 1.20 | 0.14 |
| 742.8 | Articular cartilage disorder | musculoskeletal | 13.52 | 0.33 | Fixed-effect model | 6.97 | 1.19 - 40.73 | 0.03 |
| 743 | Osteoporosis, osteopenia and pathological fracture | musculoskeletal | 11.55 | 0.48 | Fixed-effect model | 0.74 | 0.46 - 1.18 | 0.20 |
| 743.1 | Osteoporosis | musculoskeletal | 13.16 | 0.36 | Fixed-effect model | 1.08 | 0.66 - 1.79 | 0.75 |
| 743.11 | Osteoporosis NOS | musculoskeletal | 13.73 | 0.32 | Fixed-effect model | 1.25 | 0.73 - 2.14 | 0.41 |
| 743.13 | Other specified osteoporosis | musculoskeletal | 5.97 | 0.92 | Fixed-effect model | 0.79 | 0.14 - 4.43 | 0.79 |
| 743.2 | Pathologic fracture | musculoskeletal | 11.65 | 0.47 | Fixed-effect model | 1.24 | 0.22 - 7.06 | 0.81 |
| 743.9 | Osteopenia or other disorder of bone and cartilage | musculoskeletal | 11.46 | 0.49 | Fixed-effect model | 0.03 | 0.01 - 0.14 | 2.34E-06 |
| 745 | Pain in joint | musculoskeletal | 17.23 | 0.14 | Fixed-effect model | 0.76 | 0.48 - 1.18 | 0.22 |
| 747 | Cardiac and circulatory congenital anomalies | congenital anomalies | 2.44 | 1.00 | Fixed-effect model | 2.55 | 1.20 - 5.43 | 0.02 |
| 747.1 | Cardiac congenital anomalies | congenital anomalies | 3.83 | 0.99 | Fixed-effect model | 3.11 | 1.42 - 6.80 | 4.43E-03 |
| 747.11 | Cardiac shunt/ heart septal defect | congenital anomalies | 12.97 | 0.37 | Fixed-effect model | 8.70 | 1.69 - 44.80 | 0.01 |
| 747.13 | Congenital anomalies of great vessels | congenital anomalies | 2.24 | 1.00 | Fixed-effect model | 1.45 | 0.56 - 3.73 | 0.44 |
| 750 | Digestive congenital anomalies | congenital anomalies | 15.65 | 0.21 | Fixed-effect model | 2.40 | 0.54 - 10.57 | 0.25 |
| 751 | Genitourinary congenital anomalies | congenital anomalies | 18.02 | 0.12 | Fixed-effect model | 1.01 | 0.38 - 2.70 | 0.98 |
| 751.1 | Congenital anomalies of genital organs | congenital anomalies | 13.41 | 0.34 | Fixed-effect model | 0.34 | 0.08 - 1.48 | 0.15 |
| 751.2 | Congenital anomalies of urinary system | congenital anomalies | 9.71 | 0.64 | Fixed-effect model | 2.75 | 0.71 - 10.73 | 0.14 |
| 756 | Other congenital musculoskeletal anomalies | congenital anomalies | 8.63 | 0.73 | Fixed-effect model | 2.25 | 0.45 - 11.31 | 0.33 |
| 760 | Back pain | symptoms | 16.08 | 0.19 | Fixed-effect model | 0.93 | 0.63 - 1.37 | 0.71 |
| 761 | Cervicalgia | symptoms | 10.67 | 0.56 | Fixed-effect model | 0.98 | 0.38 - 2.53 | 0.97 |
| 764 | Sciatica | symptoms | 3.27 | 0.99 | Fixed-effect model | 0.59 | 0.26 - 1.34 | 0.21 |
| 766 | Neuralgia, neuritis, and radiculitis NOS | symptoms | 16.16 | 0.18 | Fixed-effect model | 0.66 | 0.21 - 2.07 | 0.47 |
| 770 | Myalgia and myositis unspecified | symptoms | 7.35 | 0.83 | Fixed-effect model | 2.13 | 0.44 - 10.26 | 0.34 |
| 771 | Musculoskeletal symptoms referable to limbs | symptoms | 7.21 | 0.84 | Fixed-effect model | 0.78 | 0.46 - 1.33 | 0.37 |
| 771.1 | Swelling of limb | symptoms | 9.97 | 0.62 | Fixed-effect model | 0.83 | 0.46 - 1.50 | 0.54 |
| 772 | Symptoms of the muscles | symptoms | 19.58 | 0.08 | Fixed-effect model | 0.53 | 0.12 - 2.36 | 0.40 |
| 773 | Pain in limb | symptoms | 15.08 | 0.24 | Fixed-effect model | 0.70 | 0.42 - 1.16 | 0.17 |
| 782 | Symptoms involving skin and other integumentary tissue | symptoms | 8.09 | 0.78 | Fixed-effect model | 0.40 | 0.16 - 1.01 | 0.05 |
| 782.3 | Edema | symptoms | 6.85 | 0.87 | Fixed-effect model | 0.45 | 0.17 - 1.20 | 0.11 |
| 783 | Fever of unknown origin | symptoms | 9.73 | 0.64 | Fixed-effect model | 1.28 | 0.68 - 2.43 | 0.44 |
| 785 | Abdominal pain | symptoms | 11.75 | 0.47 | Fixed-effect model | 0.92 | 0.75 - 1.13 | 0.43 |
| 788 | Syncope and collapse | symptoms | 5.05 | 0.96 | Fixed-effect model | 0.84 | 0.55 - 1.28 | 0.41 |
| 789 | Nausea and vomiting | symptoms | 8.54 | 0.74 | Fixed-effect model | 0.83 | 0.57 - 1.19 | 0.31 |
| 790 | Nonspecific findings on examination of blood | symptoms | 7.41 | 0.83 | Fixed-effect model | 0.60 | 0.36 - 0.99 | 0.05 |
| 790.6 | Other abnormal blood chemistry | symptoms | 6.71 | 0.88 | Fixed-effect model | 0.60 | 0.36 - 0.99 | 0.05 |
| 791 | Gangrene | symptoms | 11.07 | 0.52 | Fixed-effect model | 0.19 | 0.04 - 1.04 | 0.06 |
| 793 | Nonspecific abnormal findings on radiological and other examination of musculoskeletal system | injuries & poisonings | 5.17 | 0.95 | Fixed-effect model | 2.73 | 0.62 - 12.07 | 0.19 |
| 793.2 | Nonspecific abnormal findings on radiological and other examination of other intrathoracic organs (echocardiogram, etc) | circulatory system | 3.28 | 0.99 | Fixed-effect model | 11.28 | 2.14 - 59.38 | 4.23E-03 |
| 798 | Malaise and fatigue | symptoms | 9.97 | 0.62 | Fixed-effect model | 0.84 | 0.43 - 1.65 | 0.62 |
| 798.1 | Chronic fatigue syndrome | symptoms | 8.11 | 0.78 | Fixed-effect model | 3.84 | 0.76 - 19.31 | 0.10 |
| 800 | Fracture of lower limb | injuries & poisonings | 16.71 | 0.16 | Fixed-effect model | 0.66 | 0.41 - 1.06 | 0.09 |
| 800.1 | Fracture of neck of femur | injuries & poisonings | 9.48 | 0.66 | Fixed-effect model | 0.98 | 0.39 - 2.47 | 0.96 |
| 800.3 | Fracture of tibia and fibula | injuries & poisonings | 7.38 | 0.83 | Fixed-effect model | 0.65 | 0.27 - 1.54 | 0.33 |
| 800.4 | Fracture of patella | injuries & poisonings | 16.23 | 0.18 | Fixed-effect model | 0.77 | 0.19 - 3.22 | 0.72 |
| 801 | Fracture of ankle and foot | injuries & poisonings | 8.84 | 0.72 | Fixed-effect model | 1.40 | 0.62 - 3.17 | 0.42 |
| 802 | Fracture of pelvis | injuries & poisonings | 6.64 | 0.88 | Fixed-effect model | 1.20 | 0.27 - 5.26 | 0.81 |
| 803 | Fracture of upper limb | injuries & poisonings | 23.21 | 0.03 | Random effect model | 0.61 | 0.33 - 1.13 | 0.12 |
| 803.1 | Fracture of humerus | injuries & poisonings | 11.59 | 0.48 | Fixed-effect model | 0.31 | 0.12 - 0.80 | 0.02 |
| 803.2 | Fracture of radius and ulna | injuries & poisonings | 30.32 | 2.50E-03 | Random effect model | 0.74 | 0.30 - 1.79 | 0.50 |
| 803.3 | Fracture of clavicle or scapula | injuries & poisonings | 14.41 | 0.28 | Fixed-effect model | 1.22 | 0.44 - 3.34 | 0.70 |
| 804 | Fracture of hand or wrist | injuries & poisonings | 15.69 | 0.21 | Fixed-effect model | 0.54 | 0.27 - 1.07 | 0.08 |
| 805 | Fracture of vertebral column without mention of spinal cord injury | injuries & poisonings | 18.43 | 0.10 | Fixed-effect model | 0.11 | 0.04 - 0.31 | 4.45E-05 |
| 807 | Fracture of ribs | injuries & poisonings | 10.34 | 0.59 | Fixed-effect model | 0.52 | 0.12 - 2.16 | 0.37 |
| 809 | Fracture of unspecified bones | injuries & poisonings | 13.73 | 0.32 | Fixed-effect model | 0.14 | 0.05 - 0.42 | 4.45E-04 |
| 818 | Intracranial hemorrhage (injury) | injuries & poisonings | 3.76 | 0.99 | Fixed-effect model | 2.26 | 0.39 - 13.18 | 0.36 |
| 819 | Skull and face fracture and other intercranial injury | injuries & poisonings | 2.45 | 1.00 | Fixed-effect model | 1.18 | 0.57 - 2.44 | 0.66 |
| 823 | Torus fracture | injuries & poisonings | 7.17 | 0.85 | Fixed-effect model | 0.63 | 0.24 - 1.67 | 0.35 |
| 835 | Internal derangement of knee | injuries & poisonings | 16.51 | 0.17 | Fixed-effect model | 0.46 | 0.33 - 0.64 | 4.84E-06 |
| 836 | Traumatic arthropathy | injuries & poisonings | 20.87 | 0.05 | Fixed-effect model | 0.10 | 0.02 - 0.57 | 0.01 |
| 850 | Hemorrhage or hematoma complicating a procedure | injuries & poisonings | 15.08 | 0.24 | Fixed-effect model | 0.63 | 0.36 - 1.09 | 0.10 |
| 853 | Complication of colostomy or enterostomy | injuries & poisonings | 11.28 | 0.50 | Fixed-effect model | 1.16 | 0.20 - 6.78 | 0.87 |
| 854 | Complications of cardiac/vascular device, implant, and graft | injuries & poisonings | 17.56 | 0.13 | Fixed-effect model | 0.82 | 0.32 - 2.11 | 0.68 |
| 857 | Mechanical complication of unspecified genitourinary device, implant, and graft | injuries & poisonings | 12.23 | 0.43 | Fixed-effect model | 2.39 | 0.78 - 7.36 | 0.13 |
| 858 | Complication of internal orthopedic device | injuries & poisonings | 11.54 | 0.48 | Fixed-effect model | 0.89 | 0.44 - 1.79 | 0.73 |
| 859 | Complication due to other implant and internal device | injuries & poisonings | 9.31 | 0.68 | Fixed-effect model | 0.75 | 0.37 - 1.50 | 0.42 |
| 870 | Open wounds of head; neck; and trunk | injuries & poisonings | 13.66 | 0.32 | Fixed-effect model | 1.85 | 0.97 - 3.53 | 0.06 |
| 870.3 | Other open wound of head and face | injuries & poisonings | 14.35 | 0.28 | Fixed-effect model | 1.44 | 0.69 - 2.98 | 0.33 |
| 871 | Open wounds of extremities | injuries & poisonings | 14.01 | 0.30 | Fixed-effect model | 0.78 | 0.42 - 1.43 | 0.42 |
| 907 | Injuries to the nervous system | injuries & poisonings | 10.84 | 0.54 | Fixed-effect model | 0.50 | 0.17 - 1.48 | 0.21 |
| 915 | Superficial injury without mention of infection | injuries & poisonings | 15.35 | 0.22 | Fixed-effect model | 1.06 | 0.57 - 1.94 | 0.86 |
| 916 | Contusion | injuries & poisonings | 8.32 | 0.76 | Fixed-effect model | 0.96 | 0.34 - 2.69 | 0.93 |
| 939 | Atopic/contact dermatitis due to other or unspecified | dermatologic | 7.64 | 0.81 | Fixed-effect model | 0.64 | 0.27 - 1.52 | 0.31 |
| 946 | Anaphylactic shock NOS | injuries & poisonings | 7.77 | 0.80 | Fixed-effect model | 0.10 | 0.02 - 0.55 | 0.01 |
| 960 | Poisoning by antibiotics | injuries & poisonings | 7.64 | 0.81 | Fixed-effect model | 0.54 | 0.40 - 0.73 | 7.62E-05 |
| 960.2 | Allergy/adverse effect of penicillin | injuries & poisonings | 12.34 | 0.42 | Fixed-effect model | 0.52 | 0.38 - 0.73 | 1.29E-04 |
| 961 | Poisoning by other antiinfectives | injuries & poisonings | 4.89 | 0.96 | Fixed-effect model | 0.39 | 0.13 - 1.21 | 0.10 |
| 961.1 | Poisoning/allergy of sulfonamides | injuries & poisonings | 4.72 | 0.97 | Fixed-effect model | 0.39 | 0.10 - 1.47 | 0.16 |
| 965 | Poisoning by analgesics, antipyretics, and antirheumatics | injuries & poisonings | 12.67 | 0.39 | Fixed-effect model | 1.15 | 0.71 - 1.86 | 0.56 |
| 965.1 | Opiates and related narcotics causing adverse effects in therapeutic use | injuries & poisonings | 8.54 | 0.74 | Fixed-effect model | 0.80 | 0.25 - 2.60 | 0.72 |
| 966 | Poisoning by anticonvulsants and anti-Parkinsonism drugs | injuries & poisonings | 16.47 | 0.17 | Fixed-effect model | 0.82 | 0.14 - 4.78 | 0.83 |
| 967 | Adverse effects of sedatives or other central nervous system depressants and anesthetics | injuries & poisonings | 20.36 | 0.06 | Fixed-effect model | 0.39 | 0.08 - 2.03 | 0.27 |
| 979 | Adverse drug events and drug allergies | injuries & poisonings | 23.04 | 0.03 | Random effect model | 10.12 | 1.26 - 81.33 | 0.03 |
| 990 | Effects radiation NOS | injuries & poisonings | 13.55 | 0.33 | Fixed-effect model | 0.90 | 0.45 - 1.82 | 0.77 |
| 994 | Sepsis and SIRS | infectious diseases | 5.62 | 0.93 | Fixed-effect model | 0.64 | 0.30 - 1.37 | 0.25 |
| 994.2 | Sepsis | infectious diseases | 5.62 | 0.93 | Fixed-effect model | 0.64 | 0.30 - 1.37 | 0.25 |
| 1000 | Burns | injuries & poisonings | 6.61 | 0.88 | Fixed-effect model | 1.73 | 0.34 - 8.97 | 0.51 |
| 1001 | Foreign body injury | injuries & poisonings | 19.42 | 0.08 | Fixed-effect model | 1.88 | 0.60 - 5.89 | 0.28 |
| 1002 | Symptoms concerning nutrition, metabolism, and development | symptoms | 11.11 | 0.52 | Fixed-effect model | 0.91 | 0.53 - 1.55 | 0.72 |
| 1005 | Other symptoms | symptoms | 10.86 | 0.54 | Fixed-effect model | 1.38 | 0.44 - 4.32 | 0.58 |
| 1008 | Crushing or internal injury to organs | injuries & poisonings | 9.65 | 0.65 | Fixed-effect model | 0.99 | 0.28 - 3.47 | 0.99 |
| 1009 | Injury, NOS | injuries & poisonings | 20.73 | 0.05 | Fixed-effect model | 0.79 | 0.50 - 1.22 | 0.29 |
| 1010 | Other tests | symptoms | 11.82 | 0.46 | Fixed-effect model | 0.65 | 0.38 - 1.10 | 0.11 |
| 1011 | Complications of surgical and medical procedures | injuries & poisonings | 19.04 | 0.09 | Fixed-effect model | 0.83 | 0.54 - 1.25 | 0.37 |
| 1015 | Effects of other external causes | symptoms | 14.12 | 0.29 | Fixed-effect model | 0.71 | 0.38 - 1.31 | 0.27 |
| 1019 | Other ill-defined and unknown causes of morbidity and mortality | symptoms | 10.54 | 0.57 | Fixed-effect model | 0.82 | 0.59 - 1.14 | 0.23 |

Odds ratios (ORs) with their 95% confidence intervals (CIs) represent the effect estimates on the risk of multiple non-delirium of per 10% reduction in risk for delirium by targeting O-methylascorbate.

Significant threshold was set at *P*<2.46 x 10^-5^ (Bonferroni-corrected significance threshold calculated as 0.05 divided by 2034 [3 metabolites×678 diseases]). E signifies the exponent of 10 in the table.

Abbreviations: Phe-MR, phenome-wide Mendelian randomization.

**Table S10. The MR-Egger regression for directional pleiotropy of** **clinical LDL cholesterol, sphingomyelin, and O-methylascorbate with the risk of multiple non-delirium’s diseases in Phe-MR analyses.**

| Phe-Code | Phenotype Description | Disease Chapter | Clinical LDL cholesterol | | Sphingomyelin | | O-methylascorbate | |
| --- | --- | --- | --- | --- | --- | --- | --- | --- |
|  |  |  | **MR-Egger Intercept** | | **MR-Egger Intercept** | | **MR-Egger Intercept** | |
|  |  |  | **Estimate /Standard error** | ***P* value** | **Estimate /Standard error** | ***P* value** | **Estimate /Standard error** | **P value** |
| 8 | Intestinal infection | infectious diseases | 5.27E-04/2.77E-03 | 0.85 | -4.70E-03/2.80E-03 | 0.10 | 1.50E-02/1.04E-02 | 0.18 |
| 8.5 | Bacterial enteritis | infectious diseases | -4.76E-03/5.13E-03 | 0.35 | -1.00E-02/5.10E-03 | 0.05 | 2.10E-02/1.89E-02 | 0.28 |
| 8.52 | Intestinal infection due to C. difficile | infectious diseases | -2.52E-04/1.05E-02 | 0.98 | 4.80E-04/1.08E-02 | 0.96 | -4.80E-03/3.88E-02 | 0.90 |
| 8.6 | Viral Enteritis | infectious diseases | -1.49E-03/9.58E-03 | 0.88 | -1.90E-02/9.20E-03 | 0.04 | 6.20E-02/4.48E-02 | 0.20 |
| 38 | Septicemia | infectious diseases | -2.86E-03/4.13E-03 | 0.49 | 2.20E-04/4.20E-03 | 0.96 | -2.10E-02/1.55E-02 | 0.20 |
| 38.1 | Gram negative septicemia | infectious diseases | -2.13E-02/9.24E-03 | 0.02 | -2.60E-03/9.40E-03 | 0.78 | -9.00E-03/3.42E-02 | 0.80 |
| 41 | Bacterial infection NOS | infectious diseases | -1.76E-03/2.51E-03 | 0.49 | -3.00E-03/2.40E-03 | 0.23 | -7.40E-03/9.30E-03 | 0.44 |
| 41.1 | Staphylococcus infections | infectious diseases | -1.49E-03/5.41E-03 | 0.78 | -5.20E-03/4.90E-03 | 0.29 | -1.50E-02/1.75E-02 | 0.40 |
| 41.2 | Streptococcus infection | infectious diseases | 1.06E-03/6.43E-03 | 0.87 | 4.00E-03/6.60E-03 | 0.54 | 2.50E-02/2.44E-02 | 0.33 |
| 41.4 | E. coli | infectious diseases | -9.72E-03/5.17E-03 | 0.06 | -8.60E-03/5.20E-03 | 0.10 | -3.50E-02/1.89E-02 | 0.09 |
| 70 | Viral hepatitis | infectious diseases | 3.10E-04/8.30E-03 | 0.97 | -8.70E-03/7.70E-03 | 0.26 | 1.20E-02/2.85E-02 | 0.69 |
| 78 | Viral warts & HPV | infectious diseases | -6.36E-03/8.24E-03 | 0.44 | -1.10E-02/8.50E-03 | 0.21 | 5.70E-03/3.11E-02 | 0.86 |
| 79 | Viral infection | infectious diseases | -1.79E-03/4.93E-03 | 0.72 | -1.20E-02/5.10E-03 | 0.02 | 3.50E-02/2.26E-02 | 0.15 |
| 80 | Postoperative infection | infectious diseases | -3.61E-03/4.48E-03 | 0.42 | -5.70E-03/4.40E-03 | 0.19 | -2.30E-02/2.04E-02 | 0.29 |
| 81 | Infection/inflammation of internal prosthetic device; implant; and graft | infectious diseases | 3.04E-03/5.25E-03 | 0.56 | 5.70E-03/5.40E-03 | 0.29 | -9.80E-03/1.99E-02 | 0.63 |
| 112 | Candidiasis | infectious diseases | 1.88E-02/5.67E-03 | 1.22E-03 | 8.00E-03/5.90E-03 | 0.17 | -1.30E-02/2.14E-02 | 0.56 |
| 145 | Cancer of mouth | neoplasms | 2.83E-02/1.08E-02 | 0.01 | 7.60E-03/1.07E-02 | 0.48 | 5.20E-02/4.39E-02 | 0.26 |
| 149 | Cancer of larynx, pharynx, nasal cavities | neoplasms | 1.83E-02/1.11E-02 | 0.10 | -7.70E-03/1.07E-02 | 0.47 | 3.60E-02/3.96E-02 | 0.39 |
| 150 | Cancer of esophagus | neoplasms | 7.21E-03/1.00E-02 | 0.47 | -7.20E-03/1.11E-02 | 0.52 | -5.60E-02/3.69E-02 | 0.16 |
| 151 | Cancer of stomach | neoplasms | -2.35E-03/1.11E-02 | 0.83 | 7.70E-03/1.16E-02 | 0.51 | -8.90E-02/4.19E-02 | 0.06 |
| 153 | Colorectal cancer | neoplasms | 3.22E-03/3.94E-03 | 0.42 | -2.30E-03/4.40E-03 | 0.60 | 1.00E-02/1.57E-02 | 0.53 |
| 153.2 | Colon cancer | neoplasms | 5.96E-03/5.16E-03 | 0.25 | -3.00E-03/5.20E-03 | 0.57 | 2.00E-02/1.80E-02 | 0.30 |
| 153.3 | Malignant neoplasm of rectum, rectosigmoid junction, and anus | neoplasms | -3.51E-03/5.73E-03 | 0.54 | -3.90E-03/6.00E-03 | 0.52 | -6.10E-03/2.17E-02 | 0.78 |
| 157 | Pancreatic cancer | neoplasms | 9.42E-03/1.07E-02 | 0.38 | 1.40E-02/1.11E-02 | 0.22 | -3.70E-02/4.05E-02 | 0.39 |
| 158 | Neoplasm of unspecified nature of digestive system | neoplasms | 1.31E-02/8.17E-03 | 0.11 | 7.50E-03/8.40E-03 | 0.38 | 9.60E-04/3.59E-02 | 0.98 |
| 159 | Malignant neoplasm of other and ill-defined sites within the digestive organs and peritoneum | neoplasms | -2.82E-04/3.76E-03 | 0.94 | -4.70E-03/4.20E-03 | 0.27 | 1.30E-02/1.35E-02 | 0.34 |
| 165 | Cancer within the respiratory system | neoplasms | 6.03E-04/5.71E-03 | 0.92 | -3.50E-03/5.50E-03 | 0.53 | -4.90E-04/1.92E-02 | 0.98 |
| 165.1 | Cancer of bronchus; lung | neoplasms | -3.69E-03/6.31E-03 | 0.56 | -1.90E-03/6.40E-03 | 0.77 | -3.10E-03/2.16E-02 | 0.89 |
| 172 | Skin cancer | neoplasms | 2.57E-03/3.16E-03 | 0.42 | 4.80E-04/3.10E-03 | 0.88 | -1.30E-02/8.70E-03 | 0.16 |
| 172.1 | Melanomas of skin, dx or hx | neoplasms | 5.60E-05/5.70E-03 | 0.99 | -1.30E-03/5.50E-03 | 0.81 | 5.00E-03/1.92E-02 | 0.80 |
| 172.11 | Melanomas of skin | neoplasms | 5.60E-05/5.70E-03 | 0.99 | -1.30E-03/5.50E-03 | 0.81 | 5.00E-03/1.92E-02 | 0.80 |
| 172.2 | Other non-epithelial cancer of skin | neoplasms | 4.59E-03/3.31E-03 | 0.17 | 2.40E-03/3.30E-03 | 0.46 | -1.40E-02/9.70E-03 | 0.17 |
| 172.3 | Carcinoma in situ of skin | neoplasms | -7.63E-03/1.02E-02 | 0.45 | -2.20E-02/1.06E-02 | 0.04 | -2.80E-03/4.70E-02 | 0.95 |
| 174 | Breast cancer | neoplasms | 2.55E-04/2.66E-03 | 0.92 | 1.60E-03/3.00E-03 | 0.60 | -4.70E-03/9.00E-03 | 0.61 |
| 189 | Cancer of urinary organs (incl. kidney and bladder) | neoplasms | -1.21E-04/4.09E-03 | 0.98 | -1.40E-02/4.40E-03 | 1.80E-03 | 1.50E-02/1.55E-02 | 0.37 |
| 189.1 | Cancer of kidney and renal pelvis | neoplasms | 2.31E-03/8.73E-03 | 0.79 | -1.90E-02/8.50E-03 | 0.03 | 3.30E-03/3.06E-02 | 0.92 |
| 189.11 | Malignant neoplasm of kidney, except pelvis | neoplasms | 1.84E-03/8.81E-03 | 0.83 | -2.10E-02/8.70E-03 | 0.02 | 1.50E-03/3.12E-02 | 0.96 |
| 189.2 | Cancer of bladder | neoplasms | -8.47E-04/5.36E-03 | 0.87 | -1.50E-02/5.50E-03 | 0.01 | 5.70E-03/2.02E-02 | 0.78 |
| 189.21 | Malignant neoplasm of bladder | neoplasms | 1.90E-03/5.70E-03 | 0.74 | -1.40E-02/5.90E-03 | 0.02 | 1.40E-02/2.15E-02 | 0.53 |
| 191 | Manlignant and unknown neoplasms of brain and nervous system | neoplasms | -1.02E-02/1.03E-02 | 0.32 | -1.00E-02/1.05E-02 | 0.32 | -3.00E-02/3.95E-02 | 0.46 |
| 191.1 | Cancer of brain and nervous system | neoplasms | -1.73E-02/1.13E-02 | 0.13 | -1.20E-02/1.17E-02 | 0.29 | -3.50E-02/5.10E-02 | 0.50 |
| 195 | Cancer, suspected or other | neoplasms | 2.45E-03/2.07E-03 | 0.24 | -5.10E-03/2.40E-03 | 0.03 | 8.90E-03/7.90E-03 | 0.28 |
| 195.1 | Malignant neoplasm, other | neoplasms | 2.05E-03/2.09E-03 | 0.33 | -5.40E-03/2.30E-03 | 0.02 | 8.10E-03/8.10E-03 | 0.34 |
| 197 | Chemotherapy | neoplasms | 3.50E-03/1.80E-03 | 0.05 | 9.30E-04/1.90E-03 | 0.62 | -3.70E-03/7.30E-03 | 0.62 |
| 198 | Secondary malignant neoplasm | neoplasms | 3.80E-03/2.74E-03 | 0.17 | 6.80E-04/3.00E-03 | 0.82 | -1.30E-03/1.04E-02 | 0.90 |
| 198.1 | Secondary malignancy of lymph nodes | neoplasms | 4.17E-03/3.76E-03 | 0.27 | 3.50E-03/4.30E-03 | 0.42 | -3.40E-03/1.59E-02 | 0.84 |
| 198.2 | Secondary malignancy of respiratory organs | neoplasms | -2.47E-03/5.58E-03 | 0.66 | 7.00E-05/5.90E-03 | 0.99 | 1.70E-02/2.12E-02 | 0.45 |
| 198.3 | Secondary malignant neoplasm of digestive systems | neoplasms | -5.12E-03/6.67E-03 | 0.44 | 1.80E-03/6.90E-03 | 0.79 | 3.50E-02/2.83E-02 | 0.25 |
| 198.4 | Secondary malignant neoplasm of liver | neoplasms | 1.72E-03/5.12E-03 | 0.74 | -3.90E-03/5.30E-03 | 0.46 | 5.40E-03/1.94E-02 | 0.79 |
| 198.5 | Secondary malignancy of brain/spine | neoplasms | -7.48E-03/9.51E-03 | 0.43 | 3.70E-03/9.90E-03 | 0.71 | 4.50E-02/3.49E-02 | 0.22 |
| 198.6 | Secondary malignancy of bone | neoplasms | 2.55E-03/5.64E-03 | 0.65 | -1.60E-04/6.20E-03 | 0.98 | -7.10E-03/3.01E-02 | 0.82 |
| 199 | Neoplasm of uncertain behavior | neoplasms | 2.64E-03/6.96E-03 | 0.70 | -5.60E-03/7.20E-03 | 0.44 | -3.10E-02/2.89E-02 | 0.31 |
| 200 | Myeloproliferative disease | neoplasms | 9.85E-04/1.03E-02 | 0.92 | -2.90E-03/1.06E-02 | 0.79 | -2.30E-02/4.42E-02 | 0.62 |
| 202 | Cancer of other lymphoid, histiocytic tissue | neoplasms | -1.13E-03/6.30E-03 | 0.86 | 3.10E-03/5.70E-03 | 0.59 | 2.40E-02/2.17E-02 | 0.30 |
| 202.2 | Non-Hodgkins lymphoma | neoplasms | 1.46E-03/6.99E-03 | 0.84 | 2.00E-03/6.90E-03 | 0.78 | 1.70E-02/2.34E-02 | 0.49 |
| 202.24 | Large cell lymphoma | neoplasms | -1.41E-02/1.12E-02 | 0.21 | -7.20E-03/1.12E-02 | 0.52 | 7.30E-03/4.12E-02 | 0.86 |
| 204 | Leukemia | neoplasms | 3.53E-03/6.43E-03 | 0.58 | 3.60E-03/7.00E-03 | 0.61 | 8.90E-03/2.92E-02 | 0.77 |
| 204.1 | Lymphoid leukemia | neoplasms | 3.48E-03/1.12E-02 | 0.76 | -1.00E-02/1.20E-02 | 0.40 | -3.00E-02/5.29E-02 | 0.58 |
| 204.12 | Lymphoid leukemia, chronic | neoplasms | 3.74E-03/1.20E-02 | 0.76 | -1.10E-02/1.27E-02 | 0.41 | -5.60E-02/5.52E-02 | 0.33 |
| 204.4 | Multiple myeloma | neoplasms | 9.13E-03/1.17E-02 | 0.44 | -1.10E-03/1.14E-02 | 0.93 | 3.40E-02/4.19E-02 | 0.43 |
| 208 | Benign neoplasm of colon | neoplasms | 6.16E-03/2.65E-03 | 0.02 | -2.20E-03/2.80E-03 | 0.43 | 6.80E-03/8.50E-03 | 0.44 |
| 210 | Benign neoplasm of lip, oral cavity, and pharynx | neoplasms | 1.89E-02/8.31E-03 | 0.02 | 5.80E-03/8.60E-03 | 0.50 | -5.50E-02/3.14E-02 | 0.11 |
| 211 | Benign neoplasm of other parts of digestive system | neoplasms | -6.11E-04/3.98E-03 | 0.88 | -9.60E-03/3.80E-03 | 0.01 | 1.40E-02/1.76E-02 | 0.44 |
| 214 | Lipoma | neoplasms | -2.91E-03/3.55E-03 | 0.41 | -1.90E-03/3.60E-03 | 0.60 | -1.30E-02/1.24E-02 | 0.30 |
| 214.1 | Lipoma of skin and subcutaneous tissue | neoplasms | 3.32E-04/4.26E-03 | 0.94 | 3.50E-03/4.30E-03 | 0.42 | -2.10E-02/1.46E-02 | 0.19 |
| 215 | Other benign neoplasm of connective and other soft tissue | neoplasms | 1.97E-03/8.20E-03 | 0.81 | 6.00E-03/8.30E-03 | 0.47 | 2.00E-02/2.97E-02 | 0.52 |
| 216 | Benign neoplasm of skin | neoplasms | 1.05E-03/3.03E-03 | 0.73 | -1.60E-03/3.10E-03 | 0.60 | 5.30E-03/1.21E-02 | 0.67 |
| 217 | Vascular hamartomas and non-neoplastic nevi | neoplasms | -3.36E-03/1.03E-02 | 0.74 | 1.50E-02/1.12E-02 | 0.19 | -1.80E-03/3.97E-02 | 0.96 |
| 217.1 | Nevus, non-neoplastic | neoplasms | 2.39E-03/1.07E-02 | 0.82 | 1.50E-02/1.18E-02 | 0.19 | -3.80E-03/4.33E-02 | 0.93 |
| 225 | Benign neoplasm of brain and other parts of nervous system | neoplasms | 9.59E-03/9.09E-03 | 0.29 | 9.00E-04/1.03E-02 | 0.93 | 8.40E-04/3.42E-02 | 0.98 |
| 225.1 | Benign neoplasm of brain, cranial nerves, meninges | neoplasms | 1.11E-02/9.37E-03 | 0.24 | -1.10E-03/1.08E-02 | 0.92 | 1.80E-02/3.56E-02 | 0.62 |
| 227 | Benign neoplasm of other endocrine glands and related structures | neoplasms | -7.20E-03/8.85E-03 | 0.42 | -9.70E-04/9.10E-03 | 0.92 | 5.40E-03/3.63E-02 | 0.88 |
| 228 | Hemangioma and lymphangioma, any site | neoplasms | -2.71E-04/7.01E-03 | 0.97 | -1.40E-02/7.00E-03 | 0.06 | 1.10E-02/2.45E-02 | 0.66 |
| 229 | Benign neoplasm of unspecified sites | neoplasms | 1.55E-03/5.33E-03 | 0.77 | -8.70E-03/5.50E-03 | 0.11 | -2.80E-02/2.00E-02 | 0.20 |
| 240 | Simple and unspecified goiter | endocrine/metabolic | 1.40E-02/1.06E-02 | 0.19 | 2.90E-03/1.10E-02 | 0.80 | 3.10E-02/4.02E-02 | 0.46 |
| 241 | Nontoxic nodular goiter | endocrine/metabolic | 3.87E-04/7.71E-03 | 0.96 | 7.90E-03/8.70E-03 | 0.37 | -1.60E-02/2.93E-02 | 0.59 |
| 241.2 | Nontoxic multinodular goiter | endocrine/metabolic | 6.45E-03/1.01E-02 | 0.52 | 2.10E-02/1.09E-02 | 0.05 | -8.70E-03/3.80E-02 | 0.82 |
| 244 | Hypothyroidism | endocrine/metabolic | -1.29E-03/3.76E-03 | 0.73 | -7.30E-03/4.70E-03 | 0.12 | 1.20E-02/8.50E-03 | 0.18 |
| 244.1 | Secondary hypothyroidism | endocrine/metabolic | 1.83E-02/7.81E-03 | 0.02 | 5.20E-03/8.80E-03 | 0.55 | -5.00E-03/2.97E-02 | 0.87 |
| 244.4 | Hypothyroidism NOS | endocrine/metabolic | -2.00E-03/3.89E-03 | 0.61 | -7.10E-03/4.70E-03 | 0.13 | 1.40E-02/8.90E-03 | 0.15 |
| 250 | Diabetes mellitus | endocrine/metabolic | -9.79E-04/2.72E-03 | 0.72 | -1.30E-02/3.30E-03 | 8.70E-05 | 1.50E-03/1.02E-02 | 0.88 |
| 250.1 | Type 1 diabetes | endocrine/metabolic | -8.55E-03/6.32E-03 | 0.18 | -1.20E-02/5.40E-03 | 0.03 | -2.30E-03/2.28E-02 | 0.92 |
| 250.2 | Type 2 diabetes | endocrine/metabolic | -1.18E-03/2.79E-03 | 0.67 | -1.40E-02/3.30E-03 | 6.10E-05 | 5.00E-03/9.70E-03 | 0.62 |
| 250.23 | Type 2 diabetes with ophthalmic manifestations | endocrine/metabolic | -7.68E-03/7.35E-03 | 0.30 | -2.80E-02/8.20E-03 | 7.80E-04 | -3.30E-02/2.76E-02 | 0.25 |
| 250.24 | Type 2 diabetes with neurological manifestations | endocrine/metabolic | -2.18E-03/1.08E-02 | 0.84 | -1.20E-02/1.19E-02 | 0.33 | 2.40E-02/4.12E-02 | 0.58 |
| 250.4 | Abnormal glucose | endocrine/metabolic | -2.26E-03/9.97E-03 | 0.82 | -8.60E-03/1.12E-02 | 0.44 | -2.20E-02/3.76E-02 | 0.58 |
| 250.7 | Diabetic retinopathy | endocrine/metabolic | -7.19E-03/7.43E-03 | 0.34 | -2.60E-02/8.30E-03 | 2.10E-03 | -3.60E-02/2.72E-02 | 0.21 |
| 251 | Other disorders of pancreatic internal secretion | endocrine/metabolic | 2.02E-03/8.76E-03 | 0.82 | -1.40E-02/8.90E-03 | 0.11 | 1.10E-02/3.22E-02 | 0.74 |
| 251.1 | Hypoglycemia | endocrine/metabolic | 1.42E-03/8.80E-03 | 0.87 | -1.50E-02/8.90E-03 | 0.09 | 1.00E-02/3.22E-02 | 0.75 |
| 252 | Disorders of parathyroid gland | endocrine/metabolic | -4.31E-03/9.10E-03 | 0.64 | -5.70E-03/9.10E-03 | 0.53 | -3.70E-02/3.34E-02 | 0.29 |
| 252.1 | Hyperparathyroidism | endocrine/metabolic | -8.81E-03/9.30E-03 | 0.35 | -3.00E-03/9.60E-03 | 0.76 | -3.70E-02/3.54E-02 | 0.31 |
| 253 | Disorders of the pituitary gland and its hypothalamic control | endocrine/metabolic | 9.77E-03/9.87E-03 | 0.32 | -2.20E-02/1.08E-02 | 0.04 | -2.20E-02/3.75E-02 | 0.58 |
| 255 | Disorders of adrenal glands | endocrine/metabolic | -2.55E-02/1.03E-02 | 0.01 | -1.30E-02/1.06E-02 | 0.24 | 3.60E-02/3.97E-02 | 0.38 |
| 260 | Protein-calorie malnutrition | endocrine/metabolic | 1.97E-03/8.08E-03 | 0.81 | 3.20E-04/8.30E-03 | 0.97 | 2.50E-02/3.02E-02 | 0.42 |
| 260.6 | Anorexia | endocrine/metabolic | 9.57E-04/9.04E-03 | 0.92 | 3.50E-03/9.30E-03 | 0.71 | 3.80E-02/3.42E-02 | 0.29 |
| 261 | Vitamin deficiency | endocrine/metabolic | 5.93E-03/7.57E-03 | 0.44 | -2.90E-03/7.80E-03 | 0.70 | 1.20E-02/2.85E-02 | 0.68 |
| 261.2 | Vitamin B-complex deficiencies | endocrine/metabolic | 1.26E-03/9.73E-03 | 0.90 | 6.20E-04/9.80E-03 | 0.95 | -1.70E-02/3.62E-02 | 0.64 |
| 272 | Disorders of lipid metabolism | endocrine/metabolic | 9.80E-03/3.53E-03 | 0.01 | -5.70E-03/5.30E-03 | 0.29 | -3.70E-03/8.70E-03 | 0.68 |
| 272.1 | Hyperlipidemia | endocrine/metabolic | 9.65E-03/3.51E-03 | 0.01 | -5.80E-03/5.30E-03 | 0.28 | -3.50E-03/8.70E-03 | 0.69 |
| 272.11 | Hypercholesterolemia | endocrine/metabolic | 9.76E-03/3.54E-03 | 0.01 | -5.90E-03/5.30E-03 | 0.27 | -2.90E-03/9.10E-03 | 0.76 |
| 274 | Gout and other crystal arthropathies | endocrine/metabolic | -2.69E-03/4.71E-03 | 0.57 | -7.60E-03/5.40E-03 | 0.16 | 1.70E-02/1.63E-02 | 0.31 |
| 274.1 | Gout | endocrine/metabolic | -3.29E-03/5.06E-03 | 0.52 | -6.90E-03/6.20E-03 | 0.27 | 2.80E-02/1.75E-02 | 0.14 |
| 274.2 | Crystal arthropathies | endocrine/metabolic | -5.01E-04/1.05E-02 | 0.96 | -1.20E-02/1.08E-02 | 0.27 | -3.70E-02/3.96E-02 | 0.38 |
| 274.21 | Chondrocalcinosis | endocrine/metabolic | 1.85E-03/1.11E-02 | 0.87 | -9.50E-03/1.14E-02 | 0.41 | -3.60E-02/4.17E-02 | 0.41 |
| 275 | Disorders of mineral metabolism | endocrine/metabolic | -1.78E-02/2.44E-02 | 0.47 | -2.10E-02/2.21E-02 | 0.34 | -1.50E-02/2.15E-02 | 0.50 |
| 275.1 | Disorders of iron metabolism | hematopoietic | -3.73E-02/2.13E-02 | 0.08 | -4.30E-02/1.99E-02 | 0.03 | -2.00E-02/4.05E-02 | 0.63 |
| 275.5 | Disorders of calcium/phosphorus metabolism | endocrine/metabolic | 7.76E-03/7.53E-03 | 0.30 | 2.10E-03/7.90E-03 | 0.79 | -1.40E-02/2.85E-02 | 0.62 |
| 276 | Disorders of fluid, electrolyte, and acid-base balance | endocrine/metabolic | 2.63E-03/3.11E-03 | 0.40 | 1.90E-03/3.60E-03 | 0.61 | -3.10E-03/1.17E-02 | 0.79 |
| 276.1 | Electrolyte imbalance | endocrine/metabolic | -1.37E-03/4.32E-03 | 0.75 | 2.50E-03/4.20E-03 | 0.56 | -6.70E-04/1.55E-02 | 0.97 |
| 276.13 | Hyperpotassemia | endocrine/metabolic | -5.85E-03/8.66E-03 | 0.50 | -2.90E-03/8.70E-03 | 0.74 | 9.60E-03/3.16E-02 | 0.77 |
| 276.14 | Hypopotassemia | endocrine/metabolic | 1.09E-03/6.92E-03 | 0.88 | 6.20E-03/7.10E-03 | 0.38 | -8.30E-03/2.76E-02 | 0.77 |
| 276.4 | Acid-base balance disorder | endocrine/metabolic | -8.13E-03/7.66E-03 | 0.29 | -7.50E-03/7.90E-03 | 0.34 | -1.60E-02/2.90E-02 | 0.59 |
| 276.41 | Acidosis | endocrine/metabolic | -1.09E-02/8.04E-03 | 0.18 | -5.10E-03/8.30E-03 | 0.54 | -1.40E-02/3.04E-02 | 0.65 |
| 276.5 | Hypovolemia | endocrine/metabolic | 8.51E-03/4.94E-03 | 0.09 | 8.90E-04/5.50E-03 | 0.87 | 7.80E-03/1.87E-02 | 0.69 |
| 277 | Other disorders of metabolism | endocrine/metabolic | 1.16E-02/6.95E-03 | 0.10 | 5.70E-04/7.50E-03 | 0.94 | -3.80E-03/2.62E-02 | 0.89 |
| 278 | Overweight, obesity and other hyperalimentation | endocrine/metabolic | 2.46E-03/3.06E-03 | 0.42 | 9.70E-04/3.00E-03 | 0.75 | -3.90E-03/9.90E-03 | 0.70 |
| 278.1 | Obesity | endocrine/metabolic | 2.34E-03/3.09E-03 | 0.45 | 1.90E-03/3.10E-03 | 0.55 | -3.30E-03/9.70E-03 | 0.74 |
| 280 | Iron deficiency anemias | hematopoietic | 4.89E-03/3.60E-03 | 0.18 | 4.30E-03/3.30E-03 | 0.19 | -1.10E-03/1.15E-02 | 0.93 |
| 280.1 | Iron deficiency anemias, unspecified or not due to blood loss | hematopoietic | 4.89E-03/3.73E-03 | 0.19 | 3.40E-03/3.50E-03 | 0.33 | -1.40E-03/1.17E-02 | 0.91 |
| 281 | Other deficiency anemia | hematopoietic | -7.86E-03/9.10E-03 | 0.39 | -8.20E-03/8.50E-03 | 0.34 | 7.80E-03/2.93E-02 | 0.79 |
| 281.1 | Megaloblastic anemia | hematopoietic | -7.33E-03/9.17E-03 | 0.43 | -6.10E-03/8.70E-03 | 0.49 | 5.50E-03/2.99E-02 | 0.86 |
| 281.11 | Pernicious anemia | hematopoietic | -1.48E-02/1.19E-02 | 0.21 | -7.30E-03/1.07E-02 | 0.50 | -9.70E-03/3.62E-02 | 0.79 |
| 285 | Other anemias | hematopoietic | 2.10E-03/2.82E-03 | 0.46 | -3.90E-04/3.00E-03 | 0.90 | -3.00E-03/9.30E-03 | 0.75 |
| 285.2 | Anemia of chronic disease | hematopoietic | 6.27E-04/1.01E-02 | 0.95 | -2.70E-02/1.01E-02 | 0.01 | -1.70E-02/3.69E-02 | 0.65 |
[truncated: 92,629 more chars]
